# Supplementary material for: Human consumption of seaweed and freshwater aquatic plants in ancient Europe
Source: Nat Commun. 2023 Oct 17;14:6192. doi: 10.1038/s41467-023-41671-2 (PMC10582258; doi:10.1038/s41467-023-41671-2)
Supplement: Supplementary file 1 — Supplementary Information [file 41467_2023_41671_MOESM1_ESM.pdf]

Supplementary data

Chemical analysis of dental calculus using sequential thermal desorption-gas chromatography-mass spectrometry and pyrolysis-gas chromatography-mass spectrometry, full results

Contents

Supplementary Table of results……….…………………………………………………...7

Supplementary Notes…………………………………………………………………..14

Detailed Results: Scotland…………………………………………………..14

Isbister, Orkney Introduction………………………………………..……..14

Isbister DL42…………………………………………..………………..14

Isbister DL2104…………………………………………..……………..18

Isbister DL52…………………………………………..………………..22

Isbister DL86…………………………………………..………………..22

Isbister DL143…………………………………………..………………25

Isbister DL168…………………………………………..………………27

Isbister DL170…………………………………………..………………29

Isbister DL180…………………………………………..………………32

Isbister DL188…………………………………………..………………34

Isbister DL196…………………………………………..………………36

Isbister DL715…………………………………………..………………38

Isbister DL5083…………………………………………..……………..42

Isbister DL5117…………………………………………..……………..45

Isbister DL5119…………………………………………..……………..46

Isbister DL5838…………………………………………..……………..49

Isbister Discussion…………………………………………..…………..54

Supplementary Table 2…………………………………………..…………..59

Supplementary Table 2…………………………………………..…………..61

Supplementary Table 3…………………………………..……………..63

National Museum of Scotland Human Remains Collection Summary…..……..68

Quanterness, Orkney Introduction……………………………………………69

Quanterness 1139-01……………………………………………………...69

Quanterness 1190-A?1……………………………………………………69

Quanterness 249-01………………………………………………………70

Quanterness 2730-2734…………………………………………………...73

Quanterness 2552-02……………………………………………………...73

Quanterness 1408…………………………………………………………76

Quanterness Discussion…………………………………………………...77

Distillery Cave, Oban, Highlands Introduction………………………………79

Distillery Cave ‘1’………………………………………………………...79

Distillery Cave ‘3’………………………………………………………...82

Distillery Cave Discussion………………………………………………..84

Raschoille Cave, Oban, Highland Introduction………………………………86

Raschoille Cave ORC III 24.1……………………………………………86

Raschoille Cave Discussion………………………………………………87

Boatbridge Quarry, Thankerton, S Lanarkshire Introduction…………………...87

Boatbridge Quarry cist 2 (associated with Beaker NMS X.EG 106)………..87

Boatbridge Quarry cist 2 (associated with Beaker NMS X.EG 106)………..89

Boatbridge Quarry, Thankerton Discussion………………………………91

Juniper Green, City of Edinburgh Introduction…………………………….....92

Juniper Green, City of Edinburgh (NMS X.ET 33)…………………………92

Juniper Green, City of Edinburgh Discussion……………………………….94
Ruchlaw Mains, East Lothian Introduction……………………………………...95
Ruchlaw Mains (NMS unreg)……………………………………………..95
Ruchlaw Mains, East Lothian Discussion……………………………………97
Skateraw (Roy Richie 1958 cist), East Lothian Introduction……………………98
(Roy Richie 1958 cist) (NMS unreg)……………………………………...98
Lesmurdie cist B, Banff Introduction……………………………………………99
Lesmurdie cist B, Banff (NMS X.ET 14) ……………………………………99
Detailed results, Lithuania…………………………….……………………...100
Kretuonas Introduction……………………………………………………...101
Kretuonas SA1……………………………………………………………101
Donkalnis and Spiginas Introduction………………………………………..101
Donkalnis SA4……………………………………………………………....102
Spiginas SA5……………………………………………………………...102
Spiginas SA6……………………………………………………………...103
Spiginas SA7……………………………………………………………...105
Mesolithic and Neolithic, Lithuania Discussion……………………………...105
Obeliai, Central Lithuania flat burials. People’s Wandering Introduction….107
Obeliai, Central Lithuania flat burials. 1A5………………………………...108
Obeliai, Central Lithuania flat burials. 1A6………………………………...110
Obeliai, Central Lithuania flat burials. 1A8………………………………...111
Obeliai, Central Lithuania flat burials. 1A9………………………………...113
Obeliai, Central Lithuania flat burials. 1A10………………………………117
Obeliai, Central Lithuania flat burials. People’s Wandering Discussion……….118
Pagrybis, North Lithuanian Barrows. People’s Wandering Introduction……….123

77 Pagrybis, North Lithuanian Barrows. 1A17……………………………….123
78 Pagrybis, North Lithuanian Barrows. 1A18……………………………….124
79 Pagrybis, North Lithuanian Barrows. 1A19……………………………….124
80 Pagrybis, North Lithuanian Barrows. 1A20……………………………….126
81 Pagrybis, North Lithuanian Barrows. 1A21……………………………….127
82 Pagrybis, North Lithuanian Barrows. People's Wandering Discussion……….129
83 Vėluikiai, North Lithuanian Barrows. People's Wandering Introduction……..129
84 Vėluikiai, North Lithuanian Barrows. 1A28……………………………….130
85 Vėluikiai, North Lithuanian Barrows. People's Wandering Discussion…..130
86 Pašatrijis, North Lithuanian Barrows. Roman period Introduction……………131
87 Pašatrijis, North Lithuanian Barrows. 1A29……………………………….131
88 Pašatrijis, North Lithuanian Barrows. 1A30……………………………… 132
89 Pašatrijis, North Lithuanian Barrows. Roman period Discussion…………133
90 Jakštaičiai-Meškiai, North Lithuanian Barrows. Viking period Introduction….134
91 Jakštaičiai-Meškiai, North Lithuanian Barrows. 1A31…………………….134
92 Jakštaičiai-Meškiai, North Lithuanian Barrows. 1A32…………………….135
93 Jakštaičiai-Meškiai, North Lithuanian Barrows. 1A33…………………….138
94 Jakštaičiai-Meškiai, North Lithuanian Barrows. Viking period Discussion……141
95 Detailed Results: Kanaljorden, Sweden………………..…………………….143
96 Kanaljorden, Sweden Mesolithic Introduction…………………………143
97 Kanaljorden 122……………………………………………………...143
98 Kanaljorden 126……………………………………………………...145
99 Kanaljorden, Sweden Mesolithic Discussion…………………………...146
100 Detailed Results: Iberia……………………….……………………..….146
101 La Corona (Casa Corona), Alicante, Spain Introduction……………….146

La Corona 25……………………………………………………………147
La Corona 2……………………………………… …………………149
La Corona 4…………………………………………………………… .150
La Corona sediment (control)…………………………………………… …151
La Corona Discussion……………………………………………….....151
Bòbila Madurell, Catalonia, Spain Introduction……………………...154
Bòbila Madurell, Catalonia MF18………………………………..154
Bòbila Madurell Discussion…………………………………………155
Muge and Sado, Portugal Introduction…………………………………...155
Moita do Sebastião, Muge: MSD……………………………………….156
Moita do Sebastião, Muge: MSY……………………………………….156
Moita do Sebastião, Muge: MS7.3……………………………………..156
Moita do Sebastião, Muge: MS29……………………………………...157
Moita do Sebastião, Muge: MS32……………………………………...157
Moita do Sebastião, Muge: MS56……………………………………...158
Moita do Sebastião, Muge: MS60……………………………………...158
Moita do Sebastião, Muge Discussion……………………………....158
Cabeço da Arruda, Muge: ARR XIII (113) ……………………………159
Cabeço da Arruda, Muge Discussion…………………………………..160
Cabeço do Pez, Sado Valley: b251-b…………………………………..161
Cabeço do Pez, Sado Valley: Esq27…………………………………...163
Cabeço do Pez, Sado Valley: 6250-D…………………………….....164
Cabeço do Pez, Sado Valley Discussion……………………………....165
Detailed Results: Netherlands…………………………………………….....169
Schipluiden and Hardinxveld, Netherlands Introduction……………....169

Schipluiden Grave 1 Individual 1 TMS0081……………………….……170

Schipluiden Grave 1 Individual 2 TMS0077 (PM1)……………….… ...173

Schipluiden Grave 1 Individual 2 TMS0077 2 (incisor 1-2)……….……174

Schipluiden Grave 1 Individual 2 TMS0077 (M2-3…………………..175

Schipluiden soil (control) from Grave 1 (for Individuals 1 and 2).…...177

Schipluiden Grave 3 TMS0058 (lower labial molar)…………………177

Schipluiden Grave 3 TMS0058 (upper labial molar)…….………..…...180

Schipluiden Grave 5 child TMS0051……..……….…………….….…183

Schipluiden soil (control) from Grave 5 (for child).….…………….…183

Schipluiden Burials Discussion………….…………..…….……...185

Hardinxveld de Bruin, Brui 0011014 p.22 VI.35: Elvis F1004…..……186

Hardinxveld de Bruin soil (control) from Brui 0011014 p.22 VI.35…..188

Hardinxveld de Bruin (Meso), Box 0308…….…………..………….……189

Hardinxveld de Bruin soil (control) from burial (Meso) Box 0308.…...189

Hardinxveld Trintjuite, Box 0279 F1004 Put 25……….….…………...190

Hardinxveld de Bruin Burials Discussion……………….……….…...190

Detailed Results: Kruszyn, Poland……………………………………..192

Kruszyn, Poland Introduction…….………….….……..………….……….192

Kruszyn, Poland: Grave G93, Site 13….…….……….……….………......192

Kruszyn Discussion…….…………….…..………….……………….……193

Detailed Results: Rønstenen, Denmark…………….……………….......193

Rønstenen, Denmark Introduction…….…………….……..…….………193

Rønstenen, Denmark: AS 15/06….……….……….………………......193

Rønstenen Discussion…….…………….……………..…….…….......194

Supplementary References…………………………………..…………………194

Supplementary Table 1. Summary of results, all samples.

Sample number	Sample name	Details	Location	Age	Results
1	Isbister	DL42 M40-50 Sk8 SC1 ST2	Orkney, Scotland	Middle to Late Neolithic 3200–2800 BC	Seaweed, meat/fish/dairy exposure to fire/cooking
2	Isbister	DL2104 M17-23 Sk11 BC8/31 SC3 IS1976	Orkney, Scotland	Middle to Late Neolithic 3200–2800 BC	Seaweed, meat/fish/dairy exposure to fire/cooking
3	Isbister	DL52.14 Sk78 BC5 IS113	Orkney, Scotland	Middle to Late Neolithic 3200–2800 BC	Highly oxidised organic material (trace)
4	Isbister	DL86 Sk78 BC4 IS115	Orkney, Scotland	Middle to Late Neolithic 3200–2800 BC	Seaweed, meat/fish/dairy
5	Isbister	DL143 1-2 Sk78 SC3	Orkney, Scotland	Middle to Late Neolithic 3200–2800 BC	Non-specific protein and lipid biomarkers
6	Isbister	DL168 Sk78 SC1 ST2 mandible IS1958	Orkney, Scotland	Middle to Late Neolithic 3200–2800 BC	Non-specific protein and lipid biomarkers
7	Isbister	DL170 Sk78 SC1 ST2	Orkney, Scotland	Middle to Late Neolithic 3200–2800 BC	Seaweed, meat/fish/dairy
8	Isbister	DL180 Sk78 BC2 (a+b) SC1 ST2 mandible IS1958	Orkney, Scotland	Middle to Late Neolithic 3200–2800 BC	Meat/fish/dairy
9	Isbister	DL188 1-3 Sk78 BC7 jaw fragment IS1976	Orkney, Scotland	Middle to Late Neolithic 3200–2800	Non-specific protein and lipid biomarkers

				BC	
10	Isbister	DL196 5 BC4 ST4 ISB incisor IS1976	Orkney, Scotland	Middle to Late Neolithic 3200–2800 BC	Non-specific protein, lipid and carbohydrate biomarkers
11	Isbister	DL715 24 Sk77 BC4 mandible IS1976	Orkney, Scotland	Middle to Late Neolithic 3200–2800 BC	Seaweed (red), meat/fish/dairy
12	Isbister	DL5083 x3 Sk77 BC2c IS128 mandible IS1976	Orkney, Scotland	Middle to Late Neolithic 3200–2800 BC	Seaweed, meat/fish/dairy
13	Isbister	DL5117 x3 Sk77 BC6(3) ST5 mandible 5 IS1976	Orkney, Scotland	Middle to Late Neolithic 3200–2800 BC	Highly oxidised organic material (trace)
14	Isbister	DL5119 x4 Sk77 BC6(3) ST5 mandible 2 IS1976	Orkney, Scotland	Middle to Late Neolithic 3200–2800 BC	Seaweed, meat/fish/dairy
15	Isbister	DL5838 Sk77 BC5 IS113 incisor IS1976	Orkney, Scotland	Middle to Late Neolithic 3200–2800 BC	Seaweed (red), meat/fish/dairy, higher plant wax (e.g. from leafy greens) exposure to fire/cooking
16	Quanterness	Bag 10/Box 4 Bag 209 (bag 4) 1139-01: M1 right lingual	Orkney, Scotland	Middle to Late Neolithic c. 3200–2800 BC	Highly oxidised organic material (trace)
17	Quanterness	Box 10/Box L/Bag 216 (bag 2) 1190-A?1: M2 left labial	Orkney, Scotland	Middle to Late Neolithic c. 3200–2800 BC	Highly oxidised organic material (trace)
18	Quanterness	Box 10/Box K/bag 203 (Bag 3) 249-01: M2 right lingual	Orkney, Scotland	Middle to Late Neolithic c. 3200–2800 BC	Seaweed (red)
19	Quanterness	Box 20 (Box A Bag 666 Bag 1 or 4) 2730-2734 (mandible split):	Orkney, Scotland	Neolithic Middle to Late Neolithic c.	Highly oxidised organic material

		M1 left lingual		3200–2800 BC	
20	Quanterness	Box 20/Bag 675 (Box A: Bag 2) 2552-02: M2 right lingual	Orkney, Scotland	Neolithic Middle to Late Neolithic c. 3200–2800 BC	Seaweed (red)
21	Quanterness	Box 19/Box F/Bag 663 from skull 1408: Loose maxillary molar Lingual	Orkney, Scotland	Neolithic Middle to Late Neolithic c. 3200–2800 BC	Highly oxidised organic material
22	Distillery Cave	Box 6, adult male '1': Canine right lingual	Oban, West Scotland	Early Neolithic c. 3700 BC	Seaweed (red)
23	Distillery Cave	Box 6, prob male '3': PM2 right labial	Oban, West Scotland	Early Neolithic c. 3700 BC	Seaweed
24	Raschoille Cave	ORC III 24.1: M1 right lingual	Oban, West Scotland	Neolithic 4000-2900 cal BC	Highly oxidised organic material
25	Boatbridge Quarry	Cist 2, Thankerton, S Lanarkshire, PM2 left labial S1	Central Scotland	Chalcolithic /Early Bronze Age 2460–2140 cal BC	Submerged freshwater aquatic plant (*Nymphea spp.*)<br>exposure to fire/cooking
26	Boatbridge Quarry	Cist 2, Thankerton, S Lanarkshire, M2 right labial S2	Central Scotland	Chalcolithic /Early Bronze Age 2460–2140 cal BC	Submerged freshwater aquatic plant (*Nymphea spp.)*<br>exposure to fire/cooking
27	Juniper Green	(NMS X.ET 33): M3 right back of tooth	Central Scotland	Chalcolithic /Early Bronze Age 2335–2135 cal BC	Submerged freshwater aquatic plant; (*Nymphea spp.)*
28	Ruchlaw Mains	PM1,2 right lingual	East Scotland	Chalcolithic /Early Bronze Age 2340–2140 cal BC	Bacterially-derived biomarkers<br>exposure to fire/cooking
29	Skateraw	(Roy Richie 1958 cist),		2340–2195 cal BC	Highly oxidised organic material
30	Lesmurdie	cist B, Banff (NMS X.ET 14): R mandibular 2nd	East Scotland	2295–2040 cal BC	Highly oxidised organic material

		molar, labial			
31	Kretuonas	SA1	Lithuania	Neolithic	Highly oxidised organic material (trace)
32	Donkalnis	SA4	Lithuania	Neolithic	Highly oxidised organic material
33	Spiginas	SA5	Lithuania	Mesolithic	Submerged freshwater aquatic plant (*Nymphea spp.*)
34	Spiginas	SA6	Lithuania	Neolithic	Non-specific lipid (bacterial?) biomarkers
35	Gyvakarai	SA7	Lithuania	Neolithic/Bronze Age	Highly oxidised organic material
36	Obeliai	Sk1128 male 50-55 yrs T32 labial, 1A5	Lithuania	AD 5th-6th century	Submerged freshwater aquatic plant (*Nymphea spp.*)<br>exposure to fire/cooking
37	Obeliai	Sk1132 female 30-35 yrs T41 lingual, 1A6	Lithuania	AD 5th-6th century	Non-specific (bacterial?) lipid biomarkers
38	Obeliai	Sk1289 male 30-35 yrs T32 labial, 1A8	Lithuania	AD 5th-6th century	Freshwater micro/macroalgae
39	Obeliai	Sk1294 male 50-55 yrs T43 lingual, 1A9	Lithuania	AD 5th-6th century	Freshwater micro/macroalgae, meat/fish/dairy
40	Obeliai	Sk0984 female >55 yrs T33 lingual, 1A10	Lithuania	AD 5th-6th century	Submerged freshwater aquatic plant (*Nymphea spp.*)
41	Pagrybis	Sk1410A female 40-45 yrs T43 labial, 1A17	Lithuania	AD 5th-6th century	No detectable organic compounds
42	Pagrybis	, Sk1409C male 30-35 yrs T31 labial, 1A18	Lithuania	AD 5th-6th century	Highly oxidised organic material
43	Pagrybis	Sk1402 female 45-50 yrs T42 labial, 1A19	Lithuania	AD 5th-6th century	Non-specific (bacterial?) lipid biomarkers
44	Pagrybis	Sk1398 male >55 yrs T27 bucal, 1A20	Lithuania	AD 5th-6th century	Non-specific (bacterial?) lipid biomarkers
45	Pagrybis	Sk1058 male 30-35 yrs T41 labial, 1A21	Lithuania	AD 5th-6th century	Non-specific (bacterial?) lipid biomarkers
46	Vėluikiai	Sk1421 female 25-30 yrs T46 lingual, 1A28	Lithuania	AD 5th-6th century	Highly oxidised organic material
47	Pašatrijis	Sk7224 male 40-45 yrs T27 bucal, 1A29	Lithuania	AD 1st-3th century)	Non-specific (bacterial?) lipid biomarkers
48	Pašatrijis	Sk7227 male 20-25	Lithuania	AD 1st-3th	Highly oxidised organic

		yrs T32 labial, 1A30		century	material
49	Jakštaičiai-Meškia	Sk1388B male 20-25 yrs T36 lingual, 1A31	Lithuania	AD 7th-12th century	Non-specific (bacterial?) lipid biomarkers
50	Jakštaičiai-Meškia	Sk1386 female 20-25 yrs T46 lingual, 1A32	Lithuania	AD 7th-12th century	Freshwater micro/macroalgae
51	Jakštaičiai-Meškia	Sk1382 male 35-40 yrs T36 lingual, 1A33	Lithuania	AD 7th-12th century	Freshwater micro/macroalgae
52	Kanaljorden	122	Sweden	Mesolithic 5880–5620 cal BC	Meat/fish
53	Kanaljorden	126	Sweden	Mesolithic 5710–5570 cal BC	Meat/fish<br>exposure to fire/cooking
54	La Corona	25	Spain	Mesolithic	Seaweed, meat/fish
55	La Corona	2 M1 mfieq (lingual)	Spain	Mesolithic	Exposure to fire/cooking
56	La Corona	La Corona 4 M3 mfdch (lingual)	Spain	Mesolithic	Exposure to fire/cooking
57	Bòbila Madurell,	MF18 male	Catalonia, Sain	Neolithic	Highly oxidised organic material (trace)
58	Moita do Sebastião, Muge	MSD male	Porugal	Mesolithic	Highly oxidised organic material (trace)
59	Moita do Sebastião, Muge	MSY female	Portugal	Mesolithic	Highly oxidised organic material (trace)
60	Moita do Sebastião, Muge	MS7.3 male	Portugal	Mesolithic	Highly oxidised organic material (trace)
61	Moita do Sebastião, Muge	MS29 child F.1-6 (female)	Portugal	Mesolithic	No detectable organic compounds
62	Moita do Sebastião, Muge	MS32 male	Portugal	Mesolithic	Highly oxidised organic material (trace)
63	Moita do Sebastião, Muge	MS56 male	Portugal	Mesolithic	Highly oxidised organic material (trace)
64	Moita do Sebastião, Muge	MS60 female	Portugal	Mesolithic	No detectable organic compounds
65	Cabeço da Arruda, Muge, Portugal:	ARR XIII, female	Portugal	Mesolithic	Beeswax

	ARR XIII				
66	Cabeço do Pez, Sado Valley	B251-b female	Portugal	Mesolithic	Submerged freshwater aquatic plant (*Potamogeton* spp. pondweed)
67	Cabeço do Pez, Sado Valley		Portugal	Mesolithic	Submerged freshwater aquatic plant (*Potamogeton* spp. pondweed)
68	Cabeço do Pez, Sado Valley	6250-D Esquina A-B Jordi male	Portugal	Mesolithic	Submerged freshwater aquatic plant (*Potamogeton* spp. pondweed)
69	Schipluiden	Grave 1 Individual 1 TMS0081: maxilla labial molar	Netherlands	Neolithic	Non-specific (bacterial?) lipid biomarkers exposure to fire/cooking
70	Schipluiden	Grave 1 Individual 2 TMS0077: mandible labial right PM1	Netherlands	Neolithic	Non-specific protein and lipid biomarkers
71	Schipluiden	Grave 1 Individual 2 TMS0077: maxilla/mandible? lingual left incisor 1-2	Netherlands	Neolithic	Non-specific protein and lipid biomarkers
72	Schipluiden	Grave 1 Individual 2 TMS0077: maxilla/mandible? lingual right M2-3	Netherlands	Neolithic	Non-specific protein and lipid biomarkers exposure to fire/cooking
73	Schipluiden	Grave 3 TMS0058: lower jaw-mandible labial molar	Netherlands	Neolithic	Non-specific protein and lipid biomarkers exposure to fire/cooking
74	Schipluiden	Grave 3 TMS0058: upper jaw-maxilla labial molar	Netherlands	Neolithic	Non-specific protein and lipid biomarkers exposure to fire/cooking
75	Schipluiden	Grave 5 child TMS0051: maxilla labial PM1	Netherlands	Neolithic	Highly oxidised organic material
76	Hardinxveld - Giessendam De Bruin	Grave 2 ('Elvis'), S22-1004, find nr. 11.014 p.22 VI.35: (male)	Netherlands	Late Mesolithic c. 5200-4970 cal BC	Non-specific protein and lipid biomarkers exposure to fire/cooking
77	Hardinxveld - Giessendam De Bruin	Grave 1 ('Henk'), S21-1021, find nr. 15.165 mandible lingual right PM1(male)	Netherlands	Late Mesolithic (5500-4900 cal. BC)	No detectable organic compounds
78	Hardinxveld Giessendam Polderweg	Grave 1 ('Trijntje'), S25-1004) mandible incisor lingual	Netherlands	Late Mesolithic (c. 5500-4900 cal.	Highly oxidised organic material (trace)

		(female)		BC)	
79	Kruszyn	Site 13, Grave G93, male 35-40/45 years old, Linear Band Pottery culture (LBK)	Poland	Neolithic	Highly oxidised organic material
80	Rønstenen	AS 15/06 tandsten	Denmark	Mesolithic	Highly oxidised organic material

Detailed Results

Scotland

Isbister chambered cairn, Orkney Islands

Isbister is a chambered cairn on the island of South Ronaldsay, Orkney, North Scotland containing the disarticulated remains of around 85 individuals[1,2]. It was built during the late fourth millennium and remained in funerary use into the third millennium[3]. It is located in one of Europe's richest Neolithic landscapes combining numerous chambered cairns, two stone circles, the stone-built Late Neolithic village of Skara Brae and the huge ceremonial settlement of Ness of Brodgar, whose *floruit* was c. 3200–2800 BC[4]. Wild resources including bones from inshore and deep-water fish, birds and wild plants were found at some of these sites, in addition to bones of domesticated animals.

Fifteen samples of dental calculus taken from fifteen individuals from Isbister were analysed by sequential thermal desorption-gas chromatography-mass spectrometry (TD-GC-MS) and pyrolysis-gas chromatography-mass spectrometry (Py-GC-MS). This technique facilitates the identification of both free/unbound and bound/polymeric organic components. All fifteen calculus samples produced very little free organic material, with only three samples containing free compounds if the essentially ubiquitous and thermally generated carbon dioxide is excluded. In contrast, thirteen of the fifteen samples revealed a minor to significant amount of a bound/polymeric organic constituent.

1. Isbister DL42 M40-50 Sk8 SC1 ST2 (calculus: 2.57 mg)

The thermal desorption total ion chromatogram (TIC) revealed carbon dioxide and sulphur dioxide as the only detectable components, indicating the virtual absence of free, thermally extractable organic components in this sample. It should be noted, however, that highly polar material could have been present, which would not have successfully eluted from the column, or indeed volatilised sufficiently in the probe. It is not possible to determine the significance of the carbon dioxide, beyond it being indicative of oxidised organic material. The origin of the sulphur dioxide is also difficult to determine and its absence in other samples prevents any meaningful interpretation here.

In contrast to the thermal desorption profile, the pyrolysis-GC-MS TIC (Fig. 1) is dominated by a complex suite of organic compounds. These include a series of C_8 to C_{18} *n*-1-alkenes and

n-alkanes, typical of unsaturated and saturated fat/oil-derived acyl lipids[5,6]. A bimodal distribution with maxima at C_{12} and C_{14} suggests the original acyl lipids may have been high in polyunsaturated fatty acids. The absence of these compounds in the TD profile provides molecular evidence for a polymeric/bound acyl lipid source. However, in addition to *n*-1-alkenes and *n*-alkanes, typical of fat/oil derived lipids, the pyrogram also displayed a series of C_{10} to C_{16} iso-methyl-1-alkenes (isoalkenes) and 2-methylalkanes (isoalkanes), presumably deriving from the branched fatty acids which are significant components of bacterial triglycerides.

There were components identified in the pyrolysate TIC which can be indicative of 'black carbon', i.e. charcoal or soot[7-10]. However, although this included the presence of naphthalenes and biphenyls, the dominance of toluene, rather than the normally dominant aromatic hydrocarbon benzene, is notably atypical of chars[7-10]. The markers identified here can also be indicative of proteins[6,11] and combined with the abundant related protein markers pyrrole, benzonitrile, benzyl nitrile (phenylacetonitrile), benzenepropanenitrile and indoles[6,11] identified this is likely to be the main origin of these compounds in this sample pointing to a substantial protein-derived component[6,11]. This is also corroborated by the presence of aliphatic nitriles (carbon numbers $C_{14:0}$, $C_{16:0}$ (max) and $C_{18:0}$) suggesting a lipid (fat/oil) in this sample which has combined with protein-derived nitrogen to produce these moieties[5]. Notably however, the naphthalenes and biphenyls, combined with the polynuclear aromatic hydrocarbon (PAH) phenanthrene (a combustion marker), provides tentative molecular evidence for exposure to fire/cooking. The proteinaceous origin for the aromatic markers is further corroborated by the significant abundance of phenol, 2-methylphenol (*o*-cresol), 4-methylphenol (*p*-cresol) (with the 4-methyl isomer being markedly predominant), and lesser amounts of 2,4-dimethylphenol and 4-ethylphenol, all of which, collectively, are indicative of the amino acid tyrosine[12-14]. With an absence of guaiacol itself (all isomers) and other guaiacol moieties characteristic of lignin[15,16], the possibility that the phenols may originate from wood or woody plant material can be excluded. Alkyl phenols have also been observed in wood smoke and smoked foods[17,18], however the absence of methoxyphenols, diagnostic of lignin-derived smoke, means this possibility can also be discounted. Taken together, the presence of toluene, styrene and phenylacetonitrile (benzyl nitrile) can indicate the amino acid phenylalanine[12,13]. In addition, the major constituents indole and 3-methylindole (skatole), with a lesser but significant amount of a C_2 indole (3-ethylindole?), were also identified, which are consistent with a tryptophan amino acid source[12,13,19,20]. Proteins rich in

the amino acids tyrosine, phenylalanine and tryptophan include meat, fish and dairy products, although the presence of bacterial markers means the possibility that at least a partial origin could be microbial cannot be excluded.

However, highly notable in this context is the additional presence of appreciable amounts of the more unusual 2- and 3-methylpyrroles, 2- and 3-ethylpyrroles, 2,4-, 2,3- and 3,4-dimethyl-pyrroles, 2-ethyl-3(?)-methylpyrrole, 4-ethyl-2-methylpyrrole, 3-ethyl-4-methylpyrrole (trace), 2,3,5-trimethylpyrrole, a propylmethylpyrrole, an ethyldimethylpyrrole and 4-ethyl-2,3-dimethylpyrrole (trace). Collectively, these specific C_1, C_2, C_3 and C_4 alkyl pyrroles suggest a significant tetrapyrrole/porphyrin-derived origin and are indicative of an algal source observed in fossils and expected in similarly diagenetically altered archaeological material[11,21,22]. Notable also in this context were the presence of the moderately abundant nitrogen containing aromatic compounds 1-H-pyrazole-4-carbonitrile, ethylcyanobenzene, 2-pyridinecarbonitrile, tolyl isocyanide (x2), a C_4 pyrimidine, along with pyridine and its methyl-, dimethyl- and trimethyl derivatives (these pyridines can result from the pyrolysis of polypeptides containing the amino acid alanine[23]). The presence here of the abundant protein markers and specifically the C_1 to C_4 alkyl pyrroles, combined with the particular lipid profile and specific carbohydrate markers (see below) identified, suggests the presence of a macroalgae, i.e. seaweed, in this sample[21,23,24] (see discussion).

The thermally-derived carbohydrate markers 2-methylfuran, 2-methyl-2-cyclopenten-1-one and 2,3-dimethyl-2-cyclopenten-1one were identified as moderately significant components, along with an unidentified carbohydrate marker. That they were absent in the TD profile points to their originating from the original polymeric carbohydrate source (e.g. starch)[25]. Although levoglucosan (1,6-anhydro-β-D-glucopyranose) is usually the major component in the thermally-derived products of carbohydrates analysed by TD/Py-GC-MS, its absence from the TD profile here is consistent with the removal of hydroxyl groups in the original carbohydrate polymer, so preventing its formation in the probe. The additional absence of the normally significant carbohydrate markers 2-hydroxy-3-methyl-2-cyclopenten-1-one and 3-hydroxy-2-methyl-2-cyclopenten-1-one observed in pure standard material (glycogen, starch and cellulose) would also support this interpretation. This shows a strong matrix effect likely to be due to the inorganic component of the calculus and the microflora in the mouth. This dehydration/dehydroxylation has also been observed in dog calculus and 19th century human calculus[6], where other markers, e.g. a prevalence of nitriles, with amides very minor, deriving from dehydrating conditions in the mouth of dogs and humans (though not sheep[6]) supporting

this view[6]. The presence of carbon dioxide is also consistent with a lipid, amino acid/protein and carbohydrate component in this sample. Notably, these findings did not reveal key biomarkers typically observed in soils[20,23]. It should also be noted that highly polar material could have been present, which would not have successfully eluted from the column, or indeed volatilised sufficiently in the probe.

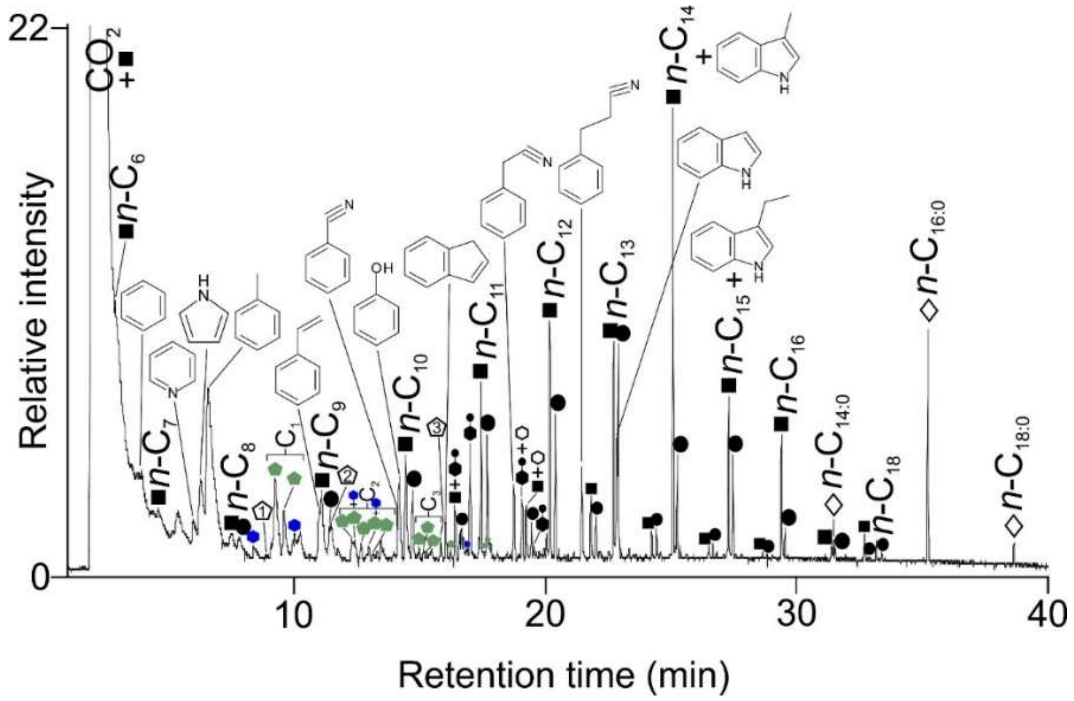

Supplementary Figure 1 Reconstructed total ion chromatogram of the pyrogram (pyrolysis profile) (610 °C for 10s) of sample DL42, after thermal desorption (310 °C for 10 s). Key to Fig. 1: Peak identities (x indicates carbon chain length): filled squares, Cx indicates alkenes; filled circles, Cx indicates alkanes; open diamonds, Cx:y indicates acyclic nitriles; blue filled hexagons indicates alkyl pyridines; green filled pentagons indicates alkyl pyrroles (with alkyl chain length above, Cx); filled hexagons with filled circles attached indicates alkyl phenols; open hexagons indicates alkyl indenes; open pentagons with numerals inside indicates carbohydrate pyrolysis markers, 1 is 2-methylfuran, 2 is 2-methyl-2-cyclopenten-1-one and 3 is 2,3-dimethyl-2-cyclopentene-1-one. Also shown are the structures of thirteen aromatic compounds identified: benzene, pyridine, pyrrole, toluene, styrene, benzonitrile, phenol, indene, benzyl nitrile, benzenepropanenitrile, indole, 3-methylindole (skatole) and 3-ethylindole. CO_2 indicates carbon dioxide.

2. Isbister DL2104 M17-23 Sk11 BC8/31 SC3 IS1976 (calculus: 3.19 mg)

The thermal desorption total ion chromatogram (TIC) (Fig. 2, inset) revealed two 2,5-diketopiperazine derivatives (DKPs). These were identified as the DKP derivative of proline-glycine (pro-gly), which was the major component, and the DKP of proline-alanine (pro-ala), which was present only as a relatively minor constituent. These are thermally generated in the pyroprobe from the amino acids proline and glycine, and proline and alanine respectively, and is indicative of proteinaceous material containing these three protein constituents[5,12,13]. Carbon dioxide was the only other compound observed indicating very little free, thermally extractable, organic material in this sample. It should be noted, however, that highly polar material could have been present, which would not have successfully eluted from the column, or indeed volatilised sufficiently in the probe. It is not possible to determine the significance of the carbon dioxide, beyond it being indicative of oxidised organic material.

In contrast to the thermal desorption profile, the pyrolysis-GC-MS TIC (Fig. 2) is dominated by a complex suite of organic compounds. These include a series of C_8 to C_{18} *n*-1-alkenes and *n*-alkanes, typical of unsaturated and saturated fat/oil-derived acyl lipids[5,6]. A bimodal distribution with maxima at C_{12} and C_{14} suggests the original acyl lipids may have been high in polyunsaturated fatty acids. The absence of these compounds in the TD profile provides molecular evidence for a polymeric/bound acyl lipid source. However, in addition to *n*-1-alkenes and *n*-alkanes, typical of fat/oil derived lipids, the pyrogram also displayed a series of C_{10} to C_{18} iso-methyl-1-alkenes (isoalkenes) and 2-methylalkanes (isoalkanes), presumably deriving from the branched fatty acids which are significant components of bacterial triglycerides.

There were components identified in the pyrolysate TIC which can be indicative of 'black carbon', i.e. charcoal or soot[7-10]. However, although this included the presence of naphthalenes, the dominance of toluene, rather than the normally dominant aromatic hydrocarbon benzene, is notably atypical of chars[7-10]. The markers identified here can also be indicative of proteins[6,11] and combined with the abundant related protein markers pyrrole, benzonitrile, benzyl nitrile (phenylacetonitrile), benzenepropanenitrile and indoles[6,11] identified this is likely to be the main origin of these compounds in this sample pointing to a substantial protein-derived component[6,11]. This is also corroborated by the presence of aliphatic nitriles (carbon numbers $C_{14:0}$, *i*-$C_{15:0}$, $C_{16:0}$ (max) *i*-$C_{17:0}$ and $C_{18:0}$) suggesting a lipid (fat/oil) in this sample which has combined with protein-derived nitrogen to produce

these moieties[5]. However, the naphthalenes and biphenyl (trace), combined with the polynuclear aromatic hydrocarbon (PAH) phenanthrene (trace; a combustion marker), provides very tentative molecular evidence for exposure to fire/cooking. The proteinaceous origin for the aromatic markers is further corroborated by the significant abundance of phenol, 2-methylphenol (*o*-cresol), 4-methylphenol (*p*-cresol) (with the 4-methyl isomer being markedly predominant), and lesser amounts of 2,4-dimethylphenol and 4-ethylphenol, all of which, collectively, are indicative of the amino acid tyrosine[12-14]. With an absence of guaiacol itself (all isomers) and other guaiacol moieties characteristic of lignin[15,16], the possibility that the phenols may originate from wood or woody plant material can be excluded. Alkyl phenols have also been observed in wood smoke and smoked foods[17,18], however the absence of methoxyphenols, diagnostic of lignin-derived smoke, means this possibility can also be discounted. Taken together, the presence of toluene, styrene and phenylacetonitrile (benzyl nitrile) can indicate the amino acid phenylalanine[12,13]. In addition, the major constituents indole and 3-methylindole (skatole), with a lesser but significant amount of a C_2 indole (3-ethylindole?), were also identified, which are consistent with a tryptophan amino acid source[12,13,19,20]. Proteins rich in the amino acids tyrosine, phenylalanine and tryptophan include meat, fish and dairy products, although the presence of bacterial markers means the possibility that at least a partial origin could be microbial cannot be excluded.

However, highly notable in this context is the additional presence of appreciable amounts of the more unusual 2- and 3-methylpyrroles, 2- and 3-ethylpyrroles, 2,4-, 2,3- and 3,4-dimethyl-pyrroles, 2-ethyl-3(?)-methylpyrrole, 4-ethyl-2-methylpyrrole, 3-ethyl-4-methylpyrrole (trace), 2,3,5-trimethylpyrrole, a propylmethylpyrrole, an ethyldimethylpyrrole (trace) and 4-ethyl-2,3-dimethylpyrrole (minor trace). Collectively, these specific C_1, C_2, C_3 and C_4 alkyl pyrroles suggest a significant tetrapyrrole/porphyrin-derived origin and are indicative of an algal source observed in fossils and expected in similarly diagenetically altered archaeological material[11,21,22]. Notable also in this context were the presence of the moderately abundant nitrogen containing aromatic compounds 1-H-pyrazole-4-carbonitrile, ethylcyanobenzene, 2-pyridinecarbonitrile, tolyl isocyanide (x2), a C_3 and C_4 pyrimidine, along with pyridine and its methyl-, dimethyl- and trimethyl derivatives (these pyridines can result from the pyrolysis of polypeptides containing the amino acid alanine[23]). The presence here of the abundant protein markers and specifically the C_1 to C_4 alkyl pyrroles, combined

with the particular lipid profile and specific carbohydrate markers (see below) identified, suggests the presence of a macroalgae, i.e. seaweed, in this sample[21,22,24] (see discussion).

The thermally-derived carbohydrate markers 2-methylfuran, 2-methyl-2-cyclopenten-1-one and 2,3-dimethyl-2-cyclopenten-1one were identified as moderately significant components, along with an unidentified carbohydrate marker. That they were absent in the TD profile points to their originating from the original polymeric carbohydrate source (e.g. starch)[25]. Although levoglucosan (1,6-anhydro-β-D-glucopyranose) is usually the major component in the thermally-derived products of carbohydrates analysed by TD/Py-GC-MS, its absence from the TD profile here is consistent with the removal of hydroxyl groups in the original carbohydrate polymer, so preventing its formation in the probe. The additional absence of the normally significant carbohydrate markers 2-hydroxy-3-methyl-2-cyclopenten-1-one and 3-hydroxy-2-methyl-2-cyclopenten-1-one observed in pure standard material (glycogen, starch and cellulose) would also support this interpretation. This shows a strong matrix effect likely to be due to the inorganic component of the calculus and the microflora in the mouth. This dehydration/dehydroxylation has also been observed in dog calculus and 19th century human calculus[6], where other markers, e.g. a prevalence of nitriles, with amides very minor, deriving from dehydrating conditions in the mouth of dogs and humans (though not sheep[6]) supporting this view[6]. The presence of carbon dioxide is also consistent with a lipid, amino acid/protein and carbohydrate component in this sample. Notably, these findings did not reveal key biomarkers typically observed in soils[20,23]. It should also be noted that highly polar material could have been present, which would not have successfully eluted from the column, or indeed volatilised sufficiently in the probe.

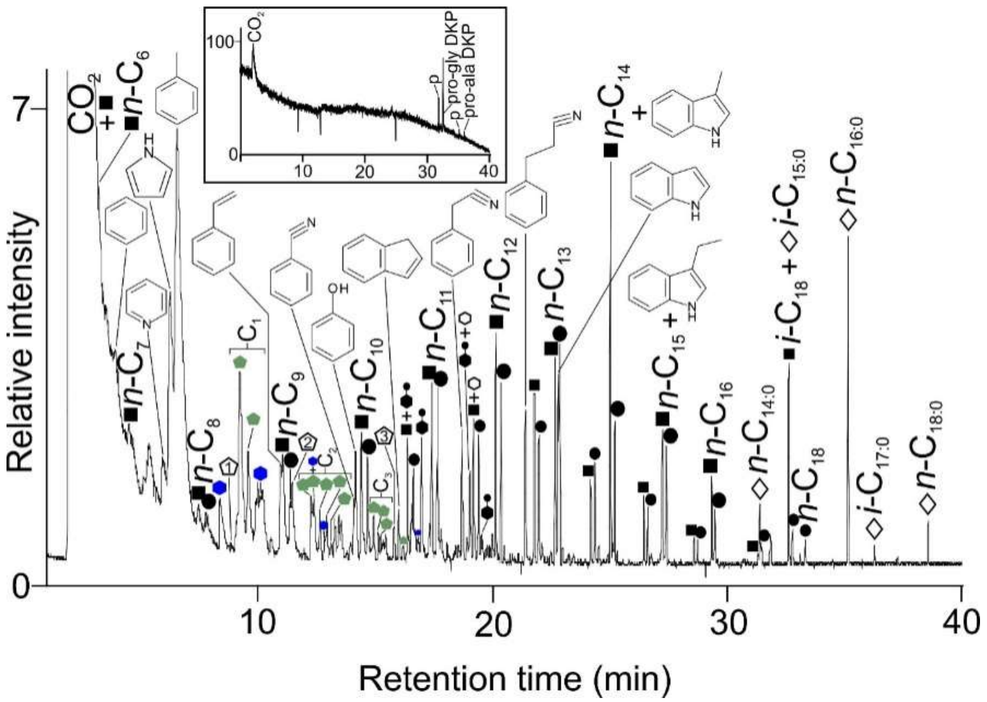

Supplementary Figure 2 Reconstructed total ion chromatogram of the pyrogram (pyrolysis profile) (610 °C for 10s) of sample DL2104, after thermal desorption (310 °C for 10 s). Key to Fig. 2: Peak identities (x indicates carbon chain length): filled squares, Cx indicates alkenes; filled circles, Cx indicates alkanes; open diamonds, Cx:y indicates acyclic nitriles; blue filled hexagons indicates alkyl pyridines; green filled pentagons indicates alkyl pyrroles (with alkyl chain length above, Cx); filled hexagons with filled circles attached indicates alkyl phenols; open hexagons indicates alkyl indenes; open pentagons with numerals inside indicates carbohydrate pyrolysis markers, 1 is 2-methylfuran, 2 is 2-methyl-2-cyclopenten-1-one and 3 is 2,3-dimethyl-2-cyclopentene-1-one. Also shown are the structures of thirteen aromatic compounds identified: benzene, pyridine, pyrrole, toluene, styrene, benzonitrile, phenol, indene, benzyl nitrile, benzenepropanenitrile, indole, 3-methylindole (skatole) and 3-ethylindole. CO_2 indicates carbon dioxide. Inset displays a reconstructed total ion chromatogram of the thermal desorption profile (310 °C for 10 s) of this sample. Peak identities: pro-gly DKP indicates the 2,5-diketopiperazine derivative of the amino acids proline and glycine and pro-ala indicates the 2,5-diketopiperazine derivative of the amino acids proline and alanine. CO_2 indicates carbon dioxide and p indicates contaminants from the pyroprobe.

3. Isbister DL52.14 Sk78 BC5 IS113 mandible IS1976 (calculus: 1.40 mg)

The thermal desorption total ion chromatogram (TIC) revealed carbon dioxide as the only detectable (trace) component, indicating the essential absence of free, thermally extractable organic components in this sample. It should be noted, however, that highly polar material could have been present, which would not have successfully eluted from the column, or indeed volatilised sufficiently in the probe. It is not possible to determine the significance of the carbon dioxide, beyond it being indicative of oxidised organic material.

The pyrolysate TIC revealed carbon dioxide and toluene as the only detectable (trace) components, indicating the essential absence of organic material in this sample. It should be noted, however, that highly polar material could have been present, which would not have successfully eluted from the column, or indeed volatilised sufficiently in the probe. It is not possible to determine the significance of the carbon dioxide, beyond it being indicative of oxidised organic material. The toluene is in too low an abundance to provide any meaningful interpretation of its presence, beyond it being indicative of an organic origin.

4. Isbister DL86 Sk78 BC4 IS115 mandible IS1976 (calculus: 4.89 mg)

The thermal desorption total ion chromatogram (TIC) revealed carbon dioxide and acetonitrile as the only detectable components, indicating the virtual absence of free, thermally extractable organic components in this sample. It should be noted, however, that highly polar material could have been present, which would not have successfully eluted from the column, or indeed volatilised sufficiently in the probe. It is not possible to determine the significance of the carbon dioxide, beyond it being indicative of oxidised organic material. The origin of the acetonitrile is also difficult to determine, beyond it being indicative of a relatively labile nitrogen-containing polymeric/bound biomolecule (hence the generation of acetonitrile at only ~300°C), although it has been correlated with the pyrolysis of proteins high in phenylalanine and tyrosine, in particular[26].

In contrast to the thermal desorption profile, the pyrolysis-GC-MS TIC (Fig. 3) is dominated by a relatively complex suite of organic compounds. These include a series of C_8 to C_{16} *n*-1-alkenes and *n*-alkanes, typical of unsaturated and saturated fat/oil-derived acyl lipids[5,6]. A bimodal distribution with maxima at C_{11}/C_{12} and C_{14} suggests the original acyl lipids may have been high in polyunsaturated fatty acids. The absence of these compounds in the TD profile provides molecular evidence for a polymeric/bound acyl lipid source. However, in

addition to *n*-1-alkenes and *n*-alkanes, typical of fat/oil derived lipids, the pyrogram also displayed a series of C_{10} to C_{15} iso-methyl-1-alkenes (isoalkenes) and 2-methylalkanes (isoalkanes), presumably deriving from the branched fatty acids which are significant components of bacterial triglycerides.

There were components identified in the pyrolysate TIC which can be indicative of 'black carbon', i.e. charcoal or soot[7-10]. However, lack of alkyl naphthalenes and biphenyls and the dominance of toluene, rather than the normally dominant aromatic hydrocarbon benzene, is notably atypical of chars[7-10]. The markers identified here can also be indicative of proteins[6,11] and combined with the abundant related protein markers pyrrole, benzonitrile, benzyl nitrile (phenylacetonitrile), benzenepropanenitrile and indoles[6,11] identified this is likely to be the main origin of these compounds in this sample pointing to a substantial protein-derived component[6,11]. This is also corroborated by the presence of the $C_{16:0}$ aliphatic nitrile, suggesting a lipid (fat/oil) in this sample which has combined with protein-derived nitrogen to produce these moieties[5]. The proteinaceous origin for the aromatic markers is further corroborated by the significant abundance of phenol, 2-methylphenol (*o*-cresol) and 4-methylphenol (*p*-cresol) (with the 4-methyl isomer being markedly predominant), which collectively are indicative of the amino acid tyrosine[12-14]. With an absence of guaiacol itself (all isomers) and other guaiacol moieties characteristic of lignin[15,16], the possibility that the phenols may originate from wood or woody plant material can be excluded. Alkyl phenols have also been observed in wood smoke and smoked foods[17,18], however the absence of methoxyphenols, diagnostic of lignin-derived smoke, means this possibility can also be discounted. Taken together, the presence of toluene, styrene and phenylacetonitrile (benzyl nitrile) can indicate the amino acid phenylalanine[12,13]. In addition, the major constituents indole and 3-methylindole (skatole) are consistent with a tryptophan amino acid source[12,13,19,20]. Proteins rich in the amino acids tyrosine, phenylalanine and tryptophan include meat, fish and dairy products, although the presence of bacterial markers means the possibility that at least a partial origin could be microbial cannot be excluded.

However, highly notable in this context is the additional presence of appreciable amounts of the more unusual 2- and 3-methylpyrroles, 2- and 3-ethylpyrroles, 2,3-dimethyl-pyrrole, 2-ethyl-3(?)-methylpyrrole, 4-ethyl-2-methylpyrrole, 3-ethyl-4-methylpyrrole, 2,3,5-trimethylpyrrole and a propylmethylpyrrole. Collectively, these specific C_1, C_2, C_3 and C_4 alkyl pyrroles suggest a tetrapyrrole/porphyrin-derived origin and are indicative of an algal source observed in fossils and expected in similarly diagenetically altered archaeological

material[11,21,22]. Notable also in this context were the presence of the relatively moderately abundant nitrogen containing aromatic compounds 1-H-pyrazole-4-carbonitrile, ethylcyanobenzene, 2-pyridinecarbonitrile and tolyl isocyanide (x2), along with pyridine and its methyl-, dimethyl- and trimethyl derivatives (these pyridines can result from the pyrolysis of polypeptides containing the amino acid alanine[23]). The presence here of the abundant protein markers and specifically the C_1 to C_4 alkyl pyrroles, combined with the particular lipid profile and specific carbohydrate markers (see below) identified, suggests the presence of a macroalgae, i.e. seaweed, in this sample[21,22,24] (see discussion).

The thermally-derived carbohydrate markers 2-methyl-2-cyclopenten-1-one and 2,3-dimethyl-2-cyclopenten-1one were identified as significant components, along with an unidentified carbohydrate marker. That they were absent in the TD profile points to their originating from the original polymeric carbohydrate source (e.g. starch)[25]. Although levoglucosan (1,6-anhydro-β-D-glucopyranose) is usually the major component in the thermally-derived products of carbohydrates analysed by TD/Py-GC-MS, its absence from the TD profile here is consistent with the removal of hydroxyl groups in the original carbohydrate polymer, so preventing its formation in the probe. The additional absence of the normally significant carbohydrate markers 2-hydroxy-3-methyl-2-cyclopenten-1-one and 3-hydroxy-2-methyl-2-cyclopenten-1-one observed in pure standard material (glycogen, starch and cellulose) would also support this interpretation. This shows a strong matrix effect likely to be due to the inorganic component of the calculus and the microflora in the mouth. This dehydration/dehydroxylation has also been observed in dog calculus and 19th century human calculus[6], where other markers, e.g. a prevalence of nitriles, with amides very minor, deriving from dehydrating conditions in the mouth of dogs and humans (though not sheep[6]) supporting this view[6]. The presence of carbon dioxide is also consistent with a lipid, amino acid/protein and carbohydrate component in this sample. Notably, these findings did not reveal key biomarkers typically observed in soils[20,23]. It should also be noted that highly polar material could have been present, which would not have successfully eluted from the column, or indeed volatilised sufficiently in the probe.

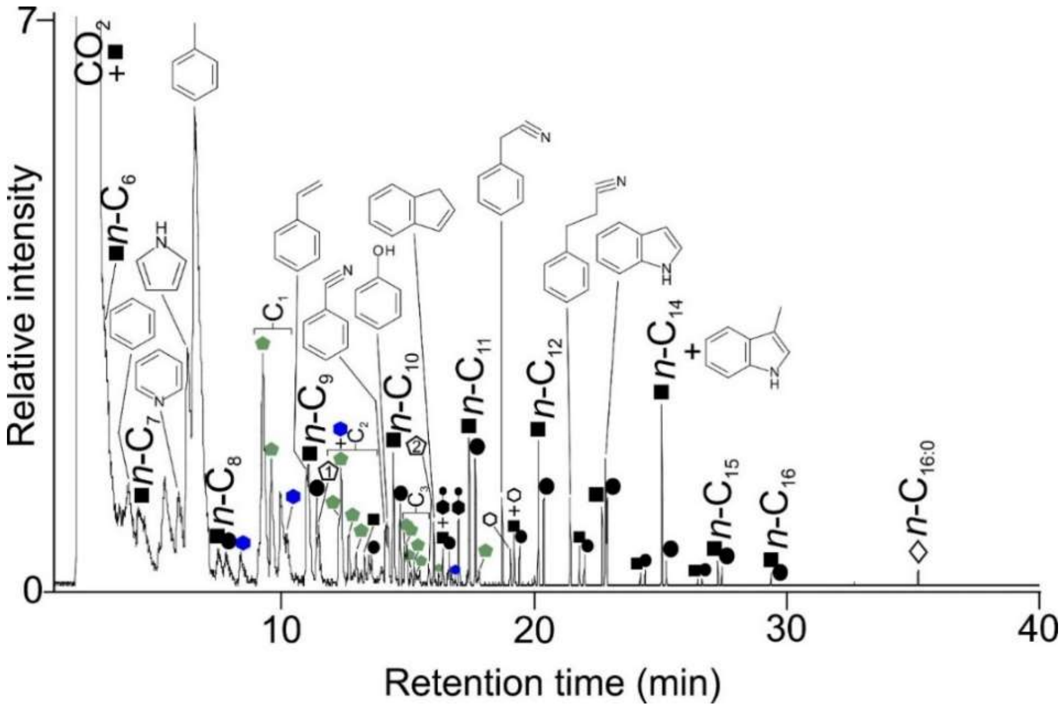

Supplementary Figure 3 Reconstructed total ion chromatogram of the pyrogram (pyrolysis profile) (610 °C for 10s) of sample DL86, after thermal desorption (310 °C for 10 s). Key to Fig. 3: Peak identities (x indicates carbon chain length): filled squares, Cx indicates alkenes; filled circles, Cx indicates alkanes; open diamonds, Cx:y indicates acyclic nitriles; blue filled hexagons indicates alkyl pyridines; green filled pentagons indicates alkyl pyrroles (with alkyl chain length above, Cx); filled hexagons with filled circles attached indicates alkyl phenols; open hexagons indicates alkyl indenes; open pentagons with numerals inside indicates carbohydrate pyrolysis markers, 1 is 2-methylfuran, 2 is 2-methyl-2-cyclopenten-1-one and 3 is 2,3-dimethyl-2-cyclopentene-1-one. Also shown are the structures of thirteen aromatic compounds identified: benzene, pyridine, pyrrole, toluene, styrene, benzonitrile, phenol, indene, benzyl nitrile, benzenepropanenitrile, indole, 3-methylindole (skatole) and 3-ethylindole. CO_2 indicates carbon dioxide.

5. Isbister DL143 1-2 Sk78 SC3 mandible IS1976 (calculus: 4.92 mg)

The thermal desorption total ion chromatogram (TIC) revealed carbon dioxide as the only detectable (trace) component, indicating the essential absence of free, thermally extractable organic components in this sample. It should be noted, however, that highly polar material could have been present, which would not have successfully eluted from the column, or

indeed volatilised sufficiently in the probe. It is not possible to determine the significance of the carbon dioxide, beyond it being indicative of oxidised organic material.

In contrast to the thermal desorption profile, the pyrolysis-GC-MS TIC (Fig. 4) revealed a number of organic compounds, albeit in relatively low abundance. These include a series of C_9 to C_{14} *n*-1-alkenes and *n*-alkanes, typical of unsaturated and saturated fat/oil-derived acyl lipids[5,6]. A bimodal distribution with maxima at C_{12} and C_{14} suggests the original acyl lipids may have been high in polyunsaturated fatty acids. The absence of these compounds in the TD profile provides molecular evidence for a polymeric/bound acyl lipid source. Although the series of ~C_{10} to ~C_{15} iso-methyl-1-alkenes (isoalkenes) and 2-methylalkanes (isoalkanes), indicative of a bacterial lipid input and identified in previous samples from Isbister (see above), were not observed, the detection of the *i*-C_{12} 1-alkene as a very minor component suggests their overall absence in this sample is due to the relatively small amount of organic material present.

There were a number of chemical markers identified in the pyrolysate TIC which can be indicative of proteins, i.e. toluene, pyrrole, 2-methylpyrrole, 3-methylpyrrole and 2-ethylpyrrole[6,11]. The complex suite of nitrogen-containing protein markers observed in previous samples (see above) were not detected, which given the notably similar pyrolysis profile of this sample when compared to those with appreciable amounts of organic material above, suggests this is again due to the relatively small amount of organic material present. This may also explain the absence of nitriles, phenols, pyridines, C_3 and C_4 alkyl pyrroles and carbohydrate markers in the pyrolysate of this sample. The presence of carbon dioxide is also consistent with a lipid and amino acid/protein component in this sample. It should also be noted that highly polar material could have been present, which would not have successfully eluted from the column, or indeed volatilised sufficiently in the probe. The low abundance of organic material in this sample does not allow any further inferences to be made (but see discussion).

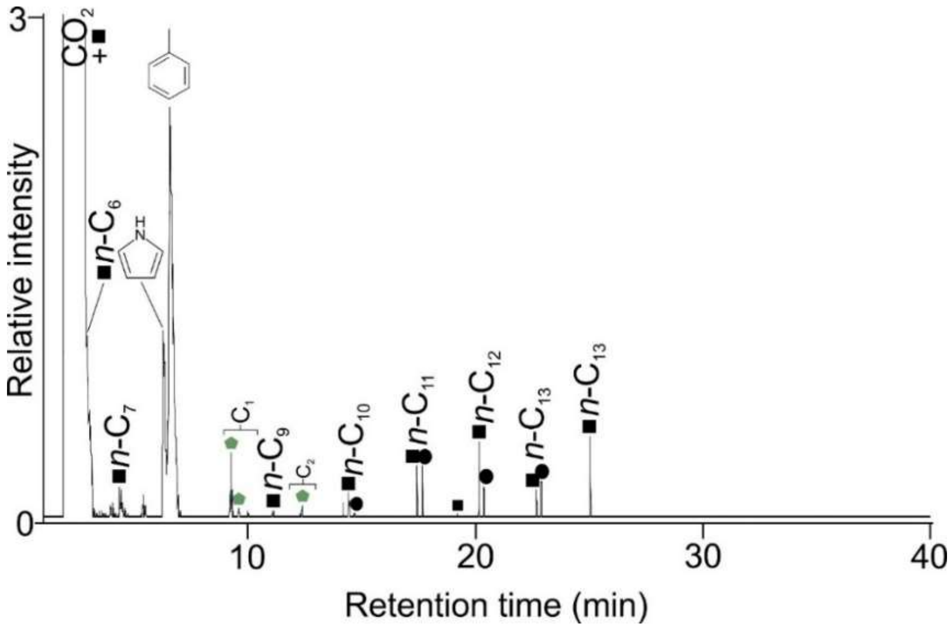

Supplementary Figure 4. Reconstructed total ion chromatogram of the pyrogram (pyrolysis profile) (610 °C for 10s) of sample DL143, after thermal desorption (310 °C for 10 s). Key to Fig. 4: Peak identities (x indicates carbon chain length): filled squares, Cx indicates alkenes; filled circles, Cx indicates alkanes; green filled pentagons indicate alkyl pyrroles (with alkyl chain length above, Cx). Also shown are the structures of two aromatic compounds identified: pyrrole and toluene. CO_2 indicates carbon dioxide.

6. Isbister DL168 Sk78 SC1 ST2 mandible IS1958 (calculus: 1.88 mg)

The thermal desorption total ion chromatogram (TIC) revealed carbon dioxide and acetonitrile as the only detectable (trace) components, indicating the virtual absence of free, thermally extractable organic components in this sample. It should be noted, however, that highly polar material could have been present, which would not have successfully eluted from the column, or indeed volatilised sufficiently in the probe. It is not possible to determine the significance of the carbon dioxide, beyond it being indicative of oxidised organic material. The origin of the acetonitrile is also difficult to determine, beyond it being indicative of a relatively labile nitrogen-containing polymeric/bound biomolecule (hence the generation of acetonitrile at only ~300°C), although it has been correlated with the pyrolysis of proteins high in phenylalanine and tyrosine, in particular[26].

In contrast to the thermal desorption profile, the pyrolysis-GC-MS TIC (Fig. 5) revealed a number of organic compounds, albeit in relatively low abundance. These include a series of

C_9 to C_{15} *n*-1-alkenes and *n*-alkanes, typical of unsaturated and saturated fat/oil-derived acyl lipids[5,6]. A bimodal distribution with maxima at C_{11}/C_{12} and C_{14} suggests the original acyl lipids may have been high in polyunsaturated fatty acids. The absence of these compounds in the TD profile provides molecular evidence for a polymeric/bound acyl lipid source. However, in addition to *n*-1-alkenes and *n*-alkanes, typical of fat/oil derived lipids, the pyrogram also displayed a series of C_{11} to C_{14} iso-methyl-1-alkenes (isoalkenes) and 2-methylalkanes (isoalkanes), albeit in minor abundance due to the relatively small amount of organic material present; these are again presumed to derive from the branched fatty acids which are significant components of bacterial triglycerides.

There were a number of chemical markers identified in the pyrolysate TIC which can be indicative of proteins, i.e. toluene, pyrrole, 2-methylpyrrole, 3-methylpyrrole, 2-ethylpyrrole, a dimethylpyridine and benzenepropanenitrile[6,11]. The complex suite of nitrogen-containing protein markers observed in previous samples (see above) were not detected, which given the notably similar pyrolysis profile of this sample when compared to those with appreciable amounts of organic material above, suggests this is yet again due to the relatively small amount of organic material present. Despite this, the $C_{16:0}$ nitrile was observed as a significant component, as was both phenol and 4-methylphenol, although the C_3 and C_4 alkyl pyrroles and carbohydrate markers were not detected in the pyrolysate. The presence of carbon dioxide is also consistent with a lipid and amino acid/protein component in this sample. It should also be noted that highly polar material could have been present, which would not have successfully eluted from the column, or indeed volatilised sufficiently in the probe. The low abundance of organic material in this sample does not allow any further inferences to be made in isolation, but see discussion below.

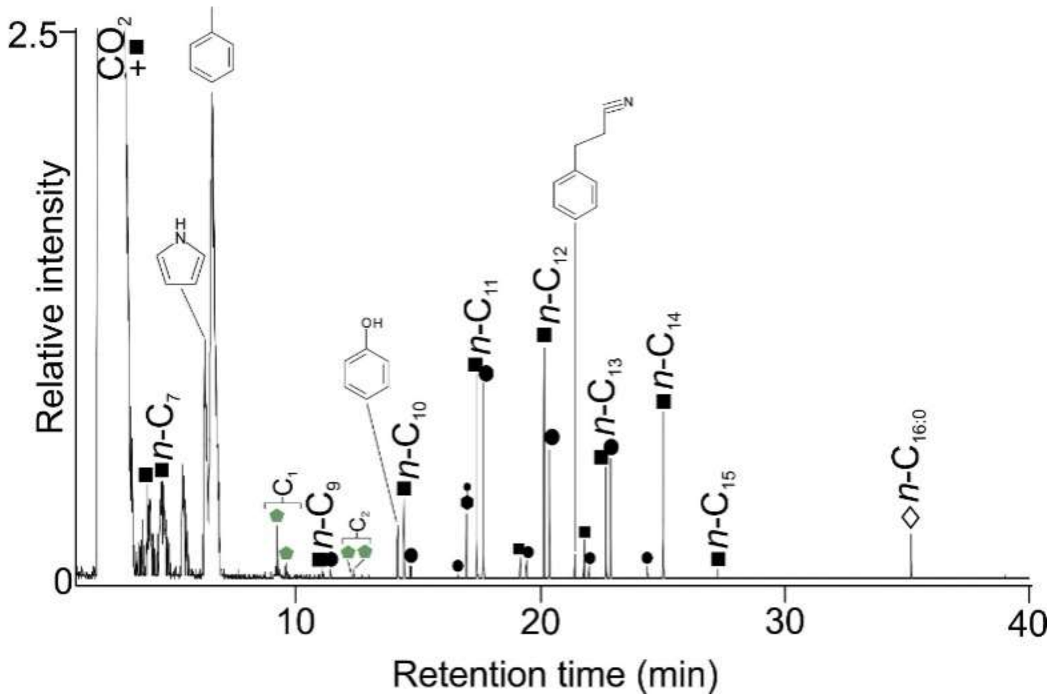

Supplementary Figure 5. Reconstructed total ion chromatogram of the pyrogram (pyrolysis profile) (610 °C for 10s) of sample DL168, after thermal desorption (310 °C for 10 s). Key to Fig. 5: Peak identities (x indicates carbon chain length): filled squares, Cx indicates alkenes; filled circles, Cx indicates alkanes; open diamond, Cx:y indicates acyclic nitrile; green filled pentagons indicates alkyl pyrroles (with alkyl chain length above, Cx); filled hexagon with filled circle attached indicates alkyl phenol. Also shown are the structures of four aromatic compounds identified: pyrrole, toluene, phenol and benzenepropanenitrile. CO_2 indicates carbon dioxide.

7. Isbister DL170 Sk78 SC1 ST2 mandible IS1958 (calculus: 3.74 mg)

The thermal desorption total ion chromatogram (TIC) revealed carbon dioxide as the only detectable (trace) component, indicating the essential absence of free, thermally extractable organic components in this sample. It should be noted, however, that highly polar material could have been present, which would not have successfully eluted from the column, or indeed volatilised sufficiently in the probe. It is not possible to determine the significance of the carbon dioxide, beyond it being indicative of oxidised organic material.

In contrast to the thermal desorption profile, the pyrolysis-GC-MS TIC (Fig. 6) revealed a number of organic compounds, albeit in low to moderate abundance. These include a series

of C_8 to C_{16} *n*-1-alkenes and *n*-alkanes, typical of unsaturated and saturated fat/oil-derived acyl lipids[5,6]. A bimodal distribution with maxima at C_{11}/C_{12} and C_{14} suggests the original acyl lipids may have been high in polyunsaturated fatty acids. The absence of these compounds in the TD profile provides molecular evidence for a polymeric/bound acyl lipid source. Although the series of ~C_{10} to ~C_{15} iso-methyl-1-alkenes (isoalkenes) and 2-methylalkanes (isoalkanes), indicative of a bacterial lipid input and identified in previous samples from Isbister (see above), were not observed, the detection of the *i*-C_{12} and *i*-C_{13} 1-alkenes as a very minor components suggests their overall absence in this sample is due to the relatively small amount of organic material present.

There were components identified in the pyrolysate TIC which can be indicative of 'black carbon', i.e. charcoal or soot[7-10]. However, lack of alkyl naphthalenes and biphenyls and the dominance of toluene, rather than the normally dominant aromatic hydrocarbon benzene, is notably atypical of chars[7-10]. The markers identified here can also be indicative of proteins[6,11] and combined with the relatively abundant related protein markers pyrrole, benzonitrile, benzyl nitrile (phenylacetonitrile), benzenepropanenitrile and indoles[6,11] identified this is likely to be the origin of these compounds in this sample, pointing to a substantial protein-derived component[6,11]. This is also corroborated by the presence of the $C_{16:0}$ aliphatic nitrile, suggesting a lipid (fat/oil) in this sample which has combined with protein-derived nitrogen to produce these moieties[5]. The proteinaceous origin for the aromatic markers is further corroborated by the relatively significant abundance of phenol and 4-methylphenol (*p*-cresol), which collectively are indicative of the amino acid tyrosine[12-14]. With an absence of guaiacol itself (all isomers) and other guaiacol moieties characteristic of lignin[15,16], the possibility that the phenols may originate from wood or woody plant material can be excluded. Alkyl phenols have also been observed in wood smoke and smoked foods[17,18], however the absence of methoxyphenols, diagnostic of lignin-derived smoke, means this possibility can also be discounted. Taken together, the presence of toluene, styrene and phenylacetonitrile (benzyl nitrile) can indicate the amino acid phenylalanine[12,13]. In addition, the significant constituents indole and 3-methylindole (skatole) are consistent with a tryptophan amino acid source [12,13,19,20]. Proteins rich in the amino acids tyrosine, phenylalanine and tryptophan include meat, fish and dairy products, although the presence of bacterial markers means the possibility that at least a partial origin could be microbial cannot be excluded.

However, notable in this context is the additional presence of appreciable amounts of the more unusual 2- and 3-methylpyrroles, 2-ethylpyrrole and 2,3-dimethyl-pyrrole, with minor

but detectable amounts of 2-ethyl-3(?)-methylpyrrole, 4-ethyl-2-methylpyrrole and 2,3,5-trimethylpyrrole. Collectively, these specific C_1, C_2, and C_3 (C_4 not detected due to the low abundance of organic material present) alkyl pyrroles suggest a tetrapyrrole/porphyrin-derived origin and are indicative of an algal source observed in fossils and expected in similarly diagenetically altered archaeological material[11,21,22]. Notable also in this context were pyridine and its methyl-, and dimethyl derivatives (these pyridines can result from the pyrolysis of polypeptides containing the amino acid alanine[23]). The presence here of the protein markers and specifically the C_1 to C_4 alkyl pyrroles, combined with the particular lipid profile, tentatively suggests the presence of a macroalgae, i.e. seaweed, in this sample[21,22,24] (see discussion).

The carbohydrate markers 2-methyl-2-cyclopenten-1-one and 2,3-dimethyl-2-cyclopenten-1-one were not detected in the pyrolysate, which given the notably similar pyrolysis profile of this sample when compared to those above, suggests this is due to the relatively small amount of organic material present. The presence of carbon dioxide is also consistent with a lipid and amino acid/protein component in this sample. It should also be noted that highly polar material could have been present, which would not have successfully eluted from the column, or indeed volatilised sufficiently in the probe. The low abundance of organic material in this sample does not allow any further inferences to be made (but see discussion).

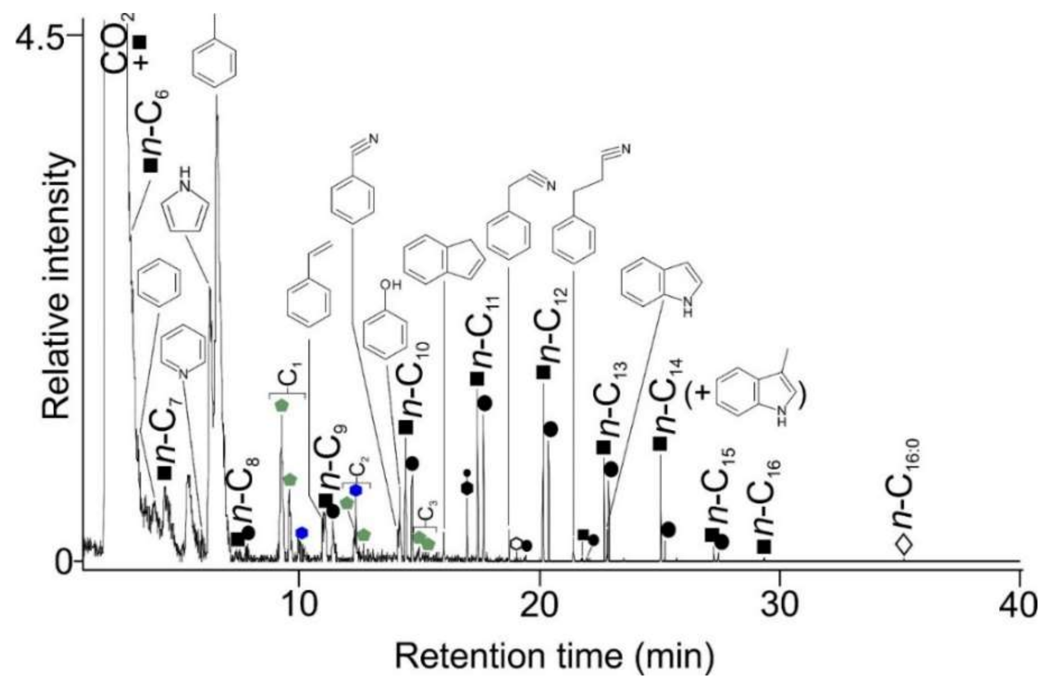

Supplementary Figure 6. Reconstructed total ion chromatogram of the pyrogram (pyrolysis profile) (610 °C for 10s) of sample DL170, after thermal desorption (310 °C for 10 s). Key to Fig. 6: Peak identities (x indicates carbon chain length): filled squares, Cx indicates alkenes; filled circles, Cx indicates alkanes; open diamond, Cx:y indicates acyclic nitrile; blue filled hexagons indicates alkyl pyridines; green filled pentagons indicates alkyl pyrroles (with alkyl chain length above, Cx); filled hexagon with filled circle attached indicates alkyl phenol; open hexagon indicates alkyl indene. Also shown are the structures of twelve aromatic compounds identified: benzene, pyridine, pyrrole, toluene, styrene, benzonitrile, phenol, indene, benzyl nitrile, benzenepropanenitrile, indole and 3-methylindole (skatole) (minor). CO_2 indicates carbon dioxide.

8. Isbister DL180 Sk78 BC2 (a+b) SC1 ST2 mandible IS1958 (calculus: 2.18 mg)

The thermal desorption total ion chromatogram (TIC) revealed carbon dioxide and acetonitrile as the only detectable (trace) components, indicating the virtual absence of free, thermally extractable organic components in this sample. It should be noted, however, that highly polar material could have been present, which would not have successfully eluted from the column, or indeed volatilised sufficiently in the probe. It is not possible to determine the significance of the carbon dioxide, beyond it being indicative of oxidised organic material. The origin of the acetonitrile is also difficult to determine, beyond it being indicative of a relatively labile nitrogen-containing polymeric/bound biomolecule (hence the generation of acetonitrile at only ~300°C), although it has been correlated with the pyrolysis of proteins high in phenylalanine and tyrosine, in particular[26].

In contrast to the thermal desorption profile, the pyrolysis-GC-MS TIC (Fig. 7) revealed a number of organic compounds, albeit in relatively low abundance. These include a series of C_8 to C_{16} *n*-1-alkenes and *n*-alkanes, typical of unsaturated and saturated fat/oil-derived acyl lipids[5,6]. A bimodal distribution with maxima at C_{11}/C_{12} and C_{13}/C_{14} suggests the original acyl lipids may have been high in polyunsaturated fatty acids. The absence of these compounds in the TD profile provides molecular evidence for a polymeric/bound acyl lipid source. However, in addition to *n*-1-alkenes and *n*-alkanes, typical of fat/oil derived lipids, the pyrogram also displayed a series of C_{10} to C_{14} iso-methyl-1-alkenes (isoalkenes) and 2-methylalkanes (isoalkanes), presumably deriving from the branched fatty acids which are significant components of bacterial triglycerides.

There were components identified in the pyrolysate TIC which can be indicative of 'black carbon', i.e. charcoal or soot[7-10]. However, lack of alkyl naphthalenes and biphenyls and the dominance of toluene, rather than the normally dominant aromatic hydrocarbon benzene, is notably atypical of chars[7-10]. The markers identified here can also be indicative of proteins[6,11] and combined with the relatively abundant related protein markers pyrrole, benzonitrile, benzyl nitrile (phenylacetonitrile), benzenepropanenitrile and indoles[6,11] identified this is likely to be the origin of these compounds in this sample, pointing to a substantial protein-derived component[6,11]. This is also corroborated by the presence of the $C_{16:0}$ and $C_{18:0}$ aliphatic nitriles, suggesting a lipid (fat/oil) in this sample which has combined with protein-derived nitrogen to produce these moieties[5]. The proteinaceous origin for the aromatic markers is further corroborated by the relatively significant abundance of phenol and 4-methylphenol (*p*-cresol), which collectively are indicative of the amino acid tyrosine[12-14]. With an absence of guaiacol itself (all isomers) and other guaiacol moieties characteristic of lignin[15,16], the possibility that the phenols may originate from wood or woody plant material can be excluded. Alkyl phenols have also been observed in wood smoke and smoked foods[17,18], however the absence of methoxyphenols, diagnostic of lignin-derived smoke, means this possibility can also be discounted. Taken together, the presence of toluene, styrene and phenylacetonitrile (benzyl nitrile) can indicate the amino acid phenylalanine[12,13]. In addition, the significant constituents indole and 3-methylindole (skatole) are consistent with a tryptophan amino acid source[12,13,19,20]. Proteins rich in the amino acids tyrosine, phenylalanine and tryptophan include meat, fish and dairy products, although the presence of bacterial markers means the possibility that at least a partial origin could be microbial cannot be excluded.

However, notable in this context is the additional presence of appreciable amounts of the more unusual 2- and 3-methylpyrroles and 2-ethylpyrrole, although C_3 and C_4 alkyl pyrroles were not detected, quite possibly due to the low abundance of organic material present. Consequently, this does not allow any further inferences to be made in isolation, but see discussion below. The carbohydrate markers 2-methyl-2-cyclopenten-1-one and 2,3-dimethyl-2-cyclopenten-1-one were detected in the pyrolysate, albeit only as trace components (for their likely significance here see discussion below). The presence of carbon dioxide is also consistent with a lipid and amino acid/protein component in this sample. It should also be noted that highly polar material could have been present, which would not have successfully eluted from the column, or indeed volatilised sufficiently in the probe. The

low abundance of organic material in this sample does not allow any further inferences to be made here (but see discussion).

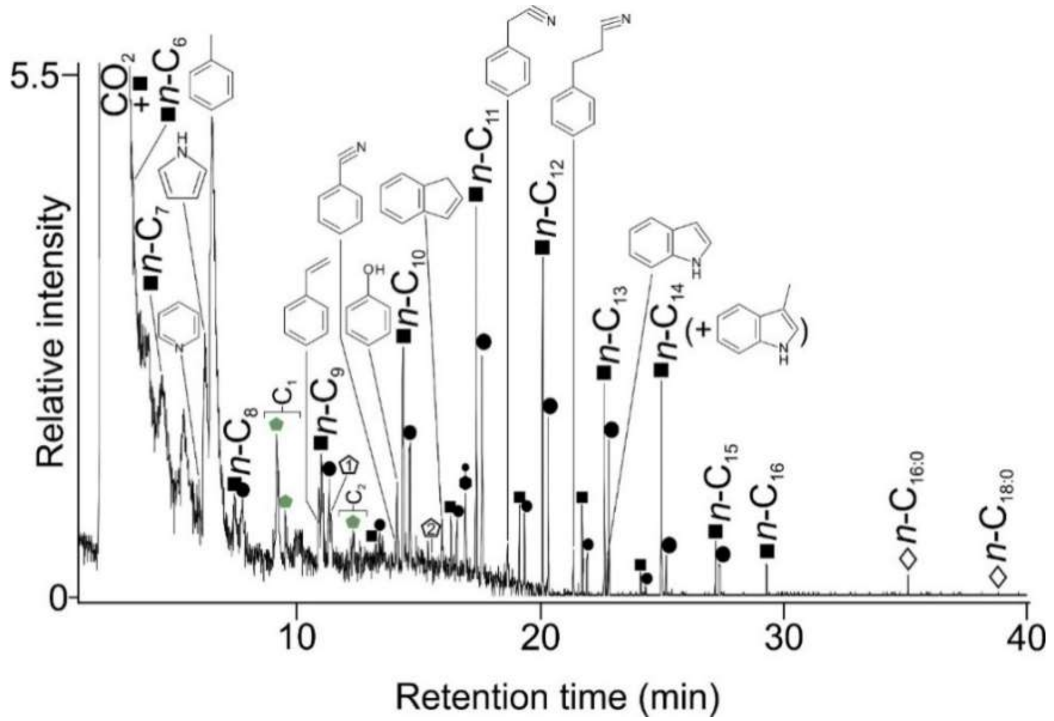

Supplementary Figure 7. Reconstructed total ion chromatogram of the pyrogram (pyrolysis profile) (610 °C for 10s) of sample DL180, after thermal desorption (310 °C for 10 s). Key to Fig. 7: Peak identities (x indicates carbon chain length): filled squares, Cx indicates alkenes; filled circles, Cx indicates alkanes; open diamonds, Cx:y indicates acyclic nitriles; green filled pentagons indicates alkyl pyrroles (with alkyl chain length above, Cx); filled hexagon with filled circle attached indicates alkyl phenol; open pentagons with numerals inside indicates carbohydrate pyrolysis markers, 1 is 2-methyl-2-cyclopenten-1-one and 2 is 2,3-dimethyl-2-cyclopentene-1-one (trace). Also shown are the structures of eleven aromatic compounds identified: pyridine, pyrrole, toluene, styrene, benzonitrile, phenol, indene, benzyl nitrile, benzenepropanenitrile, indole and 3-methylindole (skatole) (minor). CO_2 indicates carbon dioxide.

9. Isbister DL188 1-3 Sk78 BC7 jaw fragment IS1976 (calculus: 4.05 mg)

The thermal desorption total ion chromatogram (TIC) revealed carbon dioxide as the only detectable (trace) component, indicating the essential absence of free, thermally extractable organic components in this sample. It should be noted, however, that highly polar material

could have been present, which would not have successfully eluted from the column, or indeed volatilised sufficiently in the probe. It is not possible to determine the significance of the carbon dioxide, beyond it being indicative of oxidised organic material.

In contrast to the thermal desorption profile, the pyrolysis-GC-MS TIC (Fig. 8) revealed a number of organic compounds, albeit in very low abundance. These include a series of C_9 to C_{14} *n*-1-alkenes and *n*-alkanes, typical of unsaturated and saturated fat/oil-derived acyl lipids[5,6]. The absence of these compounds in the TD profile provides molecular evidence for a polymeric/bound acyl lipid source. Although the series of ~C_{10} to ~C_{15} iso-methyl-1-alkenes (isoalkenes) and 2-methylalkanes (isoalkanes), indicative of a bacterial lipid input and identified in previous samples from Isbister (see above), were not observed in this sample this is likely to be due to the very small amount of organic material present.

There were a small number of chemical markers identified in the pyrolysate TIC which can be indicative of proteins, i.e. toluene, styrene, pyrrole, and 2-methylpyrrole[6,11]. The complex suite of nitrogen-containing protein markers observed in previous samples (see above) were not detected, which again is likely to be due to the very small amount of organic material present. This may also explain the absence of nitriles, phenols, pyridines, C_2, C_3 and C_4 alkyl pyrroles, and carbohydrate markers in the pyrolysate of this sample. The presence of carbon dioxide is also consistent with a lipid and amino acid/protein component in this sample. It should also be noted that highly polar material could have been present, which would not have successfully eluted from the column, or indeed volatilised sufficiently in the probe. The low abundance of organic material in this sample does not allow any further inferences to be made (but see discussion).

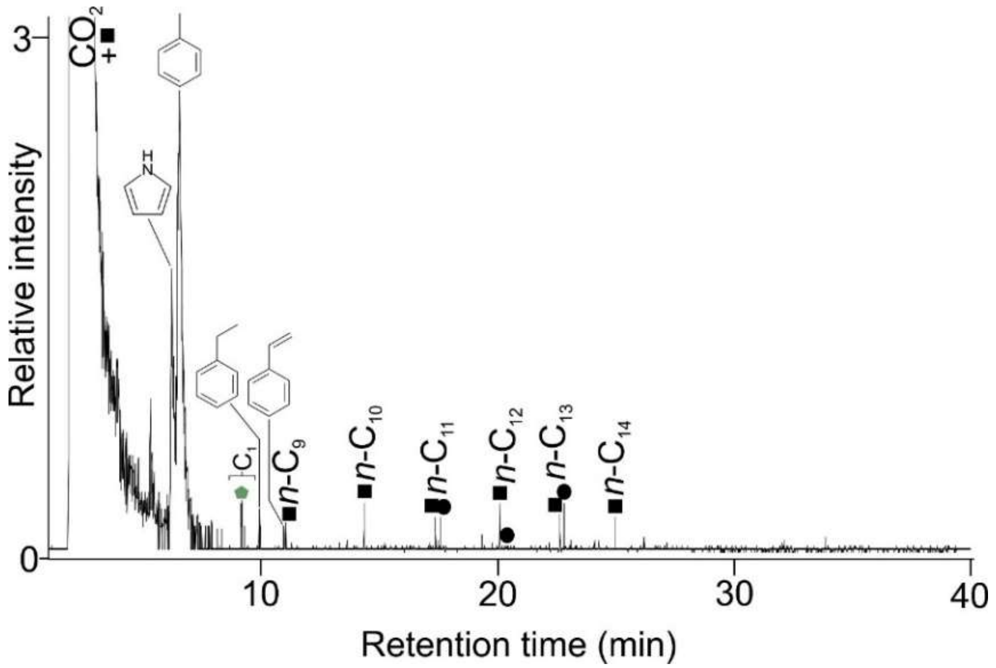

Supplementary Figure 8. Reconstructed total ion chromatogram of the pyrogram (pyrolysis profile) (610 °C for 10s) of sample DL188, after thermal desorption (310 °C for 10 s). Key to Fig. 8: Peak identities (x indicates carbon chain length): filled squares, Cx indicates alkenes; filled circles, Cx indicates alkanes; green filled pentagon indicates alkyl pyrrole (with alkyl chain length above, Cx); filled. Also shown are the structures of four aromatic compounds identified: pyrrole, toluene, ethyl benzene and styrene. CO_2 indicates carbon dioxide.

10. Isbister DL196 5 BC4 ST4 ISB incisor IS1976 (calculus: 2.58 mg)

The thermal desorption total ion chromatogram (TIC) revealed carbon dioxide and acetonitrile as the only detectable components, indicating the virtual absence of free, thermally extractable organic components in this sample. It should be noted, however, that highly polar material could have been present, which would not have successfully eluted from the column, or indeed volatilised sufficiently in the probe. It is not possible to determine the significance of the carbon dioxide, beyond it being indicative of oxidised organic material. The origin of the acetonitrile is also difficult to determine, beyond it being indicative of a relatively labile nitrogen-containing polymeric/bound biomolecule (hence the generation of acetonitrile at only ~300°C), although it has been correlated with the pyrolysis of proteins high in phenylalanine and tyrosine, in particular[26].

In contrast to the thermal desorption profile, the pyrolysis-GC-MS TIC (Fig. 9) revealed a suite of organic compounds, albeit in relatively low abundance. These include a series of C_9 to C_{15} *n*-1-alkenes and *n*-alkanes, typical of unsaturated and saturated fat/oil-derived acyl lipids[5,6]. A bimodal distribution with maxima at C_{11}/C_{12} and C_{14} suggests the original acyl lipids may have been high in polyunsaturated fatty acids. The absence of these compounds in the TD profile provides molecular evidence for a polymeric/bound acyl lipid source. However, in addition to *n*-1-alkenes and *n*-alkanes, typical of fat/oil derived lipids, the pyrogram also displayed a series of C_{11} to C_{13} iso-methyl-1-alkenes (isoalkenes) and 2-methylalkanes (isoalkanes), albeit in relatively moderate abundance due to the relatively small amount of organic material present; these are again presumed to derive from the branched fatty acids which are significant components of bacterial triglycerides.

There were a number of chemical markers identified in the pyrolysate TIC which can be indicative of proteins, i.e. toluene, styrene, pyrrole, 2-methylpyrrole, 3-methylpyrrole, 2-ethylpyrrole and a dimethylpyridine[6,11]. The complex suite of nitrogen-containing protein markers observed in previous samples (see above) were not detected, which given the notably similar pyrolysis profile of this sample when compared to those with appreciable amounts of organic material above, suggests this is again due to the relatively small amount of organic material present. Despite this, the proteinaceous origin for the non-nitrogenous aromatic markers is further corroborated by the relatively significant abundance of both phenol and 4-methylphenol (*p*-cresol), which collectively are indicative of the amino acid tyrosine[12-14]. However, alkyl nitriles and the C_3 and C_4 alkyl pyrroles were not detected in the pyrolysate, possibly because of the low abundance of organic material present. Interesting, despite the small amount of macromolecular observed, the carbohydrate markers 2-methyl-2-cyclopenten-1-one and 2,3-dimethyl-2-cyclopenten-1-one were detected in the pyrolysate, albeit as relatively minor components (for their likely significance here see discussion below). The presence of carbon dioxide is also consistent with a lipid, amino acid/protein and carbohydrate component in this sample. It should also be noted that highly polar material could have been present, which would not have successfully eluted from the column, or indeed volatilised sufficiently in the probe. The low abundance of organic material in this sample does not allow any further inferences to be made in isolation, but see discussion below.

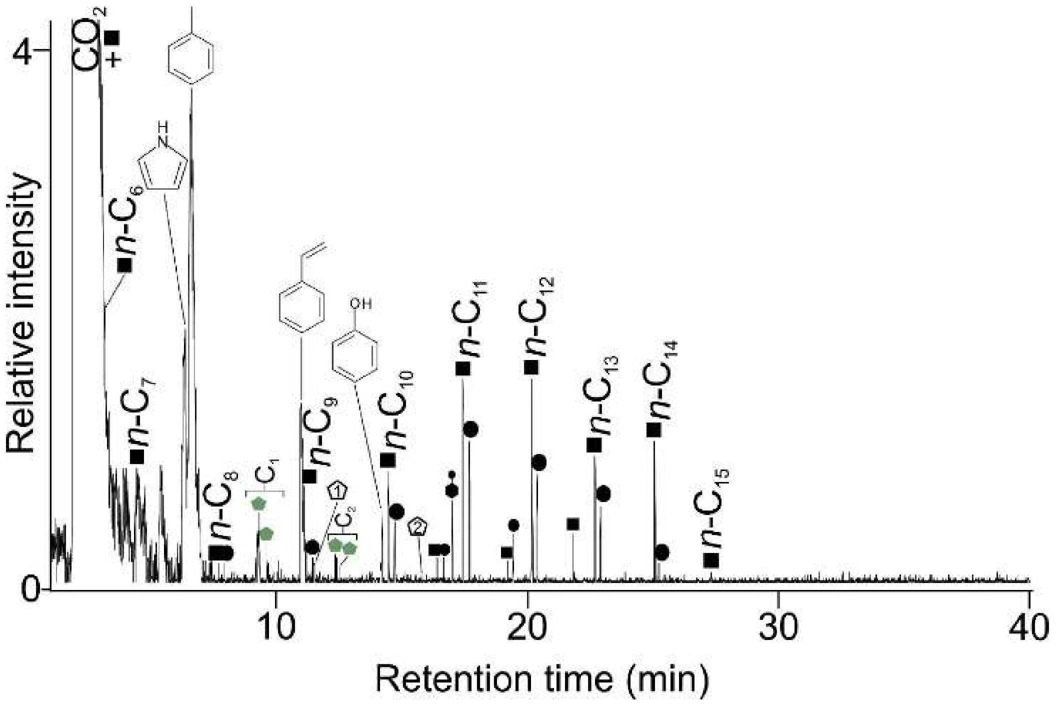

Supplementary Figure 9. Reconstructed total ion chromatogram of the pyrogram (pyrolysis profile) (610 °C for 10s) of sample DL196, after thermal desorption (310 °C for 10 s). Key to Fig. 9: Peak identities (x indicates carbon chain length): filled squares, Cx indicates alkenes; filled circles, Cx indicates alkanes; green filled pentagons indicates alkyl pyrroles (with alkyl chain length above, Cx); filled hexagon with filled circle attached indicates alkyl phenol; open pentagons with numerals inside indicates carbohydrate pyrolysis markers, 1 is 2-methyl-2-cyclopenten-1-one and 2 is 2,3-dimethyl-2-cyclopentene-1-one (trace). Also shown are the structures of four aromatic compounds identified: pyrrole, toluene, styrene and phenol. CO_2 indicates carbon dioxide.

11. Isbister DL715 24 Sk77 BC4 mandible IS1976 (calculus: 12.15 mg)

The thermal desorption total ion chromatogram (TIC) revealed carbon dioxide and acetonitrile as the only detectable components, indicating the virtual absence of free, thermally extractable organic components in this sample. It should be noted, however, that highly polar material could have been present, which would not have successfully eluted from the column, or indeed volatilised sufficiently in the probe. It is not possible to determine the significance of the carbon dioxide, beyond it being indicative of oxidised organic material. The origin of the acetonitrile is also difficult to determine, beyond it being

indicative of a relatively labile nitrogen-containing polymeric/bound biomolecule (hence the generation of acetonitrile at only ~300°C), although it has been correlated with the pyrolysis of proteins high in phenylalanine and tyrosine, in particular[26].

In contrast to the thermal desorption profile, the pyrolysis-GC-MS TIC (Fig. 10) is dominated by a complex suite of organic compounds. These include a series of C_8 to C_{17} *n*-1-alkenes and *n*-alkanes, typical of unsaturated and saturated fat/oil-derived acyl lipids[5,6]. A bimodal distribution with maxima at C_{11}/C_{12} and C_{14} suggests the original acyl lipids may have been high in polyunsaturated fatty acids. The absence of these compounds in the TD profile provides molecular evidence for a polymeric/bound acyl lipid source. However, in addition to *n*-1-alkenes and *n*-alkanes, typical of fat/oil derived lipids, the pyrogram also displayed a series of C_{10} to C_{16} iso-methyl-1-alkenes (isoalkenes) and 2-methylalkanes (isoalkanes), presumably deriving from the branched fatty acids which are significant components of bacterial triglycerides.

There were components identified in the pyrolysate TIC which can be indicative of ‘black carbon’, i.e. charcoal or soot[7-10]. However, lack of alkyl naphthalenes and biphenyls and the dominance of toluene, rather than the normally dominant aromatic hydrocarbon benzene, is notably atypical of chars[7-10]. The markers identified here can also be indicative of proteins[6,11] and combined with the abundant related protein markers pyrrole, benzonitrile, benzyl nitrile (phenylacetonitrile), benzenepropanenitrile and indoles[6,11] identified this is likely to be the main origin of these compounds in this sample, pointing to a substantial protein-derived component[6,11]. This is also corroborated by the presence of aliphatic nitriles (carbon numbers $C_{14:0}$, *i*-$C_{15:0}$, $C_{16:0}$ (max) and $C_{18:0}$) suggesting a lipid (fat/oil) in this sample which has combined with protein-derived nitrogen to produce these moieties[5]. The proteinaceous origin for the aromatic markers is further corroborated by the significant abundance of phenol, 3-methylphenol (*m*-cresol), 4-methylphenol (*p*-cresol) (with the 4-methyl isomer being predominant), and lesser amounts of an unidentified C_2 phenol, all of which, collectively, are indicative of the amino acid tyrosine[12-14]. With an absence of guaiacol itself (all isomers) and other guaiacol moieties characteristic of lignin[15,16], the possibility that the phenols may originate from wood or woody plant material can be excluded. Alkyl phenols have also been observed in wood smoke and smoked foods[17,18], however the absence of methoxyphenols, diagnostic of lignin-derived smoke, means this possibility can also be discounted. Taken together, the presence of toluene, styrene and phenylacetonitrile (benzyl nitrile) can indicate the amino acid phenylalanine[12,13]. In addition, the major constituents indole and 3-

methylindole (skatole) were also identified, which are consistent with a tryptophan amino acid source[12,13,19,20]. Proteins rich in the amino acids tyrosine, phenylalanine and tryptophan include meat, fish and dairy products, although the presence of bacterial markers means the possibility that at least a partial origin could be microbial cannot be excluded.

However, highly notable in this context is the additional presence of appreciable amounts of the more unusual 2- and 3-methylpyrroles, 2- and 3-ethylpyrroles, 2,4-, 2,3- and 3,4-dimethyl-pyrroles, 2-ethyl-4(?)-methylpyrrole, 2-ethyl-3(?)-methylpyrrole, 4-ethyl-2-methylpyrrole, 3-ethyl-4-methylpyrrole (trace), 2,3,5-trimethylpyrrole, 2,3,4-trimethylpyrrole, a propylmethylpyrrole, 2,5(?)-diethylpyrrole, 2,3(?)-diethylpyrrole, an ethyldimethylpyrrole, 4-ethyl-2,3-dimethylpyrrole, 3-ethyl-2,4-dimethylpyrrole, 2,3(?)-diethyl-5?-methylpyrrole, 2,3-dimethyl-4-n-propylpyrrole and 3-methyl-4-neopentylpyrrole (trace). Collectively, these specific C_1, C_2, C_3, C_4, C_5 and C_6 alkyl pyrroles suggest a significant tetrapyrrole/porphyrin-derived origin and are indicative of an algal source observed in fossils and expected in similarly diagenetically altered archaeological material[11,21,22]. Notable also in this context were the presence of the moderately abundant nitrogen containing aromatic compounds 1-H-pyrazole-4-carbonitrile, ethylcyanobenzene, 2-pyridinecarbonitrile and tolyl isocyanide, along with pyridine and its methyl-, dimethyl- and trimethyl derivatives (these pyridines can result from the pyrolysis of polypeptides containing the amino acid alanine[23]). The presence here of the abundant protein markers and specifically the C_1 to C_6 alkyl pyrroles, combined with the particular lipid profile and specific carbohydrate markers (see below) identified, suggests the presence of a macroalgae, i.e. seaweed, in this sample[21,22,24] (see discussion).

The thermally-derived carbohydrate markers 2-methyl-2-cyclopenten-1-one and 2,3-dimethyl-2-cyclopenten-1one were identified as significant components, along with an unidentified carbohydrate marker. That they were absent in the TD profile points to their originating from the original polymeric carbohydrate source (e.g. starch)[25]. Although levoglucosan (1,6-anhydro-β-D-glucopyranose) is usually the major component in the thermally-derived products of carbohydrates analysed by TD/Py-GC-MS, its absence from the TD profile here is consistent with the removal of hydroxyl groups in the original carbohydrate polymer, so preventing its formation in the probe. The additional absence of the normally significant carbohydrate markers 2-hydroxy-3-methyl-2-cyclopenten-1-one and 3-hydroxy-2-methyl-2-cyclopenten-1-one observed in pure standard material (glycogen, starch and cellulose) would also support this interpretation. This shows a strong matrix effect likely

to be due to the inorganic component of the calculus and the microflora in the mouth. This dehydration/dehydroxylation has also been observed in dog calculus and 19th century human calculus[6], where other markers, e.g. a prevalence of nitriles, with amides very minor, deriving from dehydrating conditions in the mouth of dogs and humans (though not sheep[6]) supporting this view[6]. The presence of carbon dioxide is also consistent with a lipid, amino acid/protein and carbohydrate component in this sample. Notably, these findings did not reveal key biomarkers typically observed in soils[20,23]. It should also be noted that highly polar material could have been present, which would not have successfully eluted from the column, or indeed volatilised sufficiently in the probe.

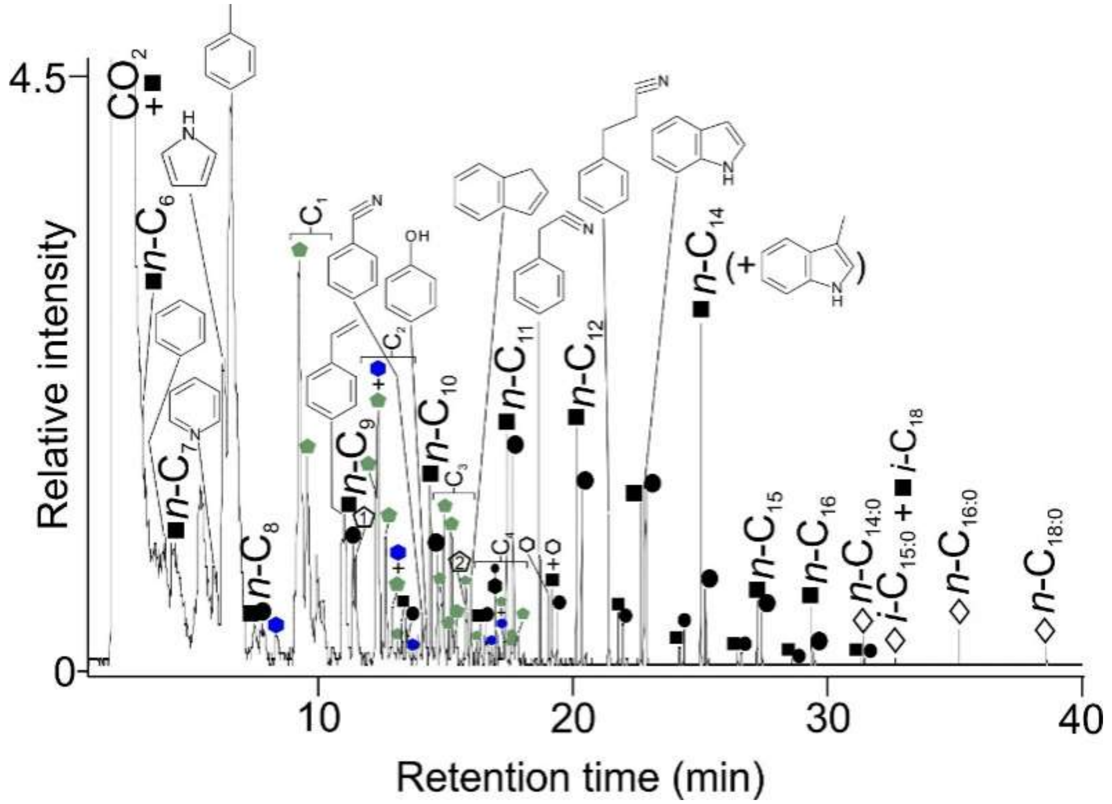

Supplementary Figure 10. Reconstructed total ion chromatogram of the pyrogram (pyrolysis profile) (610 °C for 10s) of sample DL715, after thermal desorption (310 °C for 10 s). Key to Fig. 10: Peak identities (x indicates carbon chain length): filled squares, Cx indicates alkenes; filled circles, Cx indicates alkanes; open diamonds, Cx:y indicates acyclic nitriles; blue filled hexagons indicates alkyl pyridines; green filled pentagons indicates alkyl pyrroles (with alkyl chain length above, Cx); filled hexagons with filled circles attached indicates alkyl phenols; open hexagons indicates alkyl indenes; open pentagons with

numerals inside indicates carbohydrate pyrolysis markers, 1 is 2-methyl-2-cyclopenten-1-one and 2 is 2,3-dimethyl-2-cyclopentene-1-one. Also shown are the structures of twelve aromatic compounds identified: benzene, pyridine, pyrrole, toluene, styrene, benzonitrile, phenol, indene, benzyl nitrile, benzenepropanenitrile, indole and 3-methylindole (skatole) (minor). CO_2 indicates carbon dioxide.

12. Isbister DL5083 x3 Sk77 BC2c IS128 mandible IS1976 (calculus: 10.39 mg)

The thermal desorption total ion chromatogram (TIC) revealed carbon dioxide and acetonitrile as the only detectable components, indicating the virtual absence of free, thermally extractable organic components in this sample. It should be noted, however, that highly polar material could have been present, which would not have successfully eluted from the column, or indeed volatilised sufficiently in the probe. It is not possible to determine the significance of the carbon dioxide, beyond it being indicative of oxidised organic material. The origin of the acetonitrile is also difficult to determine, beyond it being indicative of a relatively labile nitrogen-containing polymeric/bound biomolecule (hence the generation of acetonitrile at only ~300°C), although it has been correlated with the pyrolysis of proteins high in phenylalanine and tyrosine, in particular[26].

In contrast to the thermal desorption profile, the pyrolysis-GC-MS TIC (Fig. 11) is dominated by a relatively complex suite of organic compounds. These include a series of C_8 to C_{16} *n*-1-alkenes and *n*-alkanes, typical of unsaturated and saturated fat/oil-derived acyl lipids[5,6]. A bimodal distribution with maxima at C_{11}/C_{12} and C_{14} suggests the original acyl lipids may have been high in polyunsaturated fatty acids. The absence of these compounds in the TD profile provides molecular evidence for a polymeric/bound acyl lipid source. However, in addition to *n*-1-alkenes and *n*-alkanes, typical of fat/oil derived lipids, the pyrogram also displayed a series of C_{11} to C_{14} iso-methyl-1-alkenes (isoalkenes) and 2-methylalkanes (isoalkanes), presumably deriving from the branched fatty acids which are significant components of bacterial triglycerides.

There were components identified in the pyrolysate TIC which can be indicative of 'black carbon', i.e. charcoal or soot[7-10]. However, lack of alkyl naphthalenes and biphenyls and the dominance of toluene, rather than the normally dominant aromatic hydrocarbon benzene, is notably atypical of chars[7-10]. The markers identified here can also be indicative of proteins[6,11] and combined with the abundant related protein markers pyrrole, benzonitrile, benzyl nitrile

(phenylacetonitrile), benzenepropanenitrile and indoles[6,11] identified this is likely to be the main origin of these compounds in this sample pointing to a substantial protein-derived component[6,11]. This is also corroborated by the presence of the $C_{16:0}$ aliphatic nitrile, suggesting a lipid (fat/oil) in this sample which has combined with protein-derived nitrogen to produce these moieties[5]. The proteinaceous origin for the aromatic markers is further corroborated by the significant abundance of phenol, 2-methylphenol (*o*-cresol) and 4-methylphenol (*p*-cresol) (with the 4-methyl isomer being predominant), which collectively are indicative of the amino acid tyrosine[12-14]. With an absence of guaiacol itself (all isomers) and other guaiacol moieties characteristic of lignin[15,16], the possibility that the phenols may originate from wood or woody plant material can be excluded. Alkyl phenols have also been observed in wood smoke and smoked foods[17,18], however the absence of methoxyphenols, diagnostic of lignin-derived smoke, means this possibility can also be discounted. Taken together, the presence of toluene, styrene and phenylacetonitrile (benzyl nitrile) can indicate the amino acid phenylalanine[12,13]. In addition, the major constituents indole and 3-methylindole (skatole) are consistent with a tryptophan amino acid source[12,13,19,20]. Proteins rich in the amino acids tyrosine, phenylalanine and tryptophan include meat, fish and dairy products, although the presence of bacterial markers means the possibility that at least a partial origin could be microbial cannot be excluded.

However, highly notable in this context is the additional presence of appreciable amounts of the more unusual 2- and 3-methylpyrroles, 2- and 3-ethylpyrroles, 2,3-dimethyl-pyrrole, 2-ethyl-4(?)-methylpyrrole, 2-ethyl-3(?)-methylpyrrole, 4-ethyl-2-methylpyrrole, 3-ethyl-4-methylpyrrole, 2,3,5-trimethylpyrrole and a propylmethylpyrrole. Collectively, these specific C_1, C_2, C_3 and C_4 alkyl pyrroles suggest a tetrapyrrole/porphyrin-derived origin and are indicative of an algal source observed in fossils and expected in similarly diagenetically altered archaeological material[11,21,22]. Notable also in this context were the presence of the relatively moderately abundant nitrogen containing aromatic compounds 1-H-pyrazole-4-carbonitrile, 2-pyridinecarbonitrile and tolyl isocyanide (x2), along with pyridine and its methyl-, dimethyl- and trimethyl derivatives (these pyridines can result from the pyrolysis of polypeptides containing the amino acid alanine[23]). The presence here of the abundant protein markers and specifically the C_1 to C_4 alkyl pyrroles, combined with the particular lipid profile and specific carbohydrate markers (see below) identified, suggests the presence of a macroalgae, i.e. seaweed, in this sample[21,22,24] (see discussion).

The thermally-derived carbohydrate markers 2-methyl-2-cyclopenten-1-one and 2,3-dimethyl-2-cyclopenten-1one were identified as significant components, along with an unidentified carbohydrate marker. That they were absent in the TD profile points to their originating from the original polymeric carbohydrate source (e.g. starch)[25]. Although levoglucosan (1,6-anhydro-β-D-glucopyranose) is usually the major component in the thermally-derived products of carbohydrates analysed by TD/Py-GC-MS, its absence from the TD profile here is consistent with the removal of hydroxyl groups in the original carbohydrate polymer, so preventing its formation in the probe. The additional absence of the normally significant carbohydrate markers 2-hydroxy-3-methyl-2-cyclopenten-1-one and 3-hydroxy-2-methyl-2-cyclopenten-1-one observed in pure standard material (glycogen, starch and cellulose) would also support this interpretation. This shows a strong matrix effect likely to be due to the inorganic component of the calculus and the microflora in the mouth. This dehydration/dehydroxylation has also been observed in dog calculus and 19th century human calculus[6], where other markers, e.g. a prevalence of nitriles, with amides very minor, deriving from dehydrating conditions in the mouth of dogs and humans (though not sheep[6]) supporting this view[6]. The presence of carbon dioxide is also consistent with a lipid, amino acid/protein and carbohydrate component in this sample. Notably, these findings did not reveal key biomarkers typically observed in soils[20,23]. It should also be noted that highly polar material could have been present, which would not have successfully eluted from the column, or indeed volatilised sufficiently in the probe.

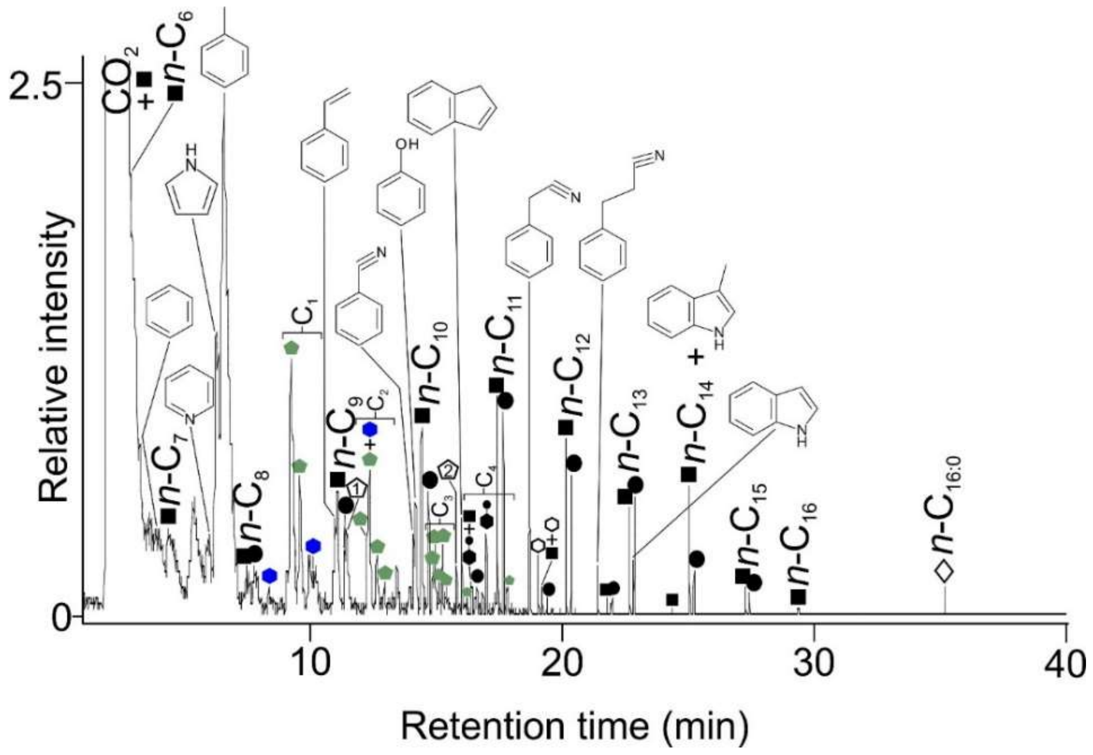

Supplementary Figure 11. Reconstructed total ion chromatogram of the pyrogram (pyrolysis profile) (610 °C for 10s) of sample DL5083, after thermal desorption (310 °C for 10 s). Key to Fig. 11: Peak identities (x indicates carbon chain length): filled squares, Cx indicates alkenes; filled circles, Cx indicates alkanes; open diamond, Cx:y indicates acyclic nitrile; blue filled hexagons indicates alkyl pyridines; green filled pentagons indicates alkyl pyrroles (with alkyl chain length above, Cx); filled hexagons with filled circles attached indicates alkyl phenols; open hexagons indicates alkyl indenes; open pentagons with numerals inside indicates carbohydrate pyrolysis markers, 1 is 2-methyl-2-cyclopenten-1-one and 2 is 2,3-dimethyl-2-cyclopentene-1-one. Also shown are the structures of twelve aromatic compounds identified: benzene, pyridine, pyrrole, toluene, styrene, benzonitrile, phenol, indene, benzyl nitrile, benzenepropanenitrile, indole and 3-methylindole (skatole). CO_2 indicates carbon dioxide.

13. Isbister DL5117 x3 Sk77 BC6(3) ST5 mandible 5 IS1976 (calculus: 5.69 mg)

The thermal desorption total ion chromatogram (TIC) revealed carbon dioxide as the only detectable (trace) component, indicating the essential absence of free, thermally extractable organic components in this sample. It should be noted, however, that highly polar material could have been present, which would not have successfully eluted from the column, or

indeed volatilised sufficiently in the probe. It is not possible to determine the significance of the carbon dioxide, beyond it being indicative of oxidised organic material.

The pyrolysate TIC revealed carbon dioxide, 1-butene, 1-heptene, a heptadiene, pyrrole, toluene and 2-methylpyrrole as the only detectable (trace) components, indicating the essential absence of organic material in this sample. It should be noted, however, that highly polar material could have been present, which would not have successfully eluted from the column, or indeed volatilised sufficiently in the probe. It is not possible to determine the significance of the carbon dioxide, beyond it being indicative of oxidised organic material. The short-chain alkenes, toluene and pyrroles are in too low an abundance to provide any meaningful interpretation of their presence, beyond being indicative of an organic origin.

14. Isbister DL5119 x4 Sk77 BC6(3) ST5 mandible 2 IS1976 (calculus: 6.03 mg)

The thermal desorption total ion chromatogram (TIC) revealed carbon dioxide and acetonitrile as the only detectable components, indicating the virtual absence of free, thermally extractable organic components in this sample. It should be noted, however, that highly polar material could have been present, which would not have successfully eluted from the column, or indeed volatilised sufficiently in the probe. It is not possible to determine the significance of the carbon dioxide, beyond it being indicative of oxidised organic material. The origin of the acetonitrile is also difficult to determine, beyond it being indicative of a relatively labile nitrogen-containing polymeric/bound biomolecule (hence the generation of acetonitrile at only ~300°C), although it has been correlated with the pyrolysis of proteins high in phenylalanine and tyrosine, in particular[26].

In contrast to the thermal desorption profile, the pyrolysis-GC-MS TIC (Fig. 12) is dominated by a relatively complex suite of organic compounds. These include a series of C_8 to C_{16} *n*-1-alkenes and *n*-alkanes, typical of unsaturated and saturated fat/oil-derived acyl lipids[5,6]. The absence of these compounds in the TD profile provides molecular evidence for a polymeric/bound acyl lipid source. However, in addition to *n*-1-alkenes and *n*-alkanes, typical of fat/oil derived lipids, the pyrogram also displayed a series of C_{11} to C_{14} iso-methyl-1-alkenes (isoalkenes) and 2-methylalkanes (isoalkanes), presumably deriving from the branched fatty acids which are significant components of bacterial triglycerides.

There were components identified in the pyrolysate TIC which can be indicative of 'black carbon', i.e. charcoal or soot[7-10]. However, lack of alkyl naphthalenes and biphenyls and the

dominance of toluene, rather than the normally dominant aromatic hydrocarbon benzene, is notably atypical of chars[7-10]. The markers identified here can also be indicative of proteins[6,11] and combined with the abundant related protein markers pyrrole, benzonitrile, benzyl nitrile (phenylacetonitrile), benzenepropanenitrile and indoles[6,11] identified this is likely to be the main origin of these compounds in this sample pointing to a substantial protein-derived component[6,11]. This is also corroborated by the presence of the $C_{16:0}$ aliphatic nitrile, suggesting a lipid (fat/oil) in this sample which has combined with protein-derived nitrogen to produce these moieties[5]. The proteinaceous origin for the aromatic markers is further corroborated by the moderate abundance of phenol and 4-methylphenol (*p*-cresol), which collectively are indicative of the amino acid tyrosine[12-14]. With an absence of guaiacol itself (all isomers) and other guaiacol moieties characteristic of lignin[15,16], the possibility that the phenols may originate from wood or woody plant material can be excluded. Alkyl phenols have also been observed in wood smoke and smoked foods[17,18], however the absence of methoxyphenols, diagnostic of lignin-derived smoke, means this possibility can also be discounted. Taken together, the presence of toluene, styrene and phenylacetonitrile (benzyl nitrile) can indicate the amino acid phenylalanine[12,13]. In addition, the major constituents indole and 3-methylindole (skatole) are consistent with a tryptophan amino acid source[12,13,19,20]. Proteins rich in the amino acids tyrosine, phenylalanine and tryptophan include meat, fish and dairy products, although the presence of bacterial markers means the possibility that at least a partial origin could be microbial cannot be excluded.

However, highly notable in this context is the additional presence of appreciable amounts of the more unusual 2- and 3-methylpyrroles, 2- and 3-ethylpyrroles, 2,4-, 2,3- and 3,4-dimethyl-pyrroles, 2-ethyl-4(?)-methylpyrrole, 2-ethyl-3(?)-methylpyrrole, 4-ethyl-2-methylpyrrole, 3-ethyl-4-methylpyrrole, 2,3,5-trimethylpyrrole, 2,3,4-trimethylpyrrole, two propylmethylpyrroles, 2,5(?)-diethylpyrrole, 2,3(?)-diethylpyrrole, 4-ethyl-2,3-dimethylpyrrole, 2,3(?)-diethyl-5?-methylpyrrole and 3-ethyl-2,4,5-trimethylpyrrole. Collectively, these specific C_1, C_2, C_3, C_4 and C_5 alkyl pyrroles suggest a tetrapyrrole/porphyrin-derived origin and are indicative of an algal source observed in fossils and expected in similarly diagenetically altered archaeological material[11,21,22]. Notable also in this context were the presence of the relatively moderately abundant nitrogen containing aromatic compounds 1-H-pyrazole-4-carbonitrile, 2-pyridinecarbonitrile and tolyl isocyanide (x2), 1-H-pyrrole-2-carbonitrile(?), along with pyridine and its methyl-, dimethyl- and trimethyl derivatives (these pyridines can result from the pyrolysis of polypeptides containing

the amino acid alanine[23]). The presence here of the abundant protein markers and specifically the C_1 to C_4 alkyl pyrroles, combined with the particular lipid profile and specific carbohydrate markers (see below) identified, suggests the presence of a macroalgae, i.e. seaweed, in this sample[21,22,24] (see discussion).

The thermally-derived carbohydrate markers 2-methyl-2-cyclopenten-1-one and 2,3-dimethyl-2-cyclopenten-1one were identified as moderately significant components, along with an unidentified carbohydrate marker. That they were absent in the TD profile points to their originating from the original polymeric carbohydrate source (e.g. starch)[25]. Although levoglucosan (1,6-anhydro-β-D-glucopyranose) is usually the major component in the thermally-derived products of carbohydrates analysed by TD/Py-GC-MS, its absence from the TD profile here is consistent with the removal of hydroxyl groups in the original carbohydrate polymer, so preventing its formation in the probe. The additional absence of the normally significant carbohydrate markers 2-hydroxy-3-methyl-2-cyclopenten-1-one and 3-hydroxy-2-methyl-2-cyclopenten-1-one observed in pure standard material (glycogen, starch and cellulose) would also support this interpretation. This shows a strong matrix effect likely to be due to the inorganic component of the calculus and the microflora in the mouth. This dehydration/dehydroxylation has also been observed in dog calculus and 19th century human calculus[6], where other markers, e.g. a prevalence of nitriles, with amides very minor, deriving from dehydrating conditions in the mouth of dogs and humans (though not sheep[6]) supporting this view[6]. The presence of carbon dioxide is also consistent with a lipid, amino acid/protein and carbohydrate component in this sample. Notably, these findings did not reveal key biomarkers typically observed in soils[20,23]. It should also be noted that highly polar material could have been present, which would not have successfully eluted from the column, or indeed volatilised sufficiently in the probe.

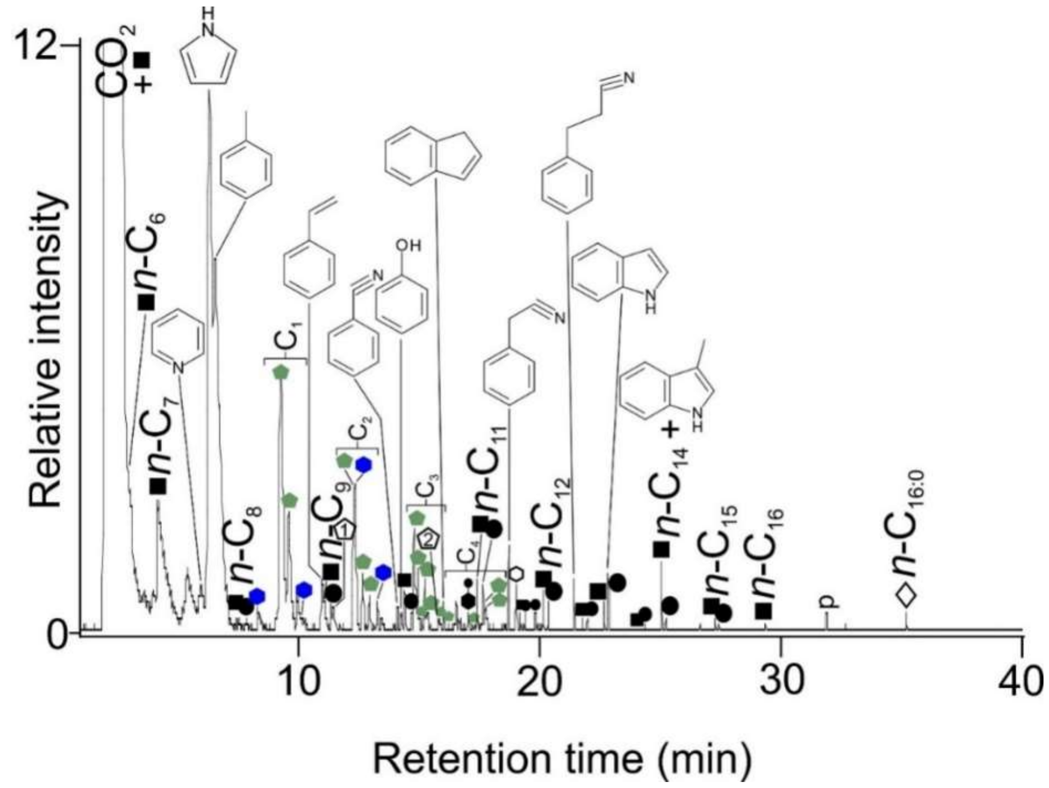

Supplementary Figure 12. Reconstructed total ion chromatogram of the pyrogram (pyrolysis profile) (610 °C for 10s) of sample DL5119, after thermal desorption (310 °C for 10 s). Key to Fig. 12: Peak identities (x indicates carbon chain length): filled squares, Cx indicates alkenes; filled circles, Cx indicates alkanes; open diamond, Cx:y indicates acyclic nitrile; blue filled hexagons indicates alkyl pyridines; green filled pentagons indicates alkyl pyrroles (with alkyl chain length above, Cx); filled hexagons with filled circle attached indicates alkyl phenol; open hexagon indicates alkyl indene; open pentagons with numerals inside indicates carbohydrate pyrolysis markers, 1 is 2-methyl-2-cyclopenten-1-one and 2 is 2,3-dimethyl-2-cyclopentene-1-one. Also shown are the structures of eleven aromatic compounds identified: pyridine, pyrrole, toluene, styrene, benzonitrile, phenol, indene, benzyl nitrile, benzenepropanenitrile, indole and 3-methylindole (skatole). CO_2 indicates carbon dioxide and p indicates contaminant from the pyroprobe.

15. Isbister DL5838 Sk77 BC5 IS113 incisor IS1976 (calculus: 10.95 mg)

The thermal desorption total ion chromatogram (TIC) (Fig. 13, inset) revealed a moderate amount of free, thermally extractable organic components in this sample, although it was dominated by carbon dioxide. Other free biomolecules identified included the nitrogen-

containing compounds pyridine, pyrrole, benzenepropanenitrile, indole, an unidentified derivative of benzenepropanenitrile and the $C_{16:0}$ nitrile. Also identified were a furan derivative and minor amounts of the C_{27}, C_{29} and C_{31} (trace) *n*-alkanes, with C_{29} predominating. It should be also noted, however, that highly polar material could have been present, which would not have successfully eluted from the column, or indeed volatilised sufficiently in the probe. It is not possible to determine the significance of the carbon dioxide, beyond it being indicative of a significant amount of highly oxidised organic material. The nitrogen-containing compounds may be indicative of thermally-labile proteinaceous material, with the aliphatic nitrile also indicating a lipid component. In isolation, the furan derivative is tentatively indicative of a carbohydrate constituent (but see Py-GC-MS below). Although minor, the particular *n*-alkanes, i.e. C_{27}, C_{29} and C_{31} (trace), with C_{29} predominant, suggest a higher plant wax origin[6] (see discussion).

In contrast to the thermal desorption profile, the pyrolysis-GC-MS TIC (Fig. 13) is dominated by a complex suite of organic compounds. These include a series of C_8 to C_{20} *n*-1-alkenes and *n*-alkanes, typical of unsaturated and saturated fat/oil-derived acyl lipids[5,6]. A distribution with maxima at C_{11}/C_{12} and C_{13}/C_{14} suggests the original acyl lipids may have been high in polyunsaturated fatty acids. The absence of these compounds in the TD profile provides molecular evidence for a polymeric/bound acyl lipid source. However, in addition to *n*-1-alkenes and *n*-alkanes, typical of fat/oil derived lipids, the pyrogram also displayed a series of C_{10} to C_{18} iso-methyl-1-alkenes (isoalkenes) and 2-methylalkanes (isoalkanes), presumably deriving from the branched fatty acids which are significant components of bacterial triglycerides.

There were components identified in the pyrolysate TIC which can be indicative of 'black carbon', i.e. charcoal or soot[7-10]. However, although this included the presence of naphthalenes, the dominance of toluene, rather than the normally dominant aromatic hydrocarbon benzene, is notably atypical of chars[7-10]. The markers identified here can also be indicative of proteins[6,11] and combined with the abundant related protein markers pyrrole, benzonitrile, benzyl nitrile (phenylacetonitrile), benzenepropanenitrile and indoles[6,11] identified this is likely to be the main origin of these compounds in this sample, pointing to a substantial protein-derived component[6,11]. This is also corroborated by the presence of aliphatic nitriles (carbon numbers $C_{14:0}$, $C_{16:0}$ (max) and $C_{18:0}$) suggesting a lipid (fat/oil) in this sample which has combined with protein-derived nitrogen to produce these moieties[5]. The proteinaceous origin for the aromatic markers is further corroborated by the significant

abundance of phenol, 2-methylphenol (*o*-cresol), 3-methylphenol (*m*-cresol), 4-methylphenol (*p*-cresol) (interestingly, with the 3-methyl isomer being markedly predominant [*cf.* DL715]), and similar amounts of 2,4-dimethylphenol and 3,4-dimethylphenol, all of which, collectively, are indicative of the amino acid tyrosine[12-14]. With an absence of guaiacol itself (all isomers) and other guaiacol moieties characteristic of lignin[15,16], the possibility that the phenols may originate from wood or woody plant material can be excluded. Alkyl phenols have also been observed in wood smoke and smoked foods[17,18], however the absence of methoxyphenols, diagnostic of lignin-derived smoke, means this possibility can also be discounted. Taken together, the presence of toluene, styrene and phenylacetonitrile (benzyl nitrile) can indicate the amino acid phenylalanine[12,13]. In addition, the major constituents indole and 3-methylindole (skatole), with a lesser but significant amount of a C_2 indole (3-ethylindole?), were also identified, which are consistent with a tryptophan amino acid source[12,13,19,20]. Proteins rich in the amino acids tyrosine, phenylalanine and tryptophan include meat, fish and dairy products, although the presence of bacterial markers means the possibility that at least a partial origin could be microbial cannot be excluded.

However, highly notable in this context is the additional presence of notably significant amounts of the more unusual alkyl pyrroles. C_1 alkyl pyrroles: 2- and 3-methylpyrroles; C_2 alkyl pyrroles: 2- and 3-ethylpyrroles, 2,4-, 2,3- and 3,4-dimethyl-pyrroles; C_3 alkyl pyrroles: 2-ethyl-4(?)-methylpyrrole, 2-ethyl-3(?)-methylpyrrole, 4-ethyl-2-methylpyrrole, 3-ethyl-4-methylpyrrole, 2,3,5-trimethylpyrrole, 2,3,4-trimethylpyrrole; C_4 alkyl pyrroles: three unidentified propylmethylpyrroles, five unidentified ethyldimethylpyrroles, 2,5(?)-diethylpyrrole, 2,4(?)-diethylpyrrole, 2,3(?)-diethylpyrrole, 3,4(?)-diethylpyrrole 4-ethyl-2,3-dimethylpyrrole, 3-ethyl-2,4-dimethylpyrrole and 2,3,4,5-tetramethylpyrrole; C_5 alkyl pyrroles: 3-methyl-4-isobutylpyrrole, 2,3(?)-diethyl-5?-methylpyrrole, 2,3-diethyl-4-methylpyrrole, 2,3-dimethyl-4-n-propylpyrrole and 3-ethyl-2,4,5-trimethylpyrrole; C_6 alkyl pyrroles: 3-ethyl-4-isobutylpyrrole, 3-methyl-4-neopentylpyrrole and 2-methyl-3-ethyl-4-n-propylpyrrole. Collectively, these specific C_1, C_2, C_3, C_4, C_5 and C_6 alkyl pyrroles are characteristic of a significant tetrapyrrole/porphyrin-derived origin and are indicative of an algal source observed in fossils and expected in similarly diagenetically altered archaeological material[11,21,22]. Notable also in this context were the presence of the moderately abundant nitrogen containing aromatic compounds 1-H-pyrazole-4-carbonitrile, ethylcyanobenzene, 2-pyridinecarbonitrile, tolyl isocyanide, a C_2 and C_4 pyrimidine, along with pyridine and its methyl-, dimethyl- and trimethyl derivatives (these pyridines can result

from the pyrolysis of polypeptides containing the amino acid alanine[23]). The presence here of the abundant protein markers and specifically the C_1 to C_6 alkyl pyrroles, combined with the particular lipid profile and specific carbohydrate markers (see below) identified, indicate the presence of a macroalgae, i.e. seaweed, in this sample[21,22,24] (see discussion).

The thermally-derived carbohydrate markers 2-methylfuran, 2-methyl-2-cyclopenten-1-one and 2,3-dimethyl-2-cyclopenten-1one were identified as significant components, along with an unidentified carbohydrate marker. That they were absent in the TD profile points to their originating from the original polymeric carbohydrate source (e.g. starch)[25]. Although levoglucosan (1,6-anhydro-β-D-glucopyranose) is usually the major component in the thermally-derived products of carbohydrates analysed by TD/Py-GC-MS, its absence from the TD profile here is consistent with the removal of hydroxyl groups in the original carbohydrate polymer, so preventing its formation in the probe. The additional absence of the normally significant carbohydrate markers 2-hydroxy-3-methyl-2-cyclopenten-1-one and 3-hydroxy-2-methyl-2-cyclopenten-1-one observed in pure standard material (glycogen, starch and cellulose) would also support this interpretation. This shows a strong matrix effect likely to be due to the inorganic component of the calculus and the microflora in the mouth. This dehydration/dehydroxylation has also been observed in dog calculus and 19th century human calculus[6], where other markers, e.g. a prevalence of nitriles, with amides very minor, deriving from dehydrating conditions in the mouth of dogs and humans (though not sheep[6]) supporting this view[6]. The presence of carbon dioxide is also consistent with a lipid, amino acid/protein and carbohydrate component in this sample. Notably, these findings did not reveal key biomarkers typically observed in soils[20,23]. It should also be noted that highly polar material could have been present, which would not have successfully eluted from the column, or indeed volatilised sufficiently in the probe.

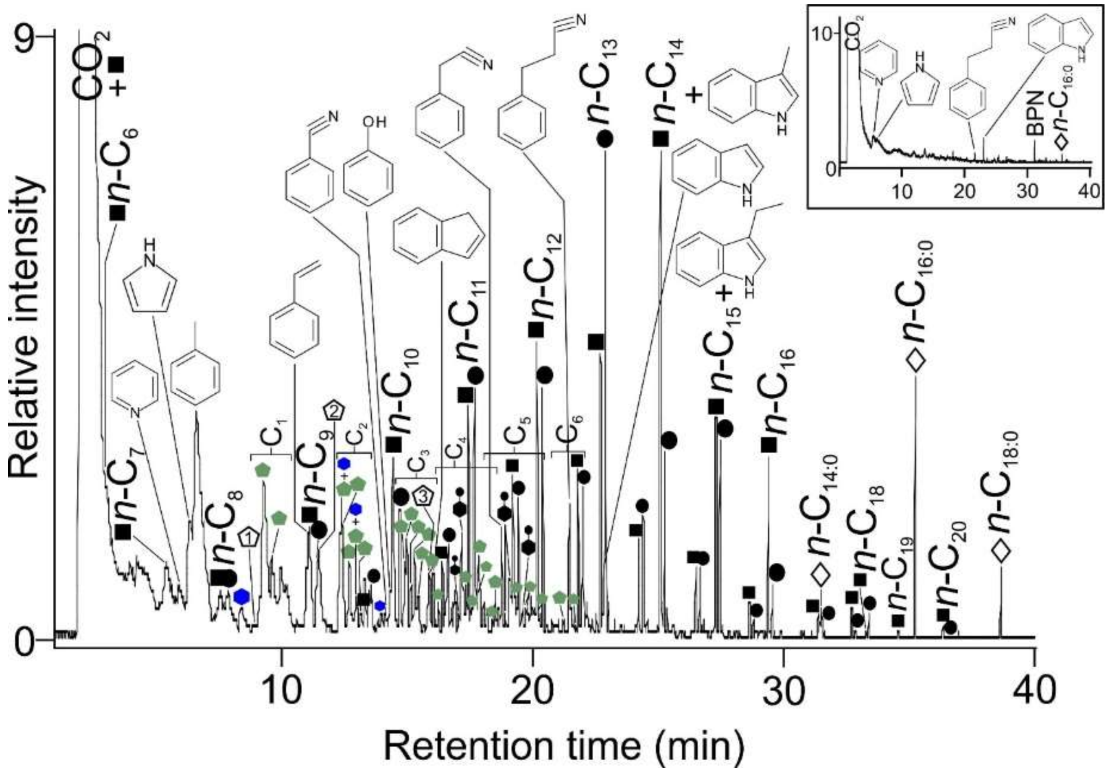

Supplementary Figure 13. Reconstructed total ion chromatogram of the pyrogram (pyrolysis profile) (610 °C for 10s) of sample DL5838, after thermal desorption (310 °C for 10 s). Key to Fig. 13: Peak identities (x indicates carbon chain length): filled squares, Cx indicates alkenes; filled circles, Cx indicates alkanes; open diamonds, Cx:y indicates acyclic nitriles; blue filled hexagons indicates alkyl pyridines; green filled pentagons indicates alkyl pyrroles (with alkyl chain length above, Cx); filled hexagons with filled circles attached indicates alkyl phenols; open pentagons with numerals inside indicates carbohydrate pyrolysis markers, 1 is 2-methylfuran, 2 is 2-methyl-2-cyclopenten-1-one and 3 is 2,3-dimethyl-2-cyclopentene-1-one. Also shown are the structures of twelve aromatic compounds identified: pyridine, pyrrole, toluene, styrene, benzonitrile, phenol, indene, benzyl nitrile, benzenepropanenitrile, indole, 3-methylindole (skatole) and 3-ethylindole. CO_2 indicates carbon dioxide. Inset displays a reconstructed total ion chromatogram of the thermal desorption profile (310 °C for 10 s) of this sample. Peak identities: BPN indicates a benzenepropanenitrile derivative; open diamond, Cx:y indicates an acyclic nitrile. Also shown are four aromatic compounds: pyridine, pyrrole, benezenepropanenitrile and indole. CO_2 indicates carbon dioxide.

Discussion Isbister

Fifteen samples of dental calculus from fifteen individuals from Isbister, Orkney were analysed by sequential thermal desorption-gas chromatography-mass spectrometry (TD-GC-MS) and pyrolysis-gas chromatography-mass spectrometry (Py-GC-MS).

The TD-GC-MS analyses revealed very little organic material in thirteen of the fifteen samples. Of the two samples in which 'free' organic compounds were detected, sample DL2104 revealed the 2,5-diketopiperazine derivative (DKP) of proline-glycine (pro-gly), along with a far lesser amount of the DKP of proline-alanine (pro-ala). These two biomarkers, along with carbon dioxide, dominated the thermal desorption profile as the only 'free' organic compounds detected, indicating little free, thermally extractable, organic components in this sample. These thermally generated DKPs suggest the amino acids proline, glycine, and alanine were constituents in a protein[5,12,13], presumably from food consumed by this individual (see below). Eight of the samples produced acetonitrile at the chosen thermal desorption temperature, which may suggest a proteinaceous component high in phenylalanine and/or tyrosine (see below). The only other calculus sample containing 'free' organic constituents was DL5838; in addition to the ubiquitous carbon dioxide, nitrogen-containing compounds dominated the profile; these included pyridine, pyrrole, benzenepropanenitrile, indole, an unidentified derivative of benzenepropanenitrile and the $C_{16:0}$ nitrile. These nitrogen-containing compounds may be indicative of thermally-labile proteinaceous constituent, with the aliphatic nitrile also indicating a lipid component which has combined with the protein[5]. A furan derivative was also identified, which is tentatively indicative of a carbohydrate constituent (but see Py-GC-MS below).

Albeit in minor amounts, the C_{27}, C_{29} and C_{31} (trace) *n*-alkanes, with the C_{29} *n*-alkane predominating, were also detected. This is indicative of a higher plant wax origin (e.g. from leafy greens)[6]. Moreover, although the interpretation has to be somewhat tentative due to the relatively low abundance of these compounds, the C_{29} *n*-alkane is notably dominant in Brassicaceae epicuticular leaf waxes such as *Brassica oleracea* (cabbage)[27-29] and *Coincya* spp.[30] and this has also been found in archaeological studies where *Brassica* leaves (cabbage) have been identified[29,31]. In modern and archaeological studies the C_{29} *n*-alkane accounted for over 90% of the hydrocarbon fraction, with the C_{29} *n*-alkane/total *n*-alkanes ratios being: ~95:5 and ~96:4 for *Brassica* and *Coincya* respectively[27-30]. The C_{29} *n*-alkane/total *n*-alkanes ratio for DL5838 is 96:4 and is consistent with a Brassicaceae source. However, it should be noted that *Brassica* spp. vegetables such as cabbages and turnips would not have been

available in Neolithic Orkney, since they are not native[32]. Those Brassicaceae plants which are native to Orkney and possible sources of leafy greens include *Cakile maritima* (sea rocket), *Crambe maritima* (sea kale), Cochlearia officinalis (scurvy grass) and *Nasturtium officinale* (watercress)[32]. However, the very high C_{29} *n*-alkane predominance has been linked to glaucousness in Brassicaceae leaf wax[30]. This is notable since sea kale (*Crambe maritima*) has a distinct blue-grey-green waxy (glaucous) appearance in contrast to the other Brassicaceae native to Orkney[33-35], reflecting the significant amount of wax on its leaves, with the dominance of the C_{29} *n*-alkane making the wax monolayers/sheets more uniform and so providing a more protective and less permeable barrier[30]. This protects against light, temperature and wind, but the amount of epicuticular leaf wax has also been linked to protection against salt stress[36], with an increase in wax production linked to significant salt exposure[36], which is consistent with *Crambe maritima*'s habitat of shingle beaches. Moreover, it's interesting to note that the distribution of *Crambe maritima* is limited in northern Scotland and Orkney, to South Ronaldsay's east coast shingle beaches, only a few miles from Isbister itself[37]. Sea kale is mentioned by Pliny the Elder as a sailor's anti-scurvy remedy[38] and by Mrs Beeton as a 'type of asparagus'[39], following botanist William Curtis's recommendation of its use as a vegetable in Britain in 1799[34] and its use as a vitamin C-rich vegetable is well recognised, despite its use dramatically falling in recent times due to over-exploitation[33-35].

In contrast to the TD-GC-MS analyses, the Py-GC-MS results revealed appreciable amounts of organic material in thirteen of the fifteen calculus samples, with nine containing moderate to major abundances of organic compounds. This indicates that although the cool environment on Orkney has allowed the survival of a wide range of biomolecules relating to fats/oil, proteins and carbohydrates (see below), diagenesis and biodegradation have resulted in the loss of most, if not all, free biomarkers. In contrast, bound/polymeric biomacromolecules (some clearly resulting from pre- and/or post-depositional biochemical reactions between the constituents of the original foods consumed) have been appreciably well preserved, albeit the more resilient moieties and compound classes are likely to have been preferentially preserved.

The pyrolysis-GC-MS analyses were dominated by a complex suite of organic compounds indicative of lipid (fat/oil), protein and carbohydrate components. These include a series of C_8/C_9 to C_{16} (up to C_{20} where more organic material was present) *n*-1-alkenes and *n*-alkanes, typical of unsaturated and saturated fat/oil-derived acyl lipids[5,6]. A bimodal distribution with

maxima at C_{11}/C_{12} and C_{14} combined with abundant short chain (C_4-C_7) *n*-1-alkenes in eleven of the fifteen samples analysed suggests the original acyl lipids in these samples are likely to have been high in polyunsaturated fatty acids. The absence of these compounds in the TD profile provides molecular evidence for a polymeric/bound acyl lipid source. However, in addition to *n*-1-alkenes and *n*-alkanes, typical of fat/oil derived lipids, the pyrograms also displayed a series of C_{10} to C_{16} (up to C_{20} where more organic material was present) iso-methyl-1-alkenes (isoalkenes) and 2-methylalkanes (isoalkanes), presumably deriving from the branched fatty acids which are significant components of bacterial triglycerides.

In thirteen of the samples analysed there were components in the pyrolysate which can be indicative of 'black carbon', i.e. charcoal or soot[7-10]. However, although this included the presence of naphthalenes, the dominance of toluene, rather than the normally dominant aromatic hydrocarbon benzene, is notably atypical of chars[7-10]. The markers identified here can also be indicative of proteins[6,11] and combined with the abundant related protein markers pyrrole, benzonitrile, benzyl nitrile (phenylacetonitrile), benzenepropanenitrile and indoles[6,11] identified in six of the samples this is likely to be the main origin of these compounds in these samples, pointing to a substantial protein-derived component[6,11]. This is also corroborated by the presence of aliphatic nitriles (carbon numbers $C_{14:0}$, $C_{16:0}$ (max) and $C_{18:0}$) suggesting a lipid (fat/oil) in these same six samples which has combined with protein-derived nitrogen to produce these moieties[1]. The proteinaceous origin for the aromatic markers is further corroborated by the significant abundance of phenol, methylphenols, and dimethylphenols, all of which, collectively, are indicative of the amino acid tyrosine[12-14] (in turn, also consistent with the acetonitrile in the TD-GC-MS analyses). With an absence of guaiacol itself (all isomers) and other guaiacol moieties characteristic of lignin[15,16], the possibility that the phenols may originate from wood or woody plant material can be excluded. Alkyl phenols have also been observed in wood smoke and smoked foods[17,18], however the absence of methoxyphenols, diagnostic of lignin-derived smoke, means this possibility can also be discounted. Taken together, the presence of toluene, styrene and phenylacetonitrile (benzyl nitrile) can indicate the amino acid phenylalanine[12,13,40], which is also consistent with the acetonitrile observed in the thermal desorption profiles of a number of the Isbister calculus samples. In addition, the major constituents indole and 3-methylindole (skatole), with a lesser amount of a C_2 indole (3-ethylindole?) in the two most organically-rich samples, were also identified, which are consistent with a tryptophan amino acid source[12,13,19,20]. Proteins rich in the amino acids tyrosine, phenylalanine and tryptophan include meat, fish and dairy products,

although without further biomolecular corroboration the presence of bacterial markers means the possibility that at least a partial origin could be microbial cannot be excluded.

Far more diagnostic of a particular dietary source are the particular suite of alkyl pyrroles observed in the pyrogram of six of the samples (DL42, DL2104, DL86, DL715, DL5119 and DL5838), with an alkyl chain length of up to C_6 (see Table 1). The presence of C_5 alkyl pyrroles in three of these six samples and C_6 alkyl pyrroles in two of them, in addition to the C_1 to C_4 moieties in all six, is highly notable. In sample DL5838, which contained the highest abundance of observed biomacromolecularly-derived compounds in the analyses, and therefore provided the most detailed chemical information on this source of organic material, it revealed very significant amounts of these biomarkers. This included the 2- and 3-dimethylpyrroles, 2- and 3-ethylpyrroles, 2,4-, 2,3- and 3,4-dimethylpyrroles, four ethylmethylpyrroles, two trimethylpyrroles, two propylmethylpyrroles, seven ethyldimethylpyrroles, four diethylpyrroles, 2,3,4,5-tetramethylpyrrole, a methylisobutylpyrrole, two diethylmethylpyrroles, a dimethyl-*n*-propylpyrrole, 3-ethyl-2,4,5-trimethylpyrrole, and three C_6 alkyl pyrroles, tentatively identified as 3-ethyl-4-isobutylpyrrole, 3-methyl-4-neopentylpyrrole and 2-methyl-3-ethyl-4-*n*-propylpyrrole. These specific C_1 to C_6 alkyl pyrroles are highly diagnostic of the presence of a significant tetrapyrrole component. Their porphyrin-derived origin, and the relative abundance of some of the key biomarkers, is indicative of an algal source observed in fossils and expected in similarly diagenetically altered archaeological material[11,21,22,] (see below). Although C_1 and C_2 alkyl pyrroles can also derive from protein-derived amino acids such as glycine, proline, hydroxyproline, serine and glutamic acid[12-14,20,40,41], the C_3+ alkyl pyrroles identified in these six samples point to a tetrapyrrole source[21,22,40,41]. Moreover, the same specific C_1 to C_5 alkylpyrroles identified in the Py-GC-MS of an algal kerogen[21,22] were observed in DL5838 (see Table 2).

As a ‘chemical fossil’, algae can be expected to reveal a distinctive suite of alkylpyrroles when analysed by pyrolysis-GC-MS[21,22]. Key research in this area studied the composition of kerogen, i.e. macromolecular fossil organic matter, from the Miocene Monterey Formation, California, USA. The Monterey Formation is an area of marine deposits, known for its many fossils, most notably a significant number of large soft-bodied seaweeds, which are rarely found as fossils elsewhere[42]. These largely occur in the siliceous shales (diatomite), which are

formed from diatoms and other silica-producing microalgae[43] and a sediment sample was taken from the Upper Miocene Monterey Formation siliceous-carbonate rock at Santa Barbara County for organic geochemical investigation[21,22] where, notably, fossil seaweed from the Upper Miocene has also been observed. Although microalgae are reasonably understood to be the primary source of organic matter in the Monterey kerogen, based on geological and organic geochemical data to date[43], macroalgae as a source of kerogen has usually been overlooked[44,45], with several studies showing macroalgae (Rhodophyta; red seaweeds) to be a major source of kerogen in some sedimentary environments,[44-46]. The organic geochemical investigation of the Monterey kerogen using Py-GC-MS revealed a highly distinctive suite of alkylpyrroles, which can be related chemically to the monomer building blocks constituting the tetrapyrrole pigments of chlorophylls, protoporphyrin IX and bilirubin[21,22]. The study concluded that the abundant tetrapyrrole pigments were likely to derive from the major algal contribution to the sediments of the Monterey kerogen, preservation characteristics or a combination of the major algal input and the particular preservation conditions, with the same alkylpyrrole distributions also observed in similar kerogens from immature upwelling sedimentary environments such as the Namibian Shelf[21,22]. This is consistent with the geological and organic geochemical evidence for abundant marine algae within the Monterey Formation, where ‘Organic matter is overwhelmingly amorphous marine algal debris’[43].

More specifically, the highly unusual alkyl pyrroles, 3-methyl-4-isobutylpyrrole, 2,3-dimethyl-4-*n*-propylpyrrole, 3-ethyl-4-isobutylpyrrole, 3-methyl-4-neopentylpyrrole and 2-methyl-3-ethyl-4-*n*-propylpyrrole (see Table 1) are known to derive from the C_8 side chain of bacteriochlorophylls c and d[47] and are biomarkers for the relatively rare bacteriochlorophylls c and d which are found in green bacteria such as *Chloroflexus* sp.[47] and are known to naturally occur in association with intertidal red seaweeds such as *Porphyra umbilicalis*[48-49]. Overall the six samples display a remarkably similar pyrolysis profile to algal-derived kerogen previously studied via very similar organic geochemical techniques[21,22].

Supplementary Table 2. C_1 to C_6 alkyl pyrrole compounds[1] present in the Py-GC-MS of the Isbister dental calculus samples

Compound (alkyl pyrrole)	DL42	DL2104	DL86	DL715	DL5119	DL5838
2-methylpyrrole	Major	major	major	major	Major	major
3-methylpyrrole	√√√√√√	√√√√√√	√√√√√	√√√	√√√√	√√√
2-ethylpyrrole	√√√	√√√√	√√	√√√	√√√√	√√√
3-ethylpyrrole	√√	√√	√√	√	√√	√
2,5-dimethylpyrrole	-	-	-	-	-	-
2,4-dimethylpyrrole	√√√	√√√	√√√	√√√	√√	√√√
2,3-dimethylpyrrole	√√√	√√√	√√	√√	√√√	√√
3,4-dimethylpyrrole	√	√	√	√	√	√
ethylmethylpyrrole (2-Et-4(?)-Me)	-	-	-	√√	√√√	√√
ethylmethylpyrrole (2-Et-3(?)-Me)	√√	√√√	√√	√√	√√	√√√
4-ethyl-2-methylpyrrole	√√	√	√	√	√	√
2,3,5-trimethylpyrrole	√	√	√	√√	√√	√√
3-ethyl-4-methylpyrrole	√	√	√	√	√	√√
2,3,4-trimethylpyrrole	-	-	-	√	√	√
Propylmethylpyrrole	√	√	√	√	√	√
Ethyldimethylpyrrole	-	-	-	-	-	√
2,5(?)-diethylpyrrole	-	-	-	√	√	√

Ethyldimethylpyrrole	-	-	-	-	√	√
Propylmethylpyrrole	-	-	-	-	√	√
2,4(?)-diethylpyrrole	-	-	-	-	-	√
Ethyldimethylpyrrole	-	-	-	-	-	√
Ethyldimethylpyrrole	√	√	√	√	-	√
4-ethyl-2,3-dimethylpyrrole	√	√	-	√	√	√
3-ethyl-2,4-dimethylpyrrole	-	-	-	√	-	√
2,3(?)-diethylpyrrole	-	-	-	√	√	√
Propylmethylpyrrole	-	-	-	-	-	√
3,4(?)-diethylpyrrole	-	-	-	-	-	√
Ethyldimethylpyrrole	-	-	-	-	-	√
2,3,4,5-tetramethylpyrrole	-	-	-	-	-	√
3-methyl-4-isobutylpyrrole	-	-	-	-	-	√
2,3?-diethyl-5-methylpyrrole	-	-	-	√	√	√
2,3-diethyl-4-methylpyrrole	-	-	-	-	-	√
2,3-diethyl-4-*n*-propylpyrrole	-	-	-	√	-	√
3-ethyl-2,4,5-trimethylpyrrole	-	-	-	-	√	√
3-ethyl-4-isobutylpyrrole	-	-	-	-	-	√
2,3-diethyl-4,5-dimethylpyrrole	-	-	-	-	-	-
2,4-diethyl-3,5-dimethylpyrrole	-	-	-	-	-	-
3-methyl-4-neopentylpyrrole	-	-	-	√	-	√
2-methyl-3-ethyl-4-*n*-proylpyrrole	-	-	-	-	-	√

[1]After Sinninghe Damste *et al.* 1992[22]

Supplementary Table 3. C_1 to C_5 alkyl pyrrole compounds present in the Py-GC-MS of natural sources of tetrapyrroles[1] and Isbister dental calculus sample DL5838

**Compound (alkyl pyrrole)**	**DL5838**	**Fossil algae (M. K.ǂ)**	**Bilirubin**	**Chlorophyll-*a***	**Protoporphyrin-IX**
2-methylpyrrole	(√√√√√*)	√	√	√	√
3-methylpyrrole	(√√√*)	√√	√	√√	√√
2,5-dimethylpyrrole	-	√	√	-	√
2,4-dimethylpyrrole	√√√	√√	√	√	√√
2,3-dimethylpyrrole	√√	√√	√	√	√√
3,4-dimethylpyrrole	√	√√	√	√	√√
ethylmethylpyrrole	√√	√	√	√	√
ethylmethylpyrrole	√√√	√	√	√	√
4-ethyl-2-methylpyrrole	√	√	√	√	√
2,3,5-trimethylpyrrole	√√	√√	√√	√	√√
3-ethyl-4-methylpyrrole	√√	√√√	√√	√√√	√√
2,3,4-trimethylpyrrole	√	√√	√√	√	√√√
ethyldimethylpyrrole	√	√	√	√	√
ethyldimethylpyrrole	√	√	√	√	√
4-ethyl-2,3-dimethylpyrrole	√	√√	√√	√√	√√

3-ethyl-2,4-dimethylpyrrole	√	√√√	√√√	√√√	√√√
2,3-diethyl-4-methylpyrrole	√	√	√	√	√
3-ethyl-2,4,5-trimethylpyrrole	√	√√	√√	√√	√√

[1]After Sinninghe Damste *et al.* 1992[22]

[ǂ]Monterey kerogen (Sinninghe Damste *et al* 1992)[22]

*High due to elevated levels of nitrate in environment (see text)

Having identified specific algal biomarkers in six of the fifteen samples, with a further six producing fewer organic compounds, but tentatively indicative of the same biological source, it is appropriate to consider the other compound classes (proteins, lipids and carbohydrates) in this context. Although diagenetic changes may have altered the original protein composition, protein markers observed in the Py-GC-MS of the six ‘algal’-containing samples suggest those high in the aromatic amino acids phenylalanine, tyrosine and tryptophan, in addition to significant levels of glycine, alanine, cysteine, proline (and possibly hydroxyproline), serine, arginine, glutamic acid and aspartic acid (see Table 3), were significant in the original food consumed. The TD-GC-MS was also indicative of a proteinaceous component relatively high in phenylalanine and glycine, proline and alanine. This is highly significant when combined with the presence of the abundant algal markers; seaweeds (macroalgae), especially red types, can be high in protein (up to 30-50% dry weight)[50-54] and green, brown and red types contain significant amounts of these particular amino acids[51-55], with some species being higher in phenylalanine, tyrosine and tryptophan than egg proteins[53]. Seaweed is known to have been exploited for food in antiquity[56], although its consumption by humans on Orkney seems only to have been tentatively considered to date[2,57].

Supplementary Table 4. Protein biomarkers[1] present in the Py-GC-MS of the Isbister dental calculus samples (relative %) and the protein-based amino acid sources from which they derive

Compound (protein pyrolysis biomarkers)	Amino acid source	DL42	DL2104	DL86	DL715	DL5119	DL5838
Pyrrole	pro	9.1	13.4	13.4	13.2	41.0	8.7
2-methylpyrrole	gly, pro, hypro, ser, glu, tetrapyr	6.8	6.9	9.7	15.7	13.9	13.2
3-methylpyrrole	gly, pro, hypro, ser, glu, tetrapyr	3.2	3.2	3.8	4.7	4.1	4.5
2-ethylpyrrole	gly, pro, hypro, ser, glu, tetrapyr	0.9	1.4	1.4	3.0	6.6	4.7
3-ethylpyrrole	gly, pro, hypro, ser, glu, tetrapyr	0.4	0.6	0.4	0.9	0.6	1.0
Pyridine	ala (asp?)	4.2	3.2	4.1	2.4	2.1	2.1
4-methylpyridine	ala, cys (asp?)	1.3	2.6	1.6	0.6	1.0	2.1
2-methylpyridine	ala (asp?)	1.1	1.6	0.5	0	0.2	0
2,4-dimethylpyridine	ala (asp?)	0.8	1.4	1.8	0.6	0	0.5
2,6?-dimethylpyridine	ala (asp?)	0.1	0.4	0	0	0	0.1
a C3 pyridine	ala (asp?)	0.4	0.5	0.1	0.3	0	1.6
1H-pyrazole-4-carbonitrile	arg(?)	0.5	0.4	0.4	0.4	0.05	0.1
2-pyridinecarbonitrile	arg(?)	0.8	0.8	0.2	0.2	0.4	0.7

Pyrimidines	arg	0.2	0.3	0	0.2	0	1.6
Imidazoles	arg, glu?	0	0	0	0	0	0.5
Toluene	phe	34.1	35.6	39.4	40.4	22.6	31.4
Ethylbenzene	phe	0.6	0.8	4.1	2.3	0.9	3.5
o+m-xylene	phe	1.1	0.9	1.4	1.4	0.2	1.4
p-xylene	phe	0	0	0	0	0	0
Styrene	phe	3.8	2.6	3.6	3.1	1.3	2.1
Benzonitrile	phe	1.5	0.9	1.1	0.6	0.6	0.7
ethylcyanobenzene	phe	0.4	0.4	0.2	0.2	0	0.3
benzyl nitrile	phe	2.5	2.2	1.2	1.1	1.3	2.4
benzenepropanenitrile	phe	3.0	2.6	1.2	1.7	0.8	3.1
Phenol	tyr	4.5	3.7	1.4	0.6	0.5	0.7
2-methylphenol	tyr	1.1	0.9	0.04	0	0	0.3
4-methylphenol	tyr	4.2	2.1	0.9	0.4	0.3	0.3
3-methylphenol	tyr	0	0	0	0.1	0	2.8
3,4?-dimethylphenol	tyr	0	0	0	0	0	0.3
2,4-dimethylphenol	tyr	0.8	0.9	0	0	0	0.2
2,6-dimethylphenol	tyr	0.4	0.3	0	0	0	0
4-ethylphenol	tyr	0	0	0	0.1	0	0.5
o-tolyl isocyanide(?)	trp	0.6	0.8	0.2	0.1	0.05	0.9

Indole	trp	3.8	2.9	1.8	1.9	0.6	3.5
3-methylindole	trp	3.0	1.8	0.6	0.7	0.2	1.9
3-ethylindole	trp	0.4	0.3	0	0	0	0.5
Non/less-specific protein markers							
Benzene	-	1.1	1.1	3.8	1.4	0	0
Indene	-	1.3	0.9	1.1	1.0	0.4	1.6
a methylindene	-	1.1	0.8	0.6	0.7	0.4	0
a methylindene	-	0.6	0.3	0.04	0.1	0	0
a C_2 indene	-	0.4	0.3	0	0	0	0
Protein markers – Total Peak Area		100.1	99.8	100.08	100.1	100.1	99.8

[1]After Stankiewicz *et al* 1996[12]; Stankiewicz, Hutchins *et al* 1997[13]; Stankiewicz, Briggs *et al* 1997[14]; Poinar and Stankiewicz 1999[58].

gly = glycine; pro = proline; hypro = hydroxylproline; ser = serine; glu = glutamic acid; ala = alanine; asp = aspartic acid; cys = cysteine; arg = arginine; phe = phenylalanine; tyr = tyrosine; trp = tryptophan; tetrapyr = tetrapyrrole

Although biomarkers for aspartic and glutamic acids are relatively low given their usual high abundance in fresh seaweeds, they would be expected to be preferentially degraded when compared to the aromatic amino acids such as phenylalanine and tyrosine. The high relative abundance of the C_1 and C_2 alkyl pyrroles may be explained by high nitrate levels in the environment from anthropogenic activity (agricultural and waste products entering the coastal waters close to the areas of seaweed exploitation), which has been shown to produce high levels of serine in macroalgae (seaweeds)[59] and terrestrial plants[60-62]. Serine in the

macroalgae exposed to high nitrate levels was shown to be ~10% compared with the reference site of ~7% (the amino acid alanine, which does not produce alkyl pyrroles in Py-GC-MS, is essentially unaffected)[59]. This nitrate can also raise the $\delta^{15}N$ values; *Catenellanipae* being raised by ~3.5 to 16.5‰ where appreciably elevated levels of NO_3^- was present[59]. This translates to significantly increased levels of methyl and ethyl pyrroles in the Py-GC-MS analyses, deriving from the high serine content (their formation from serine during pyrolysis studied in some detail mechanistically[63]).

As discussed above, the bimodal distribution of *n*-1-alkenes/*n*-alkanes with maxima at C_{11}/C_{12} and C_{14}, combined with abundant short chain (C_4-C_7) *n*-1-alkenes, suggests the original acyl lipids are likely to have been high in polyunsaturated fatty acids, which is consistent with a marine – seaweed – source. Research on seaweeds has shown that green, brown and red seaweeds differ in the relative abundances of the particular unsaturated fatty acids present in their lipid component[52,64-69]. Green seaweeds are high in C_{16} and C_{18} polyunsaturated fatty acids, with C_{20} acids in minor abundance and C_{22} acids very minor or absent; brown seaweeds have less abundant C_{16} acids, with C_{18} acids being major and C_{20} acids more significant than green; red seaweeds have C_{20} polyunsaturated acids dominating, with significant amounts of C_{22} acids[52,64-69]. Although the polyunsaturated mid and long chain moieties characteristic of a marine macroalgal input were not detected, these compounds are particularly labile and susceptible to degradation. Perhaps notably, the expected biopolymeric products generated during pyrolysis if these biomolecules were incorporated into a bound organic fraction would be expected to maximise at ~C_{11}/C_{12} and ~C_{14} *n*-1-alkene/*n*-alkanes, i.e. promote a bimodal distribution of alkene/alkanes, as is observed in these samples. Moreover, previous studies specifically on the Py-GC-MS analysis of seaweed at similar pyrolysis temperatures (500/600°C) have revealed the C_{13} *n*-1-alkene and *n*-alkane in equal abundance, yet the C_{14} *n*-1-alkene in significantly higher quantity than both of these lipid markers, while the C_{14} *n*-alkane is not reported as a constituent of the pyrolysate, suggesting it is minor or absent[70]; this is mirrored in the six samples containing macroalgal biomarkers. In addition, the same study revealed significant amounts of the protein markers phenol, 3-methyl phenol, benzenepropanenitrile, indole, 3-methylindole, and hexadecanenitrile, as also observed in the six Isbister samples. The microbial biomarkers (e.g. branched hydrocarbons) observed are consistent with the iso-and anteiso-branched chain fatty acids normally present in marine macroalgae (seaweeds)[6]. Although other possible food sources high in polyunsaturated fatty acids could also explain this molecular profile, the combination of

tetrapyrrole, protein and lipid biomarkers identify a seaweed component in these samples. Looking in more detail, the pyrolysis lipid profiles observed can be generated in appreciable amounts by the $C_{20:4}$ and $C_{20:5}$ fatty acids, but can also be indicative of significant abundances of $C_{18:3}$ and $C_{18:4}$ fatty acids, as are present in seaweeds[52,64-69]; differences can, albeit somewhat tentatively, be related to typical expected biomarkers profiles from green, brown and red seaweeds. In this context, it is interesting to note that when relative abundance of the sum of *n*-1-alkenes/*n*-alkanes for carbon chain numbers C_8 to C_{16} is plotted for green, brown, red and sample DL5838 (the sample with the highest abundance of organic material and macroalgal biomarkers), the distributions suggest the Isbister sample is most similar to red seaweeds (Fig 14).

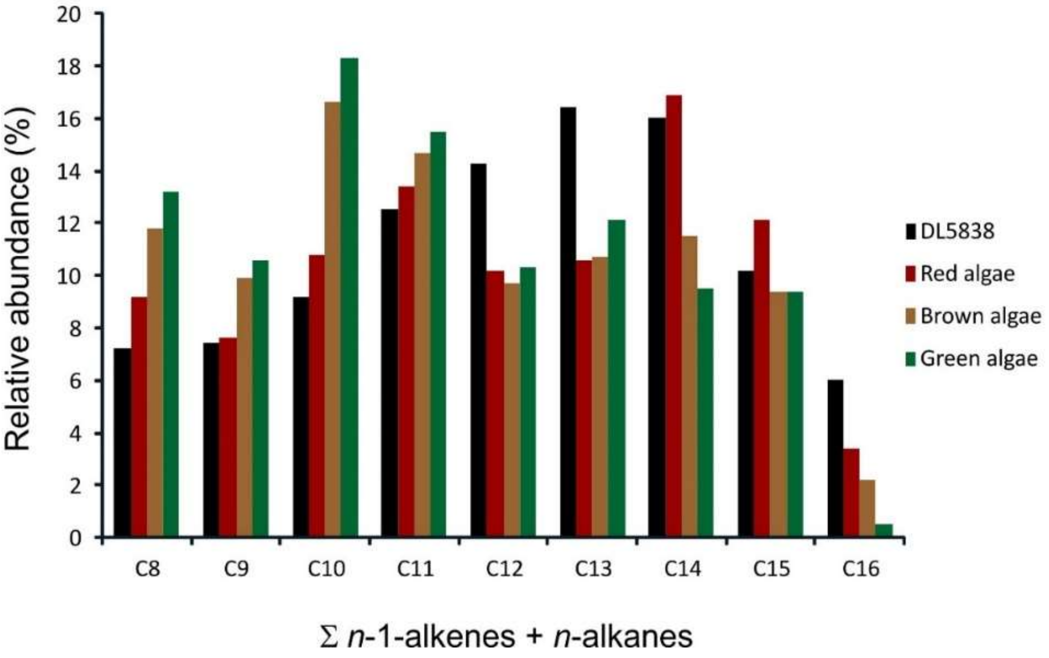

Supplementary Figure 14. Histogram showing a comparison of the sum of the *n*-1-alkene + *n*-alkane distributions (Σ *n*-1-alkene + *n*-alkane) (C_8 to C_{16} carbon numbers) of calculus sample DL5838 from Isbister (highest organic content and most abundant macroalgal biomarkers) and modelled relative abundances predicted for red, brown and green macroalgae (seaweed). These are based on their fatty acid composition and subsequent oxidative polymerisation and defunctionalisation, as observed in archaeological and ancient biomacromolecules – ‘chemical fossils’ – in previous studies[5,71,72]. Source data are provided as a Source Data file.

Although the C_{12} and C_{13} moieties are somewhat high, this may be explained either by a small input from marine fish where those with a high oil content, in particular, contain high levels of docosahexaenoic acid ($C_{22:6}$; DHA)[73-77], which is normally low in macroalgae, or a red seaweed high in both $C_{20:4}$ and $C_{22:6}$, such as *Palmaria palmata* (Dulse)[64]. This would be expected to increase the relative abundance of the C_{12} and C_{13} moieties.

Previous research on human remains from Isbister has suggested that these individuals had a largely terrestrial diet, although it has been conceded that females may have exploited seaweed or shellfish as part of their diet[2]. This same research has observed: 'The values (–19.9 to –21.2‰) suggest little or no use of marine protein', this despite the location of the monument directly on the shore, the abundant remains of marine fish found in the tomb and the historic importance of fishing in Orkney (Colley 1983a, 1983b)[57]. However, this appears to ignore the fact that macroalgae (seaweeds) can have a very wide range of stable isotopic values for $\delta^{13}C$ (-12 to -36), despite this - and the possibility of some seaweed consumption by females - being noted in connection with this research[2,78]. In fact, the isotopic data could also be consistent with a significant seaweed diet (a largely seaweed human diet at Isbister could produce the values previously reported for the site[2,57]), with a small input from marine fish[79].

Importantly, the findings presented here show seaweed (with specific biomarkers consistent with red seaweeds), and therefore marine protein, formed a significant part of the diet for both males and females at Isbister. Although diagenesis means the biomolecular information is not necessarily reflecting the original diet consumed, it nevertheless shows the organic material entrapped within the dental calculus is providing profound insights into palaeodiets not evident by other means. Moreover, it shows the exploitation of a nutritious and readily available food resource, which has been surprisingly underplayed, if not dismissed, as a valued staple in Neolithic Orkney.

National Museums Scotland human remains collection

Fifteen samples of dental calculus taken from fourteen individuals from eight sites in Scotland, curated by National Museums Scotland, were analysed by sequential thermal desorption-gas chromatography-mass spectrometry (TD-GC-MS) and pyrolysis-gas chromatography-mass spectrometry (Py-GC-MS). This technique facilitates the identification of both free/unbound and bound/polymeric organic components. All fifteen calculus samples

produced very little free organic material. The fifteen samples revealed highly variable amounts of a bound/polymeric organic constituent.

Quanterness passage tomb, Orkney

Quanterness is a large, Maeshowe-type passage tomb on the mainland island of Orkney. It was excavated by Colin Renfrew in 1972–74[80]. It is located in one of Europe's richest Neolithic landscapes containing numerous chambered tombs, two stone circles, the stone-built Neolithic village of Skara Brae and the huge ceremonial settlement of Ness of Brodgar whose *floruit* was c. 3200–2800 BC[81]. Wild resources including large numbers of bones from inshore and deep-sea fish, birds and wild plants were found at some of these sites, in addition to agricultural produce.

16. Quanterness passage tomb, Orkney (NMS unreg): Bag 10/Box 4 Bag 209 (bag 4) 1139-01: M1 right lingual (calculus: 1.55 mg)

The thermal desorption total ion chromatogram (TIC) revealed carbon dioxide as the only detectable component, indicating the essential absence of free, thermally extractable organic components in this sample. It should be noted, however, that highly polar material could have been present, which would not have successfully eluted from the column, or indeed volatilised sufficiently in the probe. It is not possible to determine the significance of the carbon dioxide, beyond it being indicative of oxidised organic material.

The pyrolysate TIC revealed carbon dioxide, C_4 and C_5 1-alkenes, cyclopentadiene and benzene as the only detectable components. The dominance of carbon dioxide, combined with the relatively minor abundance of the few small organic compounds present, suggests a highly oxidised sample. It is not possible to determine the significance of the carbon dioxide, beyond it being indicative of oxidised organic material and the other compounds are too low an abundance and too ubiquitous to provide any meaningful interpretation of their presence, beyond being indicative of an organic origin. It should be noted, however, that highly polar material could have been present, which would not have successfully eluted from the column, or indeed volatilised sufficiently in the probe.

17. Quanterness passage tomb, Orkney (NMS unreg): Box 10/Box L/Bag 216 (bag 2) 1190-A?1: M2 left labial (calculus: 2.76 mg)

The thermal desorption total ion chromatogram (TIC) revealed carbon dioxide as the only detectable component, indicating the essential absence of free, thermally extractable organic components in this sample. It should be noted, however, that highly polar material could have been present, which would not have successfully eluted from the column, or indeed volatilised sufficiently in the probe. It is not possible to determine the significance of the carbon dioxide, beyond it being indicative of oxidised organic material.

The pyrolysate TIC revealed carbon dioxide, C_4 and C_5 1-alkenes, cyclopentadiene and benzene as the only detectable components. The dominance of carbon dioxide, combined with the relatively minor abundance of the few small organic compounds present, suggests a highly oxidised sample. It is not possible to determine the significance of the carbon dioxide, beyond it being indicative of oxidised organic material and the other compounds are too low an abundance and too ubiquitous to provide any meaningful interpretation of their presence, beyond being indicative of an organic origin. It should be noted, however, that highly polar material could have been present, which would not have successfully eluted from the column, or indeed volatilised sufficiently in the probe.

18. Quanterness passage tomb, Orkney (NMS unreg): Box 10/Box K/bag 203 (Bag 3) 249-01: M2 right lingual (calculus: 5.38 mg)

The thermal desorption total ion chromatogram (TIC) revealed carbon dioxide as the only detectable component, indicating the essential absence of free, thermally extractable organic components in this sample. It should be noted, however, that highly polar material could have been present, which would not have successfully eluted from the column, or indeed volatilised sufficiently in the probe. It is not possible to determine the significance of the carbon dioxide, beyond it being indicative of oxidised organic material.

In contrast to the thermal desorption profile, the pyrolysis-GC-MS TIC (Fig. 15) is dominated by a complex suite of organic compounds. These include a series of C_7 to C_{18} *n*-1-alkenes and *n*-alkanes, typical of unsaturated and saturated fat/oil-derived acyl lipids[5,6]. The absence of these compounds in the TD profile provides molecular evidence for a polymeric/bound acyl lipid source. However, in addition to *n*-1-alkenes and *n*-alkanes, typical of fat/oil derived lipids, the pyrogram also displayed a series of C_9 to C_{16} iso-methyl-1-alkenes (isoalkenes)

and 2-methylalkanes (isoalkanes), presumably deriving from the branched fatty acids which are significant components of bacterial triglycerides.

There were components identified in the pyrolysate TIC which can be indicative of 'black carbon', i.e. charcoal or soot[7-10]. However, the dominance of toluene, rather than the normally dominant aromatic hydrocarbon benzene, is notably atypical of chars[7-10]. The markers identified here can also be indicative of proteins[6,11] and combined with the abundant related protein markers pyrrole and benzenepropanenitrile[6,9] identified this is likely to be the main origin of these compounds in this sample, pointing to an appreciable protein-derived component[6,9]. The proteinaceous origin for the aromatic markers is further corroborated by the moderate abundance of 4-methylphenol (*p*-cresol), which can be indicative of the amino acid tyrosine[12-14]. However, the presence of bacterial markers means the possibility that at least a partial origin could be microbial cannot be excluded.

However, highly notable in this context is the additional presence of notably significant amounts of the more unusual alkyl pyrroles. C_1 alkyl pyrroles: 2- and 3-methylpyrroles; C_2 alkyl pyrroles: 2- and 3-ethylpyrroles, 2,4-, 2,3- and 3,4-dimethyl-pyrroles; C_3 alkyl pyrroles: 2-ethyl-4(?)-methylpyrrole, 2-ethyl-3(?)-methylpyrrole, 4-ethyl-2-methylpyrrole, 2,3,5-trimethylpyrrole; C_4 alkyl pyrroles: an unidentified propylmethylpyrrole, 2,5(?)-diethylpyrrole, 4-ethyl-2,3-dimethylpyrrole, 3-ethyl-2,4-dimethylpyrrole and 2,3,4,5-tetramethylpyrrole; two C_5 alkyl pyrroles: 3-methyl-4-isobutylpyrrole, 2,3(?)-diethyl-5?-methylpyrrole. Collectively, these specific C_1, C_2, C_3, C_4 and C_5 alkyl pyrroles are characteristic of a significant tetrapyrrole/porphyrin-derived origin and are indicative of an algal source observed in fossils and expected in similarly diagenetically altered archaeological material[11,21,22]. The presence here of the abundant protein markers and specifically the C_1 to C_5 alkyl pyrroles, combined with the particular lipid profile and specific carbohydrate markers (see below) identified, indicate the presence of a macroalgae, i.e. seaweed, in this sample[21,22,24] (see discussion below).

The thermally-derived carbohydrate markers 2-methyl-2-cyclopenten-1-one and 2,3-dimethyl-2-cyclopenten-1one were identified as moderately abundant components. That they were absent in the TD profile points to their originating from the original polymeric carbohydrate source (e.g. starch)[25]. Although levoglucosan (1,6-anhydro-β-D-glucopyranose) is usually the major component in the thermally-derived products of carbohydrates analysed by TD/Py-GC-MS, its absence from the TD profile here is consistent with the removal of

hydroxyl groups in the original carbohydrate polymer, so preventing its formation in the probe. The additional absence of the normally significant carbohydrate markers 2-hydroxy-3-methyl-2-cyclopenten-1-one and 3-hydroxy-2-methyl-2-cyclopenten-1-one observed in pure standard material (glycogen, starch and cellulose) would also support this interpretation. This shows a strong matrix effect likely to be due to the inorganic component of the calculus and the microflora in the mouth. This dehydration/dehydroxylation has also been observed in dog calculus and 19th century human calculus[6], where other markers, e.g. a prevalence of nitriles, with amides very minor, deriving from dehydrating conditions in the mouth of dogs and humans (though not sheep[6]) supporting this view[6]. The presence of carbon dioxide is also consistent with a lipid, amino acid/protein and carbohydrate component in this sample. Notably, these findings did not reveal key biomarkers typically observed in soils[20,23]. It should also be noted that highly polar material could have been present, which would not have successfully eluted from the column, or indeed volatilised sufficiently in the probe.

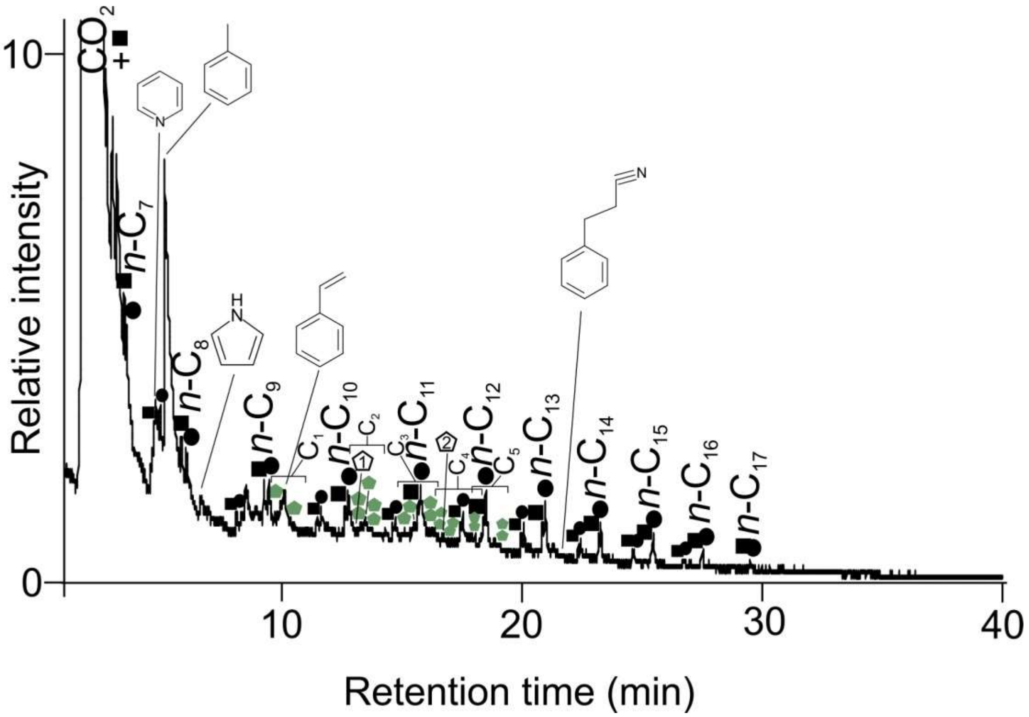

Supplementary Figure 15. Reconstructed total ion chromatogram of the pyrogram (pyrolysis profile) (610 °C for 10s) of sample 249-01, Quanterness, after thermal desorption (310 °C for 10 s). Key to Fig. 15: Peak identities (x indicates carbon chain length): filled

squares, Cx indicates alkenes; filled circles, Cx indicates alkanes; green filled pentagons indicates alkyl pyrroles (with alkyl chain length above, Cx); open pentagons with numerals inside indicates carbohydrate pyrolysis markers, 1 is 2-methyl-2-cyclopenten-1-one and 2 is 2,3-dimethyl-2-cyclopentene-1-one. Also shown are the structures of five aromatic compounds identified: pyridine, pyrrole, toluene, styrene, and benzenepropanenitrile. CO_2 indicates carbon dioxide.

19. Quanterness passage tomb, Orkney (NMS unreg): Box 20 (Box A Bag 666 Bag 1 or 4) 2730-2734 (mandible split): M1 left lingual (calculus: 7.62 mg)

The thermal desorption total ion chromatogram (TIC) revealed carbon dioxide as the only detectable (trace) component, indicating the essential absence of free, thermally extractable organic components in this sample. It should be noted, however, that highly polar material could have been present, which would not have successfully eluted from the column, or indeed volatilised sufficiently in the probe. It is not possible to determine the significance of the carbon dioxide, beyond it being indicative of oxidised organic material.

The pyrolysate TIC revealed carbon dioxide, C_4 and C_5 1-alkenes, cyclopentadiene and benzene as the only detectable components. The dominance of carbon dioxide, combined with the relatively minor abundance of the few small organic compounds present, suggests a highly oxidised sample. It is not possible to determine the significance of the carbon dioxide, beyond it being indicative of oxidised organic material and the other compounds are too low an abundance and too ubiquitous to provide any meaningful interpretation of their presence, beyond being indicative of an organic origin. It should be noted, however, that highly polar material could have been present, which would not have successfully eluted from the column, or indeed volatilised sufficiently in the probe.

20. Quanterness passage tomb, Orkney (NMS unreg): Box 20/Bag 675 (Box A: Bag 2) 2552-02: M2 right lingual (calculus: 9.31 mg)

The thermal desorption total ion chromatogram (TIC) revealed carbon dioxide as the only detectable component, indicating the essential absence of free, thermally extractable organic components in this sample. It should be noted, however, that highly polar material could have been present, which would not have successfully eluted from the column, or indeed

volatilised sufficiently in the probe. It is not possible to determine the significance of the carbon dioxide, beyond it being indicative of oxidised organic material.

In contrast to the thermal desorption profile, the pyrolysis-GC-MS TIC (Fig. 16) is dominated by a complex suite of organic compounds. These include a series of C_7 to C_{18} *n*-1-alkenes and *n*-alkanes, typical of unsaturated and saturated fat/oil-derived acyl lipids[5,6]. A distribution with maxima at C_{10}/C_{11} and C_{14} suggests the original acyl lipids may have been high in polyunsaturated fatty acids. The absence of these compounds in the TD profile provides molecular evidence for a polymeric/bound acyl lipid source. However, in addition to *n*-1-alkenes and *n*-alkanes, typical of fat/oil derived lipids, the pyrogram also displayed a series of C_9 to C_{17} iso-methyl-1-alkenes (isoalkenes) and 2-methylalkanes (isoalkanes), presumably deriving from the branched fatty acids which are significant components of bacterial triglycerides.

There were components identified in the pyrolysate TIC which can be indicative of 'black carbon', i.e. charcoal or soot[7,10]. However, the dominance of toluene, rather than the normally dominant aromatic hydrocarbon benzene, is notably atypical of chars[7-10]. The markers identified here can also be indicative of proteins[6,11] and combined with the abundant related protein markers pyrrole, benzenepropanenitrile and indole[6,11] identified this is likely to be the main origin of these compounds in this sample, pointing to a substantial protein-derived component[6,11]. This is also corroborated by the presence of aliphatic nitriles (carbon numbers $C_{14:0}$, , $C_{15:0}$, $C_{16:0}$ (max), $C_{17:0}$ and $C_{18:0}$) suggesting a lipid (fat/oil) in this sample which has combined with protein-derived nitrogen to produce these moieties[8]. The proteinaceous origin for the aromatic markers is further corroborated by the moderate abundance of 4-methylphenol (*p*-cresol), which can be indicative of the amino acid tyrosine[12-14], and the moderately abundant constituent indole which is consistent with a tryptophan amino acid source[12-14,19]. However, the presence of bacterial markers means the possibility that at least a partial origin could be microbial cannot be excluded.

However, highly notable in this context is the additional presence of notably significant amounts of the more unusual alkyl pyrroles. C_1 alkyl pyrroles: 2- and 3-methylpyrroles; C_2 alkyl pyrroles: 2- and 3-ethylpyrroles, 2,4-, 2,3- and 3,4-dimethyl-pyrroles; C_3 alkyl pyrroles: 2-ethyl-4(?)-methylpyrrole, 2-ethyl-3(?)-methylpyrrole, 4-ethyl-2-methylpyrrole, 2,3,5-trimethylpyrrole; C_4 alkyl pyrroles: two unidentified propylmethylpyrroles, four unidentified ethyldimethylpyrroles, 2,5(?)-diethylpyrrole, 2,4(?)-diethylpyrrole, 2,3(?)-diethylpyrrole,

3,4(?)-diethylpyrrole 4-ethyl-2,3-dimethylpyrrole, 3-ethyl-2,4-dimethylpyrrole and 2,3,4,5-tetramethylpyrrole; two C_5 alkyl pyrroles: 3-methyl-4-isobutylpyrrole and 2,3(?)-diethyl-5?-methylpyrrole; a C_6 alkyl pyrrole: 3-ethyl-4-isobutylpyrrole. Collectively, these specific C_1, C_2, C_3, C_4, C_5 and C_6 alkyl pyrroles are characteristic of a significant tetrapyrrole/porphyrin-derived origin and are indicative of an algal source observed in fossils and expected in similarly diagenetically altered archaeological material[11,21,22]. The presence here of the abundant protein markers and specifically the C_1 to C_6 alkyl pyrroles, combined with the particular lipid profile and specific carbohydrate markers identified, indicate the presence of a macroalgae, i.e. seaweed, in this sample[21,22,24] (see discussion).

The thermally-derived carbohydrate markers 2-methyl-2-cyclopenten-1-one and 2,3-dimethyl-2-cyclopenten-1one were identified as moderately abundant components. That they were absent in the TD profile points to their originating from the original polymeric carbohydrate source (e.g. starch)[25]. Although levoglucosan (1,6-anhydro-β-D-glucopyranose) is usually the major component in the thermally-derived products of carbohydrates analysed by TD/Py-GC-MS, its absence from the TD profile here is consistent with the removal of hydroxyl groups in the original carbohydrate polymer, so preventing its formation in the probe. The additional absence of the normally significant carbohydrate markers 2-hydroxy-3-methyl-2-cyclopenten-1-one and 3-hydroxy-2-methyl-2-cyclopenten-1-one observed in pure standard material (glycogen, starch and cellulose) would also support this interpretation. This shows a strong matrix effect likely to be due to the inorganic component of the calculus and the microflora in the mouth. This dehydration/dehydroxylation has also been observed in dog calculus and 19th century human calculus[6], where other markers, e.g. a prevalence of nitriles, with amides very minor, deriving from dehydrating conditions in the mouth of dogs and humans (though not sheep[6]) supporting this view[6]. The presence of carbon dioxide is also consistent with a lipid, amino acid/protein and carbohydrate component in this sample. Notably, these findings did not reveal key biomarkers typically observed in soils[20,23]. It should also be noted that highly polar material could have been present, which would not have successfully eluted from the column, or indeed volatilised sufficiently in the probe.

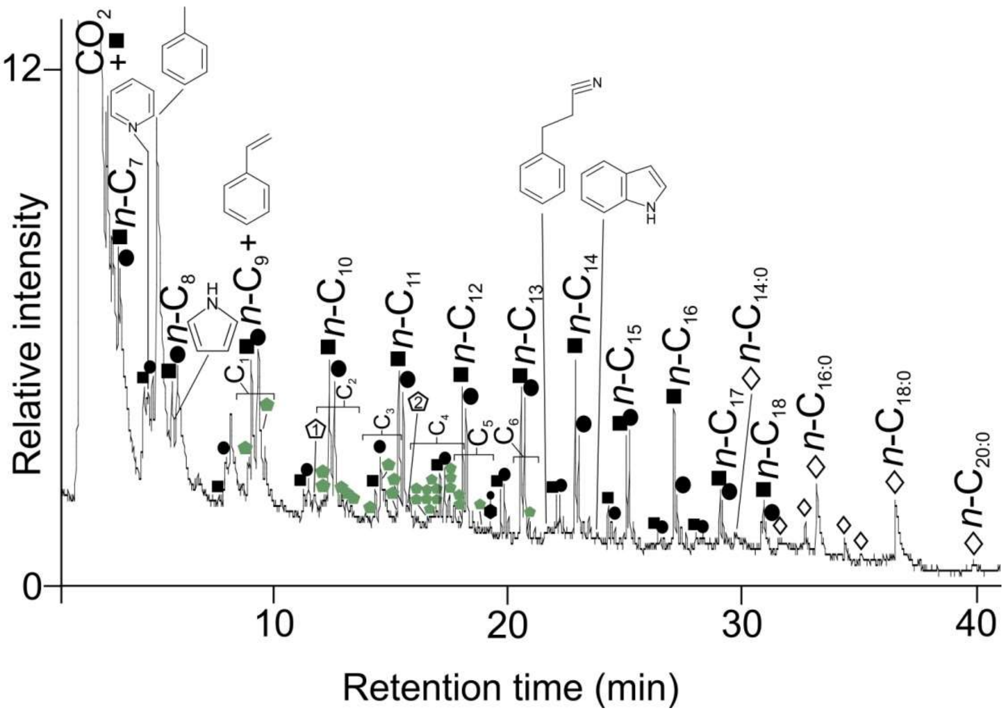

Supplementary Figure 16. Reconstructed total ion chromatogram of the pyrogram (pyrolysis profile) (610 °C for 10s) of sample 2552-02, Quanterness, after thermal desorption (310 °C for 10 s). Key to Fig. 16: Peak identities (x indicates carbon chain length): filled squares, Cx indicates alkenes; filled circles, Cx indicates alkanes; open diamonds, Cx:y indicates acyclic nitriles; green filled pentagons indicates alkyl pyrroles (with alkyl chain length above, Cx); filled hexagon with filled circle attached indicates alkyl phenol; open pentagons with numerals inside indicates carbohydrate pyrolysis markers, 1 is 2-methyl-2-cyclopenten-1-one and 2 is 2,3-dimethyl-2-cyclopentene-1-one. Also shown are the structures of four aromatic compounds identified: pyridine, pyrrole, toluene, styrene, benzenepropanenitrile and indole. CO_2 indicates carbon dioxide.

21. Quanterness passage tomb, Orkney (NMS unreg): Box 19/Box F/Bag 663 from skull 1408: Loose maxillary molar Lingual (calculus: 0.80 mg)

The thermal desorption total ion chromatogram (TIC) revealed carbon dioxide and 1-butene as the only detectable components, indicating the essential absence of free, thermally extractable organic components in this sample. It should be noted, however, that highly polar material could have been present, which would not have successfully eluted from the column, or indeed volatilised sufficiently in the probe. The 1-butene possibly derives from lipid or

proteinaceous material in the sample[11], and perhaps produced at this relatively low temperature due to matrix effects. It is not possible to determine the significance of the carbon dioxide, beyond it being indicative of oxidised organic material.

The pyrolysate TIC revealed carbon dioxide and the C_4 and C_5 1-alkenes as the only detectable components. The dominance of carbon dioxide suggests a highly oxidised sample. It is not possible to determine the significance of the carbon dioxide, beyond it being indicative of oxidised organic material. It should be noted, however, that highly polar material could have been present, which would not have successfully eluted from the column, or indeed volatilised sufficiently in the probe.

Discussion, Quanterness passage tomb, Orkney (NMS unreg)

The TD-GC-MS analyses revealed very little organic material in all of the six samples. Carbon dioxide was the only compound observed in the thermal desorption profile of all six samples (though even this was very minor in four of the six samples) indicating highly oxidised organic material with an essential absence of free, thermally extractable, organic constituents in these samples.

In contrast to the TD-GC-MS analyses, the Py-GC-MS results revealed appreciable amounts of organic material in all six calculus samples, although only two contained moderate to major abundances of organic compounds $>C_5$. This indicates that although the cool environment on Orkney has allowed the survival of a wide range of biomolecules relating to fats/oil, proteins and carbohydrates (see below), diagenesis and biodegradation have resulted in the loss of most, if not all, free biomarkers. In contrast, bound/polymeric biomacromolecules (some clearly resulting from pre- and/or post-depositional biochemical reactions between the constituents of the original foods consumed) have been appreciably well preserved in two of the samples, albeit the more resilient moieties and compound classes are likely to have been preferentially preserved. These two samples most rich in thermally extractable biomolecules (only) will be discussed below.

The pyrolysis-GC-MS analyses of the two samples containing significant amounts of organic material were dominated by a complex suite of organic compounds indicative of lipid (fat/oil), protein and carbohydrate components. These include a series of C_7 to C_{18} *n*-1-alkenes and *n*-alkanes, typical of unsaturated and saturated fat/oil-derived acyl lipids[5,6]. A bimodal distribution with maxima at C_{10}/C_{11} and C_{14} combined with abundant short chain

(C_4-C_7) *n*-1-alkenes suggests the original acyl lipids in these samples are likely to have been high in polyunsaturated fatty acids. The absence of these compounds in the TD profile provides molecular evidence for a polymeric/bound acyl lipid source. However, in addition to *n*-1-alkenes and *n*-alkanes, typical of fat/oil derived lipids, the pyrograms also displayed a series of C_8 to C_{17} iso-methyl-1-alkenes (isoalkenes) and 2-methylalkanes (isoalkanes), presumably deriving from the branched fatty acids which are significant components of bacterial triglycerides.

There were components in the pyrolysate which can be indicative of ‘black carbon’, i.e. charcoal or soot[7-10]. However, the dominance of toluene, rather than the normally dominant aromatic hydrocarbon benzene, is notably atypical of chars[7-10]. The markers identified here can also be indicative of proteins[6,11] and combined with the related protein markers pyrrole and benzenepropanenitrile[6,11] identified this is most likely to be the main origin of these compounds in this sample, pointing to an appreciable protein-derived component[6,11]. This is also corroborated by the presence of aliphatic nitriles (carbon numbers $C_{14:0}$, $C_{15:0}$, $C_{16:0}$ (max), $C_{17:0}$ and $C_{18:0}$) suggesting a lipid (fat/oil) in this sample which has combined with protein-derived nitrogen to produce these moieties[5], although the presence of bacterial markers means the possibility that at least a partial origin could be microbial cannot be excluded.

Far more diagnostic of a particular dietary source are the particular suite of alkyl pyrroles observed in the pyrograms of these samples, with an alkyl chain length of up to C_6. Both these samples revealed significant amounts of these biomarkers. This included the 2- and 3-dimethylpyrroles, 2- and 3-ethylpyrroles, 2,4-, 2,3- and 3,4-dimethylpyrroles, three ethylmethylpyrroles, a trimethylpyrrole, two propylmethylpyrroles, five ethyldimethylpyrroles, three diethylpyrroles, 2,3,4,5-tetramethylpyrrole, 3-methyl-4-isobutylpyrrole, a diethylmethylpyrrole and 3-ethyl-4-isobutylpyrrole (trace). These specific C_1 to C_6 alkyl pyrroles are highly diagnostic of the presence of a significant tetrapyrrole component. Their porphyrin-derived origin, and the relative abundance of some of the key biomarkers, is indicative of an algal source observed in fossils and expected in similarly diagenetically altered archaeological material[11,21,22]. Although C_1 and C_2 alkyl pyrroles can also derive from protein-derived amino acids such as glycine, proline, hydroxyproline, serine and glutamic acid[12-14,20,40,41], the C_3+ alkyl pyrroles identified in these six samples point to a tetrapyrrole source[21,22,40,41]. Moreover, the same specific C_1 to C_5 alkylpyrroles identified in the Py-GC-MS of an algal kerogen[21,22] were observed in these samples. More specifically, the

highly unusual alkyl pyrroles, 3-methyl-4-isobutylpyrrole and 3-ethyl-4-isobutylpyrrole are known to derive from the C_8 side chain of bacteriochlorophylls c and d[47] and are biomarkers for the relatively rare bacteriochlorophylls c and d which are found in green bacteria such as *Chloroflexus* sp.[47] and are known to naturally occur in association with intertidal red seaweeds such as *Porphyra umbilicalis*[47-49]. Overall the two samples display a remarkably similar pyrolysis profile to algal-derived kerogen previously studied via very similar organic geochemical techniques[21,22].

As discussed above, the bimodal distribution of *n*-1-alkenes/*n*-alkanes with maxima at C_{10}/C_{11} and C_{14}, combined with abundant short chain (C_4-C_7) *n*-1-alkenes, suggests the original acyl lipids are likely to have been high in polyunsaturated fatty acids, which is consistent with a marine source. Moreover, the presence here of the specific C_1 to C_6 alkyl pyrroles, combined with the particular lipid profile identified, indicate the presence of a macroalgae, i.e. seaweed, in these samples[21,22,24]. Seaweed is known to have been exploited for food in antiquity[56], although its consumption by humans in Orkney seems only to have been tentatively considered to date[2,57].

Distillery Cave

Distillery Cave was accidentally discovered in 1890[82]. The archaeological levels were disturbed, and the site was eventually destroyed. However, the remains of four adults and four children were discovered. Three of those individuals have produced radiocarbon dates ranging from 3762–3644 cal BC to 3514–3353 cal BC[83]. The Cave lay at around 12m above modern sea level.

22. Distillery Cave, Oban, Highland (NMS unreg) Box 6, adult male '1': Canine right lingual (calculus: 5.62 mg)[28]

The thermal desorption total ion chromatogram (TIC) revealed carbon dioxide and 1-butene as the only detectable components, indicating the essential absence of free, thermally extractable organic components in this sample. It should be noted, however, that highly polar material could have been present, which would not have successfully eluted from the column, or indeed volatilised sufficiently in the probe. The 1-butene possibly derives from lipid or proteinaceous material in the sample[11], and perhaps produced at this relatively low

temperature due to matrix effects. It is not possible to determine the significance of the carbon dioxide, beyond it being indicative of oxidised organic material.

In contrast to the thermal desorption profile, the pyrolysis-GC-MS TIC (Fig. 17) is dominated by a complex suite of organic compounds. These include a series of C_7 to C_{17} *n*-1-alkenes and *n*-alkanes, typical of unsaturated and saturated fat/oil-derived acyl lipids[5,6]. A distribution with maxima at C_{10}/C_{11} and C_{14} suggests the original acyl lipids may have been high in polyunsaturated fatty acids. The absence of these compounds in the TD profile provides molecular evidence for a polymeric/bound acyl lipid source. However, in addition to *n*-1-alkenes and *n*-alkanes, typical of fat/oil derived lipids, the pyrogram also displayed a series of C_9 to C_{16} iso-methyl-1-alkenes (isoalkenes) and 2-methylalkanes (isoalkanes), presumably deriving from the branched fatty acids which are significant components of bacterial triglycerides.

There were components identified in the pyrolysate TIC which can be indicative of 'black carbon', i.e. charcoal or soot[7-10]. However, the dominance of toluene, rather than the normally dominant aromatic hydrocarbon benzene, is notably atypical of chars[7-10]. The markers identified here can also be indicative of proteins[6,11] and combined with the abundant related protein markers pyrrole, benzenepropanenitrile and indoles[6,11] identified this is likely to be the main origin of these compounds in this sample, pointing to a substantial protein-derived component[6,11]. This is also corroborated by the presence of aliphatic nitriles (carbon numbers $C_{14:0}$, $C_{16:0}$ (max) and $C_{18:0}$) suggesting a lipid (fat/oil) in this sample which has combined with protein-derived nitrogen to produce these moieties[5]. In addition, the moderately abundant constituents indole and 3-methylindole (skatole) were also identified, which are consistent with a tryptophan amino acid source[12,13,19,20], although the presence of bacterial markers means the possibility that at least a partial origin could be microbial cannot be excluded.

However, highly notable in this context is the additional presence of notably significant amounts of the more unusual alkyl pyrroles. C_1 alkyl pyrroles: 2- and 3-methylpyrroles; C_2 alkyl pyrroles: 2- and 3-ethylpyrroles, 2,4-, and 2,3-dimethyl-pyrroles; C_3 alkyl pyrroles: 2-ethyl-4(?)-methylpyrrole, 2-ethyl-3(?)-methylpyrrole and 4-ethyl-2-methylpyrrole, 2,3,5-trimethylpyrrole; C_4 alkyl pyrroles: two unidentified propylmethylpyrroles, three unidentified ethyldimethylpyrroles, 2,5(?)-diethylpyrrole, 2,3(?)-diethylpyrrole, 3,4(?)-diethylpyrrole, 4-ethyl-2,3-dimethylpyrrole, 3-ethyl-2,4-dimethylpyrrole and 2,3,4,5-tetramethylpyrrole; C_5

alkyl pyrroles: 3-methyl-4-isobutylpyrrole, 3-ethyl-2,4,5-trimethylpyrrole and an unidentified diethylmethylpyrrole; C_6 alkyl pyrroles: 3-ethyl-4-isobutylpyrrole (trace). Collectively, these specific C_1, C_2, C_3, C_4, C_5 and C_6 alkyl pyrroles are characteristic of a significant tetrapyrrole/porphyrin-derived origin and are indicative of an algal source observed in fossils and expected in similarly diagenetically altered archaeological material[11,21,22]. The presence here of the abundant protein markers and specifically the C_1 to C_6 alkyl pyrroles, combined with the particular lipid profile (see below) identified, indicate the presence of a macroalgae, i.e. seaweed, in this sample[21,22,24] (see discussion below). The presence of carbon dioxide is also consistent with a lipid and amino acid/protein component in this sample. Notably, these findings did not reveal key biomarkers typically observed in soils[20,23]. It should also be noted that highly polar material could have been present, which would not have successfully eluted from the column, or indeed volatilised sufficiently in the probe.

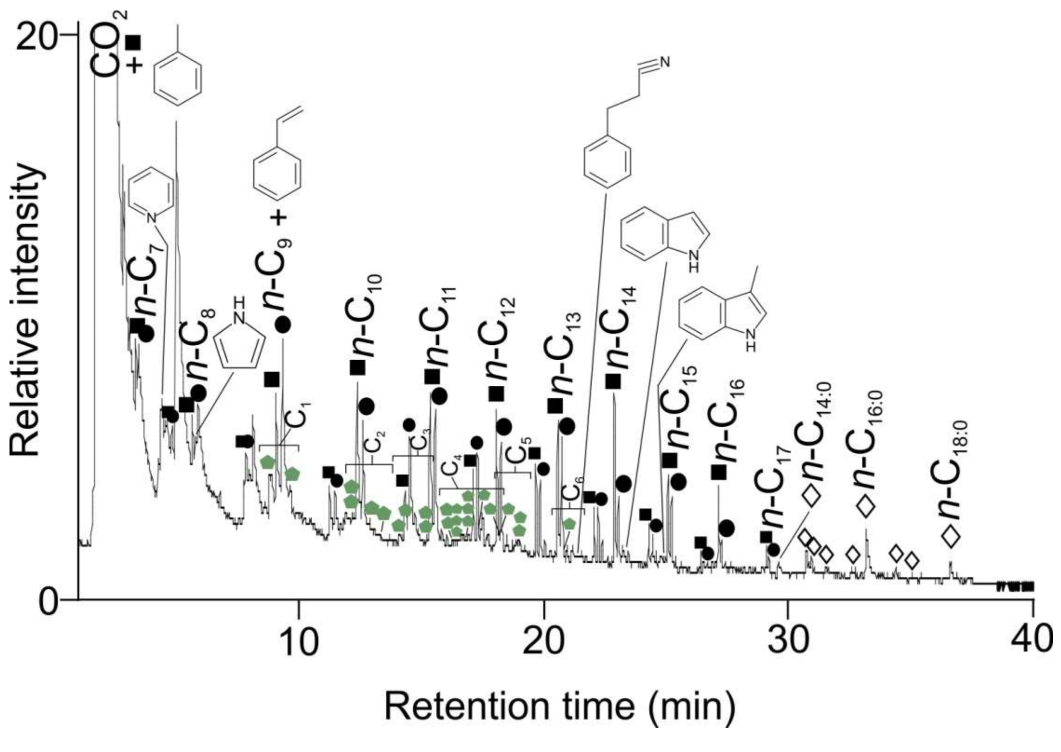

Supplementary Figure 17. Reconstructed total ion chromatogram of the pyrogram (pyrolysis profile) (610 °C for 10s) of sample adult male '1', Distillery Cave, after thermal desorption (310 °C for 10 s). Key to Fig. 17: Peak identities (x indicates carbon chain length): filled squares, Cx indicates alkenes; filled circles, Cx indicates alkanes; open diamonds, Cx:y indicates acyclic nitriles; green filled pentagons indicates alkyl pyrroles (with alkyl chain

length above, Cx). Also shown are the structures of seven aromatic compounds identified: pyridine, pyrrole, toluene, styrene, benzenepropanenitrile, indole and 3-methylindole (skatole). CO_2 indicates carbon dioxide.

23. Distillery Cave, Oban, Highland (NMS unreg) Box 6, prob male '3': PM2 right labial (calculus: 4.58 mg)[28]

The thermal desorption total ion chromatogram (TIC) revealed carbon dioxide as the only detectable component, indicating the essential absence of free, thermally extractable organic components in this sample. It should be noted, however, that highly polar material could have been present, which would not have successfully eluted from the column, or indeed volatilised sufficiently in the probe. It is not possible to determine the significance of the carbon dioxide, beyond it being indicative of oxidised organic material.

In contrast to the thermal desorption profile, the pyrolysis-GC-MS TIC (Fig. 18) is dominated by a complex suite of organic compounds. These include a series of C_7 to C_{17} *n*-1-alkenes and *n*-alkanes, typical of unsaturated and saturated fat/oil-derived acyl lipids[5,6]. The absence of these compounds in the TD profile provides molecular evidence for a polymeric/bound acyl lipid source. However, in addition to *n*-1-alkenes and *n*-alkanes, typical of fat/oil derived lipids, the pyrogram also displayed a series of C_8 to C_{16} iso-methyl-1-alkenes (isoalkenes) and 2-methylalkanes (isoalkanes), presumably deriving from the branched fatty acids which are significant components of bacterial triglycerides.

The components identified in the pyrolysate TIC of the previous sample from the same site and which can be indicative of 'black carbon' (i.e. charcoal or soot) and/or proteins were not detected, although this may reflect the lower abundance of the organic compounds in the pyrogram if this sample. Corroborating this is the presence of aliphatic nitriles (carbon numbers $C_{14:0}$, i-$C_{15:0}$ (max) and $C_{16:0}$) suggesting a lipid (fat/oil) in this sample which has combined with protein-derived nitrogen to produce these moieties[5], although the dominance of the i-$C_{15:0}$ bacterial marker means at least a partial microbial origin for them is likely.

However, highly notable in this context is the additional presence of notably significant amounts of the more unusual alkyl pyrroles. C_1 alkyl pyrroles: 2- and 3-methylpyrroles; C_2 alkyl pyrroles: 2- and 3-ethylpyrroles, 2,4-, and 2,3-dimethyl-pyrroles; C_3 alkyl pyrroles: 4-ethyl-2-methylpyrrole and 2,3,5-trimethylpyrrole. Collectively, these specific C_1, C_2 and C_3 alkyl pyrroles are indicative of a significant tetrapyrrole/porphyrin-derived origin and suggest

an algal source observed in fossils and expected in similarly diagenetically altered archaeological material[11,21,22]. The similarity to the previous sample from this site, albeit the biomarkers are in appreciably lower abundance, indicates the presence of a macroalgae, i.e. seaweed, in this sample[21,22,24] (see discussion below). The presence of carbon dioxide is also consistent with a lipid and amino acid/protein component in this sample. Notably, these findings did not reveal key biomarkers typically observed in soils[20,23]. It should also be noted that highly polar material could have been present, which would not have successfully eluted from the column, or indeed volatilised sufficiently in the probe.

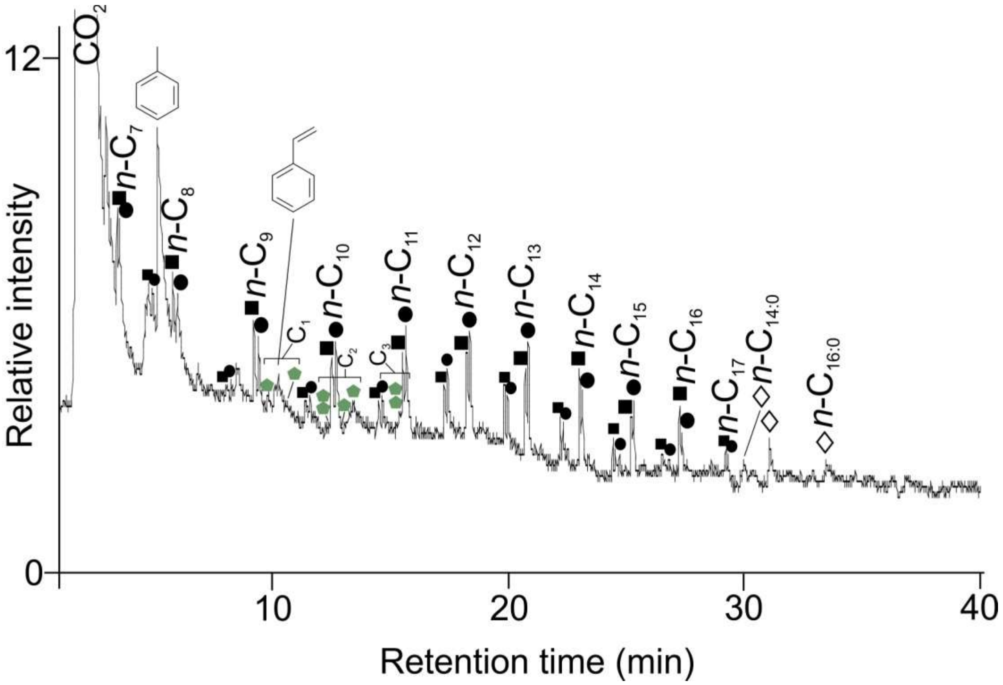

Supplementary Figure 18. Reconstructed total ion chromatogram of the pyrogram (pyrolysis profile) (610 °C for 10s) of sample prob male ‘3’, Distillery Cave, after thermal desorption (310 °C for 10 s). Key to Fig. 18: Peak identities (x indicates carbon chain length): filled squares, Cx indicates alkenes; filled circles, Cx indicates alkanes; open diamonds, Cx:y indicates acyclic nitriles; green filled pentagons indicates alkyl pyrroles (with alkyl chain length above, Cx). Also shown are the structures of two aromatic compounds identified: toluene and styrene. CO_2 indicates carbon dioxide.

Discussion, Distillery Cave, Oban, Highland (NMS unreg)

Two samples of dental calculus from Distillery Cave were analysed by sequential thermal desorption-gas chromatography-mass spectrometry (TD-GC-MS) and pyrolysis-gas chromatography-mass spectrometry (Py-GC-MS). The samples contained both free and bound/polymeric organic material.

Carbon dioxide dominated the thermal desorption profile, in addition to appreciable amounts of C_4 to C_6 1-alkenes, as the only 'free' organic compounds detected, indicating highly oxidised organic material with little free, thermally extractable, organic constituents in these samples.

In contrast to the TD-GC-MS analyses, the Py-GC-MS results revealed appreciable amounts of organic material in both calculus samples. This indicates that although the relatively cool environment has allowed the survival of a wide range of biomolecules relating to fats/oil and proteins, diagenesis and biodegradation have resulted in the loss of most, if not all, free biomarkers. In contrast, bound/polymeric biomacromolecules (some clearly resulting from pre- and/or post-depositional biochemical reactions between the constituents of the original foods consumed) have been appreciably well preserved, albeit the more resilient moieties and compound classes are likely to have been preferentially preserved.

The pyrolysis-GC-MS analyses were dominated by a complex suite of organic compounds indicative of lipid (fat/oil) and protein components. These include a series of C_7 to C_{17} *n*-1-alkenes and *n*-alkanes, typical of unsaturated and saturated fat/oil-derived acyl lipids[5,6]. A bimodal distribution with maxima at C_{10}/C_{11} and C_{14} combined with abundant short chain (C_4-C_7) *n*-1-alkenes suggests the original acyl lipids in these samples are likely to have been high in polyunsaturated fatty acids. The absence of these compounds in the TD profile provides molecular evidence for a polymeric/bound acyl lipid source. However, in addition to *n*-1-alkenes and *n*-alkanes, typical of fat/oil derived lipids, the pyrograms also displayed a series of C_9 to C_{16} iso-methyl-1-alkenes (isoalkenes) and 2-methylalkanes (isoalkanes), presumably deriving from the branched fatty acids which are significant components of bacterial triglycerides.

There were components identified in the pyrolysate TIC which can be indicative of 'black carbon', i.e. charcoal or soot[7-10]. However, the dominance of toluene, rather than the normally dominant aromatic hydrocarbon benzene, is notably atypical of chars[7-10]. The markers identified here can also be indicative of proteins[6,11] and combined with the abundant related

protein markers pyrrole, benzenepropanenitrile and indoles[6,11] identified this is likely to be the main origin of these compounds in this sample, pointing to a substantial protein-derived component[6,11]. This is also corroborated by the presence of aliphatic nitriles (carbon numbers $C_{14:0}$, $C_{16:0}$ (max) and $C_{18:0}$) suggesting a lipid (fat/oil) in this sample which has combined with protein-derived nitrogen to produce these moieties[5]. In addition, the moderately abundant constituents indole and 3-methylindole (skatole) were also identified, which are consistent with a tryptophan amino acid source[12,13,19,20], although the presence of bacterial markers means the possibility that at least a partial origin could be microbial cannot be excluded.

Far more diagnostic of a particular dietary source are the particular suite of alkyl pyrroles observed in the pyrograms of these samples, with an alkyl chain length of up to C_6. Both samples revealed significant amounts of these biomarkers. This included the 2- and 3-dimethylpyrroles, 2- and 3-ethylpyrroles, 2,4- and 2,3- dimethylpyrroles, three ethylmethylpyrroles, a trimethylpyrrole, two propylmethylpyrroles, five ethyldimethylpyrroles, three diethylpyrroles, 2,3,4,5-tetramethylpyrrole, an ethylpropylpyrrole, 3-ethyl-2,4,5-trimethylpyrrole, a diethylmethylpyrrole and an unidentified C_6 ethylpyrrole (trace). These specific C_1 to C_6 alkyl pyrroles are highly diagnostic of the presence of a significant tetrapyrrole component. Their porphyrin-derived origin, and the relative abundance of some of the key biomarkers, is indicative of an algal source observed in fossils and expected in similarly diagenetically altered archaeological material[11,21,22]. Although C_1 and C_2 alkyl pyrroles can also derive from protein-derived amino acids such as glycine, proline, hydroxyproline, serine and glutamic acid[12-14,20,40,41], the C_3+ alkyl pyrroles identified in these six samples point to a tetrapyrrole source[21,22,40,41]. Moreover, the same specific C_1 to C_5 alkylpyrroles identified in the Py-GC-MS of an algal kerogen[21,22] were observed in these samples. More specifically, the highly unusual alkyl pyrroles, 3-methyl-4-isobutylpyrrole and 3-ethyl-4-isobutylpyrrole are known to derive from the C_8 side chain of bacteriochlorophylls c and d[47] and are biomarkers for the relatively rare bacteriochlorophylls c and d which are found in green bacteria such as *Chloroflexus* sp.[47] and are known to naturally occur in association with intertidal red seaweeds such as *Porphyra umbilicalis*[47-49]. Overall the two samples display a remarkably similar pyrolysis profile to algal-derived kerogen previously studied via very similar organic geochemical techniques[21,22].

As discussed above, the bimodal distribution of *n*-1-alkenes/*n*-alkanes with maxima at C_{10}/C_{11} and C_{14}, combined with abundant short chain (C_4-C_7) *n*-1-alkenes, suggests the original acyl lipids are likely to have been high in polyunsaturated fatty acids, which is consistent with a marine source. Moreover, the presence here of the specific C_1 to C_6 alkyl pyrroles, combined with the particular lipid profile identified, indicate the presence of a macroalgae, i.e. seaweed, in these samples[21,22,24]. Seaweed is known to have been exploited for food in antiquity[56], although its consumption by humans in Orkney, for example (see below), seems only to have been tentatively considered to date[2,57].

Raschoille Cave, Oban, Highland

Raschoille Cave was discovered in 1984 and was excavated by the local archaeological society[84]. At least 57 individuals were recovered, several of whom have been radiocarbon dated, with dates ranging from 3942–3653 cal BC to 3487–3034 cal BC[85]. Marine shells, fishbones, and animal bones were also recovered, some of these dating to within the Mesolithic period (6500–6000 BC). Clearly the cave was not used for the burial of human remains until the Early Neolithic period.

24. Raschoille Cave, Oban, Highland (NMS unreg) ORC III 24.1: M1 right lingual (calculus: 2.94 mg) (4000-2900 BC)[28]

The thermal desorption total ion chromatogram (TIC) revealed carbon dioxide and 1-butene as the only detectable components, indicating the essential absence of free, thermally extractable organic components in this sample. It should be noted, however, that highly polar material could have been present, which would not have successfully eluted from the column, or indeed volatilised sufficiently in the probe. The 1-butene possibly derives from lipid or proteinaceous material in the sample[11], and perhaps produced at this relatively low temperature due to matrix effects. It is not possible to determine the significance of the carbon dioxide, beyond it being indicative of oxidised organic material.

The pyrolysate TIC revealed carbon dioxide, C_4 to C_6 1-alkenes, a C_7 diene, acetone, a methylcyclopentadiene, benzene, pyridine and toluene as the only detectable components. The dominance of carbon dioxide, combined with the relatively minor abundance of the few

small organic compounds present, suggests a highly oxidised sample. It is not possible to determine the significance of the carbon dioxide, beyond it being indicative of oxidised organic material and the other compounds are too low an abundance and too ubiquitous to provide any meaningful interpretation of their presence, beyond them being indicative of an organic origin. It should be noted, however, that highly polar material could have been present, which would not have successfully eluted from the column, or indeed volatilised sufficiently in the probe.

Discussion, Raschoille Cave, Oban, Highland (NMS unreg)

One sample of dental calculus from Rascholle Cave was analysed by sequential thermal desorption-gas chromatography-mass spectrometry (TD-GC-MS) and pyrolysis-gas chromatography-mass spectrometry (Py-GC-MS). The sample contained little free and bound/polymeric organic material, most likely reflecting unfavourable prevailing conditions for biomolecular preservation. Of those organic compounds identified, the dominance of carbon dioxide and the relatively minor abundance of only a few small organic compounds suggests that only highly oxidised organic material was present.

Boatbridge Quarry cist 2, Thankerton, S Lanarkshire

The human remains from Boatbridge cist 2 are those of an unusually tall adolescent male, radiocarbon dated to 2397–2138 cal BC and 2481–2040 cal BC[1,86,87] The cist is one of two found in a sand and gravel quarry above the River Clyde[88].

25. Boatbridge Quarry cist 2, Thankerton, S Lanarkshire (associated with Beaker NMS X.EG 106): PM2 left labial S1 (calculus: 2.60 mg)

The four main compounds in the thermal desorption total ion chromatogram (TIC) (Fig. 19) are carbon dioxide, sulphur dioxide, benzene and acetic acid. In moderate abundance is an unresolved complex mixture (UCM; apparent as a ‘hump’ in the chromatogram) of alkenes and alkanes, carbon numbers C_{16} to C_{24}, within which there is series of C_{16} to C_{24} *n*-alkanes (odd over even predominance) dominating this UCM profile and maximising at *n*-C_{21}.

Branched alkanes, primarily *iso*- and *anteiso*- with carbon numbers C_{17} to C_{24} are also present as minor constituents and are possibly indicative of microbially-derived biodegradation (but see summary below). These carbon numbers present in the TD chromatogram may suggest a submerged aquatic plant source (macrophyte)[89-91], possibly deriving from plant waxes in the original food consumed or reflecting prevailing environmental factors (but, again, see summary below). Naphthalene was also detected, albeit as a minor constituent. No characteristic protein or carbohydrate markers were observed meaning the significance of the non-diagnostic compounds carbon dioxide, benzene and acetic acid is uncertain. It should also be noted that highly polar material could have been present, which would not have successfully eluted from the column, or indeed volatilised sufficiently in the probe.

In contrast to the thermal desorption profile, the pyrolysis-GC-MS TIC (Fig. 19 inset) revealed a complex suite of organic compounds. These include a series of C_{10} to C_{16}*n*-1-alkenes and *n*-alkanes, typical of unsaturated and saturated fat/oil-derived acyl lipids[5,6]. The absence of these compounds in the TD profile provides molecular evidence for a polymeric/bound acyl lipid source. Importantly, there were no bacterially-derived hydrocarbons deriving from the original branched fatty acid acyl groups which are significant components of bacterial triglycerides, indicating this lipid component is not bacterial in origin. The pyrolysate TIC identified components indicative of 'black carbon', i.e. charcoal or soot[7-10]. Benzene and toluene were major compounds present in the pyrolysate (~15% and 30% of total quantified pyrogram peak area respectively), along with moderate quantities of ethyl benzene and o-, m- and p-xylenes (~5% total). The relative dominance of toluene, rather than the normally dominant aromatic hydrocarbon benzene, is somewhat atypical of chars[7-10]. However, the presence of significant amounts of naphthalene, methyl naphthalenes and methyl biphenyl, together with the polynuclear aromatic hydrocarbon combustion markers 1-methyl-9H-fluorene, phenanthrene, 3,6-dimethylphenathrene, 1-methylanthracene, pyrene and 1-methylpyrene, confirm a 'char' component providing molecular evidence for exposure to fire/cooking.The lack of the attendant aromatic nitrogen-containing biomarkers pyrrole, benzonitrile, benzyl nitrile (phenylacetonitrile), benzenepropanenitrile and indoles indicative of proteinaceous material[6,11] suggests a collective origin as combustion compounds ('char') rather than a significant protein component. The presence of carbon dioxide is also consistent with a lipid component in this sample. It should also be noted that highly polar material could have been present, which would not have successfully eluted from the column, or indeed volatilised sufficiently in the probe.

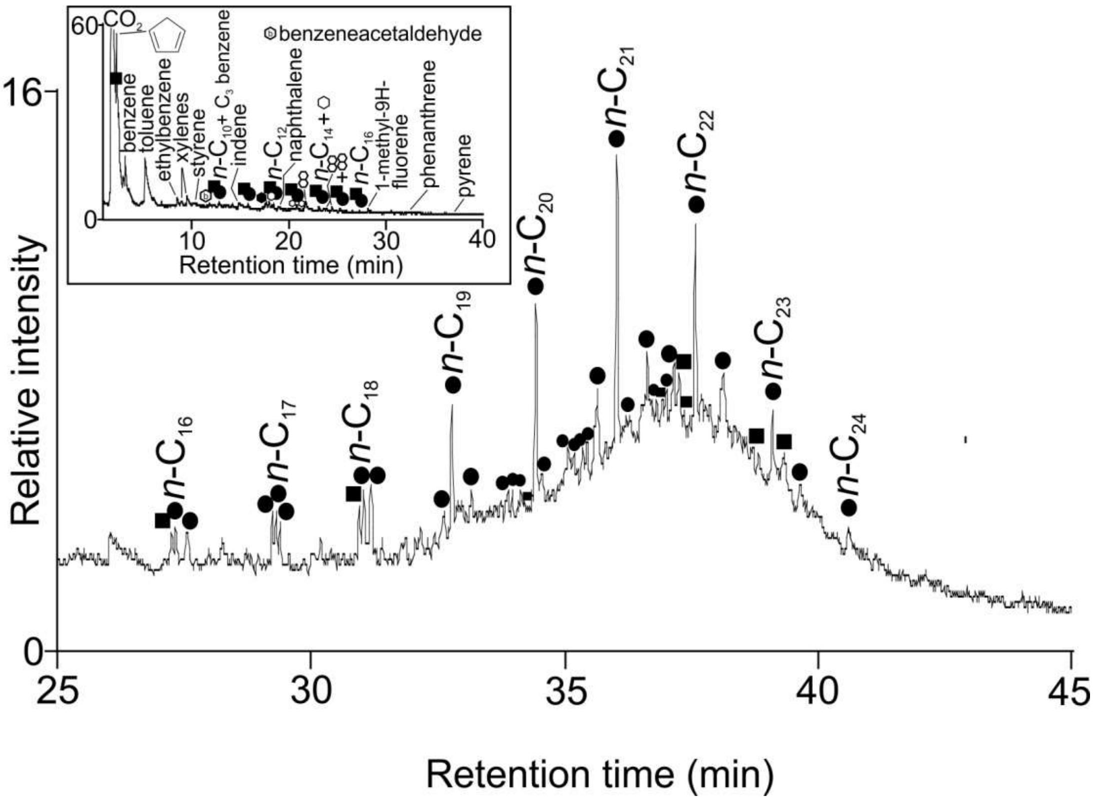

Supplementary Figure 19. Reconstructed total ion chromatogram of the thermal desorption profile (310 °C for 10 s) of sample X.EG 106, Boatbridge Quarry cist. Key to Fig. 19: Peak identities (x indicates carbon chain length): filled squares indicates alkenes; filled circles, Cx indicates alkanes. Inset displays a reconstructed total ion chromatogram of the pyrogram (pyrolysis profile) (610 °C for 10) of this sample, after thermal desorption (310 °C for 10 s). Peak identities: (x indicates carbon chain length): filled squares, Cx indicates alkenes; filled circles, Cx indicates alkanes; CO_2 indicates carbon dioxide. Also shown are benzene, toluene, ethyl benzene, xylenes (*o*-, *m*- and *p*-), styrene, benzeneacetaldehyde, an alkyl benzene, indene, naphthalene, 1-methyl-9H-fluorene, phenanthrene and pyrene.

26. Boatbridge Quarry cist 2, Thankerton, S Lanarkshire (associated with Beaker NMS X.EG 106): M2 right labial S2 (calculus: 1.33 mg)[1,86]

The four main compounds in the thermal desorption total ion chromatogram (TIC) are carbon dioxide, sulphur dioxide, benzene and acetic acid. In minor abundance are a series of alkanes, carbon numbers C_{18} to C_{22}, maximising at n-$C_{20/21}$. Branched alkanes are also present and are

possibly indicative of microbially-derived biodegradation given the similarity to the hydrocarbon profile observed in the previous Boatbridge sample (but see summary below). The carbon numbers present in the TD chromatogram, when taken together with the hydrocarbons observed in Boatbridge S1, may suggest a submerged aquatic plant source (macrophyte)[89-91], possibly deriving from plant waxes in the original food consumed or reflecting prevailing environmental factors (but, again, see summary below). No characteristic protein or carbohydrate markers were observed meaning the significance of the non-diagnostic compounds carbon dioxide, benzene and acetic acid is uncertain. It should also be note that highly polar material could have been present, which would not have successfully eluted from the column, or indeed volatilised sufficiently in the probe.

In contrast to the thermal desorption profile, the pyrolysis-GC-MS TIC revealed a complex suite of organic compounds. These include a series of C_{10} to C_{16} *n*-1-alkenes and *n*-alkanes, typical of unsaturated and saturated fat/oil-derived acyl lipids[5,6]. The absence of these compounds in the TD profile provides molecular evidence for a polymeric/bound acyl lipid source. Importantly, there were no bacterially-derived hydrocarbons deriving from the original branched fatty acid acyl groups which are significant components of bacterial triglycerides, indicating this lipid component is not bacterial in origin. The pyrolysate TIC identified components indicative of 'black carbon', i.e. charcoal or soot[7-10]. Benzene and toluene were major compounds present in the pyrolysate (~15% and 30% of total quantified pyrogram peak area respectively), along with moderate quantities of ethyl benzene and o-, m- and p-xylenes (~5% total). The relative dominance of toluene, rather than the normally dominant aromatic hydrocarbon benzene, is somewhat atypical of chars[7-10]. However, the presence of significant amounts of naphthalene, methyl naphthalenes and a methylbiphenyl, together with the polynuclear aromatic hydrocarbon combustion markers 1-methyl-9H-fluorene and phenanthrene, confirm a 'char' component providing molecular evidence for exposure to fire/cooking. The lack of the attendant aromatic nitrogen-containing biomarkers pyrrole, benzonitrile, benzyl nitrile (phenylacetonitrile), benzenepropanenitrile and indoles indicative of proteinaceous material[6,11] suggests a collective origin as combustion compounds ('char') rather than a significant protein component. The presence of carbon dioxide is also consistent with a lipid component in this sample. It should also be noted that highly polar material could have been present, which would not have successfully eluted from the column, or indeed volatilised sufficiently in the probe.

Discussion, Boatbridge Quarry, Thankerton

Two samples of dental calculus from Boatbridge Quarry, Thankerton, were analysed by sequential thermal desorption-gas chromatography-mass spectrometry (TD-GC-MS) and pyrolysis-gas chromatography-mass spectrometry (Py-GC-MS). The samples contained both free and bound/polymeric organic material.

The thermal desorption total ion chromatogram (TIC) of both samples was dominated by carbon dioxide, sulphur dioxide, benzene and, more unusually, acetic acid. Although the similarity is notable and may suggest a common source, it is difficult to explain the significance of these common and non-diagnostic organic compounds, beyond indicating that these samples have undergone diagenetic changes of a highly oxidising nature. More interestingly, a series of *n*-alkanes, with carbon numbers notably similar were observed in the TD of both samples. More apparent in sample S1, which contained appreciably more thermally extractable organic material, these *n*-alkanes range from C_{16} up to C_{24}, maximising at C_{21}. Despite evidence for some microbially-derived biodegradation, the odd over even carbon number predominance of the *n*-alkanes, which dominate the hydrocarbon fraction, suggest the biodegradation has not been extensive and therefore preserves the 'chemical fingerprint' of the original source. This is notable since the relatively unusual mid-chain carbon numbers present strongly suggests a submerged aquatic plant (macrophyte)[89-91] (albeit possibly with a small bacterial/algal component, i.e. C_{16} to C_{18} alkanes), since these typically have a plant wax component ranging from C_{19} to C_{31}/C_{33} and maximising at C_{21}/C_{23}, possibly deriving from the original food consumed in this context. The persistence of the *n*-alkanes is not unexpected given that they are known to be particularly resistant to diagenesis[90,92]. In addition to the data above, an *n*-alkane proxy for the input/presence of submerged/floating freshwater aquatic macrophytes provides another corroborating measure of aquatic plant input[89] This proxy ratio, P_{aq}, gives values of 1.0 for this sample, which also indicates a submerged/floating aquatic plant[89].

The C_{21} maximum, combined with the relatively narrow *n*-alkane carbon number range in the calculus samples is indicative of biodegraded submerged aquatic plant (macrophyte) material. Although the identification of plant remains is challenging, the use of aquatic plants as food has been suggested. Specifically, the exploitation of the roots and tubers of aquatic plants in Mesolithic Europe has been hypothesised, with plant remains of eelgrass, horned pondweed

and beaked tassel weed identified at Argus Bank in Denmark[93] and a number of aquatic plants, including the submerged/floating species yellow water lily (*Nuphar lutea*), white water lily (*Nymphaea alba*) and pondweed (*Potamogeton pectinatus*), at Star Carr in England[94]. Interestingly, the chemical profile of the dominant C_{19} to C_{24} *n*-alkanes, maximising at C_{21} is consistent with the submerged aquatic plant genus *Nymphaea*[89]. Further work corroborating this possibility using microscopy would be needed, but it would – particularly in light of the suggested earlier exploitation in the British Isles – certainly be worthy of further investigation.

The pyrolysis-GC-MS of both samples revealed a series of ~C_{10} to C_{16} *n*-1-alkenes and *n*-alkanes, typical of unsaturated and saturated fat/oil-derived acyl lipids[5,6]. The absence of these compounds in the TD profile provides molecular evidence for a polymeric/bound acyl lipid source. Importantly, there were no bacterially-derived hydrocarbons deriving from the original branched fatty acid acyl groups which are significant components of bacterial triglycerides, indicating this lipid component is not bacterial in origin. However, it is not possible to be more precise about a possible source in these samples based on these lipid biomarkers alone. A suite of aromatic hydrocarbons observed in both samples from this site provides biomolecular evidence for exposure to fire/cooking.

Juniper Green, City of Edinburgh

Juniper Green is located on the western outskirts of Edinburgh in east central Scotland. The remains of an adult male accompanied by a Beaker were found in a short stone cist in 1851. The individual has been dated to 2335–2135 cal BC[87] (see also https://canmore.org.uk/site/50298).

27. Juniper Green, City of Edinburgh (NMS X.ET 33): M3 right back of tooth (calculus: 2.48 mg)

The three main compounds in the thermal desorption total ion chromatogram (TIC) (Fig. 20) are carbon dioxide, sulphur dioxide and 1-butene. In minor abundance are a series of alkenes and alkanes, carbon numbers C_{16} to C_{24}, within which there is series of C_{17} to C_{24} *n*-alkanes (odd over even predominance) dominating this and maximising at *n*-C_{21}. Branched alkanes with carbon numbers C_{16} to C_{22} are also present as very minor constituents and are possibly

indicative of limited microbially-derived biodegradation (but see summary below). These carbon numbers present in the TD chromatogram may suggest a submerged aquatic plant source (macrophyte)[89-91], possibly deriving from plant waxes in the original food consumed or reflecting prevailing environmental factors (but, again, see summary below). The 1-butene possibly derives from lipid or proteinaceous material in the sample[11], and perhaps produced at this relatively low temperature due to matrix effects. It is not possible to determine the significance of the carbon dioxide, beyond it being indicative of oxidised organic material. It should also be note that highly polar material could have been present, which would not have successfully eluted from the column, or indeed volatilised sufficiently in the probe.

The pyrolysate TIC revealed carbon dioxide, sulphur dioxide, the C_4 1-alkene, a C_7 diene, acetone, benzene, toluene and pyrrole as the only detectable components. The dominance of carbon dioxide, combined with the relatively minor abundance of the few small organic compounds present, suggests a highly oxidised sample. It is not possible to determine the significance of the carbon dioxide, beyond it being indicative of oxidised organic material and the other compounds are too low an abundance and too ubiquitous to provide any meaningful interpretation of their presence, beyond them being indicative of an organic origin. It should be noted, however, that highly polar material could have been present, which would not have successfully eluted from the column, or indeed volatilised sufficiently in the probe.

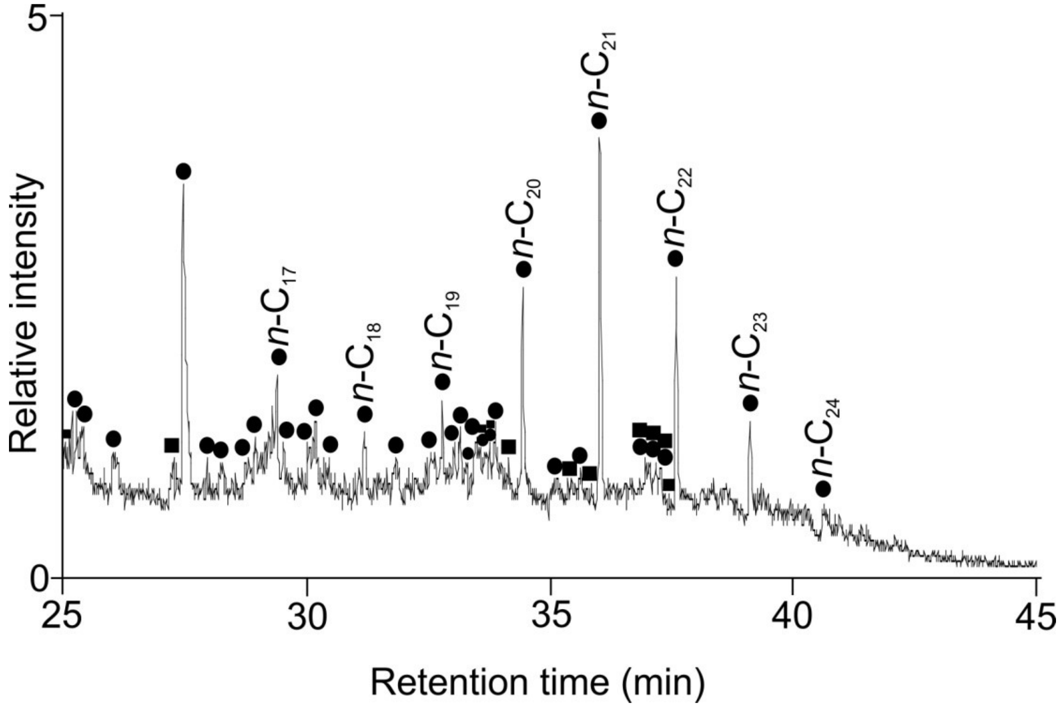

Supplementary Figure 20. Reconstructed total ion chromatogram of the thermal desorption profile (310 °C for 10 s) of sample X.ET 33, Juniper Green. Key to Fig. 20: Peak identities (x indicates carbon chain length): filled squares indicates alkenes; filled circles, Cx indicates alkanes.

Discussion, Juniper Green, City of Edinburgh

One sample of dental calculus from Juniper Green was analysed by sequential thermal desorption-gas chromatography-mass spectrometry (TD-GC-MS) and pyrolysis-gas chromatography-mass spectrometry (Py-GC-MS). The sample contained both free and bound/polymeric organic material.

The thermal desorption total ion chromatogram (TIC) of the sample was dominated by carbon dioxide, sulphur dioxide and 1-butene. It is difficult to explain the significance of these common and non-diagnostic organic compounds, beyond indicating that these samples have undergone diagenetic changes of a highly oxidising nature. Although only minor constituents, a series of *n*-alkanes, ranging from C_{17} up to C_{24} and maximising at C_{21}, were also detected. There is no odd over even carbon number predominance suggesting the samples may have undergone biodegradation as a result of diagenetic changes (although see below). This is also indicated by the presence of branched alkanes, although they are very minor in abundance compared to the *n*-alkane putative biomarkers. In this context, the relatively unusual mid-chain carbon numbers present suggests a submerged aquatic plant (macrophyte)[89-91] (albeit possibly with a small bacterial/algal component, i.e. C_{17} and C_{18} alkanes), since these typically have a plant wax component ranging from C_{19} to C_{31}/C_{33} and maximising at C_{21}/C_{23}, possibly deriving from the original food consumed in this context. The persistence of the *n*-alkanes is not unexpected given that they are known to be particularly resistant to diagenesis[90,92]. In addition to the data above, an *n*-alkane proxy for the input/presence of submerged/floating freshwater aquatic macrophytes provides another corroborating measure of aquatic plant input[89]. This proxy ratio, P_{aq}, gives values of 1.0 for this sample, which also indicates a submerged/floating aquatic plant[89].

The C_{21} maximum, combined with the relatively narrow *n*-alkane carbon number range in the calculus samples is indicative of biodegraded submerged aquatic plant (macrophyte) material. Although the identification of plant remains is challenging, the use of aquatic plants as food has been suggested. Specifically, the exploitation of the roots and tubers of aquatic plants in

Mesolithic Europe has been hypothesised, with plant remains of eelgrass, horned pondweed and beaked tassel weed identified at Argus Bank in Denmark[93] and a number of aquatic plants, including the submerged/floating species yellow water lily (*Nuphar lutea*), white water lily (*Nymphaea alba*) and pondweed (*Potamogeton pectinatus*), at Star Carr in England[94]. Interestingly, the chemical profile of the dominant C_{19} to C_{24} *n*-alkanes, maximising at C_{21} is consistent with the submerged aquatic plant genus *Nymphaea*[89]. Further work corroborating this possibility using microscopy would be needed, but it would – particularly in light of the suggested earlier exploitation in the British Isles – certainly be worthy of further investigation.

Of those bound/polymeric organic compounds identified, the dominance of carbon dioxide and the relatively minor abundance of only a few small organic compounds confirms a highly oxidised bound/polymeric organic component was present although its significance beyond this is necessarily unclear.

Ruchlaw Mains, East Lothian (NMS unreg)

A cist containing an adult male skeleton and a Beaker was discovered in 1979[95]. It has been radiocarbon dated to between 2340-2140 cal BC[87] (see also https://canmore.org.uk/site/57808/).

28. Ruchlaw Mains, East Lothian (NMS unreg): PM1,2 right lingual (calculus: 3.26 mg)

The six main compounds in the thermal desorption total ion chromatogram (TIC) (Fig. 21) are carbon dioxide, 1-butene, acetone, benzene, toluene and acetic acid. In minor abundance is an unresolved complex mixture (UCM; apparent as a ‘hump’ in the chromatogram) of alkenes and alkanes, carbon numbers C_{16} to C_{22}, within which there is series of C_{16} to C_{19} *n*-alkanes (with no odd over even predominance) dominating this UCM profile and maximising at *n*-C_{17}. Branched alkanes with carbon numbers C_{17} to C_{22} are also present as minor constituents. These carbon numbers present in the TD chromatogram, combined with the relative abundance of branched hydrocarbons compared to the *n*-alkanes suggests a bacterial source and certainly significant microbially-derived biodegradation (see discussion below). Indene and naphthalene were also detected, albeit as a minor constituents. No characteristic

protein or carbohydrate markers were observed meaning the significance of the non-diagnostic compounds carbon dioxide, benzene and acetic acid is uncertain. It should also be noted that highly polar material could have been present, which would not have successfully eluted from the column, or indeed volatilised sufficiently in the probe.

In contrast to the thermal desorption profile, the pyrolysis-GC-MS TIC (Fig. 21 inset) revealed a complex suite of organic compounds. These include a series of C_{10} to C_{19} *n*-1-alkenes and *n*-alkanes, typical of unsaturated and saturated fat/oil-derived acyl lipids[5,6]. The absence of these compounds in the TD profile provides molecular evidence for a polymeric/bound acyl lipid source. Importantly, there were no bacterially-derived hydrocarbons deriving from the original branched fatty acid acyl groups which are significant components of bacterial triglycerides, indicating this lipid component is not bacterial in origin. The pyrolysate TIC identified components indicative of 'black carbon', i.e. charcoal or soot[7-10]. Of these, benzene and toluene were major compounds present in the 'char' pyrolysate (~30% and 35% of total quantified pyrogram peak area respectively), along with moderate quantities of ethyl benzene and o-, m- and p-xylenes (~7% total). The relative dominance of toluene, rather than the normally dominant aromatic hydrocarbon benzene, is somewhat atypical of chars[7-10]. However, the presence of significant amounts of naphthalene, methyl naphthalenes and methyl biphenyl, together with the polynuclear aromatic hydrocarbon combustion markers 1-methyl-9H-fluorene, phenanthrene, 1-methylanthracene, pyrene and 1-methylpyrene, confirm a 'char' component providing molecular evidence for exposure to fire/cooking. The markers identified here can also be indicative of proteins[6,11] and combined with the related protein markers pyrrole, benzyl nitrile (phenylacetonitrile) and methyl benzyl nitrile identified this is indicative of these compounds, at least in part, originating from a protein-derived component[6,11]. The presence of carbon dioxide is also consistent with a lipid component in this sample. It should also be noted that highly polar material could have been present, which would not have successfully eluted from the column, or indeed volatilised sufficiently in the probe.

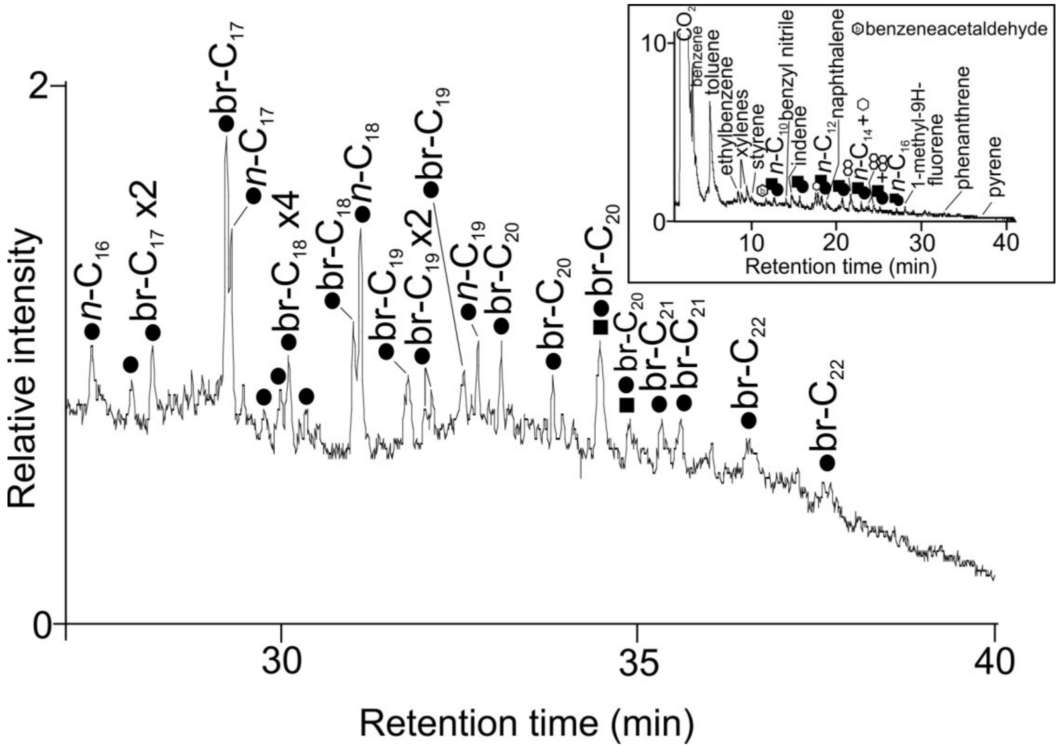

Supplementary Figure 21. Reconstructed total ion chromatogram of the thermal desorption profile (310 °C for 10 s) of sample NMS unreg, Ruchlaw Mains. Key to Fig. 21: Peak identities (x indicates carbon chain length): filled squares indicates alkenes; filled circles, Cx indicates alkanes. Inset displays a reconstructed total ion chromatogram of the pyrogram (pyrolysis profile) (610 °C for 10) of this sample, after thermal desorption (310 °C for 10 s). Peak identities: (x indicates carbon chain length): filled squares, Cx indicates alkenes; filled circles, Cx indicates alkanes; open hexagons indicate alkyl indenes; conjoined open hexagons indicate alkyl naphthalenes; CO_2 indicates carbon dioxide. Also shown are benzene, toluene, ethyl benzene, xylenes (*o*-, *m*- and *p*-), styrene, benzeneacetaldehyde, benzyl nitrile, indene, naphthalene, 1-methyl-9H-fluorene, phenanthrene and pyrene.

Discussion, Ruchlaw Mains, East Lothian

One sample of dental calculus from Ruchlaw Mains was analysed by sequential thermal desorption-gas chromatography-mass spectrometry (TD-GC-MS) and pyrolysis-gas chromatography-mass spectrometry (Py-GC-MS). The sample contained both free and bound/polymeric organic material.

The thermal desorption total ion chromatogram (TIC) was dominated by carbon dioxide, 1-butene, acetone, benzene, toluene and, more unusually, acetic acid. It is difficult to explain

the significance of these common and non-diagnostic organic compounds, beyond indicating that these samples have undergone diagenetic changes of a highly oxidising nature. In minor abundance wasan unresolved complex mixture of alkenes and alkanes, carbon numbers C_{16} to C_{22}, within which there is series of C_{16} to C_{19} *n*-alkanes (with no odd over even predominance) dominating this UCM profile and maximising at *n*-C_{17}. Branched alkanes with carbon numbers C_{17} to C_{22} are also present as minor constituents. These carbon numbers present in the TD chromatogram, combined with the relative abundance of branched hydrocarbons compared to the *n*-alkanes suggests a bacterial source and certainly significant microbially-derived biodegradation.

The pyrolysis-GC-MS revealed a series of ~C_{10} to C_{19} *n*-1-alkenes and *n*-alkanes, typical of unsaturated and saturated fat/oil-derived acyl lipids[5,6]. The absence of these compounds in the TD profile provides molecular evidence for a polymeric/bound acyl lipid source. Importantly, there were no bacterially-derived hydrocarbons deriving from the original branched fatty acid acyl groups which are significant components of bacterial triglycerides, indicating this lipid component is not bacterial in origin. However, it is not possible to be more precise about a possible source in these samples based on these lipid biomarkers alone. A suite of aromatic hydrocarbons observed provides biomolecular evidence for exposure to fire/cooking.

Skateraw (Roy Richie 1958 cist), East Lothian (NMS unreg)

A short stone cist containing an adult male skeleton and a Beaker was discovered in 1958[96]. The individual has been radiocarbon dated to 2340–2195 cal BC[87] (see also https://canmore.org.uk/site/57808/).

29. Skateraw (Roy Richie 1958 cist), East Lothian (NMS unreg): M1 right lingual (calculus: 3.28 mg)[28]

One sample of dental calculus from Skateraw was analysed by sequential thermal desorption-gas chromatography-mass spectrometry (TD-GC-MS) and pyrolysis-gas chromatography-mass spectrometry (Py-GC-MS). The sample contained virtually no free and little bound/polymeric organic material, most likely reflecting unfavourable prevailing conditions for biomolecular preservation. Of those organic compounds identified, the dominance of

carbon dioxide and the relatively minor abundance of only a few small organic compounds suggests that only highly oxidised organic material was present.

The thermal desorption total ion chromatogram (TIC) revealed carbon dioxide as the only detectable component, indicating the essential absence of free, thermally extractable organic components in this sample. It should be noted, however, that highly polar material could have been present, which would not have successfully eluted from the column, or indeed volatilised sufficiently in the probe. It is not possible to determine the significance of the carbon dioxide, beyond it being indicative of oxidised organic material.

The pyrolysate TIC revealed carbon dioxide, C_4 to C_6 1-alkenes, a methylcyclopentadiene, benzene and toluene as the only detectable components. The dominance of carbon dioxide, combined with the relatively minor abundance of the few small organic compounds present, suggests a highly oxidised sample. It is not possible to determine the significance of the carbon dioxide, beyond it being indicative of oxidised organic material and the other compounds are too low an abundance and too ubiquitous to provide any meaningful interpretation of their presence, beyond them being indicative of an organic origin. It should be noted, however, that highly polar material could have been present, which would not have successfully eluted from the column, or indeed volatilised sufficiently in the probe.

Lesmurdie cist B, Banff (NMS X.ET 14)

Several short stone cists containing individuals and Beakers were found at Lesmurdie on Scotland's north-east coast. One adult, probably male, aged 40–60 from cist B, associated with a Beaker and three flint chips, was radiocarbon dated to 2295–2040 cal BC[97] (see also https://canmore.org.uk/site/17297).

30. Lesmurdie cist B, Banff (NMS X.ET 14): R mandibular 2nd molar, labial (calculus: 1.47 mg)

One sample of dental calculus from Lesmurdie cist B was analysed by sequential thermal desorption-gas chromatography-mass spectrometry (TD-GC-MS) and pyrolysis-gas chromatography-mass spectrometry (Py-GC-MS). The sample contained little free and

bound/polymeric organic material, most likely reflecting unfavourable prevailing conditions for biomolecular preservation. Of those organic compounds identified, the dominance of carbon dioxide and the relatively minor abundance of only a few small organic compounds suggests that only highly oxidised organic material was present.

The thermal desorption total ion chromatogram (TIC) revealed carbon dioxide, sulphur dioxide and acetone as the only detectable components, indicating the essential absence of free, thermally extractable organic components in this sample. It should be noted, however, that highly polar material could have been present, which would not have successfully eluted from the column, or indeed volatilised sufficiently in the probe. It is not possible to determine the significance of the carbon dioxide, beyond it being indicative of oxidised organic material.

The pyrolysate TIC revealed carbon dioxide, sulphur dioxide, C_4 to C_6 1-alkenes, cyclopentadiene, benzene and toluene as the only detectable components. The dominance of carbon dioxide, combined with the relatively minor abundance of the few small organic compounds present, suggests a highly oxidised sample. It is not possible to determine the significance of the carbon dioxide, beyond it being indicative of oxidised organic material and the other compounds are too low an abundance and too ubiquitous to provide any meaningful interpretation of their presence, beyond them being indicative of an organic origin. It should be noted, however, that highly polar material could have been present, which would not have successfully eluted from the column, or indeed volatilised sufficiently in the probe.

Lithuania

Twenty-one samples of dental calculus taken from twenty-one individuals from six sites in Lithuania ranging in date form the Mesolithic to the 5-6th centuries AD, were analysed by sequential thermal desorption-gas chromatography-mass spectrometry (TD-GC-MS) and pyrolysis-gas chromatography-mass spectrometry (Py-GC-MS). This technique facilitates the identification of both free/unbound and bound/polymeric organic components. The twenty-one calculus samples produced very little free organic material, with only seven samples containing free compounds if the essentially ubiquitous and thermally generated carbon

dioxide is excluded. In contrast, fourteen of the twenty-one samples revealed a very minor to significant amount of a bound/polymeric organic constituent.

Kretuonas, Early – Middle Neolithic

The Kretuonas settlement belongs to a group of Neolithic-Bronze Age settlements discovered along the shore of Lake Kretuonas (Švenčionys district) in eastern Lithuania. Investigation of settlements was carried in 1978-1989. Human skeletal remains are fragmentary; some graves were destroyed through ploughing. Burial items include all kinds of tools made from flint, stone, bone, antler, clay and adornments made of amber and animal bones. Remains of domestic animals (cattle, pig, horses and dogs), nuts, shells of molluscs were found[98].

31. Kretuonas SA1 (calculus: 0.65 mg) Early – Middle Neolithic, 25-30 years old.

The thermal desorption total ion chromatogram (TIC) revealed no detectable components, indicating the absence of free, thermally extractable organic components in this sample. It should be noted, however, that highly polar material could have been present, which would not have successfully eluted from the column, or indeed volatilised sufficiently in the probe.

The pyrolysate TIC revealed carbon dioxide as the only detectable component, indicating the essential absence of organic material in this sample. It should be noted, however, that highly polar material could have been present, which would not have successfully eluted from the column, or indeed volatilised sufficiently in the probe. It is not possible to determine the significance of the carbon dioxide, beyond it being indicative of oxidised organic material, although its presence as only a very minor component makes even this inference of very little meaningful value.

Donkalnis and Spiginas

Mesolithic-Neolithic cemeteries are situated along the shores of Lake Biržulis (Telšiai district) in western Lithuania. Research excavations of the area were carried out between 1978 and 1993. Two archaeological complexes were discovered, namely the Mesolithic camp sites on the promontories of the lake peninsula (Spigino Ragas) and cemeteries and funeral feast and sacrifice pits on lake islands next to the camps (Donkalnis and Spiginas). In total the remains of 24 individuals from 17 graves were uncovered. Most graves contained one

person but at Donkalnis there were at least two double graves. The dead were placed straight on their backs or in a bent posture[99].

32. Donkalnis SA4, around 50 years old (calculus: 0.62 mg), Neolithic.

The thermal desorption total ion chromatogram (TIC) revealed carbon dioxide as the only detectable component, indicating the essential absence of free, thermally extractable organic components in this sample. It should be noted, however, that highly polar material could have been present, which would not have successfully eluted from the column, or indeed volatilised sufficiently in the probe. It is not possible to determine the significance of the carbon dioxide, beyond it being indicative of oxidised organic material.

The pyrolysate TIC revealed carbon dioxide as the only detectable component, indicating the essential absence of organic material in this sample. It should be noted, however, that highly polar material could have been present, which would not have successfully eluted from the column, or indeed volatilised sufficiently in the probe. It is not possible to determine the significance of the carbon dioxide, beyond it being indicative of oxidised organic material.

33. Spiginas SA5 (calculus: 1.63 mg). Middle Mesolithic (6412 – 6258 cal. BC), female, 30-35 years old.

The main compound in the thermal desorption total ion chromatogram (TIC) (Fig. 22) is carbon dioxide. In trace abundance are a series of *n*-alkanes, carbon numbers C_{19} to C_{22}, maximising at $C_{20/21}$. There is no odd over even carbon number predominance suggesting the sample may have undergone biodegradation as a result of diagenetic changes. This is further corroborated by the presence of branched alkanes with carbon numbers C_{20} and C_{21} observed as relatively minor constituents and indicative of microbially-derived biodegradation (see discussion below). The relatively unusual mid-chain carbon numbers present in the TD chromatogram suggests a submerged aquatic plant (macrophyte)[89-92], possibly deriving from plant waxes in the original food consumed or reflecting prevailing environmental factors (again, see discussion below). It is not possible to determine the significance of the carbon dioxide, beyond it being indicative of oxidised organic material. It should also be note that highly polar material could have been present, which would not have successfully eluted from the column, or indeed volatilised sufficiently in the probe.

The pyrolysate TIC revealed carbon dioxide and 1-butene as the only detectable components. The dominance of carbon dioxide, combined with the relatively minor abundance of 1-butene, suggests a highly oxidised sample. It is not possible to determine the significance of the carbon dioxide, beyond it being indicative of oxidised organic material. 1-Butene can be indicative of lipid or proteinaceous material in the sample[11], although its relatively minor abundance and the absence of other related biomarkers prevents any meaningful interpretation of its significance. It should be noted, however, that highly polar material could have been present, which would not have successfully eluted from the column, or indeed volatilised sufficiently in the probe.

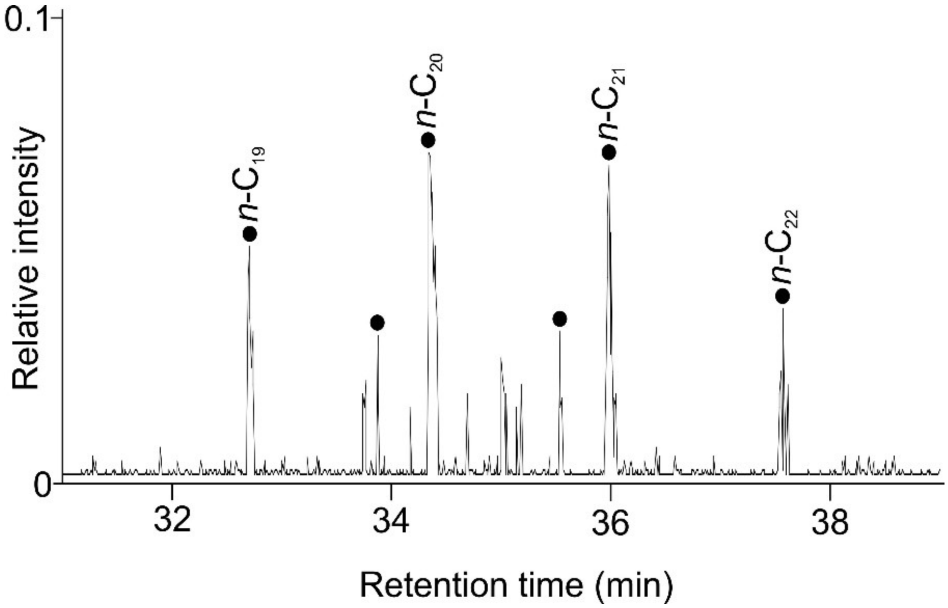

Supplementary Figure 22. Reconstructed total ion chromatogram of the thermal desorption profile (310 °C for 10 s) of sample 5A5 (Meso + Neo). Key to Fig. 22: Peak identities (x indicates carbon chain length): filled circles, Cx indicates alkanes.

34. Spiginas SA6 (calculus: 5.15 mg). Late Neolithic (2864 – 2488 cal. BC) Male, age 50-55 years old.

The thermal desorption total ion chromatogram (TIC) revealed carbon dioxide as the only detectable component, indicating the essential absence of free, thermally extractable organic components in this sample. It should be noted, however, that highly polar material could have been present, which would not have successfully eluted from the column, or indeed

volatilised sufficiently in the probe. It is not possible to determine the significance of the carbon dioxide, beyond it being indicative of oxidised organic material.

The pyrolysis-GC-MS TIC (Fig. 23) was dominated by carbon dioxide, 1-butene and 1-pentene, the alkenes being indicative of lipid or proteinaceous material in the sample[11]. Cyclopentadiene and methylcyclopentadiene are also components of the pyrolysate although it is not possible to ascribe a specific origin here. Toluene was also detected, albeit as a very minor component. In contrast to the thermal desorption profile, the pyrogram also revealed a series of C_7 to C_{16} *n*-1-alkenes and *n*-alkanes, typical of unsaturated and saturated fat/oil-derived acyl lipids[5,6]. The absence of these compounds in the TD profile provides molecular evidence for a polymeric/bound acyl lipid source. However, in addition to *n*-1-alkenes and *n*-alkanes, typical of fat/oil derived lipids, the pyrogram also displayed a series of C_8 to C_{15} iso-methyl-1-alkenes (isoalkenes) and 2-methylalkanes (isoalkanes), presumably deriving from the branched fatty acids which are significant components of bacterial triglycerides. The dominance of carbon dioxide suggests a highly oxidised sample. It is not possible to determine the significance of the carbon dioxide, beyond it being indicative of oxidised organic material. It should be noted, however, that highly polar material could have been present, which would not have successfully eluted from the column, or indeed volatilised sufficiently in the probe.

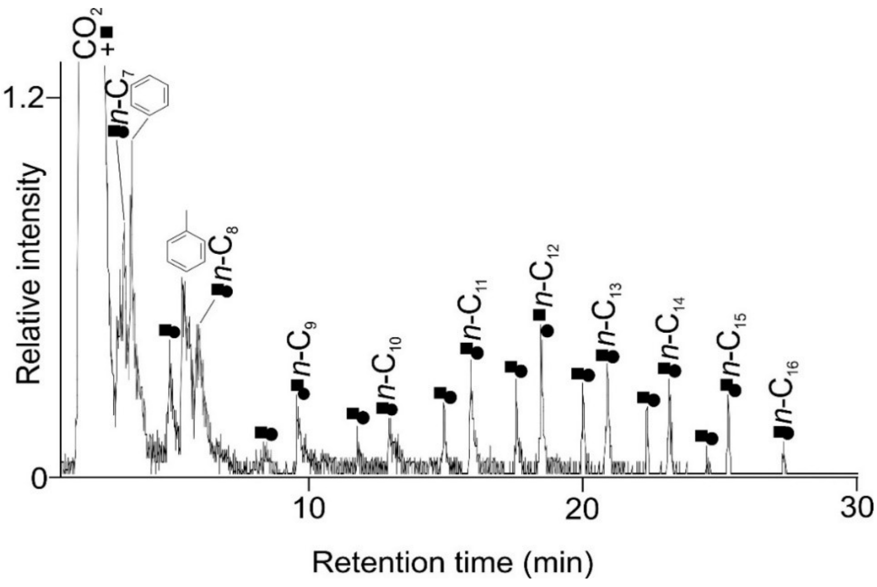

Supplementary Figure 23. Reconstructed total ion chromatogram of the pyrogram (pyrolysis profile) (610 °C for 10s) of sample 5A6 (Meso + Neo), after thermal desorption

(310 °C for 10 s). Key to Fig. 23: Peak identities (x indicates carbon chain length): filled squares, Cx indicates alkenes; filled circles, Cx indicates alkanes. Also shown are the structures of two aromatic compounds identified: benzene and toluene. CO_2indicates carbon dioxide.

35. Gyvakarai SA7. (calculus: 3.42 mg). Late Neolithic/Early Bronze Age, 2269 – 1977 cal. BC. Male, 35-40 years old.

The site of Gyvakarai (Kupiškis district) is situated in eastern Lithuania and is represented by one Late Neolithic - Bronze Age inhumation. The burial containing the skeletal remains of a middle-aged man was found accidentally in 2000. As the burial was disturbed the original position of the skeleton could not be reconstructed. Grave goods included a stone battle boat shaped axe and other flint items[100].

The thermal desorption total ion chromatogram (TIC) revealed carbon dioxide as the only detectable component, indicating the essential absence of free, thermally extractable organic components in this sample. It should be noted, however, that highly polar material could have been present, which would not have successfully eluted from the column, or indeed volatilised sufficiently in the probe. It is not possible to determine the significance of the carbon dioxide, beyond it being indicative of oxidised organic material.

The pyrolysate TIC revealed carbon dioxide as the only detectable component, indicating the essential absence of organic material in this sample. It should be noted, however, that highly polar material could have been present, which would not have successfully eluted from the column, or indeed volatilised sufficiently in the probe. It is not possible to determine the significance of the carbon dioxide, beyond it being indicative of oxidised organic material.

Discussion, Mesolithic and Neolithic samples, Lithuania.

Five samples of dental calculus from Mesolithic and Neolithic individuals were analysed by sequential thermal desorption-gas chromatography-mass spectrometry (TD-GC-MS) and pyrolysis-gas chromatography-mass spectrometry (Py-GC-MS). Three of the samples (5A1, SA4 and SA7) contained little free and bound/polymeric organic material, most likely reflecting their age and unfavourable prevailing conditions for biomolecular preservation.

Carbon dioxide was the only compound identified in these samples suggesting that only highly oxidised organic material was present.

Another sample, SA6, revealed only carbon dioxide in the TD-GC-MS analysis, indicating the essential absence of free, thermally extractable organic components in this sample. The carbon dioxide is also indicative of highly oxidised organic material. The pyrolysis-GC-MS revealed a series of C_7 to C_{16} *n*-1-alkenes and *n*-alkanes, typical of unsaturated and saturated fat/oil-derived acyl lipids[5,6]. The absence of these compounds in the TD profile provides molecular evidence for a polymeric/bound acyl lipid source. However, the additional presence of a series of C_8 to C_{15} isoalkenes and isoalkanes, presumably deriving from the branched fatty acids, which are significant components of bacterial triglycerides, suggests a microbial origin for these lipid biomarkers, at least in part (also see below). The dominance of carbon dioxide again suggests a highly oxidised sample.

The last Mesolithic/Neolithic sample, SA5, revealed both free and bound/polymeric organic material. The thermal desorption total ion chromatogram (TIC) of the sample was dominated by carbon dioxide; it is difficult to explain the significance of this non-diagnostic compound, beyond indicating that this sample has undergone diagenetic changes of a highly oxidising nature. Although only trace constituents, a series of *n*-alkanes, ranging from C_{19} up to C_{22} and maximising at $C_{20/21}$, were also detected. There is no odd over even carbon number predominance suggesting the samples may have undergone biodegradation as a result of diagenetic changes (although see below). This is also indicated by the presence of branched alkanes, although they are minor in abundance compared to the *n*-alkane putative biomarkers. In this context, the relatively unusual mid-chain carbon numbers present suggests a submerged aquatic plant (macrophyte)[89-92], since these typically have a plant wax component ranging from C_{19} to C_{31}/C_{33} and maximising at C_{21}/C_{23}, possibly deriving from the original food consumed in this context. The persistence of the *n*-alkanes is not unexpected given that they are known to be particularly resistant to diagenesis[90,92].

In addition to the data above, an *n*-alkane proxy for the input/presence of submerged/floating freshwater aquatic macrophytes can provide another corroborating measure for an aquatic plant input[89]. However, the *n*-alkane proxy ratio, P_{aq}, cannot be applied meaningfully here because of the lack of $>C_{23}$ n-alkanes. That being said, the $C_{20/21}$ maximum, combined with the relatively narrow *n*-alkane carbon number range in the calculus sample is indicative of biodegraded submerged aquatic plant (macrophyte) material. Although the identification of

plant remains is challenging, the use of aquatic plants as food has been suggested. Specifically, the exploitation of the roots and tubers of aquatic plants in Mesolithic Europe has been hypothesised, with plant remains of eelgrass, horned pondweed and beaked tassel weed identified at Argus Bank in Denmark[93] and a number of aquatic plants, including the submerged/floating species yellow water lily (*Nuphar lutea*), white water lily (*Nymphaea alba*) and pondweed (*Potamogeton pectinatus*), at Star Carr in England[94]. Interestingly, the chemical profile of the dominant C_{19} to C_{22}*n*-alkanes, maximising at C_{21} is consistent with the submerged aquatic plant genus *Nymphaea*[89], following biodegradation. Further work corroborating this possibility using microscopy would be needed, but it certainly be worthy of further investigation. With carbon preference indexes (CPIs) of 1.12 and a $C_{20/21}$ maximum it is consistent with what would be expected for a (biodegraded) submerged aquatic plant. Notably similar values have been observed for submerged aquatic plant rhizomes from southern Europe: Greece, CPI = 1.13; France, CPI = 1.15[101]. Furthermore, the rhizomes in these studies had a far less pronounced odd-over-even carbon preference than leaves from the same plants, which is likely to reflect their microenvironment in the aquatic/marine sediments where they are far more exposed to biomolecular reworking and biodegradation from microbial inputs than leaves from the same plant. Of those bound/polymeric organic compounds identified, the dominance of carbon dioxide and the relatively minor abundance of 1-butene as the only other organic compound identified confirms a highly oxidised bound/polymeric organic component was present although its significance beyond this is necessarily unclear.

Obeliai, Central Lithuania flat burials. Lithuania People's Wandering (AD 5th-6th century).

Obeliai burial ground is situated on the shore of Lake Obeliai (Ukmergės district) in central Lithuania. Investigations carried in 1978-1983 revealed 65 inhumations, 6 cremated burials, and 5 horse graves, dated 5th-6th A.D. The western part of the Iron Age burial ground abuts 14th-15th A.D. Obeliai cemetery[102].

36. Obeliai, Central Lithuania flat burials. Sk1128 male 50-55 yrs T32 labial, 1A5 (calculus: 6.83 mg)

The main compound in the thermal desorption total ion chromatogram (TIC) (Fig. 24) is carbon dioxide. In minor abundance are a series of *n*-alkanes, carbon numbers C_{15} to C_{23}, maximising at C_{21}. There is no odd over even carbon number predominance suggesting the sample may have undergone biodegradation as a result of diagenetic changes. This is further corroborated by the presence of branched alkanes with carbon numbers C_{19} to C_{22} observed as relatively minor constituents and indicative of microbially-derived biodegradation (see discussion below). The relatively unusual mid-chain carbon numbers present in the TD chromatogram suggests a submerged aquatic plant (macrophyte)[89-92] component, possibly deriving from plant waxes in the original food consumed or reflecting prevailing environmental factors (again, see discussion below), in addition to a bacterial input (C_{15} to C_{18} alkanes)[92,103]. It is not possible to determine the significance of the carbon dioxide, beyond it being indicative of oxidised organic material. It should also be note that highly polar material could have been present, which would not have successfully eluted from the column, or indeed volatilised sufficiently in the probe.

The pyrolysis-GC-MS TIC (Fig. 24, inset) was dominated by carbon dioxide, 1-butene and 1-pentene, the alkenes being indicative of lipid or proteinaceous material in the sample[11]. Cyclopentadiene and methylcyclopentadiene are also significant components of the pyrolysate although it is not possible to ascribe a specific origin here. In contrast to the thermal desorption profile, the pyrogram also revealed a series of C_6 to C_{16} *n*-1-alkenes and *n*-alkanes, typical of unsaturated and saturated fat/oil-derived acyl lipids[5,6]. The absence of these compounds in the TD profile provides molecular evidence for a polymeric/bound acyl lipid source. However, in addition to *n*-1-alkenes and *n*-alkanes, typical of fat/oil derived lipids, the pyrogram also displayed a series of C_9 to C_{16} iso-methyl-1-alkenes (isoalkenes) and 2-methylalkanes (isoalkanes), presumably deriving from the branched fatty acids which are significant components of bacterial triglycerides.

The pyrolysate TIC identified components indicative of 'black carbon', i.e. charcoal or soot[7-10]. Of these, benzene and toluene were major compounds present in the typical 'char' component pyrolysate (~50% and 30% of total quantified 'char' respectively), along with moderate quantities of ethyl benzene and o-, m- and p-xylenes (~7% total). The protein marker pyridine[11] was present in moderate abundance (~8%) and despite the absence of higher molecular weight 'char' organic compounds their collective presence provides at least

tentative molecular evidence for exposure to fire/cooking. The dominance of carbon dioxide suggests a highly oxidised sample. It is not possible to determine the significance of the carbon dioxide, beyond it being indicative of oxidised organic material. It should be noted, however, that highly polar material could have been present, which would not have successfully eluted from the column, or indeed volatilised sufficiently in the probe.

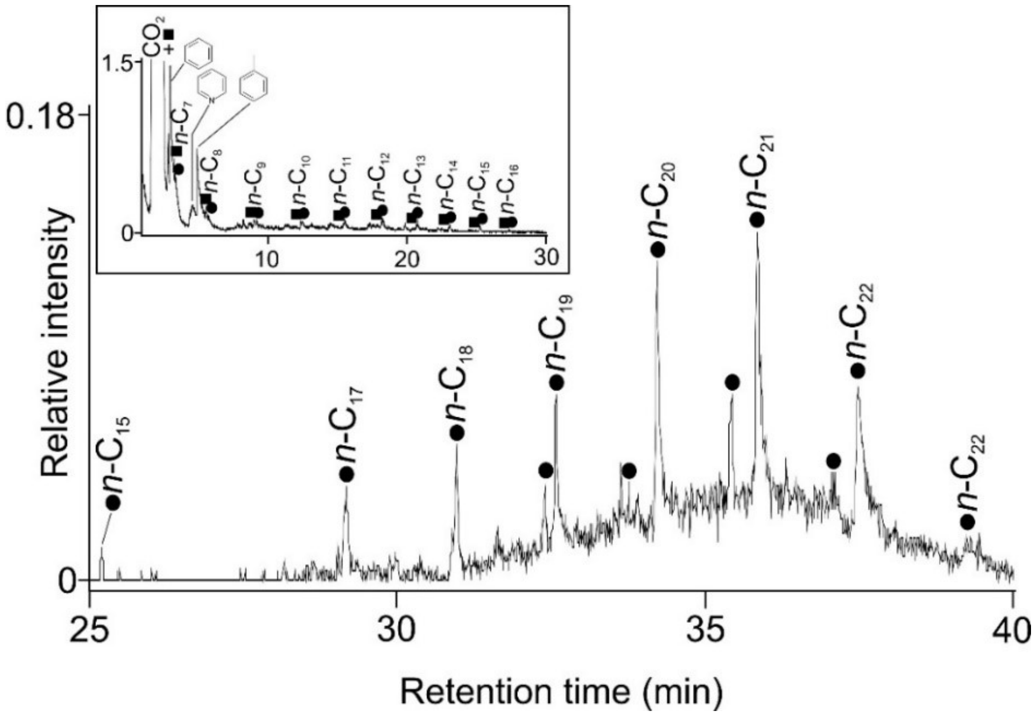

Supplementary Figure 24. Reconstructed total ion chromatogram of the thermal desorption profile (310 °C for 10 s) of sample 1A5, People's Wandering (AD 5th-6th century), Obeliai. Key to Fig. 24: Peak identities (x indicates carbon chain length): filled circles, Cx indicates alkanes. Inset displays a reconstructed total ion chromatogram of the pyrogram (pyrolysis profile) (610 °C for 10) of this sample, after thermal desorption (310 °C for 10 s). Peak identities: (x indicates carbon chain length): filled squares, Cx indicates alkenes; filled circles, Cx indicates alkanes. CO_2 indicates carbon dioxide. Also shown are the structures of three aromatic compounds identified: benzene, toluene and pyridine.

37. Obeliai, Central Lithuania flat burials. Lithuania People's Wandering (AD 5th-6th century), Sk1132 female 30-35 yrs T41 lingual, 1A6 (calculus: 2.91 mg)

The thermal desorption total ion chromatogram (TIC) revealed carbon dioxide as the only detectable component, indicating the essential absence of free, thermally extractable organic components in this sample. It should be noted, however, that highly polar material could have been present, which would not have successfully eluted from the column, or indeed volatilised sufficiently in the probe. It is not possible to determine the significance of the carbon dioxide, beyond it being indicative of oxidised organic material.

The pyrolysis-GC-MS TIC (Fig. 25) was dominated by carbon dioxide, 1-butene and 1-pentene, the alkenes being indicative of lipid or proteinaceous material in the sample[11]. Cyclopentadiene and methylcyclopentadiene are also significant components of the pyrolysate although it is not possible to ascribe a specific origin here. Toluene was also detected, albeit as a minor component. In contrast to the thermal desorption profile, the pyrogram also revealed a series of C_7 to C_{16} *n*-1-alkenes and *n*-alkanes, typical of unsaturated and saturated fat/oil-derived acyl lipids[5,6]. The absence of these compounds in the TD profile provides molecular evidence for a polymeric/bound acyl lipid source. However, in addition to *n*-1-alkenes and *n*-alkanes, typical of fat/oil derived lipids, the pyrogram also displayed a series of C_8 to C_{16} iso-methyl-1-alkenes (isoalkenes) and 2-methylalkanes (isoalkanes), presumably deriving from the branched fatty acids which are significant components of bacterial triglycerides. The dominance of carbon dioxide suggests a highly oxidised sample. It is not possible to determine the significance of the carbon dioxide, beyond it being indicative of oxidised organic material. It should be noted, however, that highly polar material could have been present, which would not have successfully eluted from the column, or indeed volatilised sufficiently in the probe.

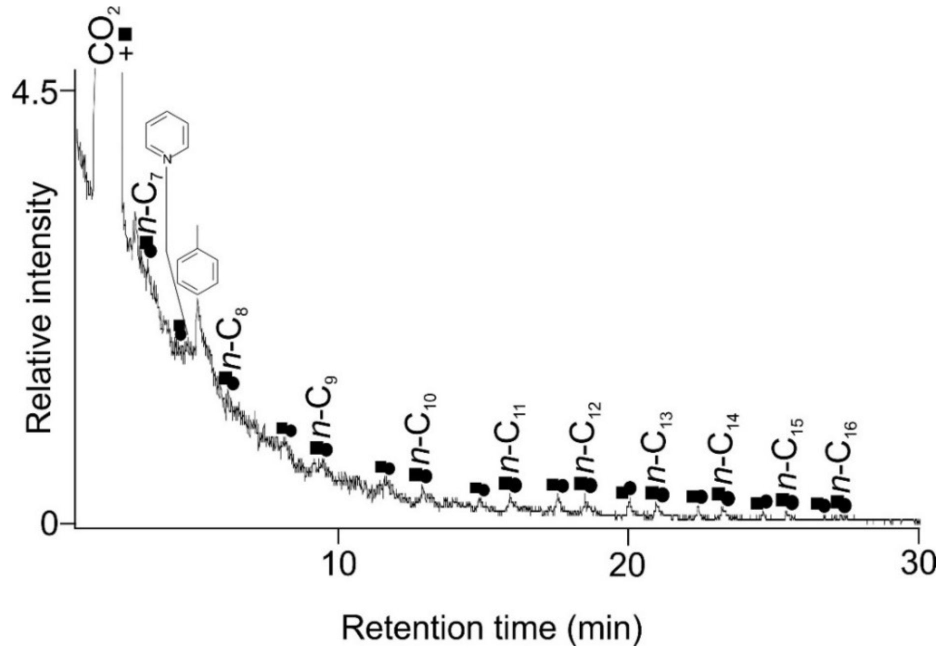

Supplementary Figure 25. Reconstructed total ion chromatogram of the thermal desorption profile (310 °C for 10 s) of sample 1A6, People's Wandering (AD 5th-6th century), Obeliai. Key to Fig. 25: Peak identities (x indicates carbon chain length): filled circles, Cx indicates alkanes. Inset displays a reconstructed total ion chromatogram of the pyrogram (pyrolysis profile) (610 °C for 10) of this sample, after thermal desorption (310 °C for 10 s). Peak identities: (x indicates carbon chain length): filled squares, Cx indicates alkenes; filled circles, Cx indicates alkanes. CO_2 indicates carbon dioxide. Also shown are the structures of two aromatic compounds identified: pyridine and toluene.

38. Lithuania People's Wandering (AD 5th-6th century), Obeliai, Central Lithuania flat burials, Sk1289 male 30-35 yrs T32 labial, 1A8 (calculus: 5.09 mg)

The main compound in the thermal desorption total ion chromatogram (TIC) (Fig. 26, inset) is carbon dioxide. In trace abundance are a series of *n*-alkanes, carbon numbers C_{17} to C_{22}, maximising at C_{19}. There is an odd over even carbon number predominance, although the relative abundance of the C_{20} and C_{22} *n*-alkanes suggests the sample has undergone some biodegradation as a result of diagenetic changes. Although the trace quantities and evidence for biodegradation necessarily mean any inference must be tentative, the relatively unusual mid-chain carbon numbers present in the TD chromatogram, combined with the C_{19} *n*-alkane being dominant and an odd over even carbon number predominance, suggests an algal source[92,101,103] (see discussion below). It is not possible to determine the significance of the

carbon dioxide, beyond it being indicative of oxidised organic material. It should also be note that highly polar material could have been present, which would not have successfully eluted from the column, or indeed volatilised sufficiently in the probe.

The pyrolysis-GC-MS TIC (Fig. 26) was dominated by carbon dioxide, 1-butene and 1-pentene, the alkenes being indicative of lipid or proteinaceous material in the sample[11]. Cyclopentadiene and methylcyclopentadiene are also significant components of the pyrolysate although it is not possible to ascribe a specific origin here. Acetone was also identified and can be indicative of the amino acid glycine or a carbohydrate component, although any interpretation should be considered tentative here. In contrast to the thermal desorption profile, the pyrogram also revealed a series of C_7 to C_{20} *n*-1-alkenes and *n*-alkanes, typical of unsaturated and saturated fat/oil-derived acyl lipids[5,6]. A distribution with maxima at C_{13} suggests the original acyl lipids may have been high in unsaturated fatty acids. The absence of these compounds in the TD profile provides molecular evidence for a polymeric/bound acyl lipid source. However, in addition to *n*-1-alkenes and *n*-alkanes, typical of fat/oil derived lipids, the pyrogram also displayed a series of C_9 to C_{16} iso-methyl-1-alkenes (isoalkenes) and 2-methylalkanes (isoalkanes), presumably deriving from the branched fatty acids which are significant components of bacterial triglycerides.

A number of the biomarkers identified here can be indicative of proteins[6,11], i.e. toluene, pyridine, pyrrole. This is also corroborated by the presence of aliphatic nitriles (carbon numbers $C_{14:0}$, *i*-$C_{15:0}$, $C_{16:0}$ (max) and $C_{18:0}$) suggesting a lipid (fat/oil) in this sample which has combined with protein-derived nitrogen to produce these moieties[5]. However, notable in this context is the additional presence of appreciable amounts of the more unusual 2-methylpyrroles, 2-ethylpyrrole and an ethylmethylpyrrole. Collectively, these C_1, C_2, and C_3 C_4 are possibly not detected due to the low abundance of organic material present) alkyl pyrroles can be indicative of a tetrapyrrole/porphyrin-derived origin and here may tentatively suggest an algal source observed in fossils and expected in similarly diagenetically altered archaeological material[11,21,22]. The presence here of the protein markers and specifically the C_1 to C_3 alkyl pyrroles, combined with the particular lipid profile, tentatively suggests the presence of a macroalgae, possibly a freshwater source, in this sample[21-23] (see discussion). The dominance of carbon dioxide suggests a highly oxidised sample. It is not possible to determine the significance of the carbon dioxide, beyond it being indicative of oxidised organic material. It should be noted, however, that highly polar material could have been

present, which would not have successfully eluted from the column, or indeed volatilised sufficiently in the probe.

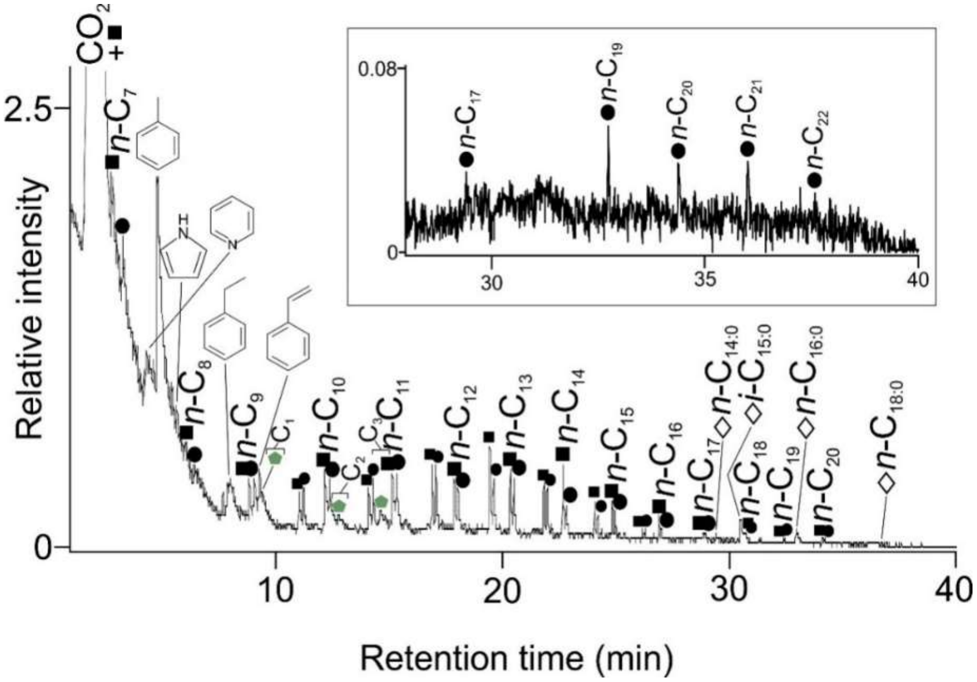

Supplementary Figure 26. Reconstructed total ion chromatogram of the pyrogram (pyrolysis profile) (610 °C for 10s) of sample 1A8, People's Wandering (AD 5th-6th century), Obeliai, after thermal desorption (310 °C for 10 s). Key to Fig. 26: Peak identities (x indicates carbon chain length): filled squares, Cx indicates alkenes; filled circles, Cx indicates alkanes; open diamonds, Cx:y indicates acyclic nitriles; green filled pentagons indicates alkyl pyrroles (with alkyl chain length above, Cx). Also shown are the structures of five aromatic compounds identified: toluene, ethyl benzene, styrene, pyridine and pyrrole. CO_2 indicates carbon dioxide. Inset displays a reconstructed total ion chromatogram of the thermal desorption profile (310 °C for 10 s) of this sample. Peak identities: (x indicates carbon chain length): filled circles, Cx indicates alkanes.

39. Lithuania People's Wandering (AD 5th-6th century), Obeliai, Central Lithuania flat burials, Sk1294 male 50-55 yrs T43 lingual, 1A9 (calculus: 13.87 mg)

The main compound in the thermal desorption total ion chromatogram (TIC) (Fig. 27, inset) is carbon dioxide. In trace abundance are a series of *n*-alkanes, carbon numbers C_{15} to C_{22},

maximising at C_{17}. There is a slight odd over even carbon number predominance, although the relative abundance of the C_{18} and C_{20} *n*-alkanes and the presence of C_{15} to C_{21} branched chain alkanes suggests the sample has undergone some microbial-derived biodegradation as a result of diagenetic changes. Although the trace quantities and evidence for biodegradation necessarily mean any inference must be tentative, the relatively unusual mid-chain carbon numbers present in the TD chromatogram, combined with the C_{17} *n*-alkane being dominant and a slight odd over even carbon number predominance, suggests an algal source[92,101,103] (see discussion below). It is not possible to determine the significance of the carbon dioxide, beyond it being indicative of oxidised organic material. It should also be note that highly polar material could have been present, which would not have successfully eluted from the column, or indeed volatilised sufficiently in the probe.

The pyrolysis-GC-MS TIC (Fig. 27) was dominated by carbon dioxide, 1-butene and 1-pentene, the alkenes being indicative of lipid or proteinaceous material in the sample[11]. Cyclopentadiene and methylcyclopentadiene are also significant components of the pyrolysate although it is not possible to ascribe a specific origin here. In contrast to the thermal desorption profile, the pyrogram also revealed a series of C_7 to C_{20} *n*-1-alkenes and *n*-alkanes, typical of unsaturated and saturated fat/oil-derived acyl lipids[5,6]. A distribution with maxima at C_{13}/C_{14} suggests the original acyl lipids may have been high in unsaturated fatty acids. The absence of these compounds in the TD profile provides molecular evidence for a polymeric/bound acyl lipid source. However, in addition to *n*-1-alkenes and *n*-alkanes, typical of fat/oil derived lipids, the pyrogram also displayed a series of C_8 to C_{16} iso-methyl-1-alkenes (isoalkenes) and 2-methylalkanes (isoalkanes), presumably deriving from the branched fatty acids which are significant components of bacterial triglycerides.

There were components identified in the pyrolysate TIC which can be indicative of 'black carbon', i.e. charcoal or soot[7-10]. However, the absence of naphthalenes and biphenyls and the dominance of tolu-ne, rather than the normally dominant aromatic hydrocarbon benzene, is notably atypical of chars[7-10] The markers identified here can also be indicative of proteins[6,11] and combined with the abundant related protein markers pyrrole, benzonitrile, benzyl nitrile (phenylacetonitrile), benzenepropanenitrile and indoles[6,11] identified this is likely to be the main origin of these compounds in this sample, pointing to a substantial protein-derived component[6,11]. This is also corroborated by the presence of aliphatic nitriles (carbon numbers *n*-$C_{14:0}$, *i*-$C_{15:0}$, *n*-$C_{15:0}$, *i*-$C_{16:0}$, *n*-$C_{16:0}$ (max) and *n*-$C_{18:0}$) suggesting a lipid (fat/oil) in this sample which has combined with protein-derived nitrogen to produce these moieties[5]. Taken

together, the presence of toluene, styrene and phenylacetonitrile (benzyl nitrile) can indicate the amino acid phenylalanine[12,13]. In addition, the significant constituents indole and 3-methylindole (skatole), with a lesser amount of a C_2 indole (3-ethylindole?), were also identified, which are consistent with a tryptophan amino acid source[12,13,19,20]. Proteins rich in the amino acids phenylalanine and tryptophan include meat, fish and dairy products, although the presence of bacterial markers means the possibility that at least a partial origin could be microbial cannot be excluded.

However, highly notable in this context is the additional presence of notably significant amounts of the more unusual alkyl pyrroles. C_1 alkyl pyrroles: 2- and 3-methylpyrroles; C_2 alkyl pyrroles: 2- and 3-ethylpyrroles, 2,4-, 2,3- and 3,4-dimethyl-pyrroles; C_3 alkyl pyrroles: 2-ethyl-4(?)-methylpyrrole, 2-ethyl-3(?)-methylpyrrole, 4-ethyl-2-methylpyrrole, 3-ethyl-4-methylpyrrole, and 2,3,5-trimethylpyrrole; C_4 alkyl pyrroles: two unidentified propylmethylpyrroles, four unidentified ethyldimethylpyrroles, 2,5(?)-diethylpyrrole, 2,4(?)-diethylpyrrole, 2,3(?)-diethylpyrrole, 3,4(?)-diethylpyrrole, 4-ethyl-2,3-dimethylpyrrole, 3-ethyl-2,4-dimethylpyrrole and 2,3,4,5-tetramethylpyrrole; C_5 alkyl pyrroles: 2,3(?)-diethyl-5?-methylpyrrole, 2,3-diethyl-4-methylpyrrole, 2,5?-diethyl-3-methylpyrrole and 3-ethyl-2,4,5-trimethylpyrrole; C_6 alkyl pyrroles: an unidentified C_6 ethylpyrrole. Collectively, these specific C_1, C_2, C_3, C_4, C_5 and C_6 alkyl pyrroles are characteristic of a significant tetrapyrrole/porphyrin-derived origin and are indicative of an algal source observed in fossils and expected in similarly diagenetically altered archaeological material[11,21,22]. Notable also in this context were pyridine and its methyl-, and dimethyl derivatives (these pyridines can result from the pyrolysis of polypeptides containing the amino acid alanine[19]). The presence here of the abundant protein markers and specifically the C_1 to C_6 alkyl pyrroles, combined with the particular lipid profile and specific carbohydrate markers (see below) identified, indicate the presence of a macroalgae, i.e. seaweed or freshwater source, in this sample[21-23] (see discussion).

The thermally-derived carbohydrate markers 2-methylfuran, 2-methyl-2-cyclopenten-1-one and 2,3-dimethyl-2-cyclopenten-1one were identified as significant components. That they were absent in the TD profile points to their originating from the original polymeric carbohydrate source (e.g. starch)[25]. Although levoglucosan (1,6-anhydro-β-D-glucopyranose) is usually the major component in the thermally-derived products of carbohydrates analysed by TD/Py-GC-MS, its absence from the TD profile here is consistent with the removal of hydroxyl groups in the original carbohydrate polymer, so preventing its formation in the

probe. The additional absence of the normally significant carbohydrate markers 2-hydroxy-3-methyl-2-cyclopenten-1-one and 3-hydroxy-2-methyl-2-cyclopenten-1-one observed in pure standard material (glycogen, starch and cellulose) would also support this interpretation. This shows a strong matrix effect likely to be due to the inorganic component of the calculus and the microflora in the mouth. This dehydration/dehydroxylation has also been observed in dog calculus and 19th century human calculus[6], where other markers, e.g. a prevalence of nitriles, with amides very minor, deriving from dehydrating conditions in the mouth of dogs and humans (though not sheep[6]) supporting this view[6]. The presence of carbon dioxide is also consistent with a lipid, amino acid/protein and carbohydrate component in this sample. Notably, these findings did not reveal key biomarkers typically observed in soils[20,23]. It should also be noted that highly polar material could have been present, which would not have successfully eluted from the column, or indeed volatilised sufficiently in the probe.

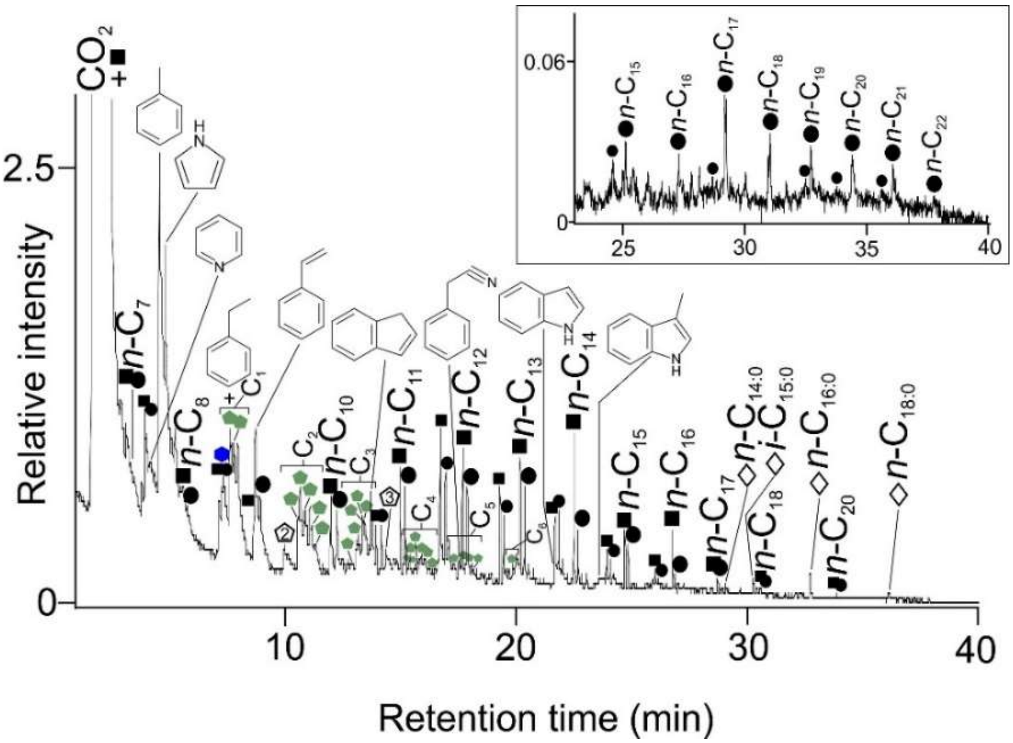

Supplementary Figure 27. Reconstructed total ion chromatogram of the pyrogram (pyrolysis profile) (610 °C for 10s) of sample 1A9, People's Wandering (AD 5th-6th century), Obeliai, after thermal desorption (310 °C for 10 s). Key to Fig. 27: Peak identities (x indicates carbon chain length): filled squares, Cx indicates alkenes; filled circles, Cx indicates

alkanes; open diamonds, Cx:y indicates acyclic nitriles; blue filled hexagon indicates alkyl pyridine; green filled pentagons indicates alkyl pyrroles (with alkyl chain length above, Cx); open pentagons with numerals inside indicates carbohydrate pyrolysis markers, 2 is 2-methyl-2-cyclopenten-1-one and 3 is 2,3-dimethyl-2-cyclopentene-1-one. Also shown are the structures of nine aromatic compounds identified: pyridine, pyrrole, toluene, ethylbenzene, styrene, indene, benzyl nitrile, indole and 3-methylindole (skatole). CO_2 indicates carbon dioxide. Inset displays a reconstructed total ion chromatogram of the thermal desorption profile (310 °C for 10 s) of this sample. Peak identities: (x indicates carbon chain length): filled circles, Cx indicates alkanes.

40. Lithuania People's Wandering (AD 5th-6th century), Obeliai, Central Lithuania flat burials, Sk0984 female >55 yrs T33 lingual, 1A10 (calculus: 3.74 mg)

The main compound in the thermal desorption total ion chromatogram (TIC) (Fig. 28) is carbon dioxide. In very minor abundance are a series of *n*-alkanes, carbon numbers C_{17} to C_{23}, maximising at C_{20}/C_{21}. There is no odd over even carbon number predominance suggesting the sample may have undergone biodegradation as a result of diagenetic changes. This is further corroborated by the presence of branched alkanes with carbon numbers C_{19} to C_{22} observed as relatively minor constituents and indicative of microbially-derived biodegradation (see discussion below). The relatively unusual mid-chain carbon numbers present in the TD chromatogram suggests a submerged aquatic plant (macrophyte)[89-92] component, possibly deriving from plant waxes in the original food consumed or reflecting prevailing environmental factors (again, see discussion below), in addition to a bacterial input (C_{17} and C_{18} alkanes)[92,103]. It is not possible to determine the significance of the carbon dioxide, beyond it being indicative of oxidised organic material. It should also be note that highly polar material could have been present, which would not have successfully eluted from the column, or indeed volatilised sufficiently in the probe.

The pyrolysis-GC-MS TIC was dominated by carbon dioxide, 1-butene, 1-pentene and hexene, the alkenes being indicative of lipid or proteinaceous material in the sample[11]. Cyclopentadiene and methylcyclopentadiene are also significant components of the pyrolysate although it is not possible to ascribe a specific origin here. Toluene was also detected, albeit as a very minor component, preventing any meaningful interpretation of its presence, beyond it being indicative of an organic origin. The dominance of carbon dioxide

suggests a highly oxidised sample. It is not possible to determine the significance of the carbon dioxide, beyond it being indicative of oxidised organic material. It should be noted, however, that highly polar material could have been present, which would not have successfully eluted from the column, or indeed volatilised sufficiently in the probe.

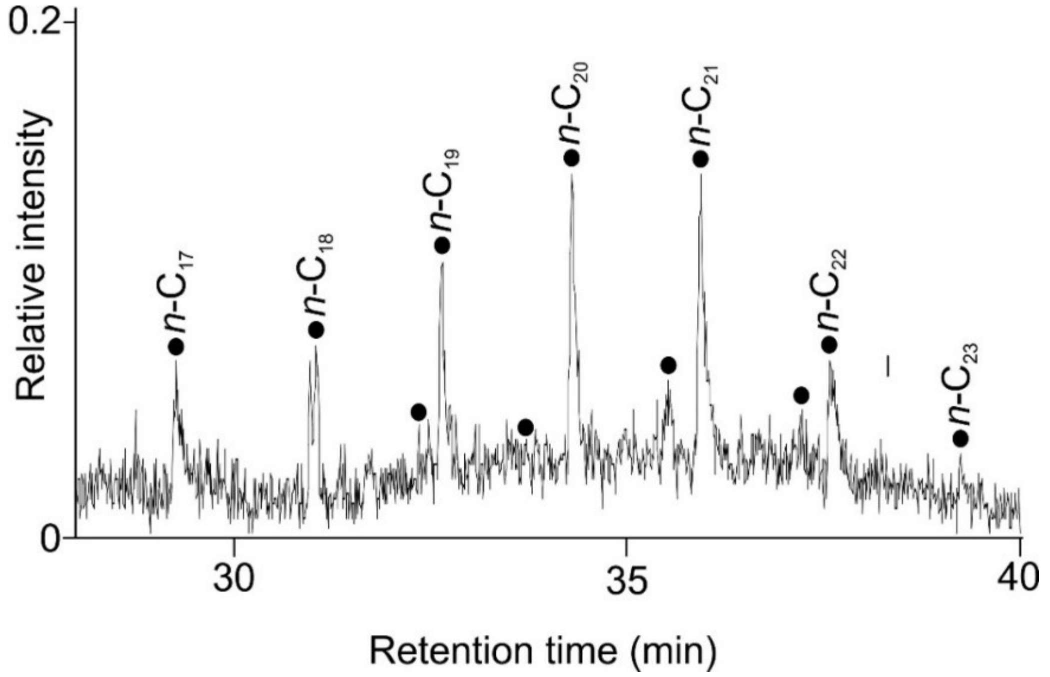

Supplementary Figure 28. Reconstructed total ion chromatogram of the thermal desorption profile (310 °C for 10 s) of sample 1A9, People's Wandering (AD 5th-6th century), Obeliai. Key to Fig. 28: Peak identities (x indicates carbon chain length): filled circles, Cx indicates alkanes.

Discussion, People's Wandering (AD 5th-6th century), Obeliai, Central Lithuania flat burials

Five samples of dental calculus from individual burials at Obeliai dating to the People's Wandering (AD 5th–6th century) were analysed by sequential thermal desorption-gas chromatography-mass spectrometry (TD-GC-MS) and pyrolysis-gas chromatography-mass spectrometry (Py-GC-MS).

Two of the samples taken from two separate individuals (1A5 and 1A10) revealed a series of *n*-alkanes, ranging from C_{17} up to C_{23} and maximising at C_{21}, as notable, albeit minor,

constituents of the thermal desorption total ion chromatogram (TIC), which were both dominated by the ubiquitous carbon dioxide, indicative of highly oxidised organic material. There is no odd over even carbon number predominance suggesting the samples have undergone biodegradation as a result of diagenetic changes (although see below). This is also indicated by the presence of branched alkanes with carbon numbers C_{19} to C_{22} observed as relatively minor constituents compared to the *n*-alkane putative biomarkers and indicative of microbially-derived biodegradation. In this context, the relatively unusual mid-chain carbon numbers present suggests a submerged aquatic plant (macrophyte)[89-92], in addition to a bacterial input evidenced by the C_{17} and C_{18} *n*-alkanes[92,103] since these typically have a plant wax component ranging from C_{19} to C_{31}/C_{33} and maximising at C_{21}/C_{23}, possibly deriving from plant waxes in the original food consumed in this context. The persistence of the *n*-alkanes is not unexpected given that they are known to be particularly resistant to diagenesis[90,92]. In addition to the data above, an *n*-alkane proxy for the input/presence of submerged/floating freshwater aquatic macrophytes can provide another corroborating measure for an aquatic plant input[89]. However, the *n*-alkane proxy ratio, P_{aq}, cannot be applied meaningfully here because of the lack of $>C_{23}$ n-alkanes. That being said, the C_{21} maximum, combined with the relatively narrow *n*-alkane carbon number range in the two calculus samples is indicative of biodegraded submerged aquatic plant (macrophyte) material.

Interestingly, the chemical profile of the dominant C_{19} to C_{23} *n*-alkanes, maximising at C_{21} is consistent with the submerged aquatic plant genus *Nymphaea*[89], following biodegradation. Further work corroborating this possibility using microscopy would be needed, but it certainly be worthy of further investigation. With a carbon preference indexes (CPIs) of 1.10 and 1.20 and C_{21} maxima they are consistent with what would be expected for a (biodegraded) submerged aquatic plant. Notably similar values have been observed for submerged aquatic plant rhizomes from southern Europe: Greece, CPI = 1.13; France, CPI = 1.15[101]. Furthermore, the rhizomes in these studies had a far less pronounced odd-over-even carbon preference than leaves from the same plants, which is likely to reflect their microenvironment in the aquatic/marine sediments where they are far more exposed to biomolecular reworking and biodegradation from microbial inputs than leaves from the same plant. In this regard it is notable that the *n*-alkanes in the two dental calculus samples also display this unimodal ‘hump’; this could be indicative of the consumption of rhizomes from a submerged aquatic plant by these individuals, although it may also be explained by post-depositional diagenetic factors.

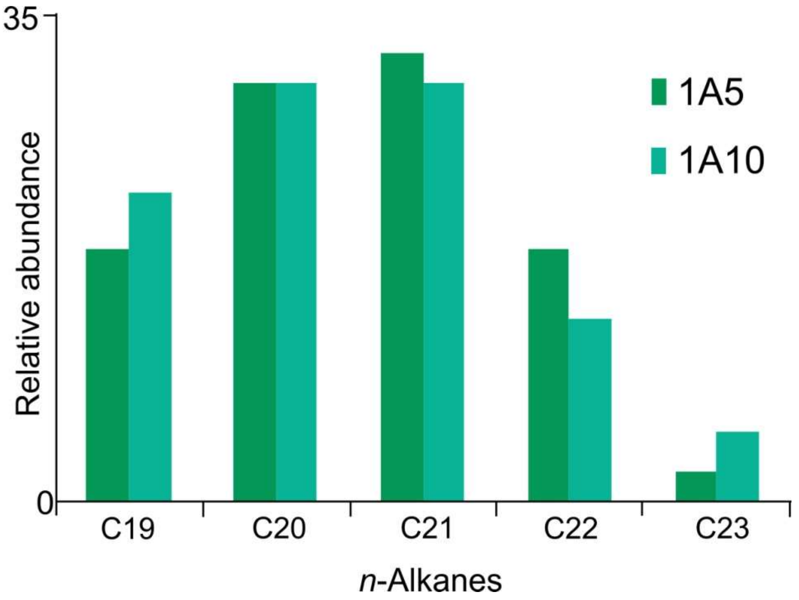

Supplementary Figure 29. *n*-Alkane distribution of two calculus samples from Obeliai, Lithuania: 1A5 male and 1A10 female. Source data are provided as a Source Data file.

The pyrolysis-GC-MS for 1A5 revealed a series of C_6 to C_{16} *n*-1-alkenes and *n*-alkanes, typical of unsaturated and saturated fat/oil-derived acyl lipids[5,6]. The absence of these compounds in the TD profile provides molecular evidence for a polymeric/bound acyl lipid source. However, the additional presence of a series of C_9 to C_{16} isoalkenes and isoalkanes, presumably deriving from the branched fatty acids, which are significant components of bacterial triglycerides, suggests a microbial origin for these lipid biomarkers, at least in part, and is consistent with the bacterial C_{17} and C_{18} *n*-alkanes observed in the TD. There were components in the pyrolysate which can be indicative of 'black carbon', i.e. charcoal or soot[7-10]. However, the dominance of toluene, rather than the normally dominant aromatic hydrocarbon benzene, is notably atypical of chars[7-10]. That being said, despite the absence of higher molecular weight 'char' organic compounds their collective presence provides at least tentative molecular evidence for exposure to fire/cooking. In contrast to 1A5, the pyrolysis-GC-MS for 1A10 revealed only C_4 to C_6 short chain alkenes, cyclopentadiene and its methyl-derivative and toluene. In the absence of more diagnostic biomolecules their significance here is uncertain. The dominance of carbon dioxide in both samples suggests highly oxidised organic material.

Another sample, 1A6, revealed only carbon dioxide in the TD-GC-MS analysis, indicating the essential absence of free, thermally extractable organic components in this sample. The carbon dioxide is also indicative of highly oxidised organic material. The pyrolysis-GC-MS revealed a series of C_7 to C_{16} *n*-1-alkenes and *n*-alkanes, typical of unsaturated and saturated fat/oil-derived acyl lipids[5,6]. The absence of these compounds in the TD profile provides molecular evidence for a polymeric/bound acyl lipid source. However, the additional presence of a series of C_8 to C_{16} isoalkenes and isoalkanes, presumably deriving from the branched fatty acids, which are significant components of bacterial triglycerides, suggests a microbial origin for these lipid biomarkers, at least in part (also see below). The dominance of carbon dioxide again suggests a highly oxidised sample.

The last two Obeliai samples, 1A8 and 1A9, revealed both free and bound/polymeric organic material. The thermal desorption total ion chromatogram (TIC) of the two samples were dominated by carbon dioxide; it is difficult to explain the significance of this non-diagnostic compound, beyond indicating that this sample has undergone diagenetic changes of a highly oxidising nature. Although only trace constituents, both samples revealed a series of *n*-alkanes, ranging from C_{15}/C_{17} up to C_{22}. Sample 1A8 maximises at C_{19}, and 1A9 maximises at C_{17}. Both samples show an odd over even carbon number predominance, although the relative abundance of the C_{16}, C_{18} and C_{20} *n*-alkanes suggests the samples have undergone some biodegradation as a result of diagenetic changes. Although the trace quantities and evidence for biodegradation necessarily mean any inferences must be tentative, the narrow range (C_{15}/C_{17} and C_{22}) and relatively unusual mid-chain carbon numbers, maximising at C_{17} or C_{19}, combined with an odd over even predominance, is characteristic of an algal source[92,101,103]. The presence of branched alkanes with carbon numbers C_{15} to C_{21} observed as relatively minor constituents is indicative of some microbially-derived biodegradation.

The pyrolysis-GC-MS analyses of these two samples revealed moderate amounts of organic material which were dominated by a complex suite of organic compounds indicative of lipid (fat/oil) and protein components. These include a series of C_7 to C_{20} *n*-1-alkenes and *n*-alkanes, typical of unsaturated and saturated fat/oil-derived acyl lipids[5,6]. A maxima C_{13}/C_{14} combined with abundant short chain (C_4-C_7) *n*-1-alkenes suggests the original acyl lipids in these samples are likely to have been high in polyunsaturated fatty acids. The absence of these compounds in the TD profile provides molecular evidence for a polymeric/bound acyl lipid source. However, in addition to *n*-1-alkenes and *n*-alkanes, typical of fat/oil derived lipids, the pyrograms also displayed a series of C_8/C_9 to C_{16} iso-1-alkenes and isoalkanes,

presumably deriving from the branched fatty acids which are significant components of bacterial triglycerides (but see below).

There were components in the pyrolysate of both samples which can be indicative of 'black carbon', i.e. charcoal or soot[7-10]. However, the dominance of toluene, rather than the normally dominant aromatic hydrocarbon benzene and the absence of naphthalenes and biphenyls, is notably atypical of chars[7-10]. The markers identified here can also be indicative of proteins[6,11] and combined with the abundant related protein markers pyrrole, benzonitrile, benzyl nitrile (phenylacetonitrile), benzenepropanenitrile and indoles[6,11] identified in sample 1A9 this is likely to be the main origin of these compounds, pointing to a substantial protein-derived component[6,11] in this sample. This is also corroborated by the presence of aliphatic nitriles (carbon numbers $C_{14:0}$,$C_{16:0}$ (max) and $C_{18:0}$) in both samples, suggesting a lipid (fat/oil) in this sample which has combined with protein-derived nitrogen to produce these moieties[5]. Taken together, the presence of toluene, styrene and phenylacetonitrile (benzyl nitrile) can indicate the amino acid phenylalanine[12,13]. In addition, the significant constituents indole and 3-methylindole (skatole), with a lesser amount of a C_2 indole (3-ethylindole?), in sample 1A9 are consistent with a tryptophan amino acid source[12,13,19,20]. Proteins rich in the amino acids phenylalanine and tryptophan include meat, fish and dairy products, although the presence of bacterial markers means the possibility that at least a partial origin could be microbial cannot be excluded.

Far more diagnostic of a particular dietary source are the particular suite of alkyl pyrroles observed in the pyrograms of these samples, with an alkyl chain length of up to C_6. Both these samples revealed significant amounts of these biomarkers. This included the 2- and 3-dimethylpyrroles, 2- and 3-ethylpyrroles, 2,4-, 2,3- and 3,4-dimethylpyrroles, four ethylmethylpyrroles, a trimethylpyrrole, two propylmethylpyrroles, six ethyldimethylpyrroles, four diethylpyrroles, 2,3,4,5-tetramethylpyrrole, three diethylmethylpyrroles, an ethyltrimethylpyrrole and an unidentified C_6 ethylpyrrole. These specific C_1 to C_6 alkyl pyrroles are highly diagnostic of the presence of a significant tetrapyrrole component. Their porphyrin-derived origin, and the relative abundance of some of the key biomarkers, is indicative of an algal source observed in fossils and expected in similarly diagenetically altered archaeological material[11,21,22]. Although C_1 and C_2 alkyl pyrroles can also derive from protein-derived amino acids such as glycine, proline, hydroxyproline, serine and glutamic acid[12-14,20,40,41], the C_3+ alkyl pyrroles identified in these two samples point to a tetrapyrrole source[21,22,40,41]. Moreover, the same specific C_1 to C_5

alkylpyrroles identified in the Py-GC-MS of an algal kerogen[21,22] were observed in these samples. Overall the two samples display a remarkably similar pyrolysis profile to algal-derived kerogen previously studied via very similar organic geochemical techniques[21,22].

Notable also in this context were pyridine and its methyl-, and dimethyl derivatives (these pyridines can result from the pyrolysis of polypeptides containing the amino acid alanine[25]). The presence here of the abundant protein markers and specifically the C_1 to C_6 alkyl pyrroles, combined with the particular lipid profile and specific carbohydrate markers (see above) identified, indicate the presence of a macroalgae, i.e. seaweed or freshwater algal source, in this sample[21-23]. Marine and freshwater algal sources are known to have been exploited for food in antiquity[52,104], although their consumption in this part of the world does not seem to have been seriously considered to date.

Lithuania People's Wandering (AD 5th-6th century), Pagrybis, North Lithuania barrows

Pagrybis burial ground represents a small hill located in the Pagrybis village near Akmena river (Šilalė region) in western Lithuania. During investigations carried in 1980-1982, 217 graves were uncovered containing 224 individuals. Adults and children were buried in grave pits in wooden coffins. Most graves were single, though some double graves were found. All had been buried on their backs with straight legs. Burial items include all kinds of tools, weapons, fragments of cloths and jewellery. 38 graves contained remains of sacrified horses[105].

41. Lithuania People's Wandering (AD 5th-6th century), Pagrybis, North Lithuania barrows, Sk1410A female 40-45 yrs T43 labial, 1A17 (calculus: 4.11 mg)

The thermal desorption total ion chromatogram (TIC) revealed no detectable components, indicating the absence of free, thermally extractable organic components in this sample. It should be noted, however, that highly polar material could have been present, which would not have successfully eluted from the column, or indeed volatilised sufficiently in the probe.

The pyrolysate TIC revealed no detectable components, indicating the essential absence of organic material in this sample. It should be noted, however, that highly polar material could have been present, which would not have successfully eluted from the column, or indeed volatilised sufficiently in the probe.

42. Lithuania People's Wandering (AD 5th-6th century), Pagrybis, North Lithuania barrows, Sk1409C male 30-35 yrs T31 labial, 1A18 (calculus: 4.14 mg)

The thermal desorption total ion chromatogram (TIC) revealed carbon dioxide as the only detectable component, indicating the essential absence of free, thermally extractable organic components in this sample. It should be noted, however, that highly polar material could have been present, which would not have successfully eluted from the column, or indeed volatilised sufficiently in the probe. It is not possible to determine the significance of the carbon dioxide, beyond it being indicative of oxidised organic material.

The pyrolysis-GC-MS TIC was dominated by carbon dioxide, 1-butene, 1-pentene and hexene, the alkenes being indicative of lipid or proteinaceous material in the sample[11]. Cyclopentadiene and methylcyclopentadiene are also significant components of the pyrolysate although it is not possible to ascribe a specific origin here. Toluene was also detected, albeit as a very minor component, preventing any meaningful interpretation of its presence, beyond it being indicative of an organic origin. The dominance of carbon dioxide suggests a highly oxidised sample. It is not possible to determine the significance of the carbon dioxide, beyond it being indicative of oxidised organic material. It should be noted, however, that highly polar material could have been present, which would not have successfully eluted from the column, or indeed volatilised sufficiently in the probe.

43. Lithuania People's Wandering (AD 5th-6th century), Pagrybis, North Lithuania barrows, Sk1402 female 45-50 yrs T42 labial, 1A19 (calculus: 7.22 mg)

The thermal desorption total ion chromatogram (TIC) revealed carbon dioxide and 1-butene as the only detectable components, indicating the essential absence of free, thermally extractable organic components in this sample. It should be noted, however, that highly polar material could have been present, which would not have successfully eluted from the column, or indeed volatilised sufficiently in the probe. The 1-butene possibly derives from lipid or proteinaceous material in the sample[11], and perhaps produced at this relatively low temperature due to matrix effects. It is not possible to determine the significance of the carbon dioxide, beyond it being indicative of oxidised organic material.

The pyrolysis-GC-MS TIC (Fig. 30) was dominated by carbon dioxide, 1-butene and 1-pentene, the alkenes being indicative of lipid or proteinaceous material in the sample[11]. Cyclopentadiene and methylcyclopentadiene are also components of the pyrolysate although it is not possible to ascribe a specific origin here. Benzene, toluene and pyridine were also detected, albeit as minor components; their significance in the absence of other more diagnostic biomolecules is unclear. In contrast to the thermal desorption profile, the pyrogram also revealed a series of C_8 to C_{16} *n*-1-alkenes and *n*-alkanes, typical of unsaturated and saturated fat/oil-derived acyl lipids[5,6]. The absence of these compounds in the TD profile provides molecular evidence for a polymeric/bound acyl lipid source. However, in addition to *n*-1-alkenes and *n*-alkanes, typical of fat/oil derived lipids, the pyrogram also displayed a series of C_9 to C_{15} iso-methyl-1-alkenes (isoalkenes) and 2-methylalkanes (isoalkanes), presumably deriving from the branched fatty acids which are significant components of bacterial triglycerides. The dominance of carbon dioxide suggests a highly oxidised sample. It is not possible to determine the significance of the carbon dioxide, beyond it being indicative of oxidised organic material. It should be noted, however, that highly polar material could have been present, which would not have successfully eluted from the column, or indeed volatilised sufficiently in the probe.

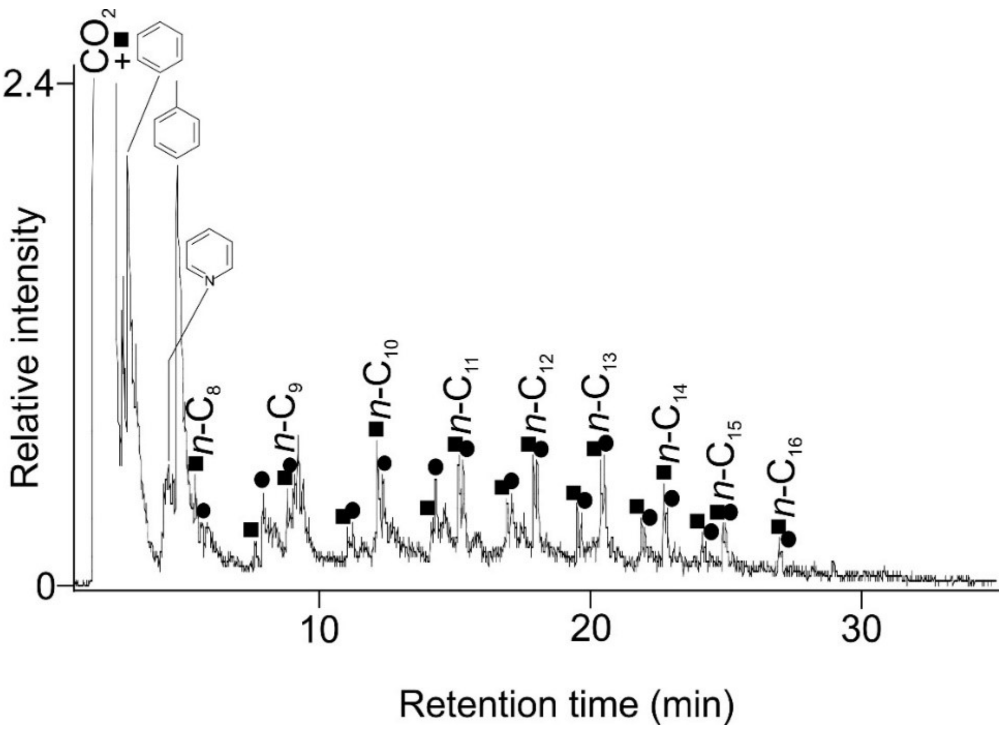

Supplementary Figure 30. Reconstructed total ion chromatogram of the pyrogram (pyrolysis profile) (610 °C for 10s) of sample 1A19, People's Wandering (AD 5th-6th century), Pagrybis, after thermal desorption (310 °C for 10 s). Key to Fig. 30: Peak identities (x indicates carbon chain length): filled squares, Cx indicates alkenes; filled circles, Cx indicates alkanes. Also shown are the structures of three aromatic compounds identified: benzene, toluene and pyridine. CO_2 indicates carbon dioxide.

44. Lithuania People's Wandering (AD 5th-6th century), Pagrybis, North Lithuania barrows, Sk1398 male >55 yrs T27 bucal, 1A20 (calculus: 3.83 mg)

The thermal desorption total ion chromatogram (TIC) revealed carbon dioxide as the only detectable component, indicating the essential absence of free, thermally extractable organic components in this sample. It should be noted, however, that highly polar material could have been present, which would not have successfully eluted from the column, or indeed volatilised sufficiently in the probe. It is not possible to determine the significance of the carbon dioxide, beyond it being indicative of oxidised organic material.

The pyrolysis-GC-MS TIC (Fig. 31) was dominated by carbon dioxide, 1-butene, 1-pentene and 1-hexene, the alkenes being indicative of lipid or proteinaceous material in the sample[11]. Cyclopentadiene and methylcyclopentadiene are also components of the pyrolysate although it is not possible to ascribe a specific origin here. Benzene, toluene and pyridine were also detected, albeit as very minor components; their significance in the absence of other more diagnostic biomolecules is unclear. In contrast to the thermal desorption profile, the pyrogram also revealed trace amounts of a series of C_9 to C_{15} *n*-1-alkenes and *n*-alkanes, typical of unsaturated and saturated fat/oil-derived acyl lipids[5,6]. The absence of these compounds in the TD profile provides molecular evidence for a polymeric/bound acyl lipid source. However, in addition to *n*-1-alkenes and *n*-alkanes, typical of fat/oil derived lipids, the pyrogram also displayed a series of C_{11} to C_{15} branched alkenes and alkanes, presumably deriving from branched fatty acids which are significant components of bacterial triglycerides. The dominance of carbon dioxide suggests a highly oxidised sample. It is not possible to determine the significance of the carbon dioxide, beyond it being indicative of oxidised organic material. It should be noted, however, that highly polar material could have been present, which would not have successfully eluted from the column, or indeed volatilised sufficiently in the probe.

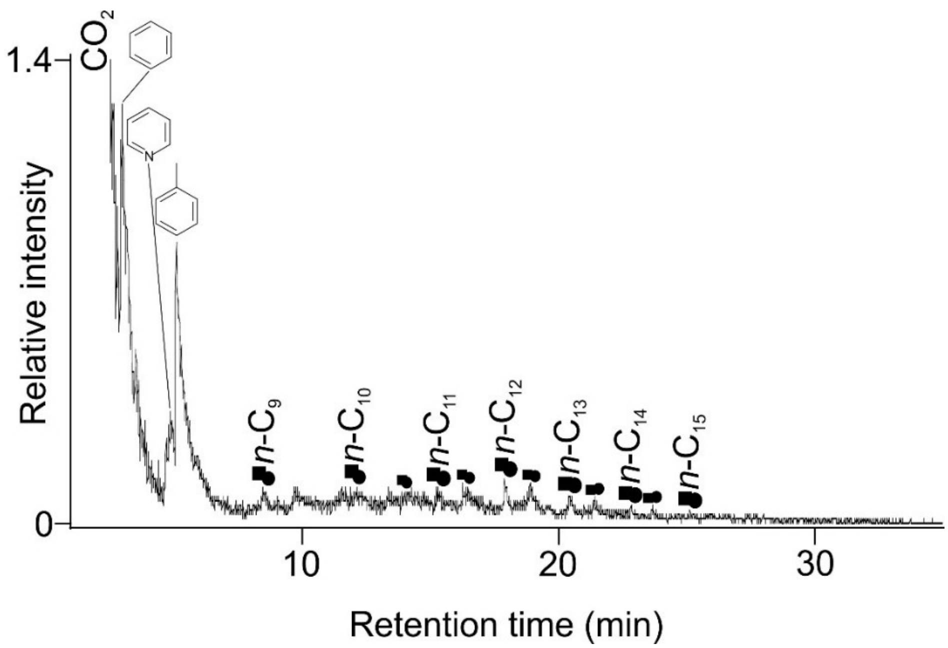

Supplementary Figure 31. Reconstructed total ion chromatogram of the pyrogram (pyrolysis profile) (610 °C for 10s) of sample 1A20, People's Wandering (AD 5th-6th century), Pagrybis, after thermal desorption (310 °C for 10 s). Key to Fig. 31: Peak identities (x indicates carbon chain length): filled squares, Cx indicates alkenes; filled circles, Cx indicates alkanes. Also shown are the structures of three aromatic compounds identified: benzene, toluene and pyridine. CO_2 indicates carbon dioxide.

45. Lithuania People's Wandering (AD 5th-6th century), Pagrybis, North Lithuania barrows, Sk1058 male 30-35 yrs T41 labial, 1A21 (calculus: 4.46 mg)

The thermal desorption total ion chromatogram (TIC) revealed carbon dioxide as the only detectable component, indicating the essential absence of free, thermally extractable organic components in this sample. It should be noted, however, that highly polar material could have been present, which would not have successfully eluted from the column, or indeed volatilised sufficiently in the probe. It is not possible to determine the significance of the carbon dioxide, beyond it being indicative of oxidised organic material.

The pyrolysis-GC-MS TIC (Fig. 32) was dominated by carbon dioxide, 1-butene, 1-pentene and 1-hexene, the alkenes being indicative of lipid or proteinaceous material in the sample[11]. Cyclopentadiene and methylcyclopentadiene are also components of the pyrolysate although

it is not possible to ascribe a specific origin here. Benzene, toluene and pyridine were also detected, albeit as very minor components; their significance in the absence of other more diagnostic biomolecules is unclear. In contrast to the thermal desorption profile, the pyrogram also revealed trace amounts of a series of C_7 to C_{16} *n*-1-alkenes and *n*-alkanes, typical of unsaturated and saturated fat/oil-derived acyl lipids[5,6]. The absence of these compounds in the TD profile provides molecular evidence for a polymeric/bound acyl lipid source. However, in addition to *n*-1-alkenes and *n*-alkanes, typical of fat/oil derived lipids, the pyrogram also displayed a series of C_{10} to C_{16} iso-methyl-1-alkenes (isoalkenes) and 2-methylalkanes (isoalkanes), presumably deriving from the branched fatty acids which are significant components of bacterial triglycerides. The dominance of carbon dioxide suggests a highly oxidised sample. It is not possible to determine the significance of the carbon dioxide, beyond it being indicative of oxidised organic material. It should be noted, however, that highly polar material could have been present, which would not have successfully eluted from the column, or indeed volatilised sufficiently in the probe.

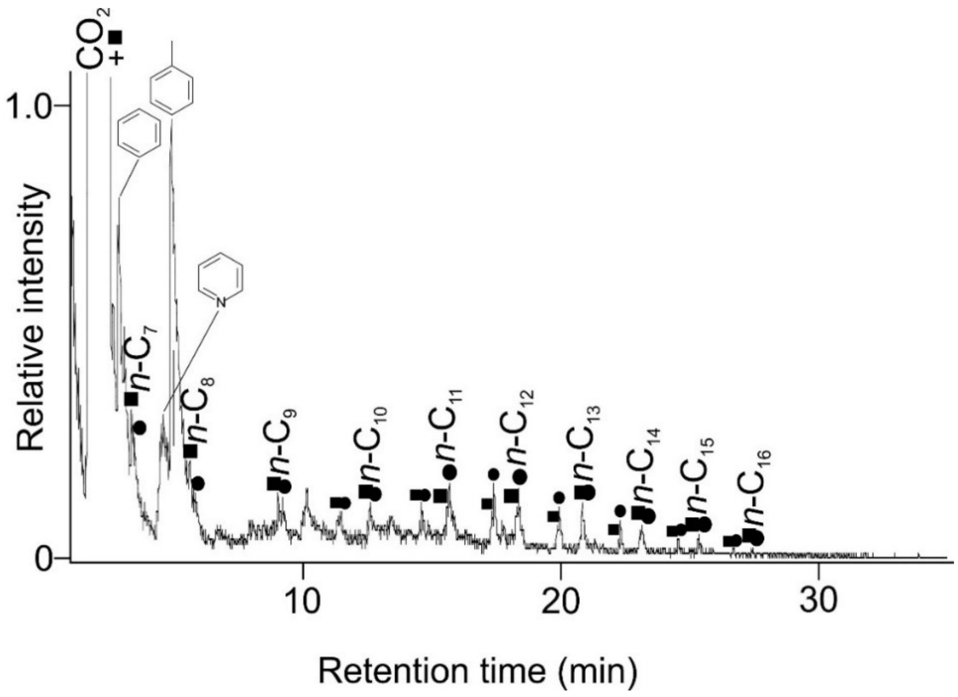

Supplementary Figure 32. Reconstructed total ion chromatogram of the pyrogram (pyrolysis profile) (610 °C for 10s) of sample 1A21, People's Wandering (AD 5th-6th century), Pagrybis, after thermal desorption (310 °C for 10 s). Key to Fig. 32: Peak identities (x indicates carbon chain length): filled squares, Cx indicates alkenes; filled circles, Cx

indicates alkanes. Also shown are the structures of three aromatic compounds identified: benzene, toluene and pyridine. CO_2 indicates carbon dioxide.

Discussion, Lithuania People's Wandering (AD 5th-6th century), Pagrybis, North Lithuania barrows

Five samples of dental calculus from individual burials at Pagrybis dating to the People's Wandering (AD 5th–6th century) were analysed by sequential thermal desorption-gas chromatography-mass spectrometry (TD-GC-MS) and pyrolysis-gas chromatography-mass spectrometry (Py-GC-MS).

One sample (1A17) contained no detectable free or bound/polymeric organic material, most likely reflecting unfavourable prevailing conditions for biomolecular preservation. A second sample (1A18) contained little free and bound/polymeric organic material, again most likely reflecting unfavourable prevailing conditions for biomolecular preservation. Of those organic compounds identified, the dominance of carbon dioxide and the relatively minor abundance of only a few small organic compounds suggests that only highly oxidised organic material was present.

The three remaining samples (1A19, 1A20 and 1A21) showed a notably similar biomolecular fingerprint for the sequential analyses. The TD-GC-MS analyses revealed only carbon dioxide indicating the essential absence of free, thermally extractable organic components in these samples. The carbon dioxide is also indicative of highly oxidised organic material. The pyrolysis-GC-MS revealed a series of C_7/C_8 to C_{16} *n*-1-alkenes and *n*-alkanes, typical of unsaturated and saturated fat/oil-derived acyl lipids[5,6]. The absence of these compounds in the TD profiles provides molecular evidence for a polymeric/bound acyl lipid source. However, the additional presence of a series of C_8 to C_{15}/C_{16} isoalkenes and isoalkanes, presumably deriving from the branched fatty acids, which are significant components of bacterial triglycerides, strongly suggests a microbial origin for these lipid biomarkers, at least in part. The dominance of carbon dioxide again suggests a highly oxidised sample.

Lithuania People's Wandering (AD 5th-6th century), Vėluikiai, North Lithuania barrows

Vėluikiai cemetery is situated on the shore of Ančia river (Tauragė district) in western Lithuania. Investigations carried in 2006 and 2020 revealed that a part of a cemetery was

destroyed by floods. In total, 5 inhumations were found. They had all been buried on their backs, with their arms in various positions and their legs straight. The burial pits had rounded ends. Artefacts recovered at the Vėluikiai cemetery suggest it belongs to the final stage of the Old (Roman) Iron Age and the beginning of the early Middle Ages[106].

46. Lithuania People's Wandering (AD 5th-6th century), Vėluikiai, North Lithuania barrows, Sk1421 female 25-30 yrs T46 lingual, 1A28 (calculus: 2.33 mg)

The thermal desorption total ion chromatogram (TIC) revealed carbon dioxide as the only detectable component, indicating the essential absence of free, thermally extractable organic components in this sample. It should be noted, however, that highly polar material could have been present, which would not have successfully eluted from the column, or indeed volatilised sufficiently in the probe. It is not possible to determine the significance of the carbon dioxide, beyond it being indicative of oxidised organic material.

The pyrolysate TIC revealed carbon dioxide, 1-butene, 1-pentene, and cyclopentadiene as the only detectable components. The dominance of carbon dioxide, combined with the relatively minor abundance of the few small organic compounds present, suggests a highly oxidised sample. It is not possible to determine the significance of the carbon dioxide, beyond it being indicative of oxidised organic material and the other compounds are too low in abundance and too ubiquitous to provide any meaningful interpretation of their presence, beyond them being indicative of an organic origin. It should be noted, however, that highly polar material could have been present, which would not have successfully eluted from the column, or indeed volatilised sufficiently in the probe.

Discussion, Lithuania People's Wandering (AD 5th-6th century), Vėluikiai, North Lithuania barrows

One sample of dental calculus from an individual burial at Vėluikiai dating to the People's Wandering (AD 5th–6th century) was analysed by sequential thermal desorption-gas chromatography-mass spectrometry (TD-GC-MS) and pyrolysis-gas chromatography-mass spectrometry (Py-GC-MS). The sample contained little free and bound/polymeric organic material, most likely reflecting unfavourable prevailing conditions for biomolecular preservation. Of those organic compounds identified, the dominance of carbon dioxide and

the relatively minor abundance of only a few small organic compounds suggests that only highly oxidised organic material was present.

Lithuania Roman period (AD 1st-3th century), Pašatrijis, North Lithuania barrows

The Pašatrija hillfort and the settlement at its foot is situated in Telšiai district in western Lithuania. Excavations carried in 2012 uncovered an area totalling 1320 m^2. Materials including pottery, iron smelting remnants, slag, animal bones, as well as metal ornaments and tools were also found. The complex was dated to the 1st/4th – late 14th / early 15th centuries AD. The earliest inhumations discovered belonged to nine individuals buried in five graves: three single graves, one double grave with two adult individuals, and one grave which contained four individuals (two adult males and two children). Most of the skeletons were in bent position[107].

47. Lithuania Roman period (AD 1st-3th century), Pašatrijis, North Lithuania barrows, Sk7224 male 40-45 yrs T27 bucal, 1A29 (calculus: 3.47 mg)

The thermal desorption total ion chromatogram (TIC) revealed carbon dioxide as the only detectable component, indicating the essential absence of free, thermally extractable organic components in this sample. It should be noted, however, that highly polar material could have been present, which would not have successfully eluted from the column, or indeed volatilised sufficiently in the probe. It is not possible to determine the significance of the carbon dioxide, beyond it being indicative of oxidised organic material.

The pyrolysis-GC-MS TIC (Fig. 33) was dominated by carbon dioxide, 1-butene, 1-pentene and 1-hexene, the alkenes being indicative of lipid or proteinaceous material in the sample[11]. Cyclopentadiene and methylcyclopentadiene are also components of the pyrolysate although it is not possible to ascribe a specific origin here. Benzene, toluene and pyridine were also detected, albeit as very minor components; their significance in the absence of other more diagnostic biomolecules is unclear. In contrast to the thermal desorption profile, the pyrogram also revealed trace amounts of a series of C_9 to C_{16} *n*-1-alkenes and *n*-alkanes, typical of unsaturated and saturated fat/oil-derived acyl lipids[5,6]. The absence of these compounds in the TD profile provides molecular evidence for a polymeric/bound acyl lipid source. However, in addition to *n*-1-alkenes and *n*-alkanes, typical of fat/oil derived lipids, the pyrogram also displayed a series of C_{11} to C_{16} iso-methyl-1-alkenes (isoalkenes) and 2-methylalkanes

(isoalkanes), presumably deriving from the branched fatty acids which are significant components of bacterial triglycerides. The dominance of carbon dioxide suggests a highly oxidised sample. It is not possible to determine the significance of the carbon dioxide, beyond it being indicative of oxidised organic material. It should be noted, however, that highly polar material could have been present, which would not have successfully eluted from the column, or indeed volatilised sufficiently in the probe.

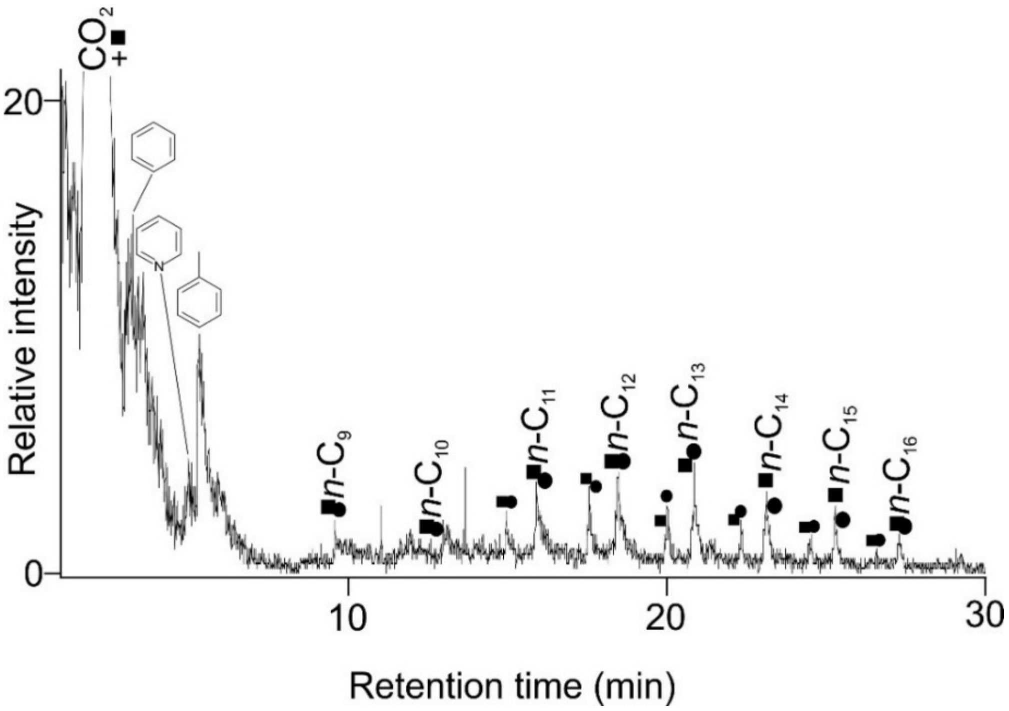

Supplementary Figure 33. Reconstructed total ion chromatogram of the pyrogram (pyrolysis profile) (610 °C for 10s) of sample 1A29, Roman period (AD 1st-3th century), Pašatrijis, after thermal desorption (310 °C for 10 s). Key to Fig. 33: Peak identities (x indicates carbon chain length): filled squares, Cx indicates alkenes; filled circles, Cx indicates alkanes. Also shown are the structures of three aromatic compounds identified: benzene, toluene and pyridine. CO_2 indicates carbon dioxide.

48. Lithuania Roman period (AD 1st-3th century), Pašatrijis, North Lithuania barrows, Sk7227 male 20-25 yrs T32 labial, 1A30 (calculus: 2.67 mg)

The thermal desorption total ion chromatogram (TIC) revealed no detectable components, indicating the absence of free, thermally extractable organic components in this sample. It

should be noted, however, that highly polar material could have been present, which would not have successfully eluted from the column, or indeed volatilised sufficiently in the probe.

The pyrolysate TIC revealed carbon dioxide, 1-butene, 1-pentene, and 1-hexene as the only detectable components. The dominance of carbon dioxide, combined with the relatively minor abundance of the few small organic compounds present, suggests a highly oxidised sample. It is not possible to determine the significance of the carbon dioxide, beyond it being indicative of oxidised organic material and the other compounds are too low in abundance to provide any meaningful interpretation of their presence, beyond them being indicative of an organic origin. It should be noted, however, that highly polar material could have been present, which would not have successfully eluted from the column, or indeed volatilised sufficiently in the probe.

Discussion, Lithuania Roman period (AD 1st-3th century), Pašatrijis, North Lithuania barrows

Two samples of dental calculus from individual burials at Pašatrijis dating to the Roman period (AD 1st–4th century) were analysed by sequential thermal desorption-gas chromatography-mass spectrometry (TD-GC-MS) and pyrolysis-gas chromatography-mass spectrometry (Py-GC-MS).

One sample (1A30) contained virtually no free and bound/polymeric organic material, most likely reflecting unfavourable prevailing conditions for biomolecular preservation. Of those organic compounds identified, the dominance of carbon dioxide and the very minor abundance of only a very few small organic compounds suggests that only highly oxidised organic material was present. The other sample (1A29) revealed only carbon dioxide in the TD-GC-MS indicating the essential absence of free, thermally extractable organic components in these samples. The carbon dioxide is also indicative of highly oxidised organic material. The pyrolysis-GC-MS revealed a series of C_9 to C_{16} *n*-1-alkenes and *n*-alkanes, typical of unsaturated and saturated fat/oil-derived acyl lipids[5,6]. The absence of these compounds in the TD profiles provides molecular evidence for a polymeric/bound acyl lipid source. However, the additional presence of a series of C_{11} to C_{16} isoalkenes and isoalkanes, presumably deriving from the branched fatty acids, which are significant components of bacterial triglycerides, strongly suggests a microbial origin for these lipid biomarkers, at least in part. The dominance of carbon dioxide in the pyrogram again suggests a highly oxidised sample.

Lithuania Viking period (AD 7th-12th century), Jakštaičiai-Meškia, North Lithuania barrows

The Jakštaičiai-Meškiai cemetery is situated on the shore of Dubysa river, between villages Jakštaičiai and Meškiai (Šiauliai district) in northern Lithuania. Investigations carried in 1973-1983 revealed 102 inhumations, 9 cremated burials and 1 horse grave. Preservation of skeletons was poor. Some graves had been destroyed through ploughing. Burial items include various kinds of tools, weapons, fragments of cloths and adornments[102].

49. Lithuania Viking period (AD 7th-12th century), Jakštaičiai-Meškia, North Lithuania barrows, Sk1388B male 20-25 yrs T36 lingual, 1A31 (calculus: 4.33 mg)

The thermal desorption total ion chromatogram (TIC) revealed carbon dioxide as the only detectable component, indicating the essential absence of free, thermally extractable organic components in this sample. It should be noted, however, that highly polar material could have been present, which would not have successfully eluted from the column, or indeed volatilised sufficiently in the probe. It is not possible to determine the significance of the carbon dioxide, beyond it being indicative of oxidised organic material.

The pyrolysis-GC-MS TIC (Fig. 34) was dominated by carbon dioxide, 1-butene, 1-pentene and 1-hexene, the alkenes being indicative of lipid or proteinaceous material in the sample[11]. Cyclopentadiene and methylcyclopentadiene are also components of the pyrolysate although it is not possible to ascribe a specific origin here. Acetone was also identified and can be indicative of the amino acid glycine or a carbohydrate component, although any interpretation should be considered tentative. Benzene, toluene and pyridine were also detected, albeit as minor components; their significance in the absence of other more diagnostic biomolecules is unclear. In contrast to the thermal desorption profile, the pyrogram also revealed trace amounts of a series of C_7 to C_{17} *n*-1-alkenes and *n*-alkanes, typical of unsaturated and saturated fat/oil-derived acyl lipids[5,6]. The absence of these compounds in the TD profile provides molecular evidence for a polymeric/bound acyl lipid source. However, in addition to *n*-1-alkenes and *n*-alkanes, typical of fat/oil derived lipids, the pyrogram also displayed a series of C_8 to C_{16} iso-methyl-1-alkenes (isoalkenes) and 2-methylalkanes (isoalkanes), presumably deriving from the branched fatty acids which are significant components of bacterial triglycerides. The dominance of carbon dioxide suggests a highly

oxidised sample. It is not possible to determine the significance of the carbon dioxide, beyond it being indicative of oxidised organic material. It should be noted, however, that highly polar material could have been present, which would not have successfully eluted from the column, or indeed volatilised sufficiently in the probe.

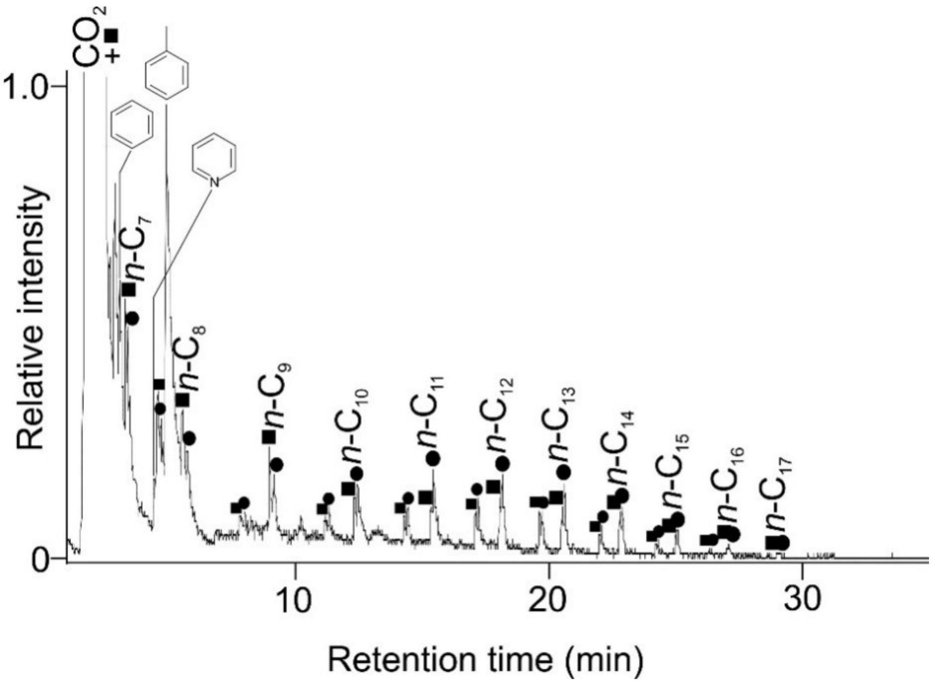

Supplementary Figure 34. Reconstructed total ion chromatogram of the pyrogram (pyrolysis profile) (610 °C for 10s) of sample 1A31, Viking period AD 7th-12th century), Jakštaičiai-Meškia, after thermal desorption (310 °C for 10 s). Key to Fig. 34: Peak identities (x indicates carbon chain length): filled squares, Cx indicates alkenes; filled circles, Cx indicates alkanes. Also shown are the structures of three aromatic compounds identified: benzene, toluene and pyridine. CO_2 indicates carbon dioxide.

50. Lithuania Viking period (AD 7th-12th century), Jakštaičiai-Meškia, North Lithuania barrows, Sk1386 female 20-25 yrs T46 lingual, 1A32 (calculus: 8.47 mg)

The thermal desorption total ion chromatogram (TIC) (Fig. 35, inset) revealed carbon dioxide as the main detectable (trace) component, indicating very little free, thermally extractable organic components in this sample. However, also in trace abundance are a series of *n*-alkanes, carbon numbers C_{15} to C_{21}, maximising at C_{17}. There is a slight odd over even

carbon number predominance, although the relative abundance of the C_{18} and C_{20} *n*-alkanes suggests the sample has undergone some biodegradation as a result of diagenetic changes. Although the trace quantities and evidence for biodegradation necessarily mean any inference must be tentative, the relatively unusual mid-chain carbon numbers present in the TD chromatogram, combined with the C_{17} *n*-alkane being dominant and a slight odd over even carbon number predominance, suggests an algal source[92,101,103] (see discussion below). It is not possible to determine the significance of the carbon dioxide, beyond it being indicative of oxidised organic material. It should also be note that highly polar material could have been present, which would not have successfully eluted from the column, or indeed volatilised sufficiently in the probe.

The pyrolysis-GC-MS TIC (Fig. 35) was dominated by carbon dioxide, 1-butene, 1-pentene and 1-hexene, the alkenes being indicative of lipid or proteinaceous material in the sample[11]. Cyclopentadiene and methylcyclopentadiene are also components of the pyrolysate although it is not possible to ascribe a specific origin here. Acetone was also identified and can be indicative of the amino acid glycine or a carbohydrate component, although any interpretation should be considered tentative. In contrast to the thermal desorption profile, the pyrogram also revealed a series of C_7 to C_{20} *n*-1-alkenes and *n*-alkanes, typical of unsaturated and saturated fat/oil-derived acyl lipids[5,6]. A distribution with maxima at C_{11} and C_{13}/C_{14} suggests the original acyl lipids may have been high in polyunsaturated fatty acids. The absence of these compounds in the TD profile provides molecular evidence for a polymeric/bound acyl lipid source. However, in addition to *n*-1-alkenes and *n*-alkanes, typical of fat/oil derived lipids, the pyrogram also displayed a series of C_8 to C_{16} iso-methyl-1-alkenes (isoalkenes) and 2-methylalkanes (isoalkanes), presumably deriving from the branched fatty acids which are significant components of bacterial triglycerides.

A number of the biomarkers identified here can be indicative of proteins[6,11], i.e. benzene, toluene, pyridine, pyrrole. This is also corroborated by the presence of aliphatic nitriles (carbon numbers $C_{14:0}$, *i*-$C_{15:0}$, $C_{16:0}$ (max) and $C_{18:0}$) suggesting a lipid (fat/oil) in this sample which has combined with protein-derived nitrogen to produce these moieties[5]. However, notable in this context is the additional presence of appreciable amounts of the more unusual alkyl pyrroles. C_1 alkyl pyrroles: 2- and 3-methylpyrroles; C_2 alkyl pyrroles: 2- and 3-ethylpyrroles, 2,4-, 2,3- and 3,4-dimethyl-pyrroles; C_3 alkyl pyrroles: 2-ethyl-4(?)-methylpyrrole, 2-ethyl-3(?)-methylpyrrole and 4-ethyl-2-methylpyrrole; C_4 alkyl pyrrole: an unidentified propylmethylpyrrole; C_5 alkyl pyrrole: 2-ethyl-5?-propylpyrrole. Collectively,

these specific C_1, C_2, C_3, C_4 and C_5 alkyl pyrroles are characteristic of a tetrapyrrole/porphyrin-derived origin and are indicative of an algal source observed in fossils and expected in similarly diagenetically altered archaeological material[11,21,22]. The presence here of protein markers and specifically the C_1 to C_5 alkyl pyrroles, combined with the particular lipid profile identified, indicate the presence of a macroalgae, i.e. seaweed or freshwater source, in this sample[21-23] (see discussion). The dominance of carbon dioxide suggests a highly oxidised sample. It is not possible to determine the significance of the carbon dioxide, beyond it being indicative of oxidised organic material. It should be noted, however, that highly polar material could have been present, which would not have successfully eluted from the column, or indeed volatilised sufficiently in the probe.

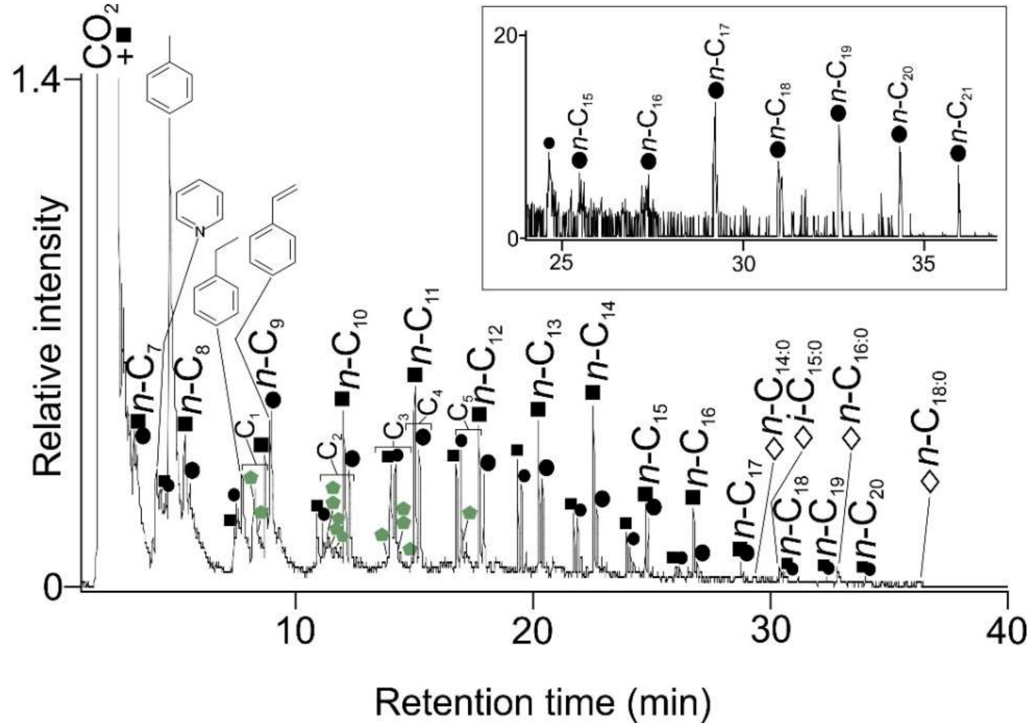

Supplementary Figure 35. Reconstructed total ion chromatogram of the pyrogram (pyrolysis profile) (610 °C for 10s) of sample 1A32, period (AD 7th-12th century), Jakštaičiai-Meškia, after thermal desorption (310 °C for 10 s). Key to Fig. 35: Peak identities (x indicates carbon chain length): filled squares, Cx indicates alkenes; filled circles, Cx indicates alkanes; open diamonds, Cx:y indicates acyclic nitriles; green filled pentagons indicates alkyl pyrroles (with alkyl chain length above, Cx). Also shown are the structures of four aromatic compounds identified: pyridine, toluene, ethylbenzene and styrene. CO_2 indicates carbon

dioxide. Inset displays a reconstructed total ion chromatogram of the thermal desorption profile (310 °C for 10 s) of this sample. Peak identities: (x indicates carbon chain length): filled circles, Cx indicates alkanes.

51. Lithuania Viking period (AD 7th-12th century), Jakštaičiai-Meškia, North Lithuania barrows, Sk1382 male 35-40 yrs T36 lingual, 1A33 (calculus: 11.05 mg)

The main compound in the thermal desorption total ion chromatogram (TIC) (Fig. 36, inset) is carbon dioxide. In very minor abundance are a series of *n*-alkanes, carbon numbers C_{16} to C_{22}, maximising at C_{19}. There is a slight odd over even carbon number predominance, although the relative abundance of the C_{18} and C_{20} *n*-alkanes and the presence of C_{15}, C_{17} and C_{19} to C_{22} branched chain alkanes suggests the sample has undergone some microbial-derived biodegradation as a result of diagenetic changes. Although the trace quantities and evidence for biodegradation necessarily mean any inference must be tentative, the relatively unusual mid-chain carbon numbers present in the TD chromatogram, combined with the C_{19} *n*-alkane being dominant and a slight odd over even carbon number predominance, suggests an algal source[92,101,103] (see discussion below). It is not possible to determine the significance of the carbon dioxide, beyond it being indicative of oxidised organic material. It should also be note that highly polar material could have been present, which would not have successfully eluted from the column, or indeed volatilised sufficiently in the probe.

The pyrolysis-GC-MS TIC (Fig. 36) was dominated by carbon dioxide, 1-butene, 1-pentene and 1-hexene, the alkenes being indicative of lipid or proteinaceous material in the sample[11]. Cyclopentadiene and methylcyclopentadiene are also components of the pyrolysate although it is not possible to ascribe a specific origin here. Acetone was also identified and can be indicative of the amino acid glycine or a carbohydrate component, although any interpretation should be considered tentative. In contrast to the thermal desorption profile, the pyrogram also revealed a series of C_7 to C_{18} *n*-1-alkenes and *n*-alkanes, typical of unsaturated and saturated fat/oil-derived acyl lipids[5,6]. A distribution with maxima at C_{11} and C_{13}/C_{14} suggests the original acyl lipids may have been high in polyunsaturated fatty acids. The absence of these compounds in the TD profile provides molecular evidence for a polymeric/bound acyl lipid source. However, in addition to *n*-1-alkenes and *n*-alkanes, typical of fat/oil derived lipids, the pyrogram also displayed a series of C_8 to C_{16} iso-methyl-1-

alkenes (isoalkenes) and 2-methylalkanes (isoalkanes), presumably deriving from the branched fatty acids which are significant components of bacterial triglycerides.

A number of the biomarkers identified here can be indicative of proteins[6,11], i.e. benzene, toluene, pyridine and pyrrole. This is also corroborated by the presence of aliphatic nitriles (carbon numbers $C_{14:0}$, *i*-$C_{15:0}$, *ai*-$C_{15:0}$, $C_{15:0}$, *i*-$C_{16:0}$, $C_{16:0}$ (max), *i*-$C_{17:0}$ and $C_{18:0}$) suggesting a lipid (fat/oil) in this sample which has combined with protein-derived nitrogen to produce these moieties[5]. However, notable in this context is the additional presence of appreciable amounts of the more unusual alkyl pyrroles. C_1 alkyl pyrroles: 2- and 3-methylpyrroles; C_2 alkyl pyrroles: 2- and 3-ethylpyrroles, 2,4-, 2,3- and 3,4-dimethyl-pyrroles; C_3 alkyl pyrroles: 2-ethyl-4(?)-methylpyrrole, 2-ethyl-3(?)-methylpyrrole and 4-ethyl-2-methylpyrrole; C_4 alkyl pyrrole: an unidentified propylmethylpyrrole; C_5 alkyl pyrrole: 2-ethyl-5?-propylpyrrole. Collectively, these specific C_1, C_2, C_3, C_4 and C_5 alkyl pyrroles are characteristic of a tetrapyrrole/porphyrin-derived origin and are indicative of an algal source observed in fossils and expected in similarly diagenetically altered archaeological material[11,21,22]. The presence here of protein markers and specifically the C_1 to C_5 alkyl pyrroles, combined with the particular lipid profile and carbohydrate markers (see below) identified, indicate the presence of a macroalgae, i.e. seaweed or freshwater source, in this sample[21-23] (see discussion).

The thermally-derived carbohydrate markers 2-methylfuran and 2-methyl-2-cyclopenten-1-one were identified as moderately signiifcant components. That they were absent in the TD profile points to their originating from the original polymeric carbohydrate source (e.g. starch)[27]. Although levoglucosan (1,6-anhydro-β-D-glucopyranose) is usually the major component in the thermally-derived products of carbohydrates analysed by TD/Py-GC-MS, its absence from the TD profile here is consistent with the removal of hydroxyl groups in the original carbohydrate polymer, so preventing its formation in the probe. The additional absence of the normally significant carbohydrate markers 2-hydroxy-3-methyl-2-cyclopenten-1-one and 3-hydroxy-2-methyl-2-cyclopenten-1-one observed in pure standard material (glycogen, starch and cellulose) would also support this interpretation. This shows a strong matrix effect likely to be due to the inorganic component of the calculus and the microflora in the mouth. This dehydration/dehydroxylation has also been observed in dog calculus and 19th century human calculus[6], where other markers, e.g. a prevalence of nitriles, with amides very minor, deriving from dehydrating conditions in the mouth of dogs and humans (though not sheep[6]) supporting this view[6]. The presence of carbon dioxide is also consistent with a lipid, amino acid/protein and carbohydrate component in this sample.

Notably, these findings did not reveal key biomarkers typically observed in soils[20,23]. It should also be noted that highly polar material could have been present, which would not have successfully eluted from the column, or indeed volatilised sufficiently in the probe.

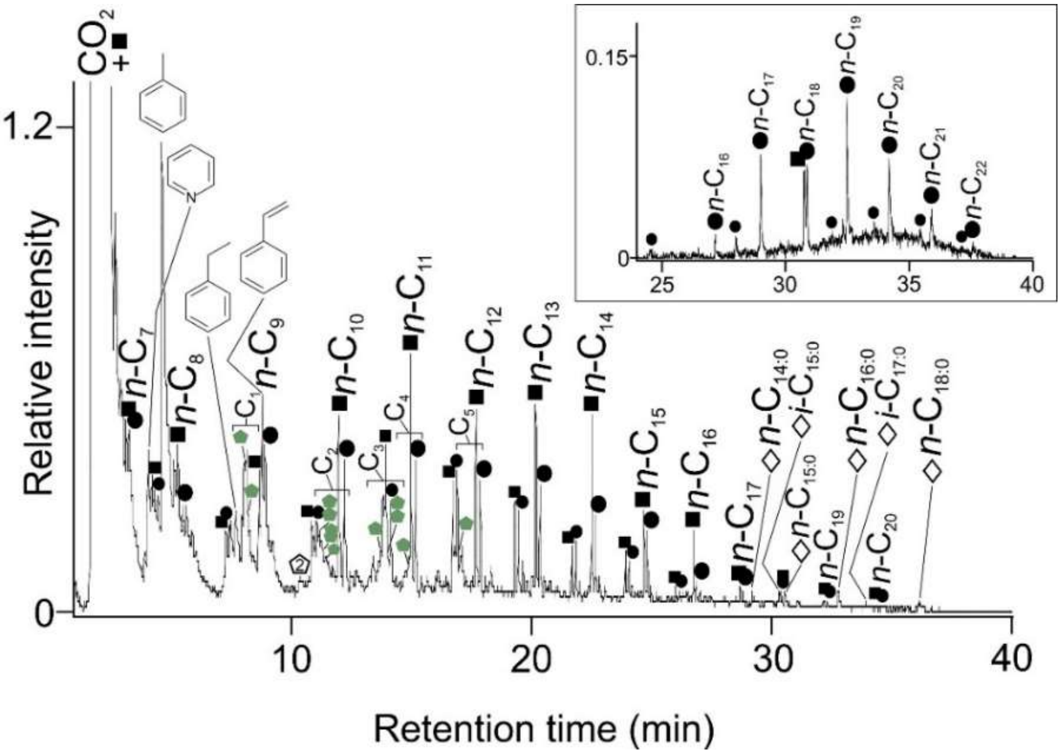

Supplementary Figure 36. Reconstructed total ion chromatogram of the pyrogram (pyrolysis profile) (610 °C for 10s) of sample 1A33, period (AD 7th-12th century), Jakštaičiai-Meškia, after thermal desorption (310 °C for 10 s). Key to Fig. 36: Peak identities (x indicates carbon chain length): filled squares, Cx indicates alkenes; filled circles, Cx indicates alkanes; open diamonds, Cx:y indicates acyclic nitriles; green filled pentagons indicates alkyl pyrroles (with alkyl chain length above, Cx); open pentagons with numerals inside indicates carbohydrate pyrolysis marker, 2 is 2-methyl-2-cyclopenten-1-one. Also shown are the structures of four aromatic compounds identified: pyridine, toluene, ethylbenzene and styrene. CO_2 indicates carbon dioxide. Inset displays a reconstructed total ion chromatogram of the thermal desorption profile (310 °C for 10 s) of this sample. Peak identities: (x indicates carbon chain length): filled square, Cx indicates alkene; filled circles, Cx indicates alkanes.

Discussion, Viking period (AD 7th-12th century), Jakštaičiai-Meškia, North Lithuania barrows

Three samples of dental calculus from individual burials at Jakštaičiai-Meškia dating to the Viking period (AD 7th–12th century) were analysed by sequential thermal desorption-gas chromatography-mass spectrometry (TD-GC-MS) and pyrolysis-gas chromatography-mass spectrometry (Py-GC-MS).

One sample (1A31) revealed only carbon dioxide in the TD-GC-MS indicating the essential absence of free, thermally extractable organic components in these samples. The carbon dioxide is also indicative of highly oxidised organic material. The pyrolysis-GC-MS revealed a series of C_7 to C_{17} *n*-1-alkenes and *n*-alkanes, typical of unsaturated and saturated fat/oil-derived acyl lipids[5,6]. The absence of these compounds in the TD profiles provides molecular evidence for a polymeric/bound acyl lipid source. However, the additional presence of a series of C_8 to C_{16} isoalkenes and isoalkanes, presumably deriving from the branched fatty acids, which are significant components of bacterial triglycerides, strongly suggests a microbial origin for these lipid biomarkers, at least in part. The dominance of carbon dioxide in the pyrogram again suggests a highly oxidised sample.

The other two Jakštaičiai-Meškia samples, 1A32 and 1A33, revealed both free and bound/polymeric organic material. The thermal desorption total ion chromatogram (TIC) of the two samples were dominated by carbon dioxide; it is difficult to explain the significance of this non-diagnostic compound, beyond indicating that this sample has undergone diagenetic changes of a highly oxidising nature. Although only trace constituents, both samples revealed a series of *n*-alkanes, ranging from C_{15}/C_{16} to C_{21}/C_{22}. Sample 1A32 maximises at C_{17}, and 1A33 maximises at C_{19}. Both samples show an odd over even carbon number predominance, although the relative abundance of the C_{18} and C_{20} *n*-alkanes suggests the samples have undergone some biodegradation as a result of diagenetic changes. Although the trace quantities and evidence for biodegradation necessarily mean any inferences must be tentative, the narrow range (between C_{15} and C_{22}) and relatively unusual mid-chain carbon numbers, maximising at C_{17} or C_{19}, combined with an odd over even predominance, is characteristic of an algal source[92,101,103]. The presence of branched alkanes with carbon numbers C_{17} and C_{19} to C_{22} observed in 1A33 as relatively minor constituents is indicative of some microbially-derived biodegradation.

The pyrolysis-GC-MS analyses of these two samples revealed appreciable amounts of organic material which were dominated by a complex suite of organic compounds indicative of lipid (fat/oil) and protein components. These include a series of C_7 to C_{20} *n*-1-alkenes and *n*-alkanes, typical of unsaturated and saturated fat/oil-derived acyl lipids[5,6]. A bimodal distribution with maxima at C_{11} and C_{13}/C_{14} combined with abundant short chain (C_4-C_7) *n*-1-alkenes suggests the original acyl lipids in these samples are likely to have been high in polyunsaturated fatty acids. The absence of these compounds in the TD profile provides molecular evidence for a polymeric/bound acyl lipid source. However, in addition to *n*-1-alkenes and *n*-alkanes, typical of fat/oil derived lipids, the pyrograms also displayed a series of C_8 to C_{16} iso-1-alkenes and isoalkanes, presumably deriving from the branched fatty acids which are significant components of bacterial triglycerides (but see below).

A number of the biomarkers identified here can be indicative of proteins[6,11], i.e. benzene, toluene, pyridine, pyrrole. This is also corroborated by the presence of aliphatic nitriles (carbon numbers $C_{14:0}$, *i*-$C_{15:0}$, $C_{16:0}$ (max) and $C_{18:0}$) in both samples, suggesting a lipid (fat/oil) in this sample which has combined with protein-derived nitrogen to produce these moieties[8]. Far more diagnostic of a particular dietary source are the particular suite of alkyl pyrroles observed in the pyrograms of these samples, with an alkyl chain length of up to C_5. Both these samples revealed appreciable amounts of these biomarkers. This included the 2- and 3-dimethylpyrroles, 2- and 3-ethylpyrroles, 2,4-, 2,3- and 3,4-dimethylpyrroles, three ethylmethylpyrroles, a propylmethylpyrrole and an ethylpropylpyrrole. These specific C_1 to C_5 alkyl pyrroles are highly diagnostic of the presence of an appreciable tetrapyrrole component. Their porphyrin-derived origin, and the relative abundance of some of the key biomarkers, is indicative of an algal source observed in fossils and expected in similarly diagenetically altered archaeological material[11,21,22]. Although C_1 and C_2 alkyl pyrroles can also derive from protein-derived amino acids such as glycine, proline, hydroxyproline, serine and glutamic acid[12-14,20,40,41], the C_3+ alkyl pyrroles identified in these two samples point to a tetrapyrrole source[21,22,40,41]. Moreover, the same specific C_1 to C_5 alkylpyrroles identified in the Py-GC-MS of an algal kerogen[21,22] were observed in these samples. Overall the two samples display a remarkably similar pyrolysis profile to algal-derived kerogen previously studied via very similar organic geochemical techniques[21,22].

The presence here of protein markers and specifically the C_1 to C_5 alkyl pyrroles, combined with the particular lipid profile and specific carbohydrate markers (see above) identified, indicate the presence of a macroalgae, i.e. seaweed or freshwater source, in this sample[21-23].

Marine and freshwater algal sources are known to have been exploited for food in antiquity[52,104], although their consumption in this part of the world does not seem to have been seriously considered to date.

Kanaljorden, Sweden. Mesolithic

Kanaljorden is a Mesolithic ritual wetland-site located at Motala, Östergötland, South Sweden. Ritual depositions, including human crania, had been placed on a stone-packing built on the bottom of a small lake. The human assemblage comprises the remains of 10 adults and one infant. The calculus samples discussed here were taken from individual 2 and 4. Sample 122 represent individual 4, a female 30 – 45 years old, dated to 7834-7573 cal BP (Ua-42118). Sample 126 represent a male 50+ years old, dated to 7664-7518 cal BP (Ua-51722). The depositions also include remains of forest game including wild boar, brown boar, elk and red deer, fish like pike, perch, pikeperch and salmonidae as well as pond-turtle. There are also depositions of hazelnuts, raspberries and tree fungus. The small lake at Kanaljorden was situated close to the large lake Vättern, and the river Motala Ström. The distance to the sea was about 30 km[1,2].

Two samples of dental calculus taken from two individuals from Kanaljorden, Motala, Sweden were analysed by sequential thermal desorption-gas chromatography-mass spectrometry (TD-GC-MS) and pyrolysis-gas chromatography-mass spectrometry (Py-GC-MS). This technique facilitates the identification of both free/unbound and bound/polymeric organic components. Both calculus samples produced very little free organic material, yet a significant amount of a bound/polymeric organic constituent.

52. Kanaljorden 122 (calculus: 0.25 mg)

The thermal desorption total ion chromatogram (TIC) revealed carbon dioxide as the only detectable component, indicating the virtual absence of free, thermally extractable organic components in this sample. It should be noted, however, that highly polar material could have been present, which would not have successfully eluted from the column, or indeed volatilised sufficiently in the probe. It is not possible to determine the significance of the carbon dioxide, beyond it being indicative of oxidised organic material.

In contrast to the thermal desorption profile, the pyrolysis-GC-MS TIC was dominated by C_8 to C_{16} *n*-1-alkenes and *n*-alkanes, typical of unsaturated and saturated fat/oil-derived acyl lipids[5,6]. The absence of these compounds in the TD profile provides molecular evidence for a polymeric/bound acyl lipid source. However, in addition to *n*-1-alkenes and *n*-alkanes, typical of fat/oil derived lipids, the pyrogram also displayed a series of C_{11} to C_{15} 2-methyl *n*-1-alkenes and 2-methyl *n*-alkanes, presumably deriving from the branched fatty acids which are significant components of bacterial triglycerides. There were components identified in the pyrolysate TIC which can be indicative of 'black carbon', i.e. charcoal or soot[7,10]. However, naphthalenes and biphenyl present in 'chars'[7,10] were absent and the dominance of toluene, rather than the normally dominant aromatic hydrocarbon benzene, is notably atypical of chars[7,10]. The markers identified here can also be indicative of proteins[6,11] and combined with the protein markers benzonitrile and indole[11] identified this is a more likely origin for these compounds in this sample. This is also corroborated by the relatively significant abundance of phenol, 2-methylphenol (*o*-cresol), 4-methylphenol (*p*-cresol) (with the 4-methyl isomer being markedly predominant), and minor amounts of 2,4-dimethylphenol and 4-ethylphenol, all of which, collectively, are indicative of the amino acid tyrosine[12-14]. With guaiacol detected only as a trace constituent, and an absence of other guaiacol moieties characteristic of lignin[15,16], the possibility that the phenols may originate from wood or woody plant material can be excluded. Alkyl phenols have also been observed in wood smoke and smoked foods[17,18], however the absence of methoxyphenols, diagnostic of lignin-derived smoke, means this possibility can also be discounted. Proteins rich in tyrosine include meat, fish and dairy products, although the presence of bacterial markers means the possibility that at least a partial origin could be microbial cannot be excluded. However, taken together, the presence of toluene, styrene and phenylacetonitrile (benzyl nitrile) can indicate the amino acid phenylalanine[12,13], which is also high in proteins from meat, fish or dairy. In addition, although minor constituents, indole and 2-methylindole were also identified which are consistent with a tryptophan amino acid source[12,13,20], also notably high in meat, fish or dairy. The presence of carbon dioxide is also consistent with both a lipid and amino acid/protein component in this sample. Notably, these findings revealed no polysaccharide markers or higher alkyl aromatics typically observed in soils[20], nor polynuclear aromatic hydrocarbons (PAHs) indicative of combustion processes.

53. Kanaljorden 126 (calculus: 0.83 mg)

The thermal desorption total ion chromatogram (TIC) revealed carbon dioxide as the only detectable component, indicating the virtual absence of free, thermally extractable organic components in this sample. It should be noted, however, that highly polar material could have been present, which would not have successfully eluted from the column, or indeed volatilised sufficiently in the probe. It is not possible to determine the significance of the carbon dioxide, beyond it being indicative of oxidised organic material.

In contrast to the thermal desorption profile, the pyrolysis-GC-MS TIC was dominated by C_9 to C_{17} *n*-1-alkenes and *n*-alkanes, typical of unsaturated and saturated fat/oil-derived acyl lipids[5,6]. The absence of these compounds in the TD profile provides molecular evidence for a polymeric/bound acyl lipid source. Notably, the series of C_{11} to C_{15} 2-methyl *n*-1-alkenes and 2-methyl *n*-alkanes observed in sample 122 were absent; the lack of branched alkenes and alkanes in this sample suggest this lipid component is not bacterial in origin. There were components identified in the pyrolysate TIC which were indicative of 'black carbon', i.e. charcoal or soot[7-10], and although toluene was the major component, rather than the normally dominant benzene, the associated 'char' markers naphthalene, methylnaphthalene and biphenyl were present in sufficient abundance to suggest a 'char' component in the sample[7-10]. Benzene constituted ~33% of the 'char' markers, with ~46% toluene and moderate quantities of ethyl benzene, xylenes and styrene (~15% total). However, the markers identified here can also be indicative of proteins[6,11], and their presence, combined with the phenolic and nitrogen-containing protein markers[11-14] (see below), is consistent with a protein component in this sample. The relatively significant abundance of phenol, 2-methylphenol (*o*-cresol), 4-methylphenol (*p*-cresol) (with the 4-methyl isomer being notably predominant), and minor amounts of 2,4-dimethylphenol and 4-ethylphenol are, collectively, indicative of the amino acid tyrosine[12-14]. With guaiacol detected only as a very minor constituent, and an absence of other guaiacol moieties characteristic of lignin[15,16], the possibility that the phenols may originate from wood or woody plant material can be excluded. Alkyl phenols have also been observed in wood smoke and smoked foods[17,18], however the absence of methoxyphenols, diagnostic of lignin-derived smoke, means this possibility can also be discounted. Proteins rich in tyrosine include meat, fish and dairy products and the absence of bacterial markers in this sample suggests it may have a dietary origin. Moreover, taken together, the presence of toluene, styrene and phenylacetonitrile (benzyl nitrile) can indicate

the amino acid phenylalanine[12,13], which is also high in proteins from meat, fish or dairy. In addition, although very minor constituents, indole and 2-methylindole were also identified which are consistent with a tryptophan amino acid source[12,13,20] also notably high in meat and fish, or dairy. The presence of carbon dioxide is also consistent with both a lipid and amino acid/protein component in this sample. Notably, these findings revealed no polysaccharide markers or higher alkyl aromatics typically observed in soils[20]. Despite chemical evidence for a 'char' component, polynuclear aromatic hydrocarbons (PAHs) indicative of combustion processes could not be detected, which may reflect the moderate amount of organic material in the sample.

Discussion, Kanaljorden

The results revealed a moderate amount of organic material in the two samples possibly reflecting prevailing environmental conditions for biomolecular preservation. The results revealed not only evidence of bacterially-derived components as one might expect from material taken from the microbially active human mouth, but also evidence for food-based fats and proteins, and in one sample evidence for a char suggesting possible smoke inhalation or cooking. More specifically, both samples revealed chemical evidence for a protein component high in tyrosine and phenylalanine, which may be indicative of a meat or fish source [no dairy likely here]. The profile of the fat markers is indicative of a source with a moderate degree of unsaturation, which is consistent with a meat or fish origin, as indicated by the protein markers.

Iberia

La Corona (Casa Corona), Alicante, Spain

Casa Corona is located in Villena, Alicante, Spain, by the Vinalopó River on the margin of an endorheic basin, around 80 km from the coast. Archaeological material from Late Mesolithic and Chalcolithic periods has been recovered, with the north sector of the site comprising the Late Mesolithic phase. The Late Mesolithic phase consists of several archaeological deposits formed in a grey-brown dark sandy matrix with ashes and organic material and 2 pit graves

with individual inhumations. The site was reoccupied during the Chalcolithic period; this phase comprises pit structures[110].

Three samples of dental calculus taken from one individual from La Corona, Alicante, Spain were analysed by sequential thermal desorption-gas chromatography-mass spectrometry (TD-GC-MS) and pyrolysis-gas chromatography-mass spectrometry (Py-GC-MS). This technique facilitates the identification of both free/unbound and bound/polymeric organic components. Two calculus samples produced no free organic material, and only very minor amounts of bound/polymeric organic constituents, whereas one sample produced a relatively complex suite of both free and bound/polymeric organic constituents.

54. La Corona, Alicante, Spain: 25 Mesolithic (calculus: 5.68 mg)

The thermal desorption total ion chromatogram (TIC) (Fig. 37, inset) is dominated by a 2,5-diketopiperazine derivative of proline-glycine (pro-gly). This is thermally generated in the pyroprobe from the amino acids proline and glycine and is indicative of proteinaceous material containing these two protein constituents[5,12,13]. No other compounds were observed indicating little free, thermally extractable, organic components in this sample. It should be noted, however, that highly polar material could have been present, which would not have successfully eluted from the column, or indeed volatilised sufficiently in the probe.

The Py-GC-MS TIC (Fig. 37) is dominated by C_9 to C_{15} *n*-1-alkenes and *n*-alkanes, typical of unsaturated and saturated fat/oil derived lipids[5,6]. A bimodal distribution with maxima at C_{12} and C_{14} suggests the original acyl lipids may have been high in polyunsaturated fatty acids. The absence of these compounds in the TD profile provides molecular evidence for a polymeric/bound acyl lipid source in this sample. Notably, there were no bacterially-derived hydrocarbons deriving from the original branched fatty acid acyl groups which are significant components of bacterial triglycerides, indicating this lipid component is not bacterial in origin. Other significant components included the aromatic hydrocarbons toluene, ethyl benzene and styrene and although these markers can be indicative of chars when other combustion markers such as benzene, xylenes, naphthalenes and biphenyls are present[7-10], the absence of these additional ‘char’ markers, combined with the abundant nitrogen-containing biomolecules pyrrole, benzonitrile, phenylacetonitrile (benzyl nitrile) and benzenepropanenitrile, is indicative of the presence of a significant protein component in this sample[6,11]. This is also corroborated by the presence, albeit as minor components, of aliphatic

nitriles (carbon numbers $C_{14:0}$ and $C_{16:0}$) also suggesting a lipid (fat/oil) in this sample, which has combined with protein-derived nitrogen to produce these moieties[5]. Taken together, the presence of toluene, styrene and phenylacetonitrile (benzyl nitrile) can indicate the amino acid phenylalanine[12,13], which is high in proteins from meat or fish.

However, highly notable in this context is the additional presence of appreciable amounts of the more unusual 2- and 3-methylpyrroles, 2- and 3-ethylpyrroles, 2,4- and 2,3-dimethylpyrroles, 2-ethyl-4(?)-methylpyrrole, 2-ethyl-3(?)-methylpyrrole, 2,3,5-trimethylpyrrole, 3-ethyl-4-methylpyrrole and an unidentified C_4 alkyl pyrrole. These specific C_1, C_2, C_3 and C_4 alkyl pyrroles suggest a significant porphyrin-derived origin and are indicative of an algal source observed in fossils and expected in similarly diagenetically altered archaeological material[11,21,22]. Notable, given their observance in remarkably similar pyrolysis profiles on dental calculus from Isbister and Quanterness in Orkney and Distillery Cave, Oban (see relevant samples above), were the presence, albeit as moderate to minor components, of the nitrogen containing aromatic compounds 1-H-pyrazole-4-carbonitrile, ethylcyanobenzene, 2-pyridinecarbonitrile and 1H-pyrrole-2-carbonitrile, and two carbohydrate markers; 2-methyl-2-cyclopenten-1-one and an unidentified derivative. The presence here of the abundant protein markers and specifically the C_1 to C_4 alkyl pyrroles, combined with the particular lipid profile and specific carbohydrate markers identified and observed in other ‘seaweed samples’ (see Isbister, Quanterness and Distillery Cave above), suggests the presence of a macroalgae, i.e. seaweed, in this sample. The presence of carbon dioxide is also consistent with both a lipid and amino acid/protein component in this sample. Notably, these findings did not reveal key markers typically observed in soils[20,23], nor polynuclear aromatic hydrocarbons (PAHs) indicative of combustion processes. It should also be note that highly polar material could have been present, which would not have successfully eluted from the column, or indeed volatilised sufficiently in the probe.

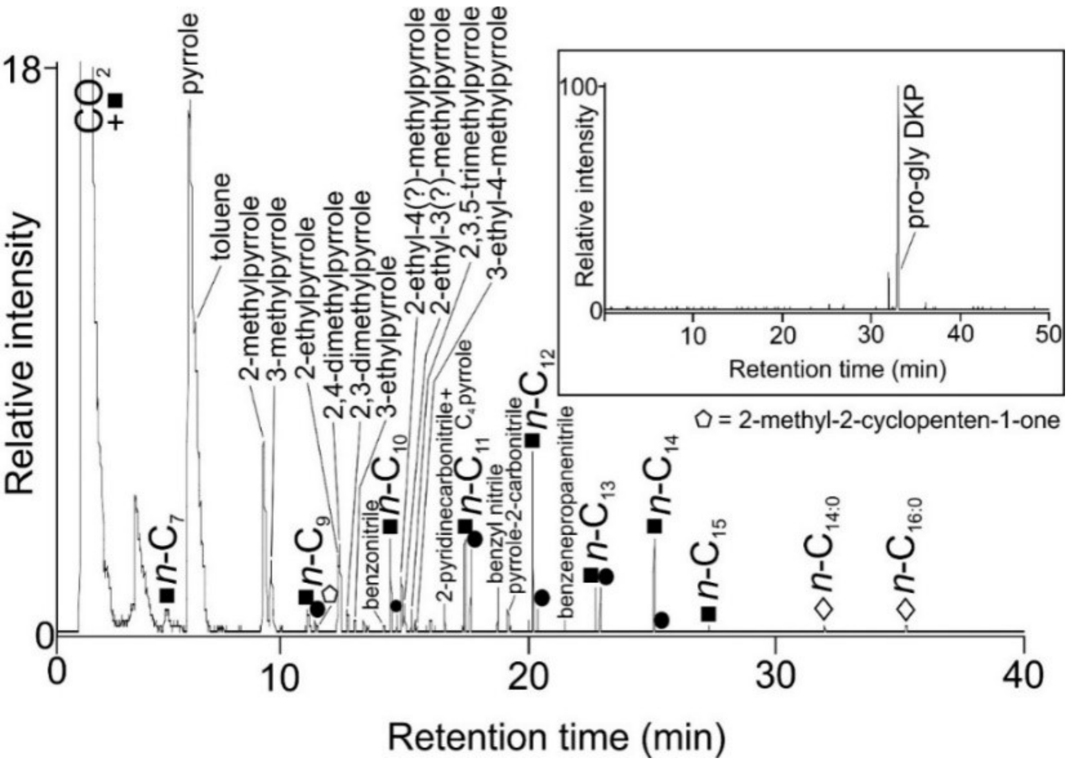

Supplementary Figure 37. Reconstructed total ion chromatogram of the pyrogram (pyrolysis profile) (610 °C for 10) of sample 25, La Corona, Alicante, Spain, after thermal desorption (310 °C for 10 s). Key to Fig. 37: Peak identities (x indicates carbon chain length): filled squares, Cx indicates alkenes; filled circles, Cx indicates alkanes; open diamonds, Cx:0 indicates nitiles; open pentagon indicates the carbohydrate pyrolysis marker, 2-methyl-2-cyclopenten-1-one; CO_2 indicates carbon dioxide. Also shown are toluene, pyrrole and its C_1 to C_4 derivatives, and five aromatic nitriles. Inset displays a reconstructed total ion chromatogram of thermal desorption profile (310 °C for 10 s) this sample. Peak identities: pro-gly DKP indicates the 2,5-diketopiperazine derivative of the amino acids proline and glycine.

55. La Corona 2 M1 mfieq (lingual) 21-05-13 (calculus: 2.58 mg)

The thermal desorption total ion chromatogram (TIC) revealed carbon dioxide as the only detectable component, indicating the essential absence of free, thermally extractable organic components in this sample. It should be noted, however, that highly polar material could have been present, which would not have successfully eluted from the column, or indeed

volatilised sufficiently in the probe. It is not possible to determine the significance of the carbon dioxide, beyond it being indicative of oxidised organic material.

The pyrolysate TIC also contained relatively little detectable organic material, and when taken together with the TD indicates a very low abundance of organic material in this calculus sample, although it should be said that this may be due to diagenetic changes. However, the components which were identified in the pyrolysate TIC were indicative of 'black carbon', i.e. charcoal or soot[7-10]. Excluding the carbon dioxide, benzene was the major compound present in the pyrolysate (~53% of total quantified pyrogram peak area), along with a significant amounts of toluene (~22%) and minor to moderate quantities of ethyl benzene, xylenes and styrene (~5% total). A moderate amount of naphthalene and very minor amount of biphenyl were also detected, which are also typical of chars[7-10]. The protein marker benzonitrile[11] was present in minor abundance (~3%) fairly typical of previously characterised protein-containing chars[7-10]. Indene and two methyl indene derivatives were also identified.

56. La Corona 4 M3 mf dch (lingual) 21-05-13 (calculus: 1.60 mg)

The thermal desorption total ion chromatogram (TIC) revealed carbon dioxide as the only detectable component, indicating the virtual absence of free, thermally extractable organic components in this sample. It should be noted, however, that highly polar material could have been present, which would not have successfully eluted from the column, or indeed volatilised sufficiently in the probe. It is not possible to determine the significance of the carbon dioxide, beyond it being indicative of oxidised organic material.

The pyrolysate TIC also contained relatively little detectable organic material, and when taken together with the TD indicates a very low abundance of organic material in this calculus sample, although it should be said that this may be due to diagenetic changes. However, the components which were identified in the pyrolysate TIC were indicative of 'black carbon', i.e. charcoal or soot[7-10]. Excluding the carbon dioxide, benzene was the major compound present in the pyrolysate (~44% of total quantified pyrogram peak area), along with a significant amounts of toluene (~22%) and moderate quantities of ethyl benzene, xylenes and styrene (~8% total). A moderate amount of naphthalene was also detected, which is also typical of chars[7-10]. The protein marker benzonitrile[11] was present in minor abundance (~2%) fairly typical of previously characterised protein-containing chars[7-10] and very similar

to the other calculus sample analysed. Indene and two methyl indene derivatives were also identified.

La Corona sediment 'control' (sediment/soil: 10.92 mg)

A sample of sediment from the area surrounding the remains at the La Corona site was also analysed. The thermal desorption total ion chromatogram (TIC), like the two calculus samples, revealed carbon dioxide as the only detectable component, indicating the virtual absence of free, thermally extractable organic components in this sample. It is not possible to determine the significance of the carbon dioxide, beyond it being indicative of oxidised organic material.

The pyrolysate TIC contained very little detectable organic material, and when taken together with the TD indicates an extremely low abundance of organic material in the sediment, likely to be due to diagenetic changes. The only components identified in the pyrolysate TIC were carbon dioxide and trace amounts of the aromatic hydrocarbons, benzene and toluene, well below the levels identified in the two dental calculus samples. The other regarded char markers observed in the two calculus samples were not detected in the sediment.

Discussion, La Corona

In sample 54, the TD-GC-MS analysis revealed only the 2,5-diketopiperazine derivative (DKP) of proline-glycine (pro-gly) as the only 'free' organic compound detected. No other compounds were observed indicating little free, thermally extractable, organic components in this sample. It is perhaps notable, however, that this DKP, along with a far lesser amount of the DKP of proline-alanine (pro-ala), dominated the thermal desorption profile of samples with an essentially identical complex chemical profile in the Py-GC-MS, where tetrapyrroles deriving from [macro]algae were observed (see below). Pyrroles can derive from proteins containing glycine, proline, hydroxyproline and glutamic acids[20,111] (although see below), which may partially explain their presence here and suggest a protein high in these four amino acids. Interestingly, these four protein constituents, along with alanine, are notably high in seaweeds[48] (see below).

The C_9 to C_{15} *n*-1-alkenes and *n*-alkanes observed in the Py-GC-MS is typical of unsaturated and saturated fat/oil derived acyl lipids[5,6]. The bimodal distribution with maxima at C_{12} and

C_{14} suggests the original acyl lipids may have been high in polyunsaturated fatty acids, which is consistent with a marine source. Although the long chain moieties characteristic of a marine input (eicosapentaenoic acid, $C_{20:5}$ [EPA] and docosahexaenoic acid, $C_{22:6}$ fatty acid [DHA]) were not detected, these compounds are particularly labile and susceptible to degradation. Perhaps notably, the expected biopolymeric products generated during pyrolysis if these biomolecules were incorporated into a bound organic fraction would be expected to maximise at C_{11}/C_{12} and C_{14} *n*-1-alkene/*n*-alkanes, i.e. promote a bimodal distribution of alkene/alkanes, as is observed in this sample, although other possible food sources high in polyunsaturated fatty acids could also explain this molecular profile. Importantly, no microbial biomarkers (e.g. branched hydrocarbons) were observed, indicating this lipid component is not microbial in origin.

Far more diagnostic of a particular dietary source are the particular suite of alkyl pyrroles observed in the pyrogram. The presence of appreciable amounts of 2-methylpyrrole, 3-methylpyrrole, 2-ethylpyrrole, 3-ethylpyrrole, 2,4-dimethylpyrrole, 2,3-dimethylpyrroles, 2-ethyl-4(?)-methylpyrrole, 2-ethyl-3(?)-methylpyrrole, 2,3,5-trimethylpyrrole, 3-ethyl-4-methylpyrrole and an unidentified C_4 alkyl pyrrole is highly significant. These specific C_1 to C_4 alkyl pyrroles are diagnostic of the presence of a significant tetrapyrrole component. Their porphyrin-derived origin are indicative of an algal source observed in fossils and expected in similarly diagenetically altered archaeological material[11,21,22]. Overall the sample displays a remarkably similar pyrolysis profile to dental calculus samples from Isbister and Quanterness in Orkney and Distillery Cave, Oban (see relevant samples above). The aromatic hydrocarbons toluene, ethyl benzene and styrene, combined with pyrrole, benzonitrile, phenylacetonitrile and benzenepropanenitrile, are also collectively indicative of the presence of a significant protein component[6,9], with the aliphatic nitriles identified suggesting a source high in both protein and fat/oil. The additional presence of specific nitrogen-containing organic compounds, such as the carbonitriles and two carbohydrate markers observed, is highly notable. These exact same compounds were also identified, in approximately the same relative proportions, in dental calculus from Isbister and Quanterness in Orkney and Distillery Cave, Oban (see relevant samples above) Collectively, the presence here of the abundant protein markers and specifically the C_1 to C_4 alkyl pyrroles, combined with the particular lipid profile and exact same protein and carbohydrate markers identified and observed in other 'seaweed samples' (see Isbister, Quanterness and Distillery Cave sections above), strongly suggests the presence of a macroalgae, i.e. seaweed, in this sample. Seaweed

is known to have been exploited for food in antiquity[52], although its consumption in this part of the world does not seem to have been seriously considered to date.

That being said, due to the relative distance between the La Corona site and the Alicante coast (~50km) it is sensible to consider other possible sources for this algal chemical signature in the dental calculus. There are endorheic lagoons and ponds in the area[112], which may have had the potential to provide sufficient nutrients for freshwater algae such as spirulina. Freshwater algae is known to have been used as a source of food in antiquity[52,113], but current evidence suggests the environmental conditions, and likely absence of the appropriate taxa in any significant abundance necessary for it to have been considered as a useful and viable food source, makes this a remote possibility at La Corona. With this considered, the consumption of seaweed as part of the diet is the most credible explanation, and if this was a seasonal component the relatively short distance to the coast means the exploitation of marine resources, particularly at this time, i.e. late Mesolithic, would not be particularly surprising.

That being said, stable isotopic analyses have not detected a marine component in the diet at this time[112,114]. The same research also concluded there was no lagoonal food source in the diet at this Late Mesolithic site[112,114]. The research has emphasised a wholly terrestrial diet, despite marine shellfish being identified the site, and whose presence has been explained as body adornment, with no exploitation of these marine invertebrates as a food resource[112]. Moreover, it is suggested that not only is there no isotopic evidence, but there is no archaeological evidence for the consumption of marine resources[114]. This interpretation is interesting given the biomolecular evidence presented here as part of this current study. Importantly, it should be noted that if there was any marine component, such as fish or shellfish, it would be expected that the isotopic data would reveal this if it was a significant dietary component, i.e. greater than ~20%. However, if marine algae, i.e. seaweed (or other marine resources), was being consumed, particularly as a seasonal food resource, the presence of this component, even if significant in the diet, i.e. up to 20%, would not be detected. This serves to emphasise the potential of this biomarker approach to provide key additional, and often complimentary, palaeodietary evidence.

To conclude, in direct contradiction to the previous conclusion that: "...this population did not have any direct and regular dietary contact with the sea."[112], the organic chemistry of the dental calculus in the individual analysed here points to a macroalgal constituent in the diet at

La Corona. When taken with the stable isotopic evidence, which would suggest that a freshwater [micro]algal source is indeed less likely, it points to the consumption of an appreciable amount of seaweed in this individual, albeit possibly a seasonal food resource at this Late Mesolithic inland site. This strongly, if not unequivocally, suggests that at least part of the population at La Corona did indeed have dietary contact with the sea, even if limited by seasonal factors, although further research on a larger sample size of individuals would be needed to better understand the significance for the wider population at this site.

The results from the other two samples, revealed very little organic material in the samples possibly reflecting unfavourable prevailing environmental conditions for biomolecular preservation. Of those organic molecules identified they were indicative of a char of soot, possibly suggesting smoke inhalation or cooking, although the identification of trace levels of the two main char markers in the sediment, which was also analysed, necessarily make this inference somewhat tentative.

Bòbila Madurell, Catalonia, Spain

Bòbila Madurell is a Neolithic cemetery in north east Iberia. It contains 179 documented pit burials[115.] Stable isotope analysis suggests the diet was based on terrestrial domesticated plants and animals[116].

One sample of dental calculus from Bòbila Madurell was analysed by sequential thermal desorption-gas chromatography-mass spectrometry (TD-GC-MS) and pyrolysis-gas chromatography-mass spectrometry (Py-GC-MS).

57. Bòbila Madurell, Catalonia, Spain: MF18 male, Neolithic (calculus: 11.82 mg)

The thermal desorption total ion chromatogram (TIC) revealed carbon dioxide as the only detectable (trace) component, indicating the essential absence of free, thermally extractable organic components in this sample. It should be noted, however, that highly polar material could have been present, which would not have successfully eluted from the column, or indeed volatilised sufficiently in the probe. It is not possible to determine the significance of the carbon dioxide, beyond it being indicative of oxidised organic material.

The pyrolysate TIC contained very little detectable organic material. The only components identified were carbon dioxide and a trace amount of the aromatic hydrocarbon toluene. Given the wide natural distribution of toluene and the absence of other organic compounds its origin here remains uncertain. Nor is it possible to determine the significance of the carbon dioxide, beyond it being indicative of oxidised organic material.

Discussion, Bòbila Madurell, Catalonia

The results revealed a virtual absence of organic material, most likely reflecting unfavourable prevailing environmental conditions for biomolecular preservation. The extremely low level of carbon dioxide and toluene as the only compounds detected make any meaningful interpretation impossible, beyond indicating the unfavourable environmental conditions already mentioned.

Muge and Sado

Mesolithic shells middens found along the Tagus River in Portugal have been known about for over 150 years[117] while Mesolithic shell middens in the nearby and Sado Valley were first recorded in the 1930s. Since this time, several periods of excavation have been undertaken, and well over 100 human skeletons have been recovered. Perforated shell jewellery and the use of ochre have been found in association with some of the burials. Radiocarbon dates suggest that the Muge middens were in use between 6100-5600 cal BC[118]. The development of the middens has been linked to the 8.2ka cold event, as a response to a decline in marine resources and an increase in sea level leading the hunter gatherer fishers to move inland. Stable isotope analysis on samples from the Sado River middens suggest that the diet comprised terrestrial animals, a small mount of terrestrial plants, and around a 20% component comprising marine resources[79].

Eleven samples of dental calculus taken from eleven individuals from the sites Moita do Sebastião and Cabeço da Arruda in the Muge Valley, Portugal and Cabeço do Pez in the Sado Valley, Portugal, were analysed by sequential thermal desorption-gas chromatography-mass spectrometry (TD-GC-MS) and pyrolysis-gas chromatography-mass spectrometry (Py-GC-MS). This technique facilitates the identification of both free/unbound and bound/polymeric

organic components. The calculus samples revealed variable amounts of both free organic material and bound/polymeric organic constituents.

58. Moita do Sebastião, Muge, Portugal: MSD male (calculus: 2.59 mg)

The thermal desorption total ion chromatogram (TIC) revealed no detectable components, indicating the absence of free, thermally extractable organic components in this sample. It should be noted, however, that highly polar material could have been present, which would not have successfully eluted from the column, or indeed volatilised sufficiently in the probe.

The pyrolysate TIC revealed carbon dioxide as the only detectable (trace) component, indicating the essential absence of organic material in this sample. It should be noted, however, that highly polar material could have been present, which would not have successfully eluted from the column, or indeed volatilised sufficiently in the probe. It is not possible to determine the significance of the carbon dioxide, beyond it being indicative of oxidised organic material, although its presence as only a very trace component makes even this inference of no meaningful value.

59. Moita do Sebastião, Muge, Portugal: MSY female (calculus: 2.39 mg)

The thermal desorption total ion chromatogram (TIC) revealed no detectable components, indicating the absence of free, thermally extractable organic components in this sample. It should be noted, however, that highly polar material could have been present, which would not have successfully eluted from the column, or indeed volatilised sufficiently in the probe.

The pyrolysate TIC revealed carbon dioxide as the only detectable (minor trace) component, indicating the essential absence of organic material in this sample. It should be noted, however, that highly polar material could have been present, which would not have successfully eluted from the column, or indeed volatilised sufficiently in the probe. It is not possible to determine the significance of the carbon dioxide, beyond it being indicative of oxidised organic material, although its presence as only a very trace component makes even this inference of no meaningful value.

60. Moita do Sebastião, Muge, Portugal: MS7.3 male (calculus: 2.37 mg)

The thermal desorption total ion chromatogram (TIC) revealed carbon dioxide as the only detectable component, indicating the essential absence of free, thermally extractable organic components in this sample. It should be noted, however, that highly polar material could have been present, which would not have successfully eluted from the column, or indeed volatilised sufficiently in the probe. It is not possible to determine the significance of the carbon dioxide, beyond it being indicative of oxidised organic material.

The pyrolysate TIC revealed carbon dioxide as the only detectable component, indicating the essential absence of organic material in this sample. It should be noted, however, that highly polar material could have been present, which would not have successfully eluted from the column, or indeed volatilised sufficiently in the probe. It is not possible to determine the significance of the carbon dioxide, beyond it being indicative of oxidised organic material.

61. Moita do Sebastião, Muge, Portugal: MS29 child F.1-6 (female) (calculus: 0.67 mg)

The thermal desorption total ion chromatogram (TIC) revealed no detectable components, indicating the absence of free, thermally extractable organic components in this sample. It should be noted, however, that highly polar material could have been present, which would not have successfully eluted from the column, or indeed volatilised sufficiently in the probe.

The pyrolysate TIC revealed no detectable components, indicating the essential absence of organic material in this sample. It should be noted, however, that highly polar material could have been present, which would not have successfully eluted from the column, or indeed volatilised sufficiently in the probe.

62. Moita do Sebastião, Muge, Portugal: MS32 male (calculus: 1.85 mg)

The thermal desorption total ion chromatogram (TIC) revealed carbon dioxide as the only detectable (trace) component, indicating the essential absence of free, thermally extractable organic components in this sample. It should be noted, however, that highly polar material could have been present, which would not have successfully eluted from the column, or indeed volatilised sufficiently in the probe. It is not possible to determine the significance of the carbon dioxide, beyond it being indicative of oxidised organic material.

The pyrolysate TIC revealed carbon dioxide as the only detectable (trace) component, indicating the essential absence of organic material in this sample. It should be noted, however, that highly polar material could have been present, which would not have successfully eluted from the column, or indeed volatilised sufficiently in the probe. It is not possible to determine the significance of the carbon dioxide, beyond it being indicative of oxidised organic material.

63. Moita do Sebastião, Muge, Portugal: MS56 male (calculus: 3.97 mg)

The thermal desorption total ion chromatogram (TIC) revealed no detectable components, indicating the absence of free, thermally extractable organic components in this sample. It should be noted, however, that highly polar material could have been present, which would not have successfully eluted from the column, or indeed volatilised sufficiently in the probe.

The pyrolysate TIC revealed carbon dioxide as the only detectable (trace) component, indicating the essential absence of organic material in this sample. It should be noted, however, that highly polar material could have been present, which would not have successfully eluted from the column, or indeed volatilised sufficiently in the probe. It is not possible to determine the significance of the carbon dioxide, beyond it being indicative of oxidised organic material.

64. Moita do Sebastião, Muge, Portugal: MS60 female (calculus: 1.30 mg)

The thermal desorption total ion chromatogram (TIC) revealed no detectable components, indicating the absence of free, thermally extractable organic components in this sample. It should be noted, however, that highly polar material could have been present, which would not have successfully eluted from the column, or indeed volatilised sufficiently in the probe.

The pyrolysate TIC revealed no detectable components, indicating the essential absence of organic material in this sample. It should be noted, however, that highly polar material could have been present, which would not have successfully eluted from the column, or indeed volatilised sufficiently in the probe.

Discussion, Moita do Sebastião, Muge

Seven samples of dental calculus taken from seven individuals from Moita do Sebastião were analysed by sequential thermal desorption-gas chromatography-mass spectrometry (TD-GC-MS) and pyrolysis-gas chromatography-mass spectrometry (Py-GC-MS). The results revealed a virtual absence of organic material in all samples, most likely reflecting unfavourable prevailing environmental conditions for biomolecular preservation. The extremely low levels of carbon dioxide as the only compound identified make any meaningful interpretation impossible, beyond indicating the unfavourable environmental conditions already mentioned.

65. Cabeço da Arruda, Muge, Portugal: ARR XIII (113) female (calculus: 5.70 mg)

The thermal desorption total ion chromatogram (TIC) (Fig. 38) is dominated by an unresolved complex mixture (UCM; apparent as a 'hump' in the chromatogram) of alkenes and alkanes, carbon numbers C_{25} to C_{35}, within which there is series of C_{25} to C_{35} *n*-alkanes (odd over even predominance) dominating this UCM profile and maximising at *n*-C_{29}. Branched alkanes, primarily *iso*- and *anteiso*- with carbon numbers C_{27}, C_{29} and C_{31}, are also notable and are possibly indicative of microbially-derived biodegradation (but see discussion below). These unusually high carbon numbers present in the TD chromatogram may suggest a higher plant source, possibly deriving from plant waxes in the original food consumed[6] (but, again, see summary below). No lipid, protein or carbohydrate markers were observed, with the only other compound detected being the non-diagnostic carbon dioxide. It should also be note that highly polar material could have been present, which would not have successfully eluted from the column, or indeed volatilised sufficiently in the probe.

The pyrolysate TIC (Fig. 38, inset) identified components indicative of fat/oil-derived lipids or higher plant waxes. Toluene was also present, albeit a relatively minor compound in the pyrolysate. No protein or carbohydrate markers were observed. The Py-GC-MS profile was dominated by C_7 to C_{21} *n*-1-alkenes and *n*-alkanes, typical of unsaturated and saturated fat/oil derived lipids and higher plant waxes [5,6]. An essentially unimodal distribution with a clear maximum at C_{11}, and a secondary lesser maximum at C_{14} may indicate the presence of polyunsaturated fatty acids in the original acyl lipids, although the 'chemical fingerprint' is notably analogous to that previously observed in archaeological beeswax[119-121] (see

discussion below). Importantly, there were no bacterially-derived hydrocarbons deriving from the original branched fatty acid acyl groups which are significant components of bacterial triglycerides, indicating this lipid component is not bacterial in origin. There was no convincing evidence of a char suggesting smoke inhalation or cooking. It should also be note that highly polar material could have been present, which would not have successfully eluted from the column, or indeed volatilised sufficiently in the probe.

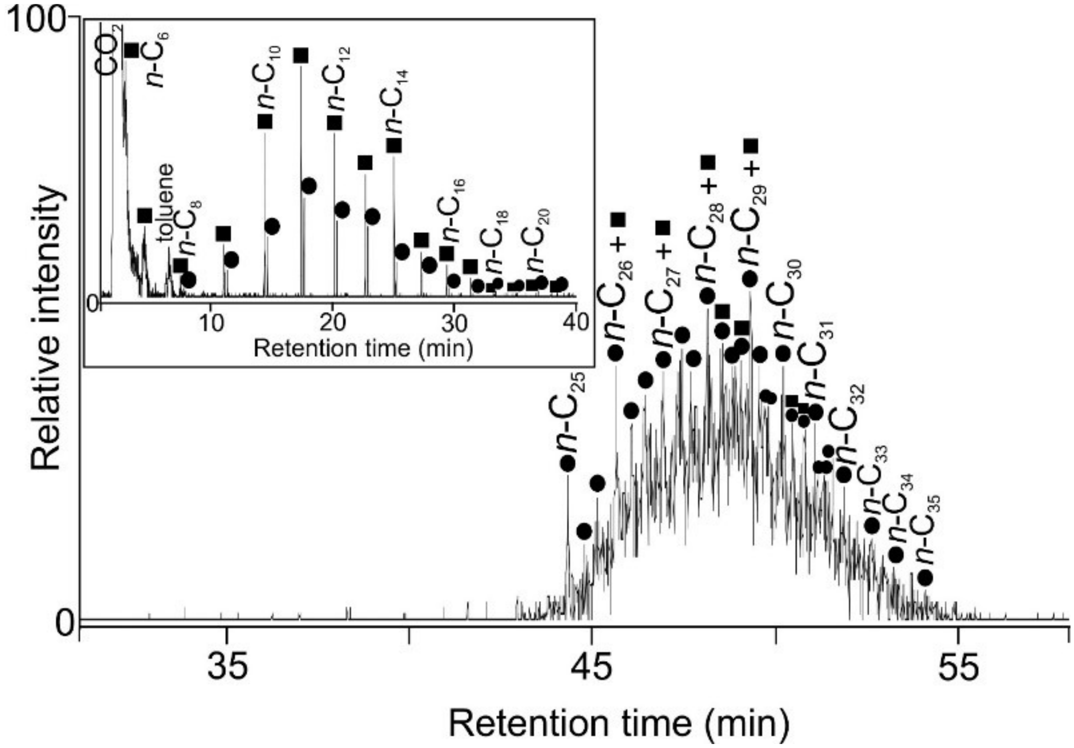

Supplementary Figure 38. Reconstructed total ion chromatogram of the thermal desorption profile (310 °C for 10 s) of sample ARR XIII (113) female, Cabeço da Arruda, Muge, Portugal. Key to Fig. 38: Peak identities (x indicates carbon chain length): filled squares indicates alkenes; filled circles, Cx indicates alkanes.Inset displays a reconstructed total ion chromatogram of the pyrogram (pyrolysis profile) (610 °C for 10) of this sample, after thermal desorption (310 °C for 10 s). Peak identities: (x indicates carbon chain length): filled squares, Cx indicates alkenes; filled circles, Cx indicates alkanes; CO_2 indicates carbon dioxide. Also shown is toluene.

Discussion, Cabeço da Arruda, Muge

One sample of dental calculus from Cabeço da Arruda was analysed by sequential thermal desorption-gas chromatography-mass spectrometry (TD-GC-MS) and pyrolysis-gas chromatography-mass spectrometry (Py-GC-MS). Although not abundant, the sample contained both free and bound/polymeric organic material indicative of a fat/oil or wax. When taken together, the TD and Py chromatograms provide two conceivable interpretations of the chemical evidence presented in this sample. 1. It is possible that the biomolecules indicate a biodegraded higher plant wax deriving from the original higher plant consumed (e.g. leafy green vegetables). 2. Both the TD-GC-MS and Py-GC-MS chemical profiles are notably similar to biodegraded/archaeological beeswax[119-121] and have interesting similarities with the closely chemically related bee product propolis[122]. The *n*-alkane distribution in beeswax is typically C_{23} to C_{35}/C_{25} to C_{33}, maximising at C_{27}[119-121]. In the dental calculus sample the C_{25} to C_{35}, maximising at C_{29}, is notably similar, and in archaeological samples where beeswax has been positively identified C_{29} has been observed as the major *n*-alkane as a result biodegradation[119-121]. The C_{27}, C_{29} and C_{31} *iso*-branched alkanes, maximising at C_{29}, also identified may be telling, since the C_{29} and C_{31} *iso*-branched alkanes, also maximising at C_{29}, have been observed as significant components in propolis wax[122]. Moreover, the 'chemical fingerprint' obtained from Py-GC-MS analysis is distinctive for beeswax given its particular chemistry, and although palmitic acid (possibly due to diagenetic changes) and the long-chain hydrocarbon moieties from C_{24} to C_{30} (even over odd predominance) were not observed (conceivably due to the small amount of organic material present; these are in relatively low abundance in archaeological samples compared to the shorter chain hydrocarbons[119,120]), the chemical profile has notable similarities with pyrolysed fresh and archaeological beeswax[119,120]. Taken together, the chemical evidence from both the TD-GC-MS and Py-GC-MS is consistent with a biodegraded beeswax or propolis wax, which would support previous evidence for its widespread use across Europe in prehistory[123-126]. However, corroborating archaeological/scientific evidence for beeswax or propolis <u>would</u> be needed in order to support this currently tentative, albeit potentially very interesting, inference.

66. Cabeço do Pez, Sado Valley, Portugal: b251-b female (calculus: 1.77 mg)

The thermal desorption total ion chromatogram (TIC) (Fig. 39) is dominated by a series of *n*-alkanes, carbon numbers C_{22} to C_{27}, maximising at C_{23}. There is no odd over even carbon

number predominance suggesting the sample may have undergone biodegradation as a result of diagenetic changes. The relatively unusual mid-chain carbon numbers present in the TD chromatogram suggests a submerged aquatic plant (macrophyte)[89-92], presumably deriving from the original food consumed. No acyl lipid (including bacterially-derived), protein or carbohydrate markers were observed, and nor were any other compounds identified. It should also be note that highly polar material could have been present, which would not have successfully eluted from the column, or indeed volatilised sufficiently in the probe.

The pyrolysate TIC revealed carbon dioxide as the only detectable (trace) component, indicating little organic material in this sample, beyond that identified in the TD. It should be noted, however, that highly polar material could have been present, which would not have successfully eluted from the column, or indeed volatilised sufficiently in the probe. It is not possible to determine the significance of the carbon dioxide; theoretically it is indicative of oxidised organic material, although its presence as only a barely detectable trace component makes even this inference of no meaningful value.

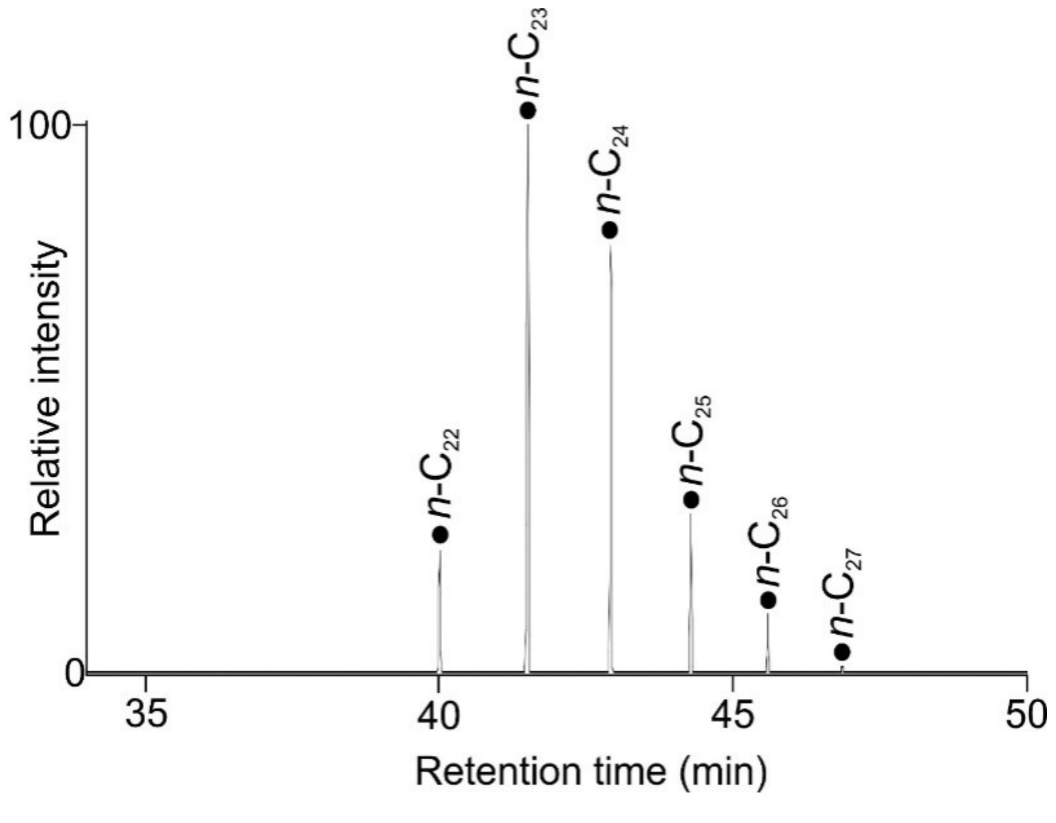

Supplementary Figure 39. Reconstructed total ion chromatogram of the thermal desorption profile (310 °C for 10 s) of sample b251-b female, Cabeço do Pez, Sado Valley, Portugal.

Key to Fig. 39: Peak identities (x indicates carbon chain length): filled circles, Cx indicates alkanes.

67. Cabeço do Pez, Sado Valley, Portugal: Esq27 Esquina male (calculus: 4.06 mg)

The thermal desorption total ion chromatogram (TIC) (Fig. 40) is dominated by a series of *n*-alkanes, carbon numbers C_{19} to C_{29}, maximising at C_{22}. There is no odd over even carbon number predominance suggesting the sample may have undergone biodegradation as a result of diagenetic changes. The relatively unusual mid-chain carbon numbers present in the TD chromatogram suggests a submerged aquatic plant (macrophyte)[89-92], presumably deriving from the original food consumed. No acyl lipid (including bacterially-derived), protein or carbohydrate markers were observed, with the only other compound detected being carbon dioxide as a minor constituent. It is not possible to determine the significance of the carbon dioxide, beyond it being indicative of a small amount of oxidised organic material. It should also be note that highly polar material could have been present, which would not have successfully eluted from the column, or indeed volatilised sufficiently in the probe.

The pyrolysate TIC revealed carbon dioxide as the only detectable (trace) component, indicating little organic material in this sample, beyond that identified in the TD. It should be noted, however, that highly polar material could have been present, which would not have successfully eluted from the column, or indeed volatilised sufficiently in the probe. It is not possible to determine the significance of the carbon dioxide, beyond it being indicative of oxidised organic material, although its presence as only a trace component makes even this inference of very little value.

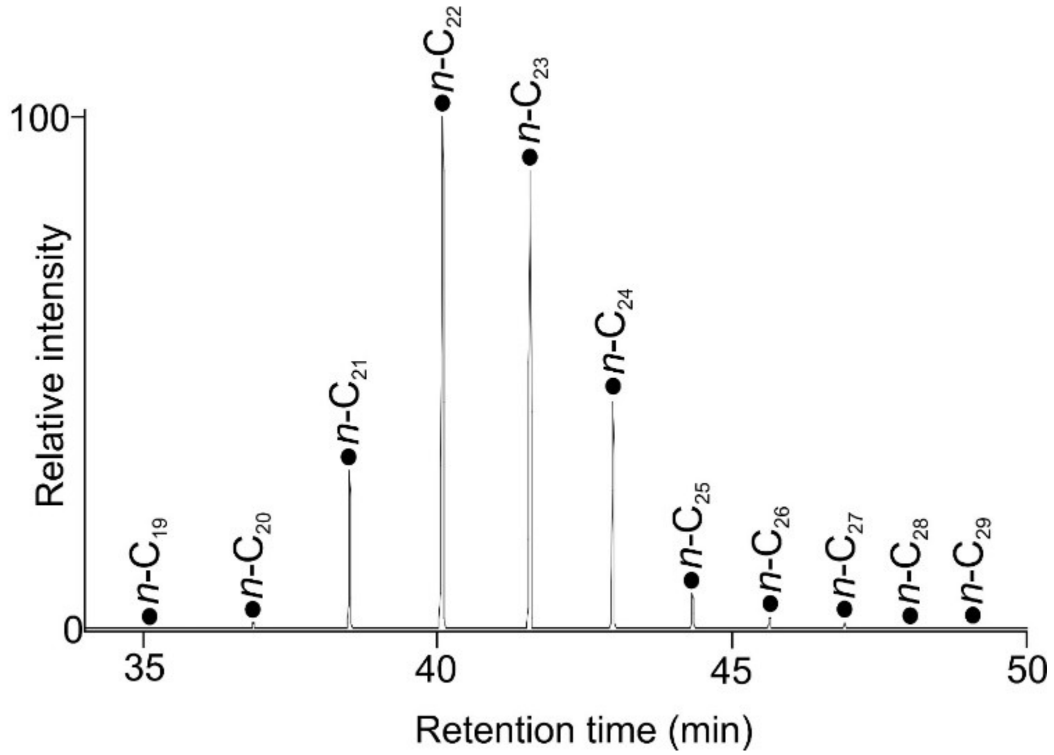

Supplementary Figure 40. Reconstructed total ion chromatogram of the thermal desorption profile (310 °C for 10 s) of sample Esq27 Esquina male, Cabeço do Pez, Sado Valley, Portugal. Key to Fig. 40: Peak identities (x indicates carbon chain length): filled circles, Cx indicates alkanes.

68. Cabeço do Pez, Sado Valley, Portugal: 6250-D Esquina A-B Jordi male (calculus: 2.65 mg)

The thermal desorption total ion chromatogram (TIC) (Fig. 41) is dominated by a series of *n*-alkanes, carbon numbers C_{21} to C_{26}, maximising at C_{23}. There is no odd over even carbon number predominance suggesting the sample may have undergone biodegradation as a result of diagenetic changes. The relatively unusual mid-chain carbon numbers present in the TD chromatogram suggests a submerged aquatic plant (macrophyte)[89-92], presumably deriving from the original food consumed. No acyl lipid (including bacterially-derived), protein or carbohydrate markers were observed, with the only other compound detected being carbon dioxide. It is not possible to determine the significance of the carbon dioxide, beyond it being indicative of a small amount of oxidised organic material. It should also be note that highly

polar material could have been present, which would not have successfully eluted from the column, or indeed volatilised sufficiently in the probe.

The pyrolysate TIC revealed carbon dioxide as the only detectable (trace) component, indicating little organic material in this sample, beyond that identified in the TD. It should be noted, however, that highly polar material could have been present, which would not have successfully eluted from the column, or indeed volatilised sufficiently in the probe. It is not possible to determine the significance of the carbon dioxide, beyond it being indicative of oxidised organic material, although its presence as only a trace component makes even this inference of very little value.

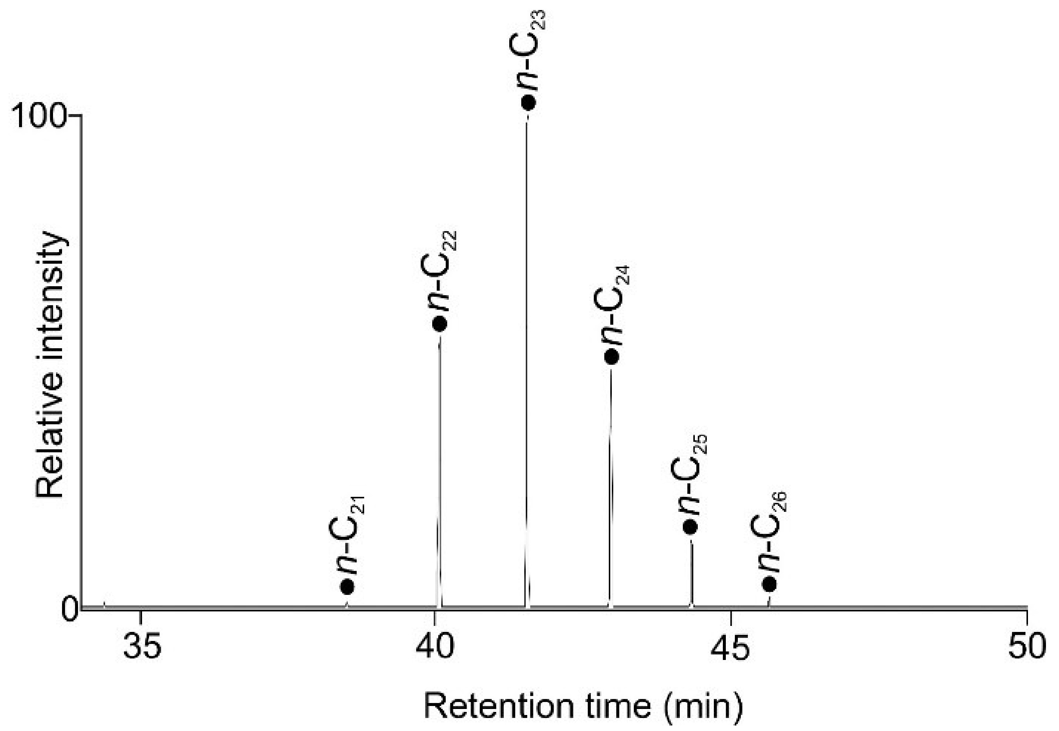

Supplementary Figure 41. Reconstructed total ion chromatogram of the thermal desorption profile (310 °C for 10 s) of sample 6250-D Esquina A-B Jordi male, Cabeço do Pez, Sado Valley, Portugal. Key to Fig. 41: Peak identities (x indicates carbon chain length): filled circles, Cx indicates alkanes.

Discussion. Cabeço do Pez, Sado

Three samples of dental calculus taken from three individuals from Cabeço do Pez, Sado were analysed by sequential thermal desorption-gas chromatography-mass spectrometry (TD-GC-MS) and pyrolysis-gas chromatography-mass spectrometry (Py-GC-MS). The Py-GC-MS results revealed trace quantities of carbon dioxide as the only compound detected in all three samples, indicating an essential absence of a preserved macromolecular organic component in these samples, and most likely reflecting relatively unfavourable prevailing environmental conditions for biomolecular preservation. Despite the virtual absence of organic material in the pyrolysate, however, the thermal desorption total ion chromatogram (TIC) of all three samples was dominated by a series of *n*-alkanes, with carbon numbers notably similar, ranging from C_{19} up to C_{29}, maximising at C_{22}/C_{23} (see Fig. 42). There is no odd over even carbon number predominance suggesting the samples may have undergone biodegradation as a result of diagenetic changes (although see below). The relatively unusual mid-chain carbon numbers present strongly suggests a submerged aquatic plant (macrophyte)[89-92], since these typically have a plant wax component ranging from C_{19} to C_{31}/C_{33} and maximising at C_{23}, presumably deriving from the original food consumed in this context. No acyl lipid (including bacterially-derived), protein or carbohydrate markers were observed, which is consistent with a significantly biodegraded organic component, as is the persistence of the *n*-alkanes which are known to be particularly resistant to diagenesis[90,92]. In addition to the data above, an *n*-alkane proxy for the input/presence of submerged/floating freshwater aquatic macrophytes provides another corroborating measure of aquatic plant input[34]. This proxy ratio, P_{aq}, gives values of 1.0 for all three samples, which also indicates a submerged/floating aquatic plant[89].

The average C_{23} maximum, combined with the variable and relatively narrow *n*-alkane carbon number range (C_{21} to C_{26} >2%) in the calculus samples, with no branched alkanes detected, identifies biodegraded submerged aquatic plant (macrophyte) material. With carbon preference indexes (CPIs) of 1.17 and 1.12 for both samples with a C_{23} maximum (suggesting less of a diagenetic impact in these samples), they are entirely consistent with what would be expected for a (biodegraded) submerged aquatic plant. Notably similar values have been observed for submerged aquatic plant rhizomes from southern Europe: Greece, CPI = 1.13; France, CPI = 1.15[101]. Furthermore, the rhizomes in these studies had a far less pronounced odd-over-even carbon preference than leaves from the same plants, which is likely to reflect their microenvironment in the marine sediments where they are far more exposed to biomolecular reworking and biodegradation from microbial inputs than leaves from the same

plant. In this regard it is notable that the *n*-alkanes in the dental calculus samples also display this unimodal ‘hump’; this could be indicative of the consumption of rhizomes from a submerged aquatic plant by these individuals, although it may also be explained by post-depositional diagenetic factors.

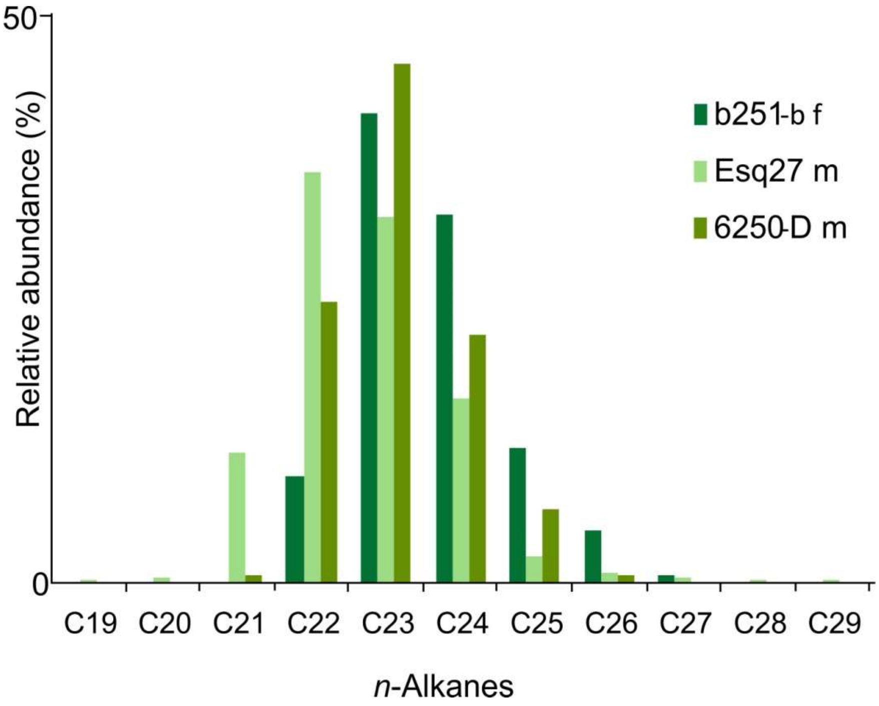

Supplementary Figure 42. *n*-Alkane distribution of three calculus samples from Cabeço do Pez, Sado: b251-b female; Esq27 Esquina male; 6250-D Esquina A-B Jordi male. Source data are provided as a Source Data file.

Although the identification of plant remains is challenging, exploitation of aquatic plants as food in the Iberian Peninsula has been suggested in the Mesolithic period, where aquatic plant storage tissue has been identified[127]; Alismataceae (water plantains), Butomaceae (flowering rushes), Typhaceae (reedmaces) and Sparganiaceae (bur-reeds) were considered possible sources. The exploitation of the roots and tubers of aquatic plants elsewhere in Mesolithic Europe has also been hypothesised, with plant remains of eelgrass, horned pondweed and beaked tassel weed identified at Argus Bank in Denmark[93] and a number of aquatic plants, including the submerged/floating species yellow water lily (*Nuphar lutea*), white water lily (*Nymphaea alba*) and pondweed (*Potamogeton pectinatus*), at Star Carr in

England[94]. Interestingly, the chemical profile of C_{19} to C_{29} *n*-alkanes, maximising at C_{23} is consistent with a submerged aquatic plant (e.g. *Potamogeton* spp. (pondweed)[89,91] at Cabeço do Pez, rather than an emergent macrophyte such as those in an Iberian context cited above. Further work would be needed to establish the macrophytes, such as *Potamogeton* spp., likely to have been available at Cabeço do Pez in order to determine possible sources.

The stable isotope data from Cabeço do Pez have been interpreted as indicating a largely terrestrial diet (animals and vegetables), with some marine protein[79]. Notably here, the isotope data did not reveal evidence for freshwater protein[79] and from this it was inferred that there was no contribution of freshwater resources[79]. However, aquatic plants have previously been shown to exhibit significant variations in their stable isotopic composition ($\delta^{13}C$ and $\delta^{15}N$), particularly submerged species such as *Potamogeton* spp.[128]. With typical $\delta^{13}C$ values of ~-23 to -31‰ and $\delta^{15}N$ values of ~-1 to +7‰, these can be equivalent to a C_3 terrestrial fauna input[129] and when combined with a small (~20-30%) marine protein component in the diet, as reasonably inferred[79], can produce values consistent with those observed at Cabeço do Pez, including the individual No.27 ($\delta^{13}C$ = -19.5 and $\delta^{15}N$ = 10.0) who was also part of the study presented here. This perhaps illustrates the value of a multiple approach to dietary reconstruction, and the dangers of an overreliance on stable isotopes alone (whether bulk or compound specific data). Significantly, perhaps, the stable isotope study at Cabeço do Pez showed no significant differences between males and females[79], which is consistent (regardless of the exact composition of the diet) with the similarity of the biomolecular profiles obtained from the calculus samples from the female and two males, i.e. the dominance of the *n*-alkanes from the presumed epicuticular wax component of the same submerged aquatic plant.

[Due to the common use of paraffin wax in archaeological excavations in the 19th and 20th centuries during the periods excavations at Cabeço do Pez took place, combined with the known use during the excavations at archaeological sites in the Sado Valley[79,128], it is sensible to take this into account when interpreting the biomolecular data. In this context, it should be said that paraffin wax can have a similar chemical profile, but with a CPI of ≤1.0 and typically C_{20} to C_{33} *n*-alkanes, maximising at C_{24}-C_{26}[130], with slightly more volatile fractions typically C_{19}/C_{20} to C_{32}/C_{33}, maximising at C_{23}[130,131], they are significantly different in molecular composition to those observed in the calculus samples. Most notably, paraffin

waxes have a wider carbon number range than those seen in the calculus samples, with a more even distribution, and small but identifiable quantities of branched alkanes. The *n*-alkanes are highly resilient and would not be expected to degrade to the point of their absence in a matter of decades, as might be the case post-excavation and subsequent museum storage if employed in excavations. Indeed, in practice, even when exposed to heat and high light levels, paraffin wax employed in early 20th century archaeological excavations, notably less environmentally favourable than can be expected in the Sado Valley, is chemically unchanged after almost a century[119]. The average C_{23} maximum, combined with the variable and relatively narrow *n*-alkane carbon number range in the calculus samples (C_{21} to C_{26} >2% *cf.* C_{20} to C_{29} >2% for paraffin wax), with no branched alkanes detected, identifies biodegraded submerged aquatic plant (macrophyte) material. In addition, it should be noted that the 'P_{aq} values' for paraffin waxes of ≤0.91 are significantly different from those for the three calculus samples, which also excludes it as a possible source here. It is also important to note that carbon stable isotopic analysis was carried out successfully on material from Cabeço do Pez[79], specifically on one of the samples presented here (No.27), presumably because those burials with paraffin wax applied during the excavations were avoided, which would, necessarily, also apply to the calculus samples, thereby avoiding any possible contamination.]

Netherlands

Schipluiden and Hardinxveld, Netherlands

Schipluiden is located some 7 km beyond the present coastline – in an extensive beach plain in between the oldest beach barrier (Voorburg-Rijswijk in Zuid Holland) and the back marshes. The settlement of Schipluiden lies on the top of a low dune. The absolute chronology of the settlement was established by means of stratified radiocarbon dates (obtained for samples of various materials) – giving a time span of 3650 until 3380 cal. years BC and as such associated with the Middle Neolithic. Many thousands of features including: wells, hearths, postholes, graves and fences reflect the long-term, intensive use of the entire dune. Wells or watering places were the most numerous. Diversity in the landscape was demonstrated throughout the occupation phases. The high salt marsh and possibly low dunes in the surroundings offered possibilities for small-scale agriculture. Emmer and naked barley

were the two cultivated crops. Besides these cereals, the diet comprised a broad spectrum of gathered wild plants, including fruits and berries, and roots and tubers. Two species were collected to a greater extend, namely sloe plums and crab apples[132].

Hardinxveld-Giessendam De Bruin is one of many river dune sites located in the Lower Rhine-Meuse river delta in the western Netherlands. This site and the neighbouring Hardinxveld-Giessendam Polderweg were inhabited during the Late Mesolithic (5th millennium BC), but the use of De Bruin, in contrast to Polderweg, continued into the Early Neolithic (Early Swifterbant). Three phases of occupation were distinguished at the De Bruin site. Phase 1, the oldest settlement, dated to c. 5500 to 5300 cal BC, Phase 2, witnessing the introduction of pottery, dated to c. 5100 to 4800 cal BC, and Phase 3, when the first domestic animals appeared at the site, dated to c. 4700 to 4450 cal BC[133,134].

The river dunes of De Bruin and Polderweg were situated in an area with slowly flowing rivers with low banks and extensive marsh vegetation dominated by reed and sedges. A variety of nuts, fruits, and berries were collected and processed for food, as indicated by their charred remains from Hardinxveld-Giessendam Polderweg and Hardinxveld-Giessendam De Bruin, including hazelnut, water chestnut, acorn, wild apple, hawthorn, dogwood, and possibly guelder rose. Numerous (uncharred) seeds of yellow water-lily (*Nuphar lutea*) and white water-lily (*Nymhaea alba*) indicate the presence of both species in the waters near the sites, and suggest that seeds of both species might have been processed for food. Starchy root tubers of lesser celandine (*Ranunculus ficaria*, syn. *Ficaria verna*) were also likely used as food, as suggested by the charred remains from Hardinxveld-Giessendam Polderweg. The human skeletal remains are dated between the end of Phase 1 and beginning of Phase2[133,134].

Ten samples of dental calculus taken from seven individuals from two sites in the Netherlands were analysed by sequential thermal desorption-gas chromatography-mass spectrometry (TD-GC-MS) and pyrolysis-gas chromatography-mass spectrometry (Py-GC-MS). Four samples of soil/burial sediment from several of the graves were also analysed. This technique facilitates the identification of both free/unbound and bound/polymeric organic components. All ten calculus samples and four soil/sediment samples produced greater amounts of a bound/polymeric organic material compared to the free organic material present, although both fractions were highly oxidised in nature.

69. Schipluiden Grave 1 Individual 1 TMS0081: maxilla labial molar (calculus: 15.19 mg)

The thermal desorption total ion chromatogram (TIC) revealed carbon dioxide, sulphur dioxide and carbon disulphide as the only detectable, albeit relatively abundant, components. No other free, thermally extractable, organic components could be detected in this sample. It should be noted, however, that highly polar material could have been present, which would not have successfully eluted from the column, or indeed volatilised sufficiently in the probe. It is not possible to determine the significance of the carbon dioxide, beyond it being indicative of oxidised organic material. The sulphur dioxide and carbon disulphide are also difficult to interpret, although these species can be indicative of oxidised and pyrolysed sulphur-containing amino acids or sulphones[135,136] (see discussion). Sulphur dioxide is also known to be a major pyrolysis product of lignosulphonates[137], and its organic nature would facilitate sulphur dioxide generation at this relatively low temperature, although this possibility in this context is probably less likely (again, see discussion).

The pyrolysis-GC-MS TIC (Fig. 43) was dominated by carbon dioxide, 1-butene and 1-pentene, the alkenes being indicative of lipid or proteinaceous material in the sample[11]. As observed in the TD profile, sulphur dioxide and carbon disulphide were also major components. However, these were also accompanied by carbonyl sulphide and the aromatic sulphur compound thiophene and its methyl and dimethyl derivatives. The presence of thiophene with the sulphur dioxide, carbonyl sulphide and carbon disulphide can be indicative of pyrolysed sulphur-containing amino acids[135,136,138] (see discussion). The presence of acetic acid as a major component is also consistent with sulphur-containing amino acids[135]. Notably, sulphur as its cyclic octaatomic S_8 form was also detected. More specifically, thiophene and its C_1 and C_2 alkyl derivatives are pyrolysis products of cysteine and cystine[135,136,138], and elemental sulphur as S_8 has also been posited as a pyrolysis product of the sulphur-rich cystine[139].

In contrast to the thermal desorption profile, the pyrogram also revealed a complex suite of organic compounds, albeit in relatively minor abundance. These include a series of C_{10} to C_{16} *n*-alkanes, typical of saturated fat/oil-derived acyl lipids[5,6], although the absence of the *n*-1-alkene counterparts may well reflect diagenetic changes, rather be indicative of a dominance of saturated acyl lipids in the original fat/oil. Indeed, the presence of C_5 to C_9 *n*-1-alkenes in relatively significant abundance compared to the longer carbon chain *n*-alkane moieties is indicative of an appreciable unsaturated acyl lipid component. The absence of these

compounds in the TD profile provides molecular evidence for a polymeric/bound acyl lipid source. However, in addition to *n*-1-alkenes and *n*-alkanes, typical of fat/oil derived lipids, the pyrogram also displayed a series of C_9 to C_{15} 2-methylalkanes (isoalkanes), presumably deriving from the branched fatty acids which are significant components of bacterial triglycerides (again, the absence of their iso-methyl-1-alkenes (isoalkenes) counterparts may well reflect diagenetic changes, rather be indicative of a dominance of saturated branched acyl lipids in the original lipid source). Although non-specific, the presence of carbon dioxide is also consistent with a (highly oxidised) lipid component in this sample.

The pyrolysate TIC identified components indicative of ‘black carbon’, i.e. charcoal or soot[7-10]. Of these, benzene and toluene were major compounds present in the ‘char’ pyrolysate (~30% and 60% of total quantified pyrogram peak area respectively), along with moderate quantities of ethyl benzene and o-, m- and p-xylenes (~8% total). The relative dominance of toluene, rather than the normally dominant aromatic hydrocarbon benzene, is somewhat atypical of chars[7-10]. However, the presence of naphthalene, combined with their relative abundances, is indicative of a ‘char’ component providing molecular evidence for likely exposure to fire/cooking. The markers identified here can also be indicative of proteins[6-11] and combined with the related protein markers pyridine, benzonitrile and benzyl nitrile (phenylacetonitrile) identified this is indicative of these compounds, at least in part, originating from a protein-derived component[6-11]. It should also be noted that highly polar material could have been present, which would not have successfully eluted from the column, or indeed volatilised sufficiently in the probe.

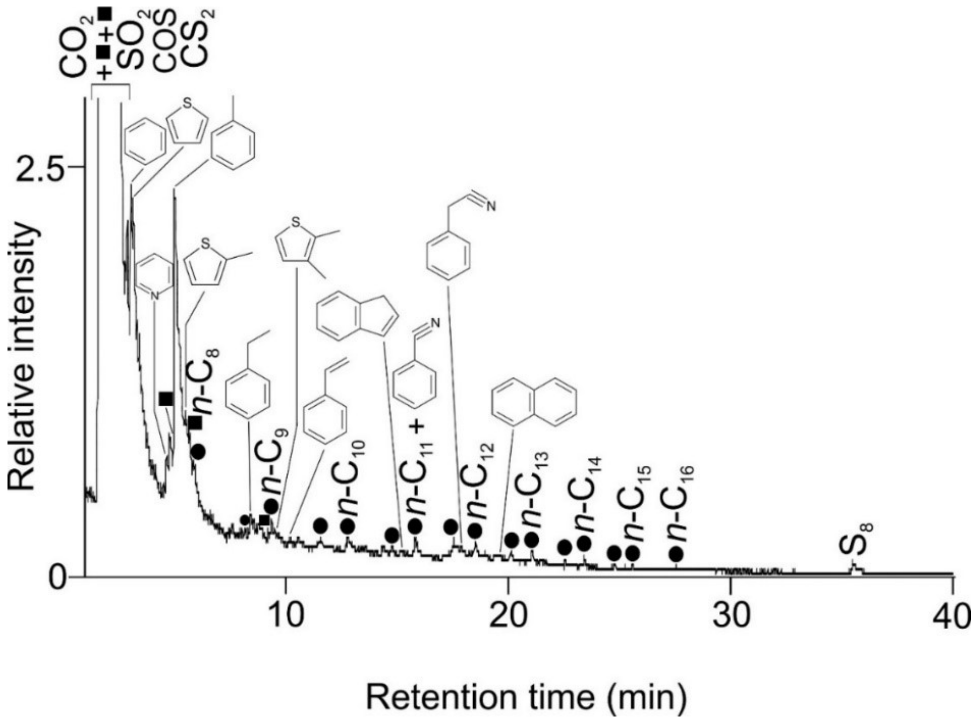

Supplementary Figure 43. Reconstructed total ion chromatogram of the pyrogram (pyrolysis profile) (610 °C for 10s), after thermal desorption (310 °C for 10 s) of sample TMS0081 Individual 1 Schipluiden Grave 1. Key to Fig. 43: Peak identities (x indicates carbon chain length): filled squares, Cx indicates alkenes; filled circles, Cx indicates alkanes. Also shown are the structures of thirteen aromatic compounds identified: benzene, thiophene, pyridine, toluene, methylthiophene, ethylbenzene, dimethylthiophene, styrene, indene, benzonitrile, benzyl nitrile and naphthalene. CO_2 indicates carbon dioxide, SO_2 indicates sulphur dioxide, COS indicates carbonyl sulphide, CS_2 indicates carbon disulphide and S_8 indicates cyclic octaatomic sulphur.

70. Schipluiden Grave 1 Individual 2 TMS0077: mandible labial right PM1 (calculus: 4.22 mg)

The thermal desorption total ion chromatogram (TIC) revealed carbon dioxide, 1-butene, sulphur dioxide and carbon disulphide as the only detectable components. No other free, thermally extractable, organic components could be detected in this sample. It should be noted, however, that highly polar material could have been present, which would not have successfully eluted from the column, or indeed volatilised sufficiently in the probe. It is not possible to determine the significance of the carbon dioxide, beyond it being indicative of

oxidised organic material. The dominance of 1-butene can be indicative of lipid or proteinaceous material in the sample[11], and perhaps produced at this relatively low temperature due to matrix effects. The sulphur dioxide is difficult to interpret, although it can be indicative of oxidised and pyrolysed sulphur-containing amino acids or sulphones[135,136] (see discussion). Sulphur dioxide is also known to be a major pyrolysis product of lignosulphonates[137], and its organic nature would facilitate sulphur dioxide generation at this relatively low temperature, although this possibility in this context is probably less likely (again, see discussion).

The pyrolysis-GC-MS TIC was dominated by carbon dioxide, 1-butene and 1-pentene, the alkenes being indicative of lipid or proteinaceous material in the sample[11]. As observed in the TD profile, sulphur dioxide was a significant component. However, like the pyrogram of the previous sample, it was accompanied by carbonyl sulphide (trace) and carbon disulphide, which collectively can be indicative of sulphur-containing amino acids or sulphones[135,136] (see discussion). Thiophene was also detected, albeit only as a trace component; this can again can be indicative of pyrolysed sulphur-containing amino acids[135,136,138] although the relative abundance here makes any interpretation very tentative. The presence of acetic acid as a major component in this sample is also consistent with sulphur-containing amino acids[135]. The pyrolysate TIC identified benzene and toluene as the only other components detected although there very minor abundance and an absence of other potentially more diagnostic biomarkers mean their significance is unclear. It should also be noted that highly polar material could have been present, which would not have successfully eluted from the column, or indeed volatilised sufficiently in the probe.

71. Schipluiden Grave 1 Individual 2 TMS0077: maxilla/mandible? lingual left incisor 1-2 (calculus: 5.89 mg)

The thermal desorption total ion chromatogram (TIC) revealed carbon dioxide, 1-butene (trace) and sulphur dioxide as the only detectable components. No other free, thermally extractable, organic components could be detected in this sample. It should be noted, however, that highly polar material could have been present, which would not have successfully eluted from the column, or indeed volatilised sufficiently in the probe. It is not possible to determine the significance of the carbon dioxide, beyond it being indicative of oxidised organic material. The 1-butene can be indicative of lipid or proteinaceous material in

the sample[11], and perhaps produced at this relatively low temperature due to matrix effects. The sulphur dioxide is difficult to interpret, although it can be indicative of oxidised and pyrolysed sulphur-containing amino acids or sulphones[135,136] (see discussion). Sulphur dioxide is also known to be a major pyrolysis product of lignosulphonates[137], and its organic nature would facilitate sulphur dioxide generation at this relatively low temperature, although this possibility in this context is probably less likely (again, see discussion).

The pyrolysis-GC-MS TIC was dominated by carbon dioxide, 1-butene and 1-pentene, the alkenes being indicative of lipid or proteinaceous material in the sample[11]. As observed in the TD profile, sulphur dioxide was a significant component. However, like the pyrogram of the two previous samples, it was accompanied by carbonyl sulphide and carbon disulphide, which collectively can be indicative of sulphur-containing amino acids or sulphones[135,136] (see discussion). Thiophene was also detected, albeit only as a very minor component; this can again can be indicative of pyrolysed sulphur-containing amino acids[135,136,138] although the relative abundance here makes any interpretation somewhat tentative. The pyrolysate TIC identified benzene and toluene as the only other components detected although there very minor abundance and an absence of other potentially more diagnostic biomarkers mean their significance is unclear. It should also be noted that highly polar material could have been present, which would not have successfully eluted from the column, or indeed volatilised sufficiently in the probe.

72. Schipluiden Grave 1 Individual 2 TMS0077: maxilla/mandible? lingual right M2-3 (calculus: 19.28 mg)

The thermal desorption total ion chromatogram (TIC) revealed carbon dioxide, 1-butene, acetone, sulphur dioxide, carbonyl sulphide and carbon disulphide as the only detectable components. No other free, thermally extractable, organic components could be detected in this sample. It should be noted, however, that highly polar material could have been present, which would not have successfully eluted from the column, or indeed volatilised sufficiently in the probe. It is not possible to determine the significance of the carbon dioxide, beyond it being indicative of oxidised organic material. The 1-butene can be indicative of lipid or proteinaceous material in the sample[11], and perhaps produced at this relatively low temperature due to matrix effects. Collectively, the sulphur dioxide, carbonyl sulphide and carbon disulphide can be indicative of oxidised and pyrolysed sulphur-containing amino

acids or sulphones[135,136] (see discussion). Sulphur dioxide is also known to be a major pyrolysis product of lignosulphonates[137], and its organic nature would facilitate sulphur dioxide generation at this relatively low temperature, although this possibility in this context is probably less likely (again, see discussion).

The pyrolysis-GC-MS TIC (Fig. 44) was dominated by carbon dioxide, 1-butene and 1-pentene, the alkenes being indicative of lipid or proteinaceous material in the sample[11]. As observed in the TD profile, sulphur dioxide, carbonyl sulphide and carbon disulphide were also significant components. However, these were also accompanied by the aromatic sulphur compound thiophene and two methyl derivatives. The presence of thiophene with the sulphur dioxide, carbonyl sulphide and carbon disulphide can be indicative of pyrolysed sulphur-containing amino acids[135,136,138] (see discussion). The presence of acetic acid as a major component is also consistent with sulphur-containing amino acids[135]. Notably, sulphur as its cyclic octaatomic S_8 form was also detected. More specifically, thiophene and its C_1 and C_2 alkyl derivatives are pyrolysis products of cysteine and cystine[135,136,138,] and elemental sulphur as S_8 has also been posited as a pyrolysis product of the sulphur-rich cystine[139].

In contrast to the thermal desorption profile, the pyrogram also revealed a complex suite of organic compounds, albeit in minor abundance. These include a series of C_{10} to C_{15} *n*-alkanes, typical of saturated fat/oil-derived acyl lipids[5,6], although the absence of the *n*-1-alkene counterparts may well reflect diagenetic changes, rather be indicative of a dominance of saturated acyl lipids in the original fat/oil. Indeed, the presence of C_5 to C_9 *n*-1-alkenes in relatively significant abundance compared to the longer carbon chain *n*-alkane moieties is indicative of an appreciable unsaturated acyl lipid component. The absence of these compounds in the TD profile provides molecular evidence for a polymeric/bound acyl lipid source. However, in addition to *n*-1-alkenes and *n*-alkanes, typical of fat/oil derived lipids, the pyrogram also displayed a series of C_9 to C_{14} 2-methylalkanes (isoalkanes), presumably deriving from the branched fatty acids which are significant components of bacterial triglycerides (again, the absence of their iso-methyl-1-alkenes (isoalkenes) counterparts may well reflect diagenetic changes, rather be indicative of a dominance of saturated branched acyl lipids in the original lipid source). Although non-specific, the presence of carbon dioxide is also consistent with a (highly oxidised) lipid component in this sample.

The pyrolysate TIC identified components indicative of 'black carbon', i.e. charcoal or soot[7-10]. Of these, benzene and toluene were major compounds present in the 'char' pyrolysate

(~30% and 65% of total quantified pyrogram peak area respectively), along with moderate quantities of ethyl benzene and o-, m- and p-xylenes (~4% total). The relative dominance of toluene, rather than the normally dominant aromatic hydrocarbon benzene, is somewhat atypical of chars[7-10]. However, their relative abundances are very similar to those observed above and so are at least tentatively indicative of a 'char' component providing molecular evidence for exposure to fire/cooking. It should also be noted that highly polar material could have been present, which would not have successfully eluted from the column, or indeed volatilised sufficiently in the probe.

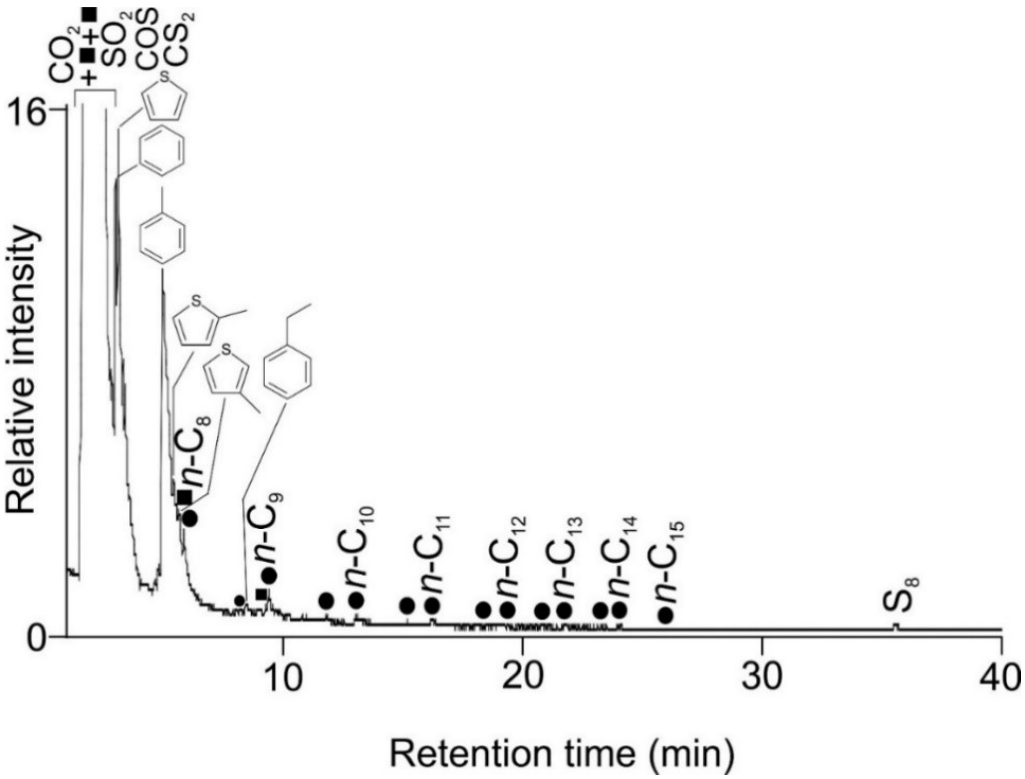

Supplementary Figure 44. Reconstructed total ion chromatogram of the pyrogram (pyrolysis profile) (610 °C for 10s), after thermal desorption (310 °C for 10 s) of sample TMS0077 (M2-3) Individual 2 Schipluiden Grave. Key to Fig. 44: Peak identities (x indicates carbon chain length): filled squares, Cx indicates alkenes; filled circles, Cx indicates alkanes. Also shown are the structures of six aromatic compounds identified: benzene, thiophene, toluene, methylthiophene (x2) and ethylbenzene. CO_2 indicates carbon dioxide, SO_2 indicates sulphur dioxide, COS indicates carbonyl sulphide, CS_2 indicates carbon disulphide and S_8 indicates cyclic octaatomic sulphur.

Schipluiden soil 'control' from Grave 1, Individuals 1 and 2 (soil/sediment: 5.28 mg)

The thermal desorption total ion chromatogram (TIC) revealed carbon dioxide, sulphur dioxide, carbonyl sulphide and carbon disulphide as the only detectable components. No other free, thermally extractable, organic components could be detected in this sample. It should be noted, however, that highly polar material could have been present, which would not have successfully eluted from the column, or indeed volatilised sufficiently in the probe. It is not possible to determine the significance of the carbon dioxide, beyond it being indicative of oxidised organic material. The sulphur dioxide, carbonyl sulphide and carbon disulphide can be indicative of oxidised and pyrolysed sulphur-containing amino acids or sulphones[135,136] (see discussion). Sulphur dioxide is also known to be a major pyrolysis product of lignosulphonates[137], and its organic nature would facilitate sulphur dioxide generation at this relatively low temperature, although this possibility in this context is probably less likely (again, see discussion). In this context, these sulphur-containing moieties derive from the burial soil/sediment (see discussion).

The pyrolysis-GC-MS TIC was dominated by carbon dioxide, with sulphur dioxide, carbonyl sulphide and carbon disulphide the only other significant components. Collectively these latter three compounds can be indicative of sulphur-containing amino acids or sulphones[135,136] (see discussion), which suggests they derive from the burial soil/sediment (see discussion). The pyrolysate TIC identified benzene as the only other component detected although its trace abundance means its significance is unclear. It should also be noted that highly polar material could have been present, which would not have successfully eluted from the column, or indeed volatilised sufficiently in the probe.

73. Schipluiden Grave 3 TMS0058: lower jaw-mandible labial molar (calculus: 13.58 mg)

The thermal desorption total ion chromatogram (TIC) revealed carbon dioxide, sulphur dioxide, carbonyl sulphide and carbon disulphide as the only detectable, albeit relatively abundant, components. No other free, thermally extractable, organic components could be detected in this sample. It should be noted, however, that highly polar material could have been present, which would not have successfully eluted from the column, or indeed volatilised sufficiently in the probe. It is not possible to determine the significance of the carbon dioxide, beyond it being indicative of oxidised organic material. Collectively, the

sulphur dioxide, carbonyl sulphide and carbon disulphide can be indicative of oxidised and pyrolysed sulphur-containing amino acids or sulphones[135,136] (see discussion). Sulphur dioxide is also known to be a major pyrolysis product of lignosulphonates[137], and its organic nature would facilitate sulphur dioxide generation at this relatively low temperature, although this possibility in this context is probably less likely (again, see discussion).

The pyrolysis-GC-MS TIC (Fig. 45) was dominated by carbon dioxide, 1-butene and 1-pentene, the alkenes being indicative of lipid or proteinaceous material in the sample[11]. As observed in the TD profile, sulphur dioxide, carbonyl and carbon disulphide were also significant components. However, these were also accompanied by the aromatic sulphur compound thiophene and its methyl and dimethyl derivatives. The presence of thiophene with the sulphur dioxide, carbonyl sulphide and carbon disulphide can be indicative of pyrolysed sulphur-containing amino acids[135,136,138] (see discussion). The presence of acetic acid as a significant component is also consistent with sulphur-containing amino acids[135]. Notably, sulphur as its cyclic octaatomic S_8 form was also detected. More specifically, thiophene and its C_1 and C_2 alkyl derivatives are pyrolysis products of cysteine and cystine[135,136,138], and elemental sulphur as S_8 has also been posited as a pyrolysis product of the sulphur-rich cystine[139].

In contrast to the thermal desorption profile, the pyrogram also revealed a complex suite of organic compounds, albeit in relatively minor abundance. These include a series of C_7 to C_{18} *n*-1-alkenes and *n*-alkanes, typical of unsaturated and saturated fat/oil-derived acyl lipids[5,6]. The absence of these compounds in the TD profile provides molecular evidence for a polymeric/bound acyl lipid source. However, in addition to *n*-1-alkenes and *n*-alkanes, typical of fat/oil derived lipids, the pyrogram also displayed a series of C_8 to C_{16} iso-methyl-1-alkenes (isoalkenes) and 2-methylalkanes (isoalkanes), presumably deriving from the branched fatty acids which are significant components of bacterial triglycerides. Although non-specific, the presence of carbon dioxide is also consistent with a (highly oxidised) lipid component in this sample.

The pyrolysate TIC identified components indicative of 'black carbon', i.e. charcoal or soot[7-10]. Of these, benzene and toluene were major compounds present in the 'char' pyrolysate (~25% and 65% of total quantified pyrogram peak area respectively), along with moderate quantities of ethyl benzene and o-, m- and p-xylenes (~10% total). The relative dominance of toluene, rather than the normally dominant aromatic hydrocarbon benzene, is somewhat

atypical of chars[7-10]. However, the presence of naphthalene, combining with their relative abundances, is indicative of a 'char' component providing molecular evidence for exposure to fire/cooking. The markers identified here can also be indicative of proteins[6-11] and combined with the related protein markers pyridine, benzonitrile and benzyl nitrile (phenylacetonitrile) identified this is indicative of these compounds, at least in part, originating from a protein-derived component[6-11]. It should also be noted that highly polar material could have been present, which would not have successfully eluted from the column, or indeed volatilised sufficiently in the probe.

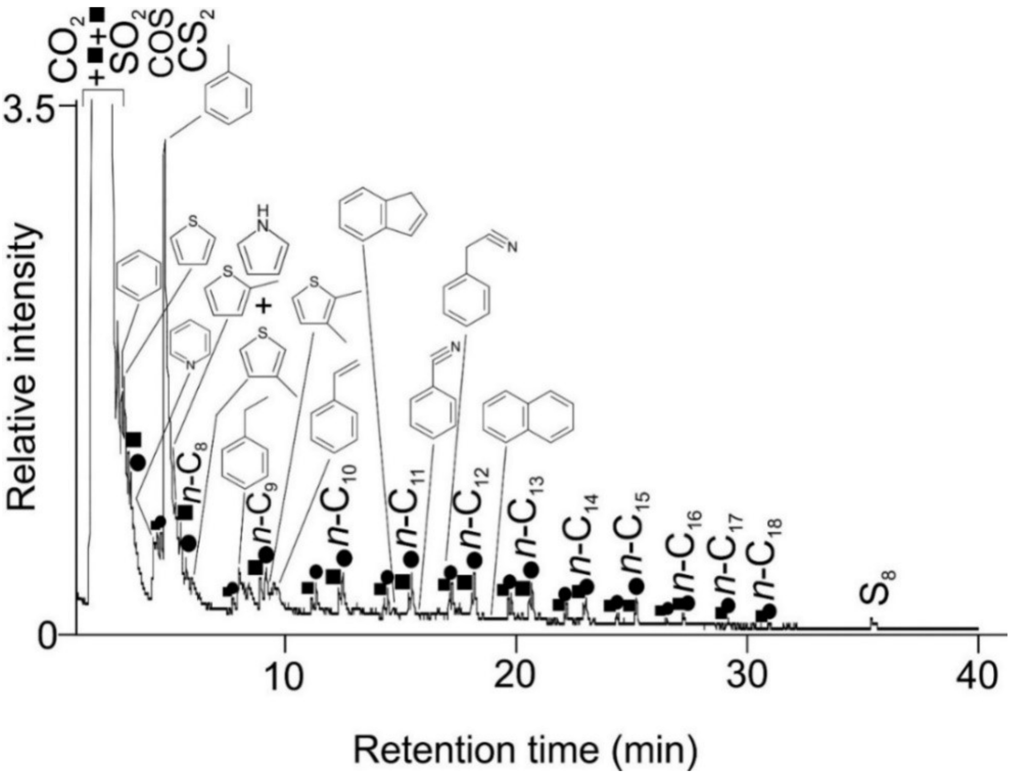

Supplementary Figure 45. Reconstructed total ion chromatogram of the pyrogram (pyrolysis profile) (610 °C for 10s), after thermal desorption (310 °C for 10 s) for sample TMS0058 (lower labial molar) Schipluiden Grave 3. Key to Fig. 45: Peak identities (x indicates carbon chain length): filled squares, Cx indicates alkenes; filled circles, Cx indicates alkanes. Also shown are the structures of fourteen aromatic compounds identified: benzene, thiophene, pyridine, toluene, methylthiophene (x2), pyrrole, ethylbenzene, dimethylthiophene, styrene, indene, benzonitrile, benzyl nitrile and naphthalene. CO_2 indicates carbon dioxide, SO_2 indicates sulphur dioxide, COS indicates carbonyl sulphide, CS_2 indicates carbon disulphide and S_8 indicates cyclic octaatomic sulphur.

74. Schipluiden Grave 3 TMS0058: upper jaw-maxilla labial molar (v. worn) (calculus: 14.64 mg)

The thermal desorption total ion chromatogram (TIC) revealed carbon dioxide, sulphur dioxide, carbonyl sulphide and carbon disulphide as the only detectable components. No other free, thermally extractable, organic components could be detected in this sample. It should be noted, however, that highly polar material could have been present, which would not have successfully eluted from the column, or indeed volatilised sufficiently in the probe. It is not possible to determine the significance of the carbon dioxide, beyond it being indicative of oxidised organic material. Collectively, the sulphur dioxide, carbonyl sulphide and carbon disulphide can be indicative of oxidised and pyrolysed sulphur-containing amino acids or sulphones[135,136] (see discussion). Sulphur dioxide is also known to be a major pyrolysis product of lignosulphonates[137], and its organic nature would facilitate sulphur dioxide generation at this relatively low temperature, although this possibility in this context is probably less likely (again, see discussion).

The pyrolysis-GC-MS TIC (Fig. 46) was dominated by carbon dioxide, 1-butene and 1-pentene, the alkenes being indicative of lipid or proteinaceous material in the sample[11]. As observed in the TD profile, sulphur dioxide, carbonyl sulphide and carbon disulphide were also significant components. However, these were also accompanied by the aromatic sulphur compound thiophene and a methyl derivative. The presence of thiophene with the sulphur dioxide, carbonyl sulphide and carbon disulphide can be indicative of pyrolysed sulphur-containing amino acids[135,136,138] (see discussion). The presence of acetic acid as a major component is also consistent with sulphur-containing amino acids[135]. Notably, sulphur as its cyclic octaatomic S_8 form was also detected. More specifically, thiophene and its C_1 and C_2 alkyl derivatives are pyrolysis products of cysteine and cystine[135,136,138], and elemental sulphur as S_8 has also been posited as a pyrolysis product of the sulphur-rich cystine[139].

In contrast to the thermal desorption profile, the pyrogram also revealed a complex suite of organic compounds, albeit in relatively minor abundance. These include a series of C_{10} to C_{17} *n*-alkanes, typical of saturated fat/oil-derived acyl lipids[5,6], although the absence of the *n*-1-alkene counterparts may well reflect diagenetic changes, rather be indicative of a dominance of saturated acyl lipids in the original fat/oil. Indeed, the presence of C_5 to C_9 *n*-1-alkenes in relatively significant abundance compared to the longer carbon chain *n*-alkane moieties is

indicative of an appreciable unsaturated acyl lipid component. The absence of these compounds in the TD profile provides molecular evidence for a polymeric/bound acyl lipid source. However, in addition to *n*-1-alkenes and *n*-alkanes, typical of fat/oil derived lipids, the pyrogram also displayed a series of C_{10} to C_{15} 2-methylalkanes (isoalkanes), presumably deriving from the branched fatty acids which are significant components of bacterial triglycerides (again, the absence of their iso-methyl-1-alkenes (isoalkenes) counterparts may well reflect diagenetic changes, rather be indicative of a dominance of saturated branched acyl lipids in the original lipid source). Although non-specific, the presence of carbon dioxide is also consistent with a (highly oxidised) lipid component in this sample.

The pyrolysate TIC identified components indicative of 'black carbon', i.e. charcoal or soot[7-10]. Of these, benzene and toluene were major compounds present in the 'char' pyrolysate (~40% and 55% of total quantified pyrogram peak area respectively), along with moderate quantities of ethyl benzene and o-, m- and p-xylenes (~3% total). The relative dominance of toluene, rather than the normally dominant aromatic hydrocarbon benzene, is somewhat atypical of chars[7-10]. However, their relative abundances are similar to those observed above and so are at least tentatively indicative of a 'char' component providing molecular evidence for exposure to fire/cooking. It should also be noted that highly polar material could have been present, which would not have successfully eluted from the column, or indeed volatilised sufficiently in the probe.

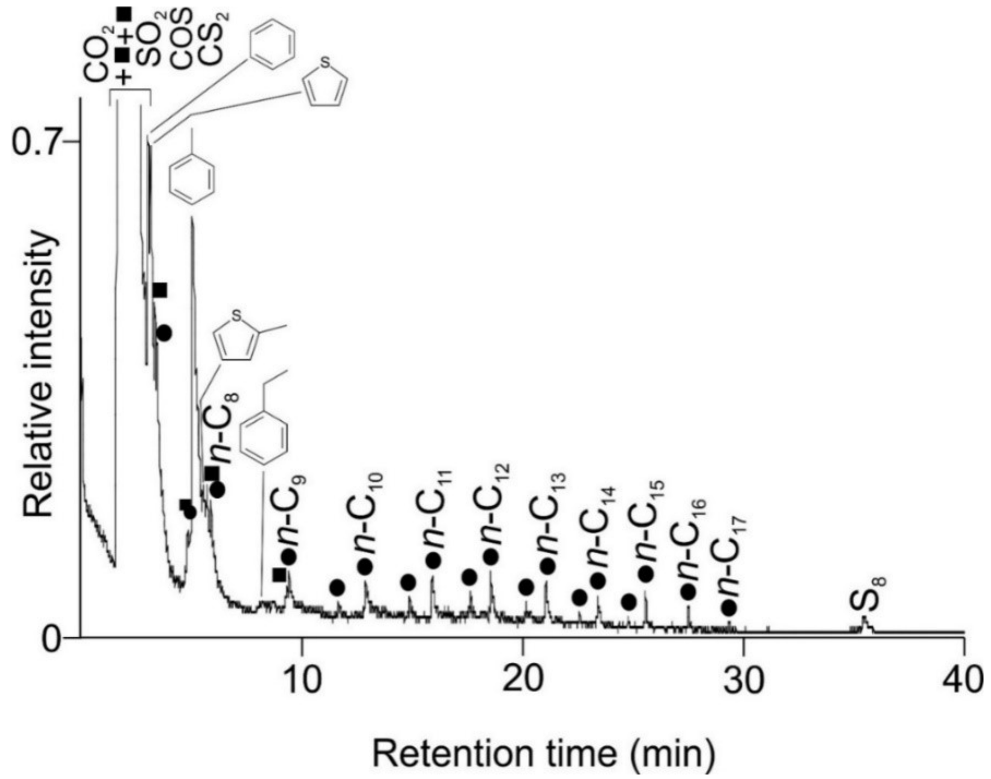

Supplementary Figure 46. Reconstructed total ion chromatogram of the pyrogram (pyrolysis profile) (610 °C for 10s), after thermal desorption (310 °C for 10 s) of sample TMS0058 (upper labial molar) Schipluiden Grave 3). Key to Fig. 46: Peak identities (x indicates carbon chain length): filled squares, Cx indicates alkenes; filled circles, Cx indicates alkanes. Also shown are the structures of five aromatic compounds identified: benzene, thiophene, toluene, methylthiophene and ethylbenzene. CO_2 indicates carbon dioxide, SO_2 indicates sulphur dioxide, COS indicates carbonyl sulphide, CS_2 indicates carbon disulphide and S_8 indicates cyclic octaatomic sulphur.

75. Schipluiden Grave 5 child TMS0051: maxilla labial PM1 (calculus: 3.54 mg)

The thermal desorption total ion chromatogram (TIC) revealed carbon dioxide, sulphur dioxide, carbonyl sulphide and carbon disulphide as the only detectable components. No other free, thermally extractable, organic components could be detected in this sample. It should be noted, however, that highly polar material could have been present, which would not have successfully eluted from the column, or indeed volatilised sufficiently in the probe. It is not possible to determine the significance of the carbon dioxide, beyond it being indicative of oxidised organic material. Collectively, the sulphur dioxide, carbonyl sulphide

and carbon disulphide can be indicative of oxidised and pyrolysed sulphur-containing amino acids or sulphones[135,136] (see discussion). Sulphur dioxide is also known to be a major pyrolysis product of lignosulphonates[137], and its organic nature would facilitate sulphur dioxide generation at this relatively low temperature, although this possibility in this context is probably less likely (again, see discussion).

The pyrolysis-GC-MS TIC revealed carbon dioxide, cyclopentadiene, sulphur dioxide and carbonyl sulphide as the only detectable components. The presence of sulphur dioxide and carbonyl sulphide can be indicative of pyrolysed sulphur-containing amino acids or sulphones[135,136], although the absence of more diagnostic sulphur-containing organic species makes any connection somewhat tentative. It is not possible to determine the significance of the carbon dioxide, beyond it being indicative of oxidised organic material. It should also be noted that highly polar material could have been present, which would not have successfully eluted from the column, or indeed volatilised sufficiently in the probe.

Schipluiden soil 'control' from Grave 5, child (soil/sediment: 8.75 mg)

The thermal desorption total ion chromatogram (TIC) was dominated by carbon dioxide, sulphur dioxide and carbon disulphide. The presence of sulphur dioxide and carbonyl sulphide can be indicative of pyrolysed sulphur-containing amino acids or sulphones[135,136]. The presence of acetic acid as a major component is also consistent with sulphur-containing amino acids[135]. Notably, sulphur as its cyclic octaatomic S_8 form was also detected as a very minor constituent, which has been posited as a pyrolysis product of the sulphur-rich cystine[139]. 3(2H)-pyridazinone was also detected as a minor constituent. It should be noted, however, that highly polar material could have been present, which would not have successfully eluted from the column, or indeed volatilised sufficiently in the probe. It is not possible to determine the significance of the carbon dioxide, beyond it being indicative of oxidised organic material.

The pyrolysis-GC-MS TIC (Fig. 47) was dominated by carbon dioxide, with sulphur dioxide, carbonyl sulphide and carbon disulphide the only other major components. Collectively these latter three compounds can be indicative of sulphur-containing amino acids or sulphones[135,136] (see discussion), which suggests they derive from the burial soil/sediment (see discussion). These sulphur compounds were also accompanied by the aromatic sulphur compound thiophene. The presence of thiophene with the sulphur dioxide, carbonyl sulphide

and carbon disulphide can be indicative of pyrolysed sulphur-containing amino acids[135,136,138] (see discussion).

In contrast to the thermal desorption profile, the pyrogram also revealed a complex suite of organic compounds, albeit in relatively minor abundance. These include a series of C_{11} to C_{25} *n*-1-alkenes and *n*-alkanes, typical of unsaturated and saturated fat/oil-derived acyl lipids[5,6]. The absence of these compounds in the TD profile provides molecular evidence for a polymeric/bound acyl lipid source in the sediment of Grave 5. Interestingly, there were no bacterially-derived hydrocarbons deriving from the original branched fatty acid acyl groups which are significant components of bacterial triglycerides, indicating this lipid component is not bacterial in origin. Although non-specific, the presence of carbon dioxide is also consistent with a (highly oxidised) lipid component in this sample.

The pyrolysate TIC identified benzene and toluene as the only other components detected although in the absence of more diagnostic compounds their significance here is unclear. It should also be noted that highly polar material could have been present, which would not have successfully eluted from the column, or indeed volatilised sufficiently in the probe.

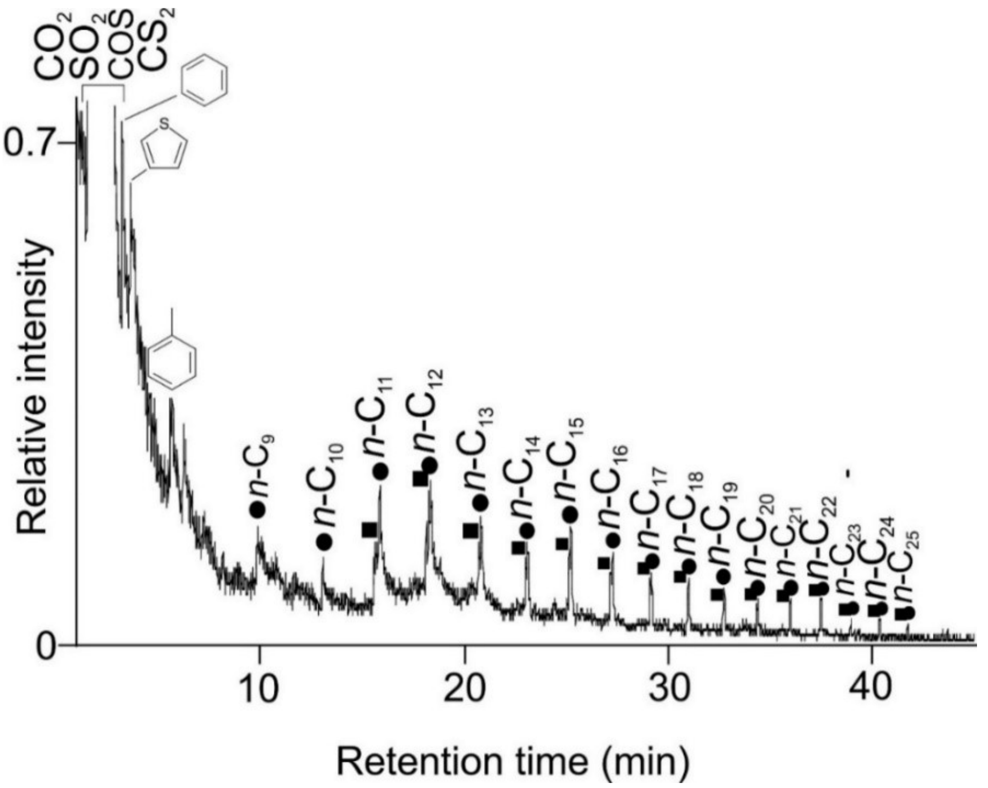

Supplementary Figure 47. Reconstructed total ion chromatogram of the pyrogram (pyrolysis profile) (610 °C for 10s), after thermal desorption (310 °C for 10 s) of the soil (control) from Schipluiden Grave 5. Key to Fig. 47: Peak identities (x indicates carbon chain length): filled squares, Cx indicates alkenes; filled circles, Cx indicates alkanes. Also shown are the structures of three aromatic compounds identified: benzene, thiophene and toluene. CO_2 indicates carbon dioxide, SO_2 indicates sulphur dioxide, COS indicates carbonyl sulphide and CS_2 indicates carbon disulphide.

Discussion, Schipluiden burials

Seven samples of dental calculus and two burial 'control' samples of soil from Schipluiden were analysed by sequential thermal desorption-gas chromatography-mass spectrometry (TD-GC-MS) and pyrolysis-gas chromatography-mass spectrometry (Py-GC-MS). The samples contained both free and bound/polymeric organic material, albeit highly oxidised.

Beyond significant abundances of carbon dioxide, carbonyl sulphide, carbon disulphide and sulphur dioxide, the calculus and soil samples contained little free and bound/polymeric organic compounds, most likely reflecting unfavourable prevailing conditions for good biomolecular preservation. The dominance of only a few small organic compounds, primarily carbon dioxide, suggests that only highly oxidised sulphur-rich organic material was present.

Notably, the Netherlands has one of the highest sulphur contents in subsoils[140,141]. The site of Schipluiden is in close proximity to salt marsh and peat[142], which are also known to be high in organic and inorganic sulphur[143,144]. The alkyl thiophenes, in addition to elemental sulphur (as S_8), may be indicative of the amino acids cysteine and cystine, although the presence of other pyrolysis markers consistent with these amino acids in the soil samples (carbonyl sulphide, carbon disulphide and sulphur dioxide) means their presence here may be due to the burial matrix rather than deriving from the dental calculus itself. It is also possible that the sulphur dioxide may, at least in part, derive from the thermal decomposition of sulphuric acid in the probe, which can be expected to take place at the thermal desorption and pyrolysis temperatures employed.

The bound/polymeric acyl lipid component observed in four of the calculus samples from three individuals does not appear in the soil samples from Schipluiden and therefore this indicates a bacterial lipid constituent present in the calculus, which may well derive from the

individuals' own oral microbiomes. Although biomolecular preservation was poor, there was at least tentative organic chemical evidence for exposure to fire/cooking, and within this 'char' the indication of a protein component. Beyond this, there was nothing which could be indicative of dietary input or environmental exposure in any of the Schipluiden samples.

76. Hardinxveld de Bruin, Brui 0011014 p.22 VI.35: Elvis F1004 (Box 0307) mandible lingual left PM2 (calculus: 10.77 mg)

The thermal desorption total ion chromatogram (TIC) revealed carbon dioxide and sulphur dioxide (trace) and benzene as the only detectable components. No other free, thermally extractable, organic components could be detected in this sample. It should be noted, however, that highly polar material could have been present, which would not have successfully eluted from the column, or indeed volatilised sufficiently in the probe. It is not possible to determine the significance of the carbon dioxide, beyond it being indicative of oxidised organic material. The sulphur dioxide is in too low relative abundance to make and meaningful interpretation of its presence here and benzene is too ubiquitous to assign any origin in the absence of other associated and more diagnostic biomarkers.

The pyrolysis-GC-MS TIC (Fig. 48) was dominated by carbon dioxide, 1-butene and 1-pentene, the alkenes being indicative of lipid or proteinaceous material in the sample[11]. As observed in the TD profile, sulphur dioxide was a significant component, although in the absence of other sulphur compounds any interpretation of its significance has to be very tentative, beyond an organic sulphur source. Cyclopentadiene and methylcyclopentadiene are also significant components of the pyrolysate although it is not possible to ascribe a specific origin here. Acetone was also identified and can be indicative of the amino acid glycine or a carbohydrate component, although any interpretation should be considered tentative here.

In contrast to the thermal desorption profile, the pyrogram also revealed a complex suite of organic compounds, albeit in relatively minor abundance. These include a series of C_7 to C_{18} *n*-1-alkenes and *n*-alkanes, typical of unsaturated and saturated fat/oil-derived acyl lipids[5,6]. The absence of these compounds in the TD profile provides molecular evidence for a polymeric/bound acyl lipid source. However, in addition to *n*-1-alkenes and *n*-alkanes, typical of fat/oil derived lipids, the pyrogram also displayed a several branched alkenes and alkanes (C_9, C_{13}, C_{15} and C_{18}), presumably deriving from the branched fatty acids which are

significant components of bacterial triglycerides. Although non-specific, the presence of carbon dioxide is also consistent with a (highly oxidised) lipid component in this sample.

The pyrolysate TIC identified components indicative of 'black carbon', i.e. charcoal or soot[7-10]. Benzene and toluene were moderately abundant compounds present in the pyrolysate (~30% and 45% of total quantified pyrogram peak area respectively), along with significant quantities of ethyl benzene and o-, m- and p-xylenes (~10% total). The relative dominance of toluene, rather than the normally dominant aromatic hydrocarbon benzene, is somewhat atypical of chars[7-10]. However, the presence of significant amounts of naphthalene, methyl naphthalenes and dimethyl naphthalenes, together with the polynuclear aromatic hydrocarbon combustion marker fluorene, confirm a 'char' component providing molecular evidence for exposure to fire/cooking. The lack of the attendant aromatic nitrogen-containing biomarkers pyrrole, benzonitrile, benzyl nitrile (phenylacetonitrile), benzenepropanenitrile and indoles indicative of proteinaceous material[6-11] suggests a collective origin as combustion compounds ('char') rather than a significant protein component, although pyridine, styrene, indene and C_1 and C_2 alkyl indenes are indicative of a protein constituent and so diagenetic processes may explain the lack of nitrogenous organic molecules. The presence of carbon dioxide is also consistent with a lipid component in this sample. It should also be noted that highly polar material could have been present, which would not have successfully eluted from the column, or indeed volatilised sufficiently in the probe.

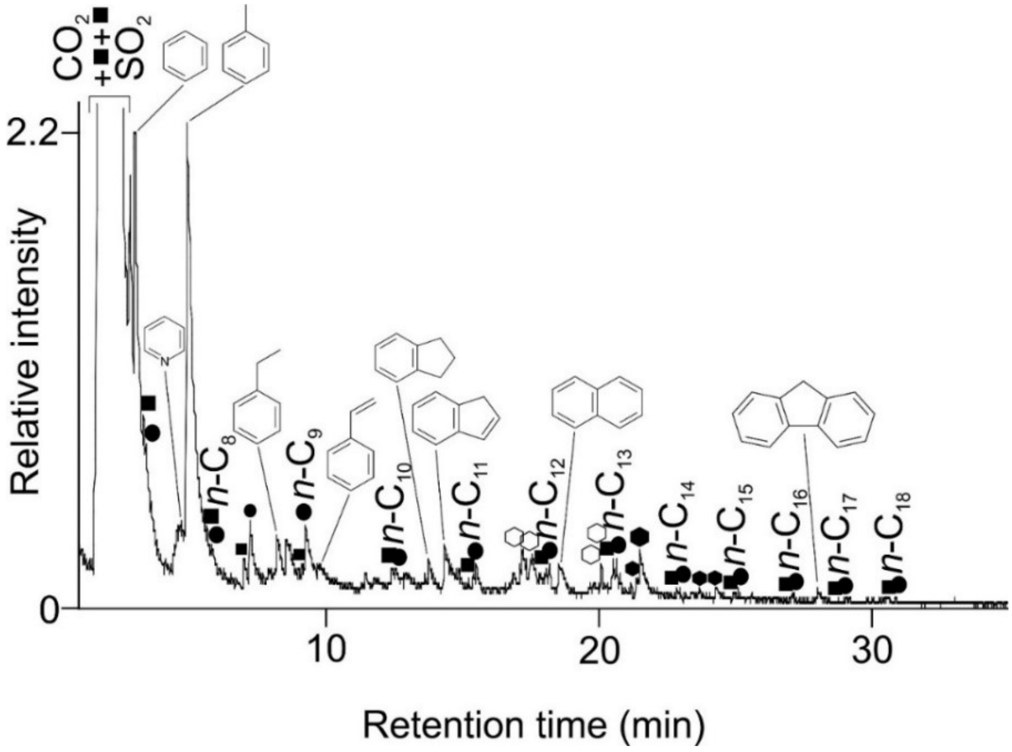

Supplementary Figure 48. Reconstructed total ion chromatogram of the pyrogram (pyrolysis profile) (610 °C for 10s), after thermal desorption (310 °C for 10 s) of sample Elvis F1004 from Brui 0011014 p.22 VI.35 Hardinxveld de Bruin. Key to Fig. 48: Peak identities (x indicates carbon chain length): filled squares, Cx indicates alkenes; filled circles, Cx indicates alkanes. Also shown are the structures of nine aromatic compounds identified: benzene, pyridine, toluene, ethylbenzene, styrene, indane, indene, naphthalene and fluorene. Open hexagons indicates alkyl indenes; filled hexagons indicates alkyl naphthalenes. CO_2 indicates carbon dioxide and SO_2 indicates sulphur dioxide.

Hardinxveld de Bruin soil 'control' from Brui 0011014 p.22 VI.35 (soil/sediment: 10.48 mg)

The thermal desorption total ion chromatogram (TIC) revealed carbon dioxide as the only detectable (trace) component, indicating the essential absence of free, thermally extractable organic components in this sample. It should be noted, however, that highly polar material could have been present, which would not have successfully eluted from the column, or indeed volatilised sufficiently in the probe. It is not possible to determine the significance of the carbon dioxide, beyond it being indicative of oxidised organic material.

The pyrolysate TIC revealed carbon dioxide and sulphur dioxide (trace) as the only detectable components, indicating very little organic material in this sample. It should be noted, however, that highly polar material could have been present, which would not have successfully eluted from the column, or indeed volatilised sufficiently in the probe. It is not possible to determine the significance of the carbon dioxide, beyond it being indicative of oxidised organic material.

77. Hardinxveld de Bruin (Meso), Box 0308 mandible lingual right PM1 (Henk) (calculus: 0.23 mg)

The thermal desorption total ion chromatogram (TIC) revealed no detectable components, indicating the absence of free, thermally extractable organic components in this sample. It should be noted, however, that highly polar material could have been present, which would not have successfully eluted from the column, or indeed volatilised sufficiently in the probe.

The pyrolysate TIC revealed carbon dioxide as the only detectable (minor trace) component, indicating the essential absence of organic material in this sample. It should be noted, however, that highly polar material could have been present, which would not have successfully eluted from the column, or indeed volatilised sufficiently in the probe. It is not possible to determine the significance of the carbon dioxide, beyond it being indicative of oxidised organic material, although its presence as only a very trace component makes even this inference of no meaningful value.

Hardinxveld de Bruin (Meso) Box 0308 soil 'control' from burial (soil/sediment: 11.47 mg)

The thermal desorption total ion chromatogram (TIC) revealed carbon dioxide and sulphur dioxide as the only detectable components. No other free, thermally extractable, organic components could be detected in this sample. It should be noted, however, that highly polar material could have been present, which would not have successfully eluted from the column, or indeed volatilised sufficiently in the probe. It is not possible to determine the significance of the carbon dioxide, beyond it being indicative of oxidised organic material. Sulphur dioxide is also known to be a major pyrolysis product of lignosulphonates[137], and its organic

nature would facilitate sulphur dioxide generation at this relatively low temperature, although this possibility in this context is probably less likely (again, see discussion/summary).

The pyrolysis-GC-MS TIC was dominated by carbon dioxide, with sulphur dioxide and carbon disulphide the only other major components. Collectively these latter two compounds can be indicative of sulphur-containing amino acids or sulphones[135,136] (see discussion), which suggests they derive from the burial soil/sediment (see discussion). It is not possible to determine the significance of the carbon dioxide, beyond it being indicative of oxidised organic material.

78. Hardinxveld Trintjuite, Box 0279 F1004 Put 25 mandible incisor lingual (female) (calculus: 0.15 mg)

The thermal desorption total ion chromatogram (TIC) revealed carbon dioxide as the only detectable (trace) component, indicating the essential absence of free, thermally extractable organic components in this sample. It should be noted, however, that highly polar material could have been present, which would not have successfully eluted from the column, or indeed volatilised sufficiently in the probe. It is not possible to determine the significance of the carbon dioxide, beyond it being indicative of oxidised organic material.

The pyrolysate TIC revealed carbon dioxide as the only detectable (minor trace) component, indicating the essential absence of organic material in this sample. It should be noted, however, that highly polar material could have been present, which would not have successfully eluted from the column, or indeed volatilised sufficiently in the probe. It is not possible to determine the significance of the carbon dioxide, beyond it being indicative of oxidised organic material, although its presence as only a very trace component makes even this inference of no meaningful value.

Discussion, Hardinxveld de Bruin burials

Three samples of dental calculus and two burial ‘control’ samples of soil from Hardinxveld were analysed by sequential thermal desorption-gas chromatography-mass spectrometry (TD-GC-MS) and pyrolysis-gas chromatography-mass spectrometry (Py-GC-MS). The samples contained both free and bound/polymeric organic material, albeit highly oxidised.

Beyond the relatively major abundance of carbon dioxide, with minor amounts of carbon disulphide and sulphur dioxide, the calculus and soil samples contained very little free and bound/polymeric organic compounds, most likely reflecting particularly unfavourable prevailing conditions for biomolecular preservation. The dominance of only a very few small organic compounds, primarily carbon dioxide, suggests that only highly oxidised sulphur-containing organic material was present.

Notably, the Netherlands has one of the highest sulphur contents in subsoils[140,141], which includes the site of Hardinxveld. The site is also in close proximity to marshland and peat[145], which are also known to be high in organic and inorganic sulphur[143,144]. At this site the alkyl thiophenes and elemental sulphur (as S_8) were not observed and so no meaningful interpretation of the significance of the carbon disulphide and sulphur dioxide can be given beyond being indicative of an organic, and possibly inorganic, sulphur source, although their presence here may be due to the burial matrix rather than having any direct connection with the dental calculus itself. It's also possible that the sulphur dioxide may, at least in part, derive from the thermal decomposition of sulphuric acid in the probe, which can be expected to take place at the thermal desorption and pyrolysis temperatures employed.

The bound/polymeric acyl lipid component observed in the Hardinxveld de Bruin Brui 0011014 calculus sample does not appear in the soil samples from the same burial site and therefore this indicates a bacterial lipid constituent present in the calculus, which may well derive from the individual's own oral microbiome. Although biomolecular preservation was poor, there was organic chemical evidence for exposure to fire/cooking, and within this 'char' the indication of an appreciable protein component, which correlates with the abundance of the protein marker 1-butene in this calculus sample. Beyond this, there was nothing which could be indicative of dietary input or environmental exposure. The Mesolithic samples (calculus and soil) and Hardinxveld Trintjuite sample revealed a virtual absence of evidence for an organic constituent, suggesting the age and particular archaeological context at these sites were extremely unfavourable for biomolecular preservation.

Overall, it is worth noting that the limited biomolecular information obtained from the Schuipluden and Hardinxveld samples here may well reflect poor environmental conditions for the chemical preservation of an organic component in dental calculus, and indeed other organic residues.

Kruszyn, Poland

Introduction

One sample of dental calculus from an individual from the Linear Band Pottery culture was taken from the site of Kruszyn. Kryszyn is situated on the left bank of Vistula river (Włocławek district, central Poland). Rescue excavations conducted in 2004, 2007-2009 revealed multicultural settlement and graves from Neolithic, Early Bronze Age, Roman Period, Medieval Age and Modern Times. Grave goods of LBK included fragments of pottery[146]. The sample was analysed by sequential thermal desorption-gas chromatography-mass spectrometry (TD-GC-MS) and pyrolysis-gas chromatography-mass spectrometry (Py-GC-MS). This technique facilitates the identification of both free/unbound and bound/polymeric organic components.

79. Kruszyn, Poland: Grave G93, Site 13, male 35-40/45 years old Linear Band Pottery culture (calculus: 9.73 mg)

The thermal desorption total ion chromatogram (TIC) revealed carbon dioxide as the only detectable (trace) component, indicating the essential absence of free, thermally extractable organic components in this sample. It should be noted, however, that highly polar material could have been present, which would not have successfully eluted from the column, or indeed volatilised sufficiently in the probe. It is not possible to determine the significance of the carbon dioxide, beyond it being indicative of oxidised organic material.

The pyrolysate TIC revealed carbon dioxide and the C_4 and C_5 1-alkenes as the only detectable components. It is not possible to determine the significance of the carbon dioxide, beyond it being indicative of oxidised organic material and the two short chain hydrocarbons are too low an abundance and too ubiquitous to provide any meaningful interpretation of their presence, beyond being indicative of an organic origin. It should be noted, however, that highly polar material could have been present, which would not have successfully eluted from the column, or indeed volatilised sufficiently in the probe.

Discussion, Kruszyn

The sample contained very little free and bound/polymeric organic material, most likely reflecting unfavourable prevailing conditions for biomolecular preservation. Of the organic

compounds identified, the dominance of carbon dioxide and the relatively minor abundance of only two small organic compounds suggests that only highly oxidised organic material was present.

Rønstenen, Denmark

Rønstenen is a Middle Mesolithic settlement currently submerged to around two meters in marine water. It is well known among local divers, as a site rich in lithic items, while, occasionally, organic materials have also been recovered. It has been regularly inspecred by the Danish Authority for Cultural Heritage who salvaged material of scientific importance. Among these finds was a right mandibula with three extant teeth that dates to the same period as the lithics: 7542±42 ^{14}C years BP (AAR-11355)[147].

One sample of dental calculus taken from one individual from the site of Rønstenen was analysed by sequential thermal desorption-gas chromatography-mass spectrometry (TD-GC-MS) and pyrolysis-gas chromatography-mass spectrometry (Py-GC-MS). This technique facilitates the identification of both free/unbound and bound/polymeric organic components.

80. Rønstenen, Denmark: AS 15/06 (calculus: 0.60 mg)

The thermal desorption total ion chromatogram (TIC) revealed carbon dioxide as the only detectable (trace) component, indicating the essential absence of free, thermally extractable organic components in this sample. It should be noted, however, that highly polar material could have been present, which would not have successfully eluted from the column, or indeed volatilised sufficiently in the probe. It is not possible to determine the significance of the carbon dioxide, beyond it being indicative of oxidised organic material.

The pyrolysate TIC contained very little detectable organic material. The only components identified were carbon dioxide, sulphur dioxide, C_4 to C_6 1-alkenes, cyclopentadiene and a trace amount of the aromatic hydrocarbon benzene. The dominance of carbon dioxide, combined with the relatively minor abundance of the few small organic compounds present, suggests a highly oxidised sample. It is not possible to determine the significance of the carbon dioxide, beyond it being indicative of oxidised organic material and the other compounds are too low an abundance and too ubiquitous to provide any meaningful interpretation of their presence, beyond being indicative of an organic origin. It should be

noted, however, that highly polar material could have been present, which would not have successfully eluted from the column, or indeed volatilised sufficiently in the probe.

Discussion, Rønstenen

The sample contained very little free and bound/polymeric organic material, most likely reflecting unfavourable prevailing conditions for biomolecular preservation. Of those organic compounds identified, the dominance of carbon dioxide and the relatively minor abundance of only a few small organic compounds suggests that only highly oxidised organic material was present.

Supplementary References:

1. Olalde, I., Brace, S., Allentoft, M.E., Armit, I., Kristiansen, K., Booth, T., Rohland, N., Mallick, S., Szécsényi-Nagy, A., Mittnik, A. and Altena, E., 2018. The Beaker phenomenon and the genomic transformation of northwest Europe. *Nature*, *555*(7695), pp.190–196.

2. Lawrence DM (2012) *Orkney's First Farmers: Reconstructing biographies from osteological analysis to gain insights into life and society in a Neolithic community on the edge of Atlantic Europe*. PhD Thesis (University of Bradford, Bradford, UK).

3. Sheridan, J.A. & Schulting, R. 2021 Making sense of Scottish Neolithic funerary monuments: tracing trajectories and understanding their rationale. In A.B. Gebauer, L. Soerensen [replace the 'oe' by the Danish character for this], A. Teather and A.C. Valera (eds), *Monumentalising Life in the Neolithic: narratives of change and continuity*, 195–215. Oxford: Oxbow

4. Card, N., Mainland, I., Timpany, S., Towers, R., Batt, C., Ramsey, C.B., Dunbar, E., Reimer, P., Bayliss, A., Marshall, P. and Whittle, A., 2018. To cut a long story short: formal chronological modelling for the Late Neolithic site of Ness of Brodgar, Orkney. *European Journal of Archaeology*, *21*(2), pp.217–263.

5. Buckley SA, Stott AW, Evershed RP (1999) Studies of organic residues from ancient Egyptian mummies using high temperature-gas chromatography-mass spectrometry and

sequential thermal desorption-gas chromatography-mass spectrometry and pyrolysis-gas chromatography-mass spectrometry. *Analyst* 124:443–452.

6. Hardy K, Buckley S, Collins MJ, Estalrrich A, Brothwell DR, et al. (2012) Neanderthal medics? Evidence for food, cooking and medicinal plants entrapped in dental calculus. *Naturwissenschaften* 99: 617–626. doi: 10.1007/s00114-012-0942-0

7. Kaal J, Martínez Cortizas A, Eckmeier E, Costa Casais M, Santos Estévez M, et al. (2008) Holocene fire history of black colluvial soils revealed by pyrolysis-GC/MS: a case study from Campo Lameiro (NW Spain). *Journal of Archaeological Science* 35: 2133–2143. doi: 10.1016/j.jas.2008.01.013

8. Kaal J, Brodowski S, Baldock JA, Nierop KGJ, Martínez Cortizas A (2008) Characterisation of aged black carbon pyrolysis-GC/MS, thermally assisted hydrolysis and methylation (THM), direct and cross polarisation 13C nuclear magnetic resonance (DP/CP NMR) and the benzenepolycarboxylic acid (BPCA) method. *Organic Geochemistry* 39: 1415–1426. doi: 10.1016/j.orggeochem.2008.06.011

9. Kaal J, Martínez-Cortizas A, Buurman P, Criado Boado F (2008) 8000 yr of black carbon accumulation in a colluvial soil from NW Spain. *Quaternary Research* 69: 56–61. doi:10.1016/j.yqres.2007.10.005

10. Kaal J, Martínez Cortizas A, Nierop KGJ (2009) Characterisation of aged charcoal using a coil probe pyrolysis-GC/MS method optimised for black carbon. *Journal of Analytical and Applied Pyrolysis* 85: 408-416. doi: 10.1016/j.jaap.2008.11.007

11. Simmonds PG (1970) Whole microorganisms studied by pyrolysis-gas chromatography-mass spectrometry: significance for extraterrestrial life detection experiments. *Applied Microbiology* 20(4): 567-572.

12. Stankiewicz BA, van Bergen PF, Duncan IJ, Carter JF, Briggs DEG, Evershed RP (1996) Recognition of Chitin and Proteins in Invertebrate Cuticles Using Analytical Pyrolysis/Gas Chromatorgraphy/Mass Spectrometry. *Rapid Communications in Mass Spectrometry* 10: 1747–1757.

13. Stankiewicz BA, Hutchins JC, Thomson R, Briggs DEG, Evershed RP (1997) Assessment of Bog Body Tissue Preservation by Pyrolysis-Gas Chromatorgraphy/Mass Spectrometry. *Rapid Communications in Mass Spectrometry* 11: 1884–1890.

14. Stankiewicz BA, Briggs DEG, Evershed RP, Flannery MB, Wuttke M (1997) Preservation of Chitin in 25-Million-Year-Old Fossils. *Science* 276: 1541–1543.

15. van der Hage ERE, Mulder MM, Boon JJ (1993) Structural characterization of lignin polymers by temperature-resolved in-source pyrolysis-mass spectrometry and Curie-point pyrolysis-gas chromatorgraphy/mass spectrometry. *Journal of Analytical and Applied Pyrolysis* 25: 149–183.

16. Galletti GC, Bocchini P (1995) Pyrolysis/Gas Chromatography/Mass Spectrometry of Lignocellulose. *Rapid Communications in Mass Spectrometry* 9: 815–826.

17. Ré-Poppi N, Santiago-Silva MR (2002) Identification of polycyclic aromatic hydrocarbons and methoxylated phenols in wood smoke emitted during production of charcoal. *Chromatographia* 55:475–481.

18. Varlet V, Knockaert C, Prost C, Serot T (2006) Comparison of odoractive volatile compounds of fresh and smoked salmon. *Journal Agricultural and Food Chemistry* 54:3391–3401.

19. Chiavari G, Galletti GC (1992) Pyrolysis-gas chromatography/mass spectrometry of amino acids. *Journal of Analytical and Applied Pyrolysis* 24:123–137

20. Fuentes M, Baigorri R, González-Vila FJ, González-Gaitano G, García-Mina JM (2010) Pyrolysis-Gas Chromatography/Mass Spectrometry Identification of Distinctive Structures Providing Humic Character to Organic Materials. *Journal of Environmental Quality* 39: 1486–1497.

21. SinningheDamsté JS, Eglinton TI, de Leeuw JW (1991) Alkylpyrroles in kerogen pyrolysates : evidence for abundant macromolecularly-bound tetrapyrrole pigments. *Preprints American Chemical Society - Division Fuel Chemistry* 36: 710–713.

22. SinningheDamsté JS, Eglinton TI, de Leeuw JW (1992) Alkylpyrroles in a kerogen pyrolysate: Evidence for abundant tetrapyrrole pigments. *Geochimica et Cosmochimica Acta* 56: 1743–1751.

23. Schulten H-R, Schnitzer M (1998) The chemistry of soil organic nitrogen: a review. *Biology and Fertility of Soils* 26: 1–15.

24. Morgan PJ, Smith K (1978) Potentiality of Seaweed as a Resource: Analysis of the Pyrolysis Products of *Fucus serratus*. *Analyst* 103: 1053–1060.

25. McCobb LME, Briggs DEG, Evershed RP, Hall AR, Hall RA (2001) Preservation of Fossil Seeds From a 10th Century AD Cess Pit at Coppergate, York. *Journal of Archaeological Science* 28: 929–940.

26. Bracewell JM, Robertson GW (1984) Quantitative comparison of the nitrogen-containing pyrolysis products and amino acid composition of soil humic acids. *Journal of Analytical and Applied Pyrolysis* 6: 19–29.

27. Kolattukudy PE (1965) Biosynthesis of Wax in Brassica oleracea. *Biochemistry* 4: 1844–1855.

28. Hill AS, Mattick LR (1966) The *n*-alkanes of cabbage (var. copenhagen) and sauerkraut. *Phytochemistry* 5, pp.693–697.

29. Charters S, Evershed RP, Quye A, Blinkhorn PW, Reeves V (1997) Simulation Experiments for Determining the Use of Ancient Pottery Vessels: the Behaviour of Epicuticular Leaf Wax During Boiling of a Leafy Vegetable. *Journal of Archaeological Science* 24: 1–7.

30. Vioque J, Pastor J, Vioque E (1994) Leaf wax alkanes in the genus *Coincya*. *Phytochemistry* 36: 349–353.

31. Evershed RP, Heron C, Goad LJ (1991) Epicuticular wax components preserved in potsherds as chemical indicators of leafy vegetables in ancient diets. *Antiquity* 65: 540–544.

32. Crossley J (2013) *A Checklist of the Flora of Orkney*.

33. Masefield GB, Wallis M, Harrison SG, Nicholson BE (1969) *The Oxford Book of Food Plants.* (Oxford University Press, London).

34. Mabey R (1972) *Food for Free: A guide to the edible wild plants of Britain.* (Collins, London).

35. Soothill E, Thomas MJ (1983) *Nature's Wild Harvest.* (New Orchard Editions Ltd, Poole).

36. Shepherd T, Wynne Griffiths D (2006) The effects of stress on plant cuticular waxes. *New Phytologist* 171: 469–499.

37. Online Atlas of the British and Irish Flora: https://www.brc.ac.uk/plantatlas/plant/crambe-maritima

38. Travillian, T.T. ed., 2015. *Pliny the Elder: The Natural History Book VII (with Book VIII 1–34)*. Bloomsbury Publishing.

39. *Mrs Beeton's Book of Household Management.* (1861) Beeton Publishing.

40. Stankiewicz BA, van Bergen PF (eds.) (1998) *Nitrogen-containing Macromolecules in the Bio- and Geosphere*. (American Chemical Society, Washington).

41. Patience RL, Baxby M, Bartle KD, Perry DL, Rees AGW, Rowland SJ (1992) The functionality of organic nitrogen in some recent sediments from the Peru upwelling region. *Organic Geochemistry* 18: 161–169.

42. Parker, B.C. & Dawson, E.Y. Non-calcareous marine algae from California Miocene deposits. *Nova Hedwigia* **10**, 273-295 Plates 76-96 (1965).

43. Behl, R.J. Since Bramlette(1946): The Miocene Monterey Formation of California revisited. In: Moores, E.M., Sloan, D. & Stout, D.L.(Eds.), Classic Cordilleran Concepts: A View from California. *Geological Society of America Special Paper* **338**, Boulder. 301-313 (1999).

44. Cao, J., Bian, L.Z., Hu, K., Liu, Y.T., Yang, S.Y.,Wang, L.Q., Chen, Y. & Peng, X.Q. Benthic macro red alga: a new possible bio-precursor of Jurassic mudstone source rocks in the northern Qaidam Basin, northwestern China. *Sci. China. Ser. D Earth Sci*. **52**, 647–654 (2009).

45. Xie, X, Volkman, J.K., Qin, J, Borjigin, T, Bian, L. & Zhen L. Petrology and hydrocarbon potential of microalgal and macroalgal dominated oil shales from the Eocene Huadian Formation, NE China. *Int. J. Coal Geol.* **124**, 36-47 (2014).

46. Zhang, S.C., Zhang, B.M., Bian, L.Z., Jin, Z.J., Wang, D.R. & Chen, J.F. The Xiamaling oil shale generated through Rhodophyta over 800 Ma ago. *Sci. China, Ser. D Earth Sci.* **50**, 527–535 (2007).

47. Bryant DA, Hunter CN, Warren MJ (2020) Biosynthesis of the modified tetrapyrroles – the pigments of life. *Journal of Biological Chemistry* 295: 6888–6925.

48. Miranda LN, Hutchison K, Grossman AR, Brawley SH (2013) Diversity and Abundance of the Bacterial Community of the Red Macroalga *Porphyra umbilicalis*: Did Bacterial Farmers Produce Macroalgae? *PLOS ONE* 8: e58269.

49. Longford SR, Tujula NA, Crocetti GR, Holmes AJ, Holmström C, Kjelleberg S, Steinberg PD, Taylor MW (2007) Comparisons of diversity of bacterial communities associated with three sessile marine eukaryotes. *Aquatic Microbial Ecology* 48: 217–229.

50. Morrissey J, Kraan S, Guiry MD (2001) *A Guide to Commercially Important Seaweeds on the Irish Coast.* (Irish Sea Fisheries Board, DunLaoghaire, Co. Dublin).

51. Taboada C, Millan R, Miguez I (2013) Evaluation of marine *Undaria pinnatifida* and *Porphyrapurpurea* as a food supplement: composition, nutritional value and effect of intake on intestinal, hepatic and renal enzyme activities in rats. *Journal of the Science of Food and Agriculture* 93: 1863–1868.

52. Dawczynski C, Schubert R, Jahreis G (2007) Amino acids, fatty acids, and dietary fibre in edible seaweed products. *Food Chemistry* 103: 891–899.

53. Vinoj Kumar V, Kaladharan P (2007) Amino acids in the seaweeds as an alternative source of protein for animal feed. *Journal of Marine Biology Association of India* 49: 35–40.

54. Galland-Irmouli A-V, Fleurence J, Lamghari R, Luçon M, Rouxel C, Barbaroux O, Bronowicki J-P, Villaume C, Guéant J-L (1999) Nutritional value of proteins from edible seaweed *Palmariapalmata* (Dulse). *Journal of Nutritional Biochemistry* 10: 353–359.

55. Takruri I, Haslett BG, Boulter D, Andrew PW, Rogers LJ (1978) The Amino Acid Sequence of Ferredoxin from the Red Alga *Porphyraumbilicalis*. *Biochemical Journal* 173: 459–466.

56. Brothwell D, Brothwell P (1969) *Food in Antiquity* (Thames and Hudson, London), p.120.

57. Schulting, R J and Richards, M P (2002) ‘The wet, the wild and the domesticated: the Mesolithic-Neolithic transition on the west coast of Scotland’. *European Journal of Archaeology* 5: 147–89.

58. Poinar HN, Stankiewicz BA (1999) Protein preservation and DNA retrieval from ancient tissues. Proceedings of the National Academy of Sciences 96: 8426–8431.

59. Jones AB, O'Donohue MJ, Udy J, Dennison WC (2001) Assessing Ecological Impacts of Shrimp and Sewage Effluent: Biological Indicators with Standard Water Quality Analyses. *Estuarine, Coastal and Shelf Science* 52: 91–109.

60. Lawlor DW, Boyle FA, Young AT, Kendall AC, Keys AJ (1987) Nitrate Nutrition and Temperature Effects on Wheat: Soluble Components of Leaves and Carbon Fluxes to Amino Acids and Sucrose. *Journal of Experimental Botany* 38: 1091–1103.

61. Kiladze TD, Tsanava VP, Izmailov SF (1989) Biosynthesis and distribution of nitrogen-containing compounds in tea plants fed through roots on nitrogen-15 nitrate and carbon-14 sucrose. *FiziologiyaRastenii (Moscow)* 36: 1155–1163.

62. Silveira JSM, Sant'anna R, Rena AB, Garcia R (1985) Nitrogen translocation as a function of the proportion of nitrate and ammonia. *Pesquisa Agropecuaria Brasileira* 20: 15–24.

63. Yaylayan VA, Keyhani A (2001) Elucidation of the mechanism of pyrrole formation during thermal degradation of ^{13}C-labeled L-serines. *Food Chemistry* 74: 1–9.

64. Hilditch TP, Williams PN (1964) *The Chemical Constitution of Natural Fats* (4thEdn.) (Chapman and Hall, London) pp.27–29.

65. Hayashi K, Kida S, Kato K, Yamada M (1974) Component Fatty Acids of Acetone-Soluble Lipids of 17 Species of Marine Benthic Algae. *Bulletin of the Japanese Society of Scientific Fish*eries 40: 609–617.

66. Jamieson GR, Reid EH (1972) The component fatty acids of some marine algal lipids. *Phytochemistry* 11: 1423–1432.

67. Johns RB, Nichols PD, Perry GJ (1979) Fatty acid composition of ten marine algae from Australian waters. *Phytochemistry* 18: 799–802.

68. van Ginneken VJT, Helsper JPFG, de Visser W, van Keulen H, Brandenburg WA (2011) Polyunsaturated fatty acids in various macroalgal species from north Atlantic and tropical seas. *Lipids in Health and Disease* 10: 104.

69. Fleurence J, Gutbier G, Mabeau S, Leray C (1994) Fatty acids from 11 marine macroalgae of the Brittany coast. *Journal of Applied Phycology* 6: 527–532.

70. Wang S, Wang Q, Jiang X, Han X, Ji H (2013) Compositional analysis of bio-oil derived from pyrolysis of seaweed. *Energy Conversion and Management* 68: 273–280.

71.Versteegh, G.J.M., Blokker, P., Wood, G.D., Collinson, M.E., Sinninghe Damsté, J.S. & de Leeuw, J.W. An example of oxidative polymerisation of unsaturated fatty acids as a preservation pathway for dinoflagellate organic matter. *Org. Geochem.* **35**, 1129-1139 (2004).

72. Gupta, N.S., et al. Evidence for the *in situ* polymerisation of labile aliphatic organic compounds during the preservation of fossil leaves: Implications for organic matter preservation. *Org. Geochem.* **38**, 499-522 (2007).

73. Strobel C, Jahreis G, Kuhnt K (2012) Survey of *n*-3 and *n*-6 polyunsaturated fatty acids in fish and fish products. *Lipids in Health and Disease* 11: 144.

74. Bell JG, Tocher DR, Henderson RJ, Dick JR, Crampton VO (2003) Altered Fatty Acid Composition in Atlantic Salmon (*Salmo salar*) Fed Diets Containing Linseed and Rapeseed Oils Can Be Partially Restored by a Subsequent Fish Oil Finishing Diet. *Journal of Nutrition* 133: 2793–2801.

75. Hamilton MC, Hites RA, Schwager SJ, Foran JA, Knuth BA, Carpenter DO (2005) Lipid Composition and Contaminants in Farmed and Wild Salmon. *Environmental Science and Technology* 39: 8622–8629.

76. Huynh MD, Kitts DD, Hu C, Trites AW (2007) Comparison of fatty acid profiles of spawning and non-spawning Pacific herring, *Clupea harenguspallasi*. *Comparative Biochemistry and Physiology, Part B* 146: 504–511.

77. Aminullah Bhuiyan AKM, Ratnayake WMN, Ackman RG (1986) Stability of Lipids and Polyunsaturated Fatty Acids During Smoking of Atlantic Mackerel (*Scomber scombrus* L.). Journal of the American Oil Chemists Society 63: 324–328.

78. Maberly SC, Raven JA, Johnston AM (1992) Discrimination between ^{12}C and ^{13}C by marine plants. *Oecologia* 91: 481–492.

79. Fontanals-Coll M, Subirà ME, Marín-Moratalla N, Ruiz J (2014) From Sado Valley to Europe: Mesolithic dietary practices through different geographic distributions. *Journal of Archaeological Science* 50: 539–550.

80. Renfrew, C (ed) 1979. *Investigations in Orkney*. London: Thames & Hudson.

81. Schulting R, Sheridan JA, Crozier R, Murphy E (2010) Revisiting Quanterness: New AMS Dates and Stable Isotope Data from an Orcadian Chamber Tomb. Proceedings of the Society of Antiquaries of Scotland, 140: 1–50.

82. Anderson, J., 1895, November. Notice of a cave recently discovered at Oban, containing human remains, and a refuse-heap of shells and bones of animals, and stone and bone implements. In *Proceedings of the Society of Antiquaries of Scotland,* 29:211–230.

83. Armit, I, Sheridan, JA, Reich, D, Cook, G, Tripney, B and Naysmith, P 2016 Radiocarbon dates obtained for the GENSCOT ancient DNA project. *Discovery and Excavation in Scotland* 17, 195–8.

84. Connock, K. 1985 *Rescue Excavation of the Ossuary Remains at Raschoille Cave, Oban: an interim report*. Oban: Lorn Archaeological and Historical Society Special Publication

85. Bonsall, C, Pickard, C and Ritchie, GA 2012 From Assynt to Oban: some observations on prehistoric cave use in western Scotland. In KA Bergsvik and R Skeates (eds), *Caves in Context: the cultural significance of caves and rockshelters in Europe*, 10–21. Oxford: Oxbow.

86. Sheridan, JA., Armit, I., Reich, D., Booth, T., Bernardos, R., Barnes, I., Thomas, M., Charlton, S., Craig, O., Lawson, J. and Dulias, K., 2018. A summary round-up list of scottish archaeological human remains that have been sampled/analysed for DNA as of January 2019. *Discovery and Excavation in Scotland* Vol 19.

87. Pearson, MP, Sheridan, JA, Jay, M, Chamberlain, A, Richards, M and Evans, J., 2019. *The Beaker People: isotopes, mobility and diet in prehistoric Britain*. Oxbow Books.

88. Clarke, DV, Ritchie, A and Ritchie, JNG 1984 Two cists from Boatbridge Quarry, Thankerton, Lanarkshire. *Proceedings of the Society of Antiquaries of Scotland* 114, 557–88

89. Ficken KJ, Li B, Swain DL, Eglinton G (2000) An *n*-alkane proxy for the sedimentary input of submerged/floating freshwater aquatic macrophytes. *Organic Geochemistry* 31: 745–749.

90. Gao L, Hou J, Toney J, MacDonald D, Huang Y (2011) Mathematical modeling of the aquatic macrophyte inputs of mid-chain *n*-alkyl lipids to lake sediments: Implications for interpreting compound specific hydrogen isotopic records. *Geochimica et Cosmochimica Acta* 75: 3781–3791.

91. Tuo J, Wu C, Zhang M, Chen R (2011) Distribution and carbon isotope composition of lipid biomarkers in Lake Erhai and Lake Gahai sediments on the Tibetan Plateau. *Journal of Great Lakes Research* 37: 477–455.

92. Street JH, Anderson RS, Rosenbauer RJ, Paytan A (2013) *n*-Alkane evidence for the onset of wetter conditions in the Sierra Nevada, California (USA) at the mid-late Holocene transition, ~3.0 ka. *Quaternary Research* 79: 14–23.

93. Fischer A, Richards M, Olsen J, Robinson DE, Bennike P, Kubiak-Martens L, Heinemeier J 2007. The Composition of Mesolithic Food: Evidence from the Submerged Settlement on the Argus Bank, Denmark. *Acta Archaeologica* 78(2): 163–178.

94. Hardy K .2007. Food for thought: starch in Mesolithic diet. *Mesolithic Miscellany* 18(2): 2–11.

95. Ashmore, PJ, Brooks, M, Maté, I and Strong, P 1982 A cist at Ruchlaw Mains, East Lothian (NT 616742). *Proceedings of the Society of Antiquaries of Scotland* 112, 542–8

96. Cruden, S. 1958. Skateraw, East Lothian. *Discovery and Excavation in Scotland* 1958, 39

97. Sheridan, JA 2004 The National Museums of Scotland radiocarbon dating programmes: results obtained during 2003/4. *Discovery and Excavation in Scotland* 5, 174–6

98. Girininkas A. Kretuonas. Vidurinis ir vėlyvasis neolitas. Lietuvos archeologija, T. 7, Vilnius, 1990.

99. Butrimas A. Donkalnio ir Spigino mezolito-neolito kapinynai. Seniausi laidojimo paminklai Lietuvoje. Vilniaus dailės akademijos leidykla, 2012.

100. Tebelškis P. Gyvakarų kapinynas, Archeologiniai tyrinėjimai Lietuvoje 2000 m., Vilnius, 2001, p. 24-25.

101. Viso A-C, Pesando D, Bernard P, Marty J-C (1993) Lipid components of the Mediterranean Seagrass *Posidonia oceanica*. *Phytochemistry* 34(2): 381-387.

102. Urbanavičius V. Obelių plokštinio kapinyno tyrinėjimai. Archeologiniai tyrinėjimai Lietuvoje 1982 ir 1983 m., Vilnius, 1984 P. 95-98

103. Han J, Calvin M (1969) Hydrocarbon Distribution of Algae and Bacteria, and Microbial Activity in Sediments. Proceedings of the National Academy of Sciences 64(2): 436-443.

104. Aaronson S (1986) A role for algae as human food in antiquity. Food and Foodways 1(3): 311-315.

105. Vaitkunskienė L. The Pagrybis burial ground. In Pagrybio kapinynas. Lietuvos archeologija, t.13, p. 174-181.

106. Juškaitis V. Vėluikių kapinynas. Archeologiniai tyrinėjimai Lietuvoje 2006 m., Vilnius, 2007, p. 168-170.

107. Daubaras M. Pašatrijos piliakalnis ir papėdės gyvenvietė. Archeologiniai tyrinėjimai Lietuvoje 2012 m., Vilnius, 2013, p. 77-86.108. Hallgren, F., Gummesson, S., Berggren, K. & Storå, J. 2021. Human – animal symbolism within a ritual space in the Mesolithic wetland deposit at Kanaljorden, Motala. In: Borić, D. D., Antonović, A. & B. Mihailović (eds.), *Foraging Assemblages, Volume 2.* Serbian Archaeological Society/The Italian Academy for Advanced Studies in America, Columbia University, Belgrade & New York. p.644–648.

109. Hallgren, F. & Fornander, E. 2016. Skulls on stakes and skulls in water. Mesolithic mortuary rituals at Kanaljorden, Motala, Sweden 7000 BP. In: Gramsch, B., Meller, H., Orschiedt, J., Larsson, L. & Grünberg, J. M. (red.) *Mesolithic burials: rites, symbols and social organisation of early postglacial communities.* Tagungen des Landesmuseums für Vorgeschichte Halle, Band 13/I, 2016. Halle. p.161–174.

110. de Pablo, J.F.L., Salazar-García, D.C., Subirà-Galdacano, M.E., de Togores, C.R., Gómez-Puche, M., Richards, M.P. and Esquembre-Bebiá, M.A., 2013. Late Mesolithic burials at Casa Corona (Villena, Spain): direct radiocarbon and palaeodietary evidence of the last forager populations in Eastern Iberia. *Journal of Archaeological Science*, *40*(1), pp.671–680.

111. Boon JJ, de Leeuw JW (1987) Amino acid sequence information in proteins and complex proteinaceous material revealed by pyrolysis-capillary gas chromatography-low and high resolution mass spectrometry. *Journal of Analytical and Applied Pyrolysis* 11: 313–327.

112. Fernández-López de Pablo J, Salazar-García DC, Subirà-Galdacano ME, Roca de Togores C, Gómez-Puche M, Richards MP, Esquembre-Bebiá MA (2013) Late Mesolithic burials at Casa Corona (Villena, Spain): direct radiocarbon and palaeodietary evidence of the last forager populations in Eastern Iberia. *Journal of Archaeological Science* 40: 671-680.

113. http://www.algaeindustrymagazine.com/special-report-spirulina-part-2-first-human-consumption-and-cultivation/

114. Salazar-García DC, Aura JE, Olària CR, Talamo S, Morales JV, Richards MP (2014) Isotopic evidence for the use of marine resources in the Eastern Iberian Mesolithic. *Journal of Archaeological Science* 42: 231–240.

115. Gibaja, J.F., Morell, B., Álvarez, J.A.B., Duboscq, S., Masclans, A., Remolins, G., Roig, J., Martín, A., González, P., Plasencia, J. and Coll, J.M., 2017. The chronology of the Neolithic necropolis Bòbila Maduell-Can Gambús in the Northeast Iberian Peninsula: Dating the Pit Burials cultural horizon and long-range raw material exchange networks. *Radiocarbon*, *59*(6), pp.1713–1736.

116. Fontanals-Coll, M., Subirà, M.E., Bonilla, M.D.Z., Duboscq, S. and Gibaja, J.F., 2015. Investigating palaeodietary and social differences between two differentiated sectors of a Neolithic community, La Bòbila Madurell-Can Gambús (north-east Iberian Peninsula). *Journal of Archaeological Science: Reports*, *3*, pp.160–170.

117. Bicho, N., Detry, C., Price, T.D. and Cunha, E. eds., 2015. *Muge 150th: The 150th Anniversary of the Discovery of Mesolithic Shellmiddens: Volume 1* (Vol. 1). Cambridge Scholars Publishing.

118. Bicho, N., Umbelino, C., Detry, C. and Pereira, T., 2010. The emergence of Muge Mesolithic shell middens in central Portugal and the 8200 cal yr BP cold event. *Journal of Island & Coastal Archaeology*, *5*(1), pp.86–104.

119. Buckley SA (2001) *Chemical Investigations of the Organic Embalming Agents Employed in Ancient Egyptian Mummification*. PhD Thesis (University of Bristol, Bristol, UK).

120. Buckley SA, Evershed RP (2001) The Organic Chemistry of Embalming Agents in Pharaonic and Graeco-Roman Mummies. *Nature* 413(6858): 837–841.

121. Buckley SA, Clark KA, Evershed RP (2004) Complex Organic Chemical Balms of Pharaonic Animal Mummies. *Nature* 431(7006): 294–299.

122. Negri G, Marcuccia MC, Salatinob A, Salatino MLF (2000) Comb and Propolis Waxes from Brazil (States of São Paulo and Paraná), *J. Braz Chem Soc* 11(5): 453–457.

123. Dams, M. and Dams, L., 1977. Spanish rock art depicting honey gathering during the Mesolithic. *Nature*, *268*(5617), pp.228–230.

124. Baales, M., Birker, S. and Mucha, F., 2017. Hafting with beeswax in the Final Palaeolithic: a barbed point from Bergkamen. *antiquity*, *91*(359), pp.1155–1170.

125. Bernardini, F., Tuniz, C., Coppa A., Mancini, L., Dreossi, D., Eichert, D., Turco, G., Biasotto, M., Terrasi, F., De Cesare, N., Hua, Q. and Levchenko, V. 2012. Beeswax as Dental Filling on a Neolithic Human Tooth. *PLoS ONE* 7(9), e44904. doi:10.1371/journal.pone.0044904

126. Roffet-Salque, M., Regert, M., Evershed, R.P., Outram, A.K., Cramp, L.J., Decavallas, O., Dunne, J., Gerbault, P., Mileto, S., Mirabaud, S. and Pääkkönen, M., 2015. Widespread exploitation of the honeybee by early Neolithic farmers. *Nature*, *527*(7577): 226–230.

127. Holden T, Hather JG, Watson JPN (1995) Mesolithic Plant Exploitation at the Roc del Migdia, Catalonia. *Journal of Archaeological Science* 22: 769–778.

128. Boon PI, Bunn SE (1994) Variations in the stable isotope composition of aquatic plants and their implications for food web analysis. *Aquatic Botany* 48: 99–108.

129. Tykot RH (2006) Isotope Analyses and the Histories of Maize. In Staller JE, Tykot RH, Benz BF (eds.), *Histories of Maize: Multidisciplinary Approaches to the Prehistory,Linguistics, Biogeography, Domestication, and Evolution of Maize*, 131–142. AcademicPress (Elsevier).

130. Paso KG, Scott Fogler H (2003) Influence of *n*-Paraffin Composition on the Aging of Wax-Oil Gel Deposits. *American Institute of Chemical Engineers Journal* 49(12): 3241–3252.

131. Himran S, Suwono A, Mansoori GA (1994) Characterization of Alkanes and Paraffin Waxes for Application as Phase Change Energy Storage Medium. *Energy Sources* 16(1): 117–128.

132.Louwe Kooijmans, L.L.P. & P.F.B. Jongste 2006: *Schipluiden, a Neolithic settlement on the Dutch* North Sea coast c. 3500 cal BC, Leiden (Analecta Praehistorica Leidensia 37/38).

133.Louwe Kooijmans (ed.) 2001a: *Hardinxveld-Giessendam Polderweg. Een mesolithisch jachtkamp in het rivierengebied (5500-4450 v.Chr.)*, Amersfoort (Rapportage Archeologische Monumentenzorg 83).

134. Louwe Kooijmans (ed.) 2001b: *Archeologie in de Betuweroute. Hardinxveld-Giessendam De Bruin. Een kampplaats uit het Laat-Mesolithicum en het begin van de Swifterbant-cultuur (5500-4450* v.Chr.), Amersfoort (Rapportage Archeologische Monumentenzorg 88).

135. Merritt Jr. C, Robertson DH (1967) The Analysis of Proteins, Peptides and Amino Acids by Pyrolysis-Gas Chromatography and Mass Spectrometry. *Journal of Gas Chromatography* 5(2): 9698.

136. Patterson JM, Shiue C-Y, Smith Jr. WT (1976) Pyrolysis of Some Sulfur-Containing Amino Acids at 850°C. *Journal of Agricultural and Food Chemistry* 24(5): 988–991.

137. van Loon WMGM, Boon JJ (1991) Qualitative analysis of chlorolignins and lignosulphonates in pulp mill effluents entering the river Rhine using pyrolysis mass spectrometry and pyrolysis-gas chromatography/mass spectrometry. *Journal of Analytical and Applied Pyrolysis* 20: 275–302.

138. Kato S, Kurata T, Ishiguro S, Fujimaki M (1973) Additional Volatile Compounds Produced by Pyrolysis of Sulfur-containing Amino Acids. *Agricultural and Biological Chemistry* 37(7): 1759–1761.

139. Fujimaki M, Kato S, Kurata T (1969) Pyrolysis of Sulfur-containing Amino Acids. *Agricultural and Biological Chemistry* 33(8): 1144–1151.

140. de Bakker H (1978) *Major soils and soil regions in the Netherlands.* Springer, Netherlands.

141. Salminen, R., Tarvainen, T., Demetriades, A., Duris, M., Fordyce, F.M., Gregorauskiene, V., Kahelin, H., Kivisilla, J., Klaver, G., Klein, H. and Larson, J., 1998. *FOREGS geochemical mapping field manual.*

142. van der Plassche O (1995) Periodic Clay Deposition in a Fringing Peat Swamp in the Lower Rhine-Meuse River Area, 5,400–3,400 Cal BC. *Journal of Coastal Research* Special Issue No.17: 95–102.

143. Krairapanond N, DeLaune RD, Patrick Jr. WH (1992) Distribution of organic and reduced sulfur forms in marsh soils of coastal Louisiana. *Organic Geochemistry* 18(4): 489–500.

144. Wieder RK, Lang GE (1988) Cycling of Inorganic and Organic Sulfur in Peat from Big Run Bog, West Virginia. *Biogeochemistry* 5(2): 221–242.

145. Louwe Kooijmans LP (2003) The Hardinxveld sites in the Rhine/Meuse Delta, The Netherlands, 5500-4500 cal BC. in *Mesolithic on the move* (ed. L. Larsson): 608–624.

146. Pospieszny Ł, Sobkowiak-Tabaka I, Frei K M, Hildebrandt-Radke I, Kowalewska-Marszałek H, Krenz-Niedbała M, Osypińska M, Price DT., Stróżyk M, Winiarska-Kabacińska M (2015). Remains of a late Neolithic barrow at Kruszyn. A glimpse of ritual and everyday life in early Corded Ware societies of the Polish Lowland. Praehistorische Zeitschrift 90 (1-2): 185-213.

147. Fischer, A., Jensen, T.Z. T., 2018. Radiocarbon dates for submarine and maritime finds from early prehistory. In *Oceans of Archaeology,* A. Fisher, L. Pedersen Eds. (Jutland Archaeological Society, Højbjerg, 2018), pp. 202-221.
