# Supplementary material for: Human consumption of seaweed and freshwater aquatic plants in ancient Europe
Source: Nat Commun. 2023 Oct 17;14:6192. doi: 10.1038/s41467-023-41671-2 (PMC10582258; doi:10.1038/s41467-023-41671-2)
Supplement: Supplementary file 3 — Description of Additional Supplementary Files [file 41467_2023_41671_MOESM3_ESM.pdf]

Description of Additional Supplementary Files

Supplementary Data 1: Summary of results, all samples.

Supplementary Data 2: C_1 to C_6 alkyl pyrrole compounds present in the Py-GC-MS of the Isbister dental calculus samples.

Supplementary Data 3: C_1 to C_5 alkyl pyrrole compounds present in the Py-GC-MS of natural sources of tetrapyrroles and Isbister dental calculus sample DL5838.

Supplementary Data 4: Protein biomarkers present in the Py-GC-MS of the Isbister dental calculus samples (relative %) and the protein-based amino acid sources from which they derive.

Supplementary Data 5: Chemical information (retention times and mass spectra) on the presence/absence of organic compounds/ biomarkers in all individual samples in this study.
