# Supplementary material for: Human consumption of seaweed and freshwater aquatic plants in ancient Europe
Source: Nat Commun. 2023 Oct 17;14:6192. doi: 10.1038/s41467-023-41671-2 (PMC10582258; doi:10.1038/s41467-023-41671-2)
Supplement: Supplementary file 9 — Reporting Summary [file 41467_2023_41671_MOESM9_ESM.pdf]

nature portfolio

Corresponding author(s): K Hardy and S Buckley

Last updated by author(s): Aug 4, 2022

Reporting Summary

Nature Portfolio wishes to improve the reproducibility of the work that we publish. This form provides structure for consistency and transparency in reporting. For further information on Nature Portfolio policies, see our Editorial Policies and the Editorial Policy Checklist.

Statistics

For all statistical analyses, confirm that the following items are present in the figure legend, table legend, main text, or Methods section.

n/a	Confirmed	
☒	☐	The exact sample size (*n*) for each experimental group/condition, given as a discrete number and unit of measurement
☒	☐	A statement on whether measurements were taken from distinct samples or whether the same sample was measured repeatedly
☒	☐	The statistical test(s) used AND whether they are one- or two-sided<br>*Only common tests should be described solely by name; describe more complex techniques in the Methods section.*
☒	☐	A description of all covariates tested
☒	☐	A description of any assumptions or corrections, such as tests of normality and adjustment for multiple comparisons
☒	☐	A full description of the statistical parameters including central tendency (e.g. means) or other basic estimates (e.g. regression coefficient) AND variation (e.g. standard deviation) or associated estimates of uncertainty (e.g. confidence intervals)
☒	☐	For null hypothesis testing, the test statistic (e.g. *F*, *t*, *r*) with confidence intervals, effect sizes, degrees of freedom and *P* value noted<br>*Give P values as exact values whenever suitable.*
☒	☐	For Bayesian analysis, information on the choice of priors and Markov chain Monte Carlo settings
☒	☐	For hierarchical and complex designs, identification of the appropriate level for tests and full reporting of outcomes
☒	☐	Estimates of effect sizes (e.g. Cohen's *d*, Pearson's *r*), indicating how they were calculated

Our web collection on statistics for biologists contains articles on many of the points above.

Software and code

Policy information about availability of computer code

Data collection	N/A
Data analysis	N/A

For manuscripts utilizing custom algorithms or software that are central to the research but not yet described in published literature, software must be made available to editors and reviewers. We strongly encourage code deposition in a community repository (e.g. GitHub). See the Nature Portfolio guidelines for submitting code & software for further information.

Data

Policy information about availability of data

All manuscripts must include a data availability statement. This statement should provide the following information, where applicable:

- Accession codes, unique identifiers, or web links for publicly available datasets
- A description of any restrictions on data availability
- For clinical datasets or third party data, please ensure that the statement adheres to our policy

All information on the samples and the data generated and analysed in this study are included in the manuscript, supplementary information files and supplementary data files. MassSpecII32 and NIST Mass Spectral Database were used to collect and interpret the data analysed by TD/Py-GC-MS.

Human research participants

Policy information about studies involving human research participants and Sex and Gender in Research.

Reporting on sex and gender	N/A
Population characteristics	N/A
Recruitment	N/A
Ethics oversight	N/A

Note that full information on the approval of the study protocol must also be provided in the manuscript.

Field-specific reporting

Please select the one below that is the best fit for your research. If you are not sure, read the appropriate sections before making your selection.

☐ Life sciences ☒ Behavioural & social sciences ☐ Ecological, evolutionary & environmental sciences

For a reference copy of the document with all sections, see nature.com/documents/nr-reporting-summary-flat.pdf

Behavioural & social sciences study design

All studies must disclose on these points even when the disclosure is negative.

Study description	Integrating archaeological information and organic residue analysis, it has been possible to identify a number of food- and environmentally-related organic material entrapped in dental calculus (tartar). Eighty dental calculus samples were analysed, with soil/sediment controls analysed where available. The study was qualitative and semi-quantitative, primarily based on the presence/absence of organic compounds/biomarkers.
Research sample	The study is based on samples provided from different archaeological sites, in different regions of Europe and from different time periods. Sample procedure can best be described as a 'grab sample'. There is insufficient availability of the dental calculus material from the time periods and geographical regions, for detailed consistency. The samples obtained provide a snapshot approach to the detect materials recovered from dental calculus in the regions and time scales studied.
Sampling strategy	The Scottish samples were taken based on the availability offered by the museums. In other cases, samples were provided by the researchers or excavators.
Data collection	Samples from the Scottish museums were taken using standard procedures. Samples were selected, recorded, photographed, and material extracted using a dental pick. Samples were placed immediately into Eppendorf tubes. All samples from other countries were provided by local researchers. No data is available on their methods of sample collection. There were no preconceptions or expectations on the possible findings of the study. MassSpecII32 and NIST Mass Spectral Database was used to collect and interpret the data analysed by TD/Py-GC-MS.
Timing	The first set of samples (Orkney Museum) were taken in 2013. Following their analysis, and the very unusual (then unique) set of results obtained, it became evident that broader contextual data should be provided. Therefore, further samples were obtained in 2015-6. There was no expectation of results; the aim of expanding the study was to determine whether the Orkney results were specific to the highly maritime environment of the Orkney Islands, or represented a broader geographical behaviour. The start date for collection was May 2013 and the stop date for collection was 2016.
Data exclusions	No data were excluded from the analysis.
Non-participation	No participants were involved in this study.
Randomization	The study involved analysing a set number of samples obtained from a number of archaeological sites using GC-MS and therefore randomization was not relevant in this study. Standards and blanks were used to ensure the integrity of the results obtained, but since there were no preconceptions of what the results might, or might not, be randomization was not relevant to this study.

Reporting for specific materials, systems and methods

We require information from authors about some types of materials, experimental systems and methods used in many studies. Here, indicate whether each material, system or method listed is relevant to your study. If you are not sure if a list item applies to your research, read the appropriate section before selecting a response.

Materials & experimental systems

n/a	Involved in the study
☒	☐ Antibodies
☒	☐ Eukaryotic cell lines
☐	☒ Palaeontology and archaeology
☒	☐ Animals and other organisms
☒	☐ Clinical data
☒	☐ Dual use research of concern

Methods

n/a	Involved in the study
☒	☐ ChIP-seq
☒	☐ Flow cytometry
☒	☐ MRI-based neuroimaging

Palaeontology and Archaeology

Specimen provenance

No permits were required or issued for the samples.
National Museums of Scotland samples were taken in the Museum in November/December 2015 under the supervision of the Principal Curator of Early Prehistory (Dr Alison Sheridan, now retired).
Samples from Orkney were taken at the Orkney Museum, Kirkwall in May 2013, under the supervision of the Curator (Sheila Garson, now retired).
All other samples were taken by other people and provided in 2016 as follows:
Lithuanian samples were provided (by post) from Dr. Zydrune Miliauskiene, Dept. of Anatomy, Histology and Anthropology, Faculty of Medicine, Vilnius University, M.K. Čiurlionio 21, LT-03101 Vilnius, Lithuania.
Kanaljorden, Sweden. The samples were provided (by post) from the excavator of the site: Dr. Fredrik Hallgren, Uppsala University, Sweden.
Iberia. All Iberian samples were provided by Dr. Eulalia Subirats, Universitat Autonoma de Barcelona (by hand).
Netherlands. All samples from the Netherlands were provided (by post) from Dr. Lucy Kubiak Martens, BIAX Consult, Netherlands.
Kruszyn, Poland. Sample was provided (by post) from Dr. Iwona Sobkowiak-Tabaka, Faculty of Archaeology, Adam Mickiewicz University, Uniwersytetu Poznańskiego 7, 61-614 Poznań, Poland.
Rønstenen, Denmark. Sample was provided by Dr. Anders Fischer, Ministry of Culture, Copenhagan, Denmark (now retired).

Specimen deposition

Samples were taken or provided specifically for this study and were entirely used in this study, reflecting the small sample sizes, the nature of the (minimally) destructive analyses and the need to maximise the scientific and archaeological information resulting from the analyses.

Dating methods

Any radiocarbon dates given were from previous publications (see references) and not generated as part of the study presented here.

☐ Tick this box to confirm that the raw and calibrated dates are available in the paper or in Supplementary Information.

Ethics oversight

No ethical approval or guidance was required.

Note that full information on the approval of the study protocol must also be provided in the manuscript.
